# Synthesis of α,β-unsaturated ketones through nickel-catalysed aldehyde-free hydroacylation of alkynes

**DOI:** 10.1038/s42004-022-00633-3

**Published:** 2022-02-03

**Authors:** Joon Ho Rhlee, Saikat Maiti, Jeong Woo Lee, Ho Seung Lee, Ismoili Ahror Bakhtiyorzoda, Soochan Lee, Jaehyun Park, Seok Ju Kang, Yung Sam Kim, Jeong Kon Seo, Kyungjae Myung, Wonyoung Choe, Sung You Hong

**Affiliations:** 1 Department of Chemistry, Ulsan National Institute of Science & Technology (UNIST), Ulsan, Republic of Korea; 2 School of Energy and Chemical Engineering, Ulsan National Institute of Science & Technology (UNIST), Ulsan, Republic of Korea; 3 Center for Genomic Integrity (CGI), Institute for Basic Science (IBS), Ulsan, Republic of Korea; 4 UNIST Central Research Facility (UCRF), Ulsan National Institute of Science & Technology (UNIST), Ulsan, Republic of Korea

**Keywords:** Synthetic chemistry methodology, Homogeneous catalysis

## Abstract

α,β-Unsaturated ketones are common feedstocks for the synthesis of fine chemicals, pharmaceuticals, and natural products. Transition metal-catalysed hydroacylation reactions of alkynes using aldehydes have been recognised as an atom-economical route to access α,β-unsaturated ketones through chemoselective aldehydic C–H activation. However, the previously reported hydroacylation reactions using rhodium, cobalt, or ruthenium catalysts require chelating moiety-bearing aldehydes to prevent decarbonylation of acyl-metal-hydride complexes. Herein, we report a nickel-catalysed anti-Markovnikov selective coupling process to afford non-tethered *E*-enones from terminal alkynes and *S*-2-pyridyl thioesters in the presence of zinc metal as a reducing agent. Utilization of a readily available thioester as an acylating agent and water as a proton donor enables the mechanistically distinctive and aldehyde-free hydroacylation of terminal alkynes. This non-chelation-controlled approach features mild reaction conditions, high step economy, and excellent regio- and stereoselectivity.

## Abstract (web summary)

Transition metal-catalysed hydroacylation of alkynes using aldehydes is an atom-economical route to access α,β-unsaturated ketones, but typically requires aldehydes that bear chelating moieties. Here, the authors report a nickel-catalysed anti-Markovnikov selective coupling process to afford non-tethered *E*-enones from terminal alkynes and *S*-2-pyridyl thioesters.

## Introduction

**Fig. 1:**
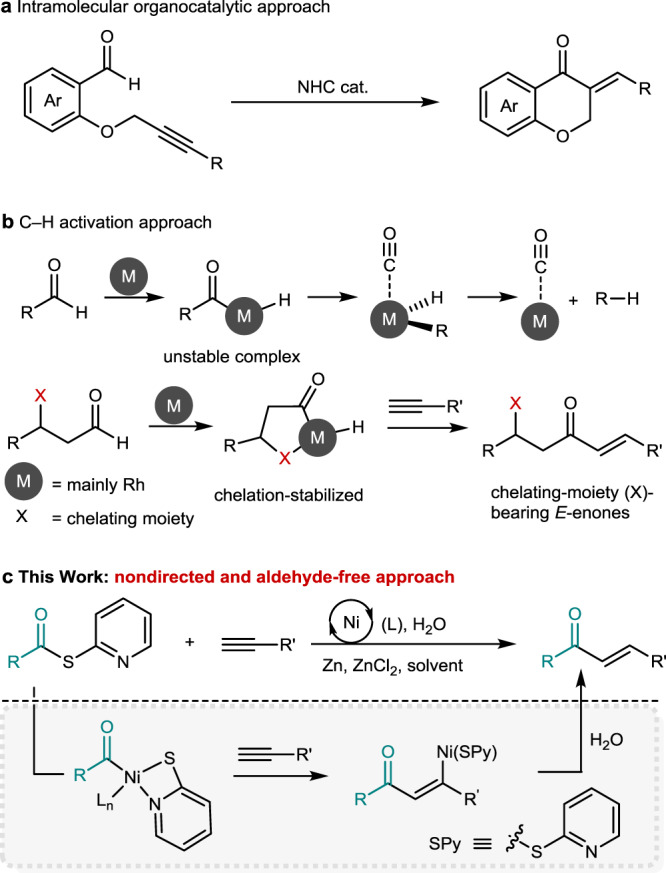
Hydroacylation of alkynes. Previous works: aldehyde-alkyne coupling, **a** Intramolecular organocatalytic approach and **b** C–H activation approach. **c**) This work: nondirected and aldehyde-free approach.

α,β-Unsaturated ketones have been extensively applied as versatile compounds in synthetic organic chemistry, for example, as key substrates for conjugate addition^1–4^, Morita–Baylis–Hillman^5,6^, Diels–Alder^7,8^, and epoxidation^9–11^ reactions. They have been conventionally prepared through aldol condensation^12^, Horner–Wadsworth–Emmons olefination^13^, the dehydrogenation of ketones^14,15^, or palladium-catalyzed carbonylation^16,17^ reactions. However, these methods often suffer from the use of strong bases, elevated reaction temperature, *E*/*Z*-selectivity control, and/or multi-step synthetic operations. Alkynes have been widely applied as readily available synthetic platforms for catalytic transformations to directly access functionalized cyclic or acyclic products^18–22^. The catalytic hydroacylation of alkynes using aldehydes inherently provides an atom-economical process leading to the formation of enones. Organocatalytic intramolecular hydroacylation reactions of alkynes using organophosphines or *N*-heterocyclic carbenes have been demonstrated (Fig. 1a)^23–25^. Metal-catalyzed intermolecular hydroacylation has emerged as a prominent method for rapidly accessing *E*-enones through alkyne–aldehyde coupling^26–31^. This atom-economical method involves the chemoselective activation of an aldehydic C(*sp*^2^)–H, and chelating moiety-bearing aldehydes have been applied to prevent decarbonylative side pathways (Fig. 1b).

Stabilization of an acyl-metal complex assisted by heteroatom chelation is a powerful strategy for obtaining *E*-enones. However, the installation and removal of the coordinating moieties entailed extra synthetic steps while reducing the step economy of the hydrofunctionalization. Though nondirected hydroacylation methods have been developed for alkenes or dienes^32–36^, to our knowledge, there is no general hydroacylation method for unactivated terminal alkynes that lead to chelating moiety-free *E*-enones. Mukaiyama, Weix, and Kishi independently reported that *S*-2-pyridyl (SPy) thioesters act as potential acyl donors for (cross-electrophile) coupling reactions^37–40^. We surmised that the thioester may act as both a transient SPy ligand and an acyl component^41–44^ under nickel-catalyzed reductive coupling conditions to lead to acyl-Ni-SPy complex. A consecutive alkyne insertion and subsequent protodemetalation process may lead to hydroacylation product formation (Fig. 1c). However, the challenges of this anticipated reaction process to attain traceless alkyne hydroacylation are fourfold: (i) competition with non-conjunctive cross-electrophile (proton and thioester) coupling, (ii) reduction of substrates, (iii) iterative alkyne additions, and (iv) regio- and stereoselectivity issues. Therefore, precise reactivity and selectivity controls are crucial for a general approach to the hydroacylation of unactivated terminal alkynes. Herein, we report a nondirected and aldehyde-free approach to afford *E*-enones via a nickel-catalyzed reductive pathway.

## Results and discussion

### Optimization studies

To optimize the reaction conditions, *S*-(pyridin-2-yl) 4-methoxybenzothioate (**1**) and 3,3-dimethyl-1-butyne (**2**) were chosen as model substrates, and a thorough screening of catalysts, reducing agents, additives, and solvents was conducted (Table 1, see also the Supplementary Information (SI), Section III, Supplementary Tables 1–7). The standard conditions were established on the basis of inexpensive nickel(II) perchlorate hexahydrate, Zn, and ZnCl_2_ in 1,2-DME to exclusively afford *E*-enone **3** in 81% isolated yield at room temperature (entry 1). The use of THF resulted in a similar yield (entry 2). Interestingly, coordinated water molecules were also found to be a suitable proton source (entries 3–5). The use of 17 mol% of Ni catalyst was appropriate for providing a stoichiometric 1 equiv of protons to the reaction. No desired product formation was observed in the absence of the nickel catalyst, Zn, or ZnCl_2_ (entries 6–8). Mn as the reducing agent instead of Zn also appeared successful; however, this resulted in a diminished yield (entry 9). ZnCl_2_ was found to be superior to MgCl_2_ (entry 10). The complexation of thioester **1** by ZnCl_2_ was examined by ^1^H NMR spectroscopic studies (see the SI, Section VI). Using 1.5 equiv of terminal alkyne **2** was required for better conversion of the thioester (entry 11). The standard optimized reaction conditions were developed under an inert argon atmosphere; however, a significant amount of product formation was observed even under open atmosphere conditions (entry 12). Reduced reaction time or a decreased amount of Ni catalyst led to diminished yields (entries 13, 14). In addition, the employment of acyl chloride or aldehyde as an acyl donor instead of thioester appeared to be completely unproductive (entries 15, 16). Additional ligands in the hydroacylation resulted in slightly diminished yields for the substrates (entries 17, 18).

**Table 1:** Reaction optimization.


Entry	Change from above conditions	Yield (%)
1	none	87(81)
2	THF instead of 1,2-DME	84(78)
3	Ni(NO_3_)_2_^.^6H_2_O as the catalyst	69
4	NiCl_2_^.^6H_2_O as the catalyst	67
5	NiCl_2_ with H_2_O (3.0 equiv)	54
6	no Ni catalyst	0
7	no Zn	0
8	no ZnCl_2_	0
9	Mn instead of Zn	49
10	MgCl_2_ instead of ZnCl_2_	18
11	1.0 equiv of **2**	41
12	open atmosphere	66
13	12 h	68
14	10 mol% of Ni catalyst	68
15	4-methoxybenzoyl chloride	0
16	4-methoxybenzaldehyde	0
17	2-mercaptopyridine, 20 mol%	65
18	2,2′-dipyridyl disulfide, 20 mol%	(48)

*1,2-DME* 1,2-dimethoxyethane, *PMP* para-methoxyphenyl.

Reaction conditions: **1** (0.20 mmol), **2** (0.30 mmol). Yields were determined by GC using dodecane as the internal standard. Isolated yields are given in parentheses.

### Scope of the reaction

**Fig. 2:**
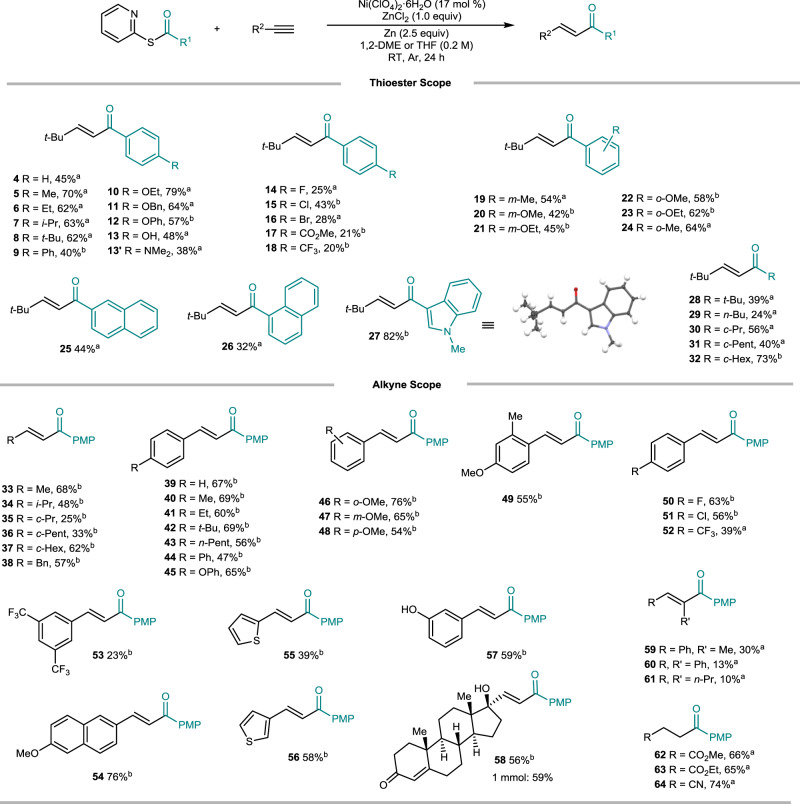
Scope of the nickel-catalyzed hydroacylation. Reaction conditions:^a^ Thioester (0.20 mmol), alkyne (1.5 equiv), 1,2-DME. Reaction conditions:^b^ Thioester (0.20 mmol), alkyne (1.5 equiv), THF, 2,2′-dipyridyl disulfide (20 mol%). Isolated yields.

With the optimized conditions in hand, we set out to explore the generality of this alkyne hydroacylation by determining the thioester scope (Fig. 2, see also Supplementary Figs. 1–5). Various alkyl substituents worked well to afford *E*-enone products (**5**–**8**) in moderate to good yields. Hydrogen and phenyl groups, however, led to slightly lower yields (**4**, **9**). Generally, electron-donating groups gave the products in moderate to good yields (**10**–**13**). The thioester bearing -NMe_2_ gave enone **13**′ in a low yield of 38%, suggesting competition between the Lewis-basic moiety and carbonyl group towards ZnCl_2_ (see the SI, Section VIII-2). Although yields were low due to the oxidative addition capability of Ni(0) species, halide groups were tolerated in the reaction (**14**–**16**). Electron-withdrawing groups such as CO_2_Me and CF_3_ led to products (**17**, **18**) with low yields. Oxidative addition of C(*sp*^*2*^)–halides to Ni(0) or thioester homocoupling^43^ may result in reduced yields. *Meta*- and *ortho*-substitution patterns also provided the products in moderate yields (**19**–**24**). A 2-naphthyl substituent afforded the product a slightly higher yield (44%) than a 1-naphthyl substituent (32%) (**25**, **26**). Upon **26** synthesis, naphthalene byproduct formation via decarbonylation was also observed in 15% yield with the consumption of substrates. An electron-rich indole substituent led to product **27** with an 82% yield. Both acyclic- and cyclic aliphatic substituents could access the products with moderate yields (**28**–**32**).

**Fig. 3:**
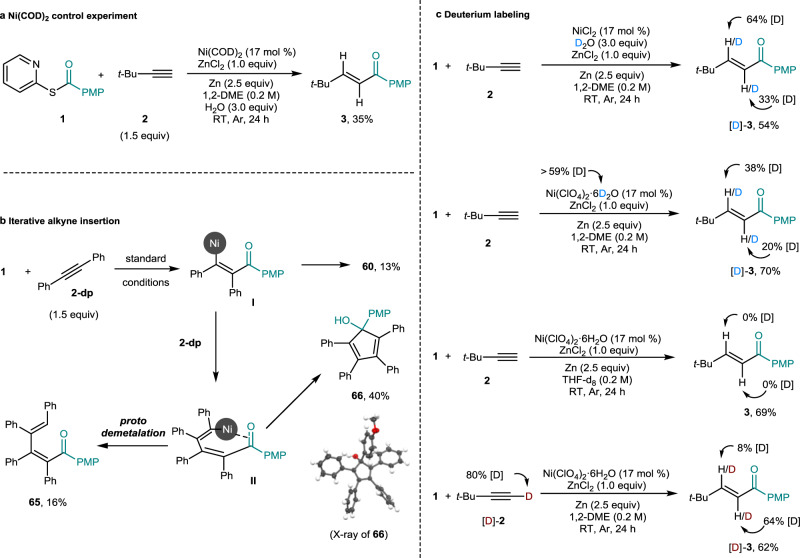
Mechanistic and deuterium labeling studies. **a** Ni(COD)_2_ control experiment. **b** Iterative alkyne insertion. **c** Deuterium labeling.

We then examined the scope of a wide range of alkynes. It is noteworthy that the reaction conditions b in Fig. 2 employ 2,2′-dipyridyl disulfide (Py_2_S_2_) to improve the reaction efficacy. The reduced SPy anion from Py_2_S_2_ may provide an additional ligand source to stabilize nickel complexes (see also Fig. 1c). Acyclic- as well as cyclic aliphatic terminal alkynes, underwent the reaction to afford the corresponding vinyl ketones (**33**–**38**) in moderate yields, although cyclopropyl- and cyclopentyl-derived alkynes gave the diminished yields. Aromatic alkynes bearing alkyl, phenyl, and phenoxy substituents worked well to give the desired products (**39**–**45**) with moderate to good yields. *Ortho-*, *meta*-, and *para*-substituted methoxy groups were also tolerated to obtain the products (**46**–**48**). Product **49** was isolated in 55% yield by using a disubstituted arylalkyne. Fluoro- and chloro-groups were examined and gave **50** and **51** in 63% and 56% yields, respectively. The strongly electron-withdrawing trifluoromethyl group led to product formation (**52**, **53**) with diminished yields. 2-Ethylnyl-6-methoxy-naphthalene efficiently produced **54** in 76% yield. 2-Ethynylthiophene and 3-ethynylthiophene underwent the reaction smoothly to obtain the corresponding products (**55**, **56**) in 39% and 58% yields, respectively. A free hydroxyl group was compatible affording **57** in 59% yield. We were delighted to find that the reaction was feasible with ethisterone, an agent for gynecological disease treatment, to afford **58** in 56% yield. The chemistry was also operative on a 1 mmol scale to give a similar yield. Symmetrical as well as unsymmetrical internal alkynes gave hydroacylation products (**59**–**61**) in low yields. The iterative reactivity yielding double-alkyne-insertion byproducts was observed (see also Fig. 3b). Activated alkenes also underwent the reaction smoothly to afford **62**–**64** in good yields.

### Mechanistic investigations and deuterium labeling experiments

Control experiments were conducted to gain the insight into the reaction pathway. When the reaction was performed using Ni(COD)_2_ as a catalyst, the desired hydroacylation product **3** was obtained in a 35% isolated yield. This study corroborates the oxidative addition of thioester **1** to zero-valent nickel species over the reaction course (Fig. 3a). The formation of hydroacylation product **60** suggests the protodemetalation of nucleophilic vinyl nickel complex **I**^45^. Interestingly, **65** and **66** were also isolated, verifying an iterative double-alkyne-insertion (Fig. 3b) and the presence of vinyl nickel species **II**^46–48^.

**Fig. 4:**
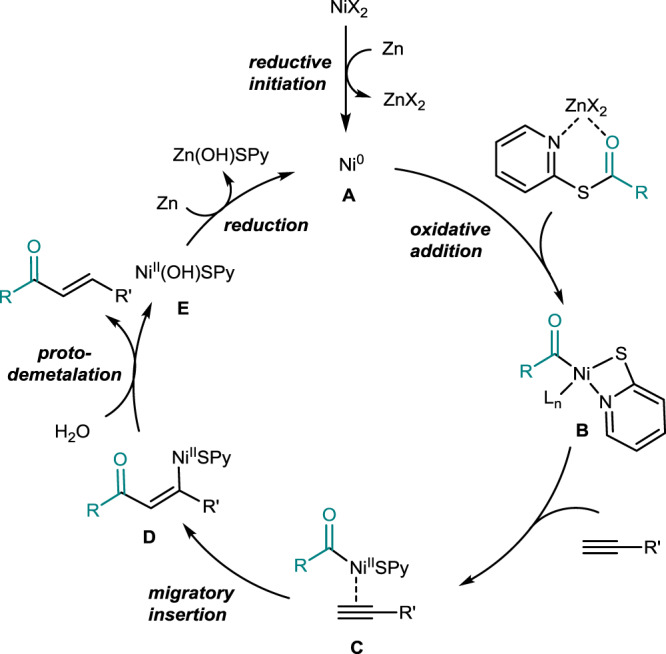
Proposed catalytic cycle. Synthesis of non-tethered *E*-enones via the Ni-catalyzed aldehyde-free alkyne hydroacylation.

To identify the proton source of the hydroacylation, deuterium labeling tests were performed (Fig. 3c, see also Supplementary Figs. 6–11). First, a reaction conducted using deuterium oxide (3.0 equiv) resulted in the formation of the product with 64 and 33% [D] incorporation at the β- and α-positions, respectively, of unsaturated ketone [D]-**3**. π-Complexation of terminal alkynes to transition metal species gives its increased acidity^49,50^. Reversible H–D exchange between alkyne complex **C** (shown in Fig. 4) and D_2_O and subsequent migratory insertion lead to the incorporation of deuterium in the α-position of enone framework (see also the SI, Supplementary Fig. 12). Employing deuterated nickel(II) perchlorate hexahydrate also resulted in the deuterated enone [D]-**3** (see also the SI, Section V). When we also performed the hydroacylation reaction in THF-*d*_8_, enone **3** was obtained with no deuterium labeling. The employment of terminally deuterated alkyne [D]-**2** for the Ni-catalyzed reductive coupling reaction also reveals H/D scrambling behavior.

### Proposed mechanism

Based on the above mechanistic studies and findings from previous reports^45–48,51,52^, we proposed a reaction pathway for this reductive hydroacylation as illustrated in Fig. 4. The reaction is initiated by the generation of Ni(0) species **A** through the reduction of Ni(II) catalyst with the help of zinc. With the assistance of zinc chloride as a Lewis-acidic additive (see also the SI, Section VI, Supplementary Figs. 13 and 14), oxidative addition of electrophilic thioester to Ni(0) can be promoted^53,54^, forming acyl-Ni(II)-X complex **B**. Terminal alkynes approached complex **B** to give Ni(II)-alkyne complex **C**. Migratory insertion of terminal alkynes gives rise to vinyl nickel complex **D**. Then, protodemetalation of nucleophilic vinyl Ni(II) species **D** with the help of water affords the desired *E*-enone^45^. The reduction of Ni(II)(OH)X species **E** by zinc regenerates the active Ni(0) species **A**. Overall, readily available electrophiles (i.e., “H^+^” and “Ac^+^”) are incorporated into terminal alkynes in a reductive fashion^55^ with excellent regio- and *E*-selectivity under mild conditions. This method can avoid the use of strong bases, organometallic reagents, and pre-installed chelating moieties.

## Conclusions

In summary, we have demonstrated a nickel-catalyzed hydroacylation method of terminal alkynes to exclusively afford non-tethered *E*-enones. *S*-(2-Pyridyl) thioesters not only serve as the acyl donor but also give the acyl-Ni(II)-SPy species as a key intermediate over the course of non-chelation-controlled catalytic events. It requires neither additional steps for removal of the coordinating group nor the use of a nucleophilic hydride source, further enhancing the efficacy of the method. We anticipate that these Ni-catalyzed reductive hydroacylation reactions will have an impact on synthesizing important synthetic intermediates, functional materials, and pharmaceuticals.

## Methods

### General experimental procedure for *E*-enones

A 1-dram screw-cap vial equipped with a magnetic stir bar was charged with thioester (0.2 mmol, 1.0 equiv), Zn (33 mg, 0.5 mmol, 2.5 equiv), ZnCl_2_ (27 mg, 0.2 mmol, 1.0 equiv), and Ni(ClO_4_)_2_·6H_2_O (12 mg, 0.034 mmol, 17 mol%) inside a glove box. The mixture was dissolved in 1,2-DME (1 mL). Then, alkyne (0.3 mmol, 1.5 equiv) was added. The reaction mixture was stirred for 24 h at room temperature. After completion of the reaction, the mixture was purified by flash column chromatography to afford the desired product.

## Supplementary information

Supplementary Information

Supplementary Data 1

Supplementary Data 2

Description of Additional Supplementary Files

## Data Availability

Detailed experimental procedures, HRMS-ESI data and NMR spectra (PDF) for all compounds were provided in the Supplementary Information. Single-crystal X-ray data for **27** and **66** (CIF) are available free of charge from the Cambridge Crystallographic Database Centre (CCDC) under reference numbers 2039192 and 2036543.
