# Supplementary material for: Synthesis of α,β-unsaturated ketones through nickel-catalysed aldehyde-free hydroacylation of alkynes
Source: Commun Chem. 2022 Feb 3;5:13. doi: 10.1038/s42004-022-00633-3 (PMC9814684; doi:10.1038/s42004-022-00633-3)
Supplement: Supplementary file 1 — Supplementary Information [file 42004_2022_633_MOESM1_ESM.pdf]

- Supplementary Information -

Synthesis of α,β-unsaturated ketones through nickel-catalysed aldehyde-free hydroacylation of alkynes

[1]Department of Chemistry, Ulsan National Institute of Science & Technology (UNIST), Ulsan, Republic of Korea. [2]School of Energy and Chemical Engineering, Ulsan National Institute of Science & Technology (UNIST), Ulsan, Republic of Korea. [3]Center for Genomic Integrity (CGI), Institute for Basic Science (IBS), Ulsan, Republic of Korea. [4]UNIST Central Research Facility (UCRF), Ulsan National Institute of Science & Technology (UNIST), Ulsan, Republic of Korea. [a]These authors equally contributed to this work.

Corresponding author (S.Y.H.): *e-mail: syhong@unist.ac.kr

Table of Contents

Supplementary Methods

I. General information ………………..………..……….…………………..………… S2

II. Preparation of thioesters ……………………………..……………………………... S2

III. Optimisation studies ………………………..……………..………..…………….. S11

Supplementary Discussion

IV. Experimental procedures …………………………………..………….............. S16

V. Deuterium labelling experiments……………………….……………............. S18

VI. $ZnCl_2$ and ^{1}H NMR study ………………………………..…………..…..…. S24

VII. X-ray single crystal diffraction data …………..………………….…………….. S25

VIII. Characterization data …………………………………….……………..…..…. S28

Supplementary Note

IX. NMR spectra data ………………………………………………………….….. S43

X. Supplementary References …………………………………….……….. S140

Supplementary Methods

I. General information

All reagents were purchased from standard suppliers (Sigma-Aldrich, Alfa Aesar, or TCI) and were used without further purification. The progress of reaction was monitored by thin layer chromatography (Merck, TLC silica gel 60 F_{254} glass plate with 0.25 mm thickness), and visualized by irradiating with an UV lamp (254 nm). Melting points were recorded on a Stuart™ melting point apparatus SMP10 and are uncorrected. Compound purification was carried out through flash column chromatography (Merck silica gel 60, mesh size: 230–400). ^{1}H, ^{13}C and ^{19}F NMR spectra were recorded on a Bruker Avance III HD (400 MHz for ^{1}H, 100 MHz for ^{13}C, 377 MHz for ^{19}F). Chemical shift was given on the δ-scale in ppm, residual solvent peaks were used as an internal standard ($CDCl_3$: 7.26 ppm for ^{1}H NMR, 77.16 ppm for ^{13}C NMR; DMSO: 2.50 ppm for ^{1}H NMR, 39.50 ppm for ^{13}C NMR at 298 K). For ^{19}F NMR, anhydrous α,α,α-trifluorotoluene (-62.61 ppm in $CDCl_3$) was used as an internal standard. ^{1}H and ^{13}C multiplicities were reported as follows: singlet (s), doublet (d), triplet (t), quartet (q), multiplet (m). High resolution mass spectrometry (HRMS) data were recorded by Q Exactive Plus Hybrid Quadrupole-Orbitrap™ mass spectrometer from Thermo Scientific. GC(MS) analyses were conducted by Agilent GC 7890A/B or Agilent 5977B. Functional group analysis was performed by a Spectrum Two™ FT-IR spectrometer by Perkin Elmer with attenuated total reflection (ATR). Air-sensitive reactions were set up inside an argon-filled glovebox.

II. Preparation of thioesters

Thioesters were prepared according to the literature methods.[1–7]

Supplementary Fig. 1. Synthesis of *S*-(pyridin-2-yl) 4-methoxybenzothioate (1)

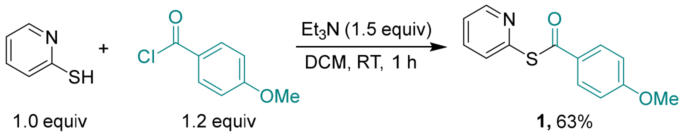

General procedure for synthesizing a thioester (GP-I): To the solution of 2-mercaptopyridine (1.0 g, 9.0 mmol, 1.0 equiv) in DCM (30 mL), 4-methoxybenzoyl chloride (1.8 g, 10.8 mmol, 1.2 equiv) and TEA (1.4 g, 13.5 mmol, 1.5 equiv) were added. The reaction mixture was stirred for 1 h at room temperature. After completion of the reaction, the mixture was diluted with additional DCM (100 mL), and washed with saturated aqueous $NaHCO_3$

solution (50 mL × 2) and brine (50 mL × 2). The organic layer was dried over anhydrous Na_2SO_4 and concentrated *in vacuo*. The residue was purified by flash column chromatography (hexane/EtOAc = 1:1) to afford the title compound **1** (a white solid, 1.38 g, 63%).

Supplementary Fig. 2. Synthesis of *S*-(pyridin-2-yl) cyclopropanecarbothioate (S28)

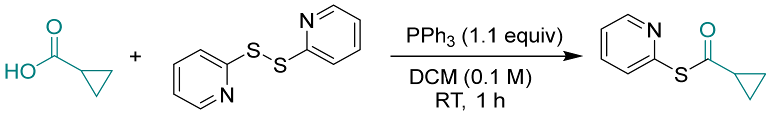

1.0 equiv 1.1 equiv **S28**, 83%

General procedure for synthesizing a thioester (GP-II): In a 5-dram vial equipped with a magnetic stir bar, 2,2´-dipyridyl disulfide (242 mg, 1.1 mmol, 1.1 equiv) and PPh_3 (289 mg, 1.1 mmol, 1.1 equiv) were dissolved in DCM (10 mL). Cyclopropanecarboxylic acid (86 mg, 1.0 mmol, 1.0 equiv) was then added to the solution. The reaction mixture was stirred for 1 h at room temperature. After completion of the reaction, the mixture was diluted with additional DCM (50 mL) and washed with saturated aqueous $NaHCO_3$ solution (30 mL × 2) and brine (30 mL × 2). The organic layer was dried over anhydrous Na_2SO_4 and concentrated *in vacuo*. The residue was purified by flash column chromatography (hexane/EtOAc = 1:1) to afford the title compound **S28** (a yellow oil, 148 mg, 83%).

***S*-(Pyridin-2-yl) 4-methoxybenzothioate (1)**[3]: method **GP-I**; a white solid (1.38 g, 63%); **^{1}H NMR** (400 MHz, $CDCl_3$) δ 8.67 (ddd, *J* = 4.8, 1.9, 0.9 Hz, 1H), 8.00 (d, *J* = 9.0 Hz, 2H), 7.78 (td, *J* = 7.6, 1.9 Hz, 1H), 7.72 (dt, *J* = 7.9, 1.2 Hz, 1H), 7.32 (ddd, *J* = 7.3, 4.9, 1.4 Hz, 1H), 6.97 (d, *J* = 8.9 Hz, 2H), 3.89 (s, 3H); **^{13}C NMR** (100 MHz, $CDCl_3$) δ 187.6, 164.4, 151.6, 150.3, 137.5, 131.2, 130.0, 129.3, 123.7, 114.1, 55.7.

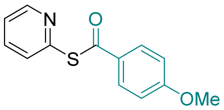

***S*-(Pyridin-2-yl) benzothioate (S2)**[4]: method **GP-I**; a yellow solid (839 mg, 87%); **^{1}H NMR** (400 MHz, $CDCl_3$) δ 8.69 (ddd, *J* = 4.9, 1.9, 0.9 Hz, 1H), 8.03 (dd, *J* = 8.4, 1.2 Hz, 2H), 7.81 (td, *J* = 7.6, 1.9 Hz, 1H), 7.74 (dt, *J* = 7.9, 1.1 Hz, 1H), 7.65 –

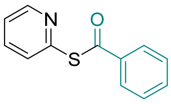

7.60 (m, 1H), 7.50 (t, *J* = 7.7 Hz, 2H), 7.35 (ddd, *J* = 7.4, 4.9, 1.3 Hz, 1H) ; **^{13}C NMR** (100 MHz, $CDCl_3$) δ 189.5, 151.4, 150.6, 137.4, 136.7, 134.1, 131.9, 129.0, 127.7, 123.8.

***S*-(Pyridin-2-yl) 4-methylbenzothioate (S3)**[3]: method **GP-I**; a yellow solid (913 mg, 89%);

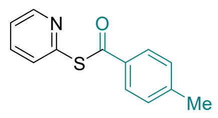

^{1}H NMR (400 MHz, $CDCl_3$) δ 8.68 (d, *J* = 5.8 Hz, 1H), 7.92 (d, *J* = 8.2 Hz, 2H), 7.79 (td, *J* = 7.6, 1.9 Hz, 1H), 7.73 (d, *J* = 7.8 Hz, 1H), 7.33 (ddd, *J* = 7.2, 4.9, 1.2 Hz, 1H), 7.29 (d, *J* = 8.0 Hz, 2H), 2.44 (s, 3H); **^{13}C NMR** (100 MHz, $CDCl_3$) δ 189.0, 151.6, 150.6, 145.1, 137.3, 134.1, 131.0, 129.6, 127.8, 123.7, 21.9.

***S*-(Pyridin-2-yl) 4-ethylbenzothioate (S4)**[5]: method **GP-I**; a white solid (920 mg, 84%);

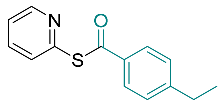

^{1}H NMR (400 MHz, $CDCl_3$) δ 8.67 (ddd, *J* = 4.9, 1.9, 0.9 Hz, 1H), 7.94 (d, *J* = 8.3 Hz, 2H), 7.78 (td, *J* = 7.6, 1.9 Hz, 1H), 7.73 (dt, *J* = 7.8, 1.1 Hz, 1H), 7.36 – 7.28 (m, 3H), 2.72 (q, *J* = 7.6 Hz, 2H), 1.27 (t, *J* = 7.6 Hz, 3H); **^{13}C NMR** (100 MHz, $CDCl_3$) δ 189.0, 151.7, 151.2, 150.6, 137.2, 134.3, 131.0, 128.4, 127.9, 123.6, 29.1, 15.2.

***S*-(Pyridin-2-yl) 4-isopropylbenzothioate (S5)**: method **GP-I**; a white solid (260 mg, 37%);

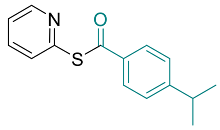

m.p. 92 °C**;** **^{1}H NMR** (400 MHz, $CDCl_3$) δ 8.67 (ddd, *J* = 4.8, 2.0, 0.9 Hz, 1H), 7.96 (d, *J* = 8.4 Hz, 2H), 7.78 (td, *J* = 7.6, 1.9 Hz, 1H), 7.73 (dt, *J* = 7.9, 1.2 Hz, 1H), 7.38 – 7.29 (m, 3H), 2.98 (m, 1H), 1.28 (d, *J* = 6.9 Hz, 6H); **^{13}C NMR** (100 MHz, $CDCl_3$) δ 189.0, 155.8, 151.8, 150.6, 137.2, 134.5, 131.0, 128.0, 127.1, 123.7, 34.5, 23.8; **HRMS (ESI):** m/z $[M+H]^+$ calcd for ($C_{15}H_{16}NOS^+$): 258.09471, found: 258.09467.

***S*-(Pyridin-2-yl) 4-(*tert*-butyl)benzothioate (S6)**[6]: method **GP-I**; a white solid (102 mg, 38%);

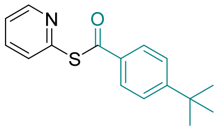

^{1}H NMR (400 MHz, $CDCl_3$) δ 8.68 (ddd, *J* = 4.8, 1.9, 0.9 Hz, 1H), 7.96 (d, *J* = 8.6 Hz, 2H), 7.79 (td, *J* = 7.6, 1.9 Hz, 1H), 7.73 (dt, *J* = 7.9, 1.2 Hz, 1H), 7.51 (d, *J* = 8.6 Hz, 2H), 7.33 (ddd, *J* = 7.3, 4.8, 1.4 Hz, 1H), 1.36 (s, 9H); **^{13}C NMR** (100 MHz, $CDCl_3$) δ 189.0, 157.9, 151.7, 150.6, 137.2, 134.0, 130.9, 127.6, 125.9, 123.6, 35.4, 31.2.

***S*-(Pyridin-2-yl) [1,1'-biphenyl]-4-carbothioate (S7)**: method **GP-I**; a white solid (707 mg, 90%); **m.p.** 154 °C; **^{1}H NMR** (400 MHz, $CDCl_3$) δ 8.70 (d, *J* = 3.9 Hz, 1H), 8.10 (d, *J* = 8.4 Hz, 2H), 7.81 (td, *J* = 7.6, 1.9 Hz, 1H), 7.76 (d, *J* = 7.6 Hz, 1H), 7.72 (d, *J* = 8.6 Hz, 2H), 7.64 (d, *J* = 7.2 Hz, 2H), 7.49 (t, *J* = 7.4 Hz, 2H), 7.42 (t, *J* = 7.3 Hz, 1H), 7.35 (ddd, *J* = 7.2, 4.9, 1.3 Hz, 1H); **^{13}C NMR** (100 MHz, $CDCl_3$) δ 189.0, 151.5, 150.7, 146.8, 139.8, 137.3, 135.4, 131.0, 129.2, 128.6, 128.3, 127.6, 127.4, 123.8; **HRMS (ESI):** m/z $[M+H]^+$ calcd for ($C_{18}H_{14}NOS^+$): 292.07906, found: 292.07892.

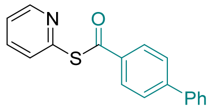

***S*-(Pyridin-2-yl) 4-ethoxybenzothioate (S8):** method **GP-I**; a white solid (619 mg, 53%); **m.p.** 91 °C; **^{1}H NMR** (400 MHz, $CDCl_3$) δ 8.67 (ddd, *J* = 4.9, 1.9, 0.9 Hz, 1H), 7.99 (d, *J* = 8.9 Hz, 2H), 7.78 (td, *J* = 7.6, 1.9 Hz, 1H), 7.73 (dt, *J* = 7.9, 1.1 Hz, 1H), 7.32 (ddd, *J* = 7.3, 4.8, 1.4 Hz, 1H), 6.95 (d, *J* = 8.9 Hz, 2H), 4.12 (q, *J* = 7.0 Hz, 2H), 1.45 (t, *J* = 7.0 Hz, 3H); **^{13}C NMR** (100 MHz, $CDCl_3$) δ 187.8, 163.8, 151.9, 150.5, 137.2, 131.1, 130.0, 129.2, 123.6, 114.6, 64.1, 14.8; **HRMS (ESI):** m/z $[M+H]^+$ calcd for ($C_{14}H_{14}NO_2S^+$): 260.07398, found: 260.07388.

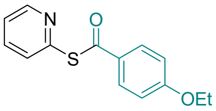

***S*-(Pyridin-2-yl) 4-(benzyloxy)benzothioate (S9):** method **GP-I**; a white solid (466 mg, 54%); **m.p.** 127 °C; **^{1}H NMR** (400 MHz, $CDCl_3$) δ 8.67 (ddd, *J* = 4.8, 1.9, 0.9 Hz, 1H), 8.00 (d, *J* = 8.8 Hz, 2H), 7.78 (td, *J* = 7.6, 1.9 Hz, 1H), 7.72 (d, *J* = 7.9 Hz, 1H), 7.46 – 7.40 (m, 4H), 7.39 – 7.35 (m, 1H), 7.32 (ddd, *J* = 7.3, 4.9, 1.4 Hz, 1H), 7.04 (d, *J* = 8.8 Hz, 2H), 5.15 (s, 2H); **^{13}C NMR** (100 MHz, $CDCl_3$) δ 187.9, 163.5, 151.8, 150.6, 137.2, 136.1, 131.1, 130.0, 129.6, 128.9, 128.5, 127.7, 123.6, 115.0, 70.4; **HRMS (ESI):** m/z $[M+H]^+$ calcd for ($C_{19}H_{16}NO_2S^+$): 322.08963, found: 322.08942.

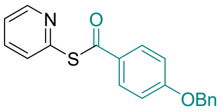

***S*-(Pyridin-2-yl) 4-phenoxybenzothioate (S10):** method **GP-II;** a white solid (271 mg, 88%); **m.p.** 83 °C; **^{1}H NMR** (400 MHz, $CDCl_3$) δ 8.67 (ddd, *J* = 4.9, 1.9, 1.0 Hz, 1H), 8.00 (d, *J* = 8.9 Hz, 2H), 7.78 (td, *J* = 7.6, 1.8 Hz, 1H), 7.72 (dt, *J* = 7.9, 1.1 Hz, 1H), 7.44 – 7.38 (m, 2H), 7.33 (ddd, *J* = 7.3, 4.8, 1.3 Hz, 1H), 7.22 (t, *J* = 7.4 Hz, 1H), 7.09 (dd, *J* = 8.6, 1.1 Hz, 2H), 7.02 (d, *J* = 8.8 Hz, 2H); **^{13}C NMR** (100 MHz, $CDCl_3$) δ 188.0, 162.9, 155.3, 151.6, 150.6, 137.3, 131.0, 131.0, 130.3, 130.0, 125.0, 123.7, 120.5, 117.5; **HRMS (ESI):** m/z $[M+H]^+$ calcd for ($C_{18}H_{14}NO_2S^+$): 308.07398, found: 308.07382.

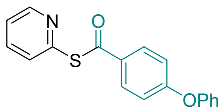

***S*-(Pyridin-2-yl) 4-hydroxybenzothioate (S11):** method **GP-II**; a white solid (165 mg, 72%);

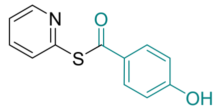

m.p. 156 °C; **^{1}H NMR** (400 MHz, DMSO-d_6) δ 10.68 (br, 1H), 8.63 (ddd, *J* = 4.8, 2.0, 0.9 Hz, 1H), 7.92 (td, *J* = 7.7, 1.9 Hz, 1H), 7.86 (d, *J* = 8.8 Hz, 2H), 7.72 (d, *J* = 7.8 Hz, 1H), 7.47 (ddd, *J* = 7.6, 4.9, 1.2 Hz, 1H), 6.93 (d, *J* = 8.8 Hz, 2H); **^{13}C NMR** (100 MHz, DMSO-d_6) δ 186.6, 163.2, 150.8, 150.4, 137.6, 131.0, 129.8, 126.9, 124.0, 115.8; **HRMS (ESI):** m/z $[M+H]^+$ calcd for ($C_{12}H_{10}NO_2S^+$): 232.04268, found: 232.04259.

***S*-(Pyridin-2-yl) 4-fluorobenzothioate (S12):** method **GP-I**; a white solid (150 mg, 64%);

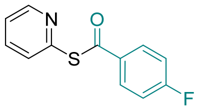

m.p. 90 °C; **^{1}H NMR** (400 MHz, $CDCl_3$) δ 8.68 (ddd, *J* = 4.9, 1.9, 0.9 Hz, 1H), 8.05 (dd, *J* = 8.9, 5.3 Hz, 2H), 7.80 (td, *J* = 7.7, 1.9 Hz, 1H), 7.72 (dt, *J* = 7.8, 1.1 Hz, 1H), 7.35 (ddd, *J* = 7.5, 4.9, 1.2 Hz, 1H), 7.18 (t, *J* = 8.6 Hz, 2H); **^{19}F NMR** (377 MHz, $CDCl_3$) δ -103.5; **^{13}C NMR** (100 MHz, $CDCl_3$) δ 188.0, 166.4 (d, *J* = 255.9 Hz), 151.2, 150.7, 137.4, 133.0 (d, *J* = 3.2 Hz), 131.0, 130.3 (d, *J* = 9.5 Hz), 123.9, 116.2 (d, *J* = 22.2 Hz); **HRMS (ESI):** m/z $[M+H]^+$ calcd for ($C_{12}H_9FNOS^+$): 234.03834, found: 234.03830.

***S*-(Pyridin-2-yl) 4-chlorobenzothioate (S13)**[4]: method **GP-I**; a white solid (744 mg, 66%);

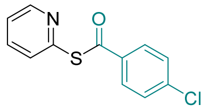

^{1}H NMR (400 MHz, $CDCl_3$) δ 8.69 (ddd, *J* = 4.8, 1.9, 0.9 Hz, 1H), 7.96 (d, *J* = 8.8 Hz, 2H), 7.80 (td, *J* = 7.7, 1.9 Hz, 1H), 7.72 (dt, *J* = 7.8, 1.1 Hz, 1H), 7.48 (d, *J* = 8.8 Hz, 2H), 7.35 (ddd, *J* = 7.5, 4.9, 1.2 Hz, 1H); **^{13}C NMR** (100 MHz, $CDCl_3$) δ 188.4, 151.0, 150.8, 140.5, 137.4, 135.0, 131.0, 129.3, 129.0, 123.9.

***S*-(Pyridin-2-yl) 4-bromobenzothioate (S14)**[3]: method **GP-I**; a white solid (988 mg, 75%);

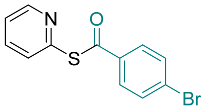

^{1}H NMR (400 MHz, $CDCl_3$) δ 8.68 (ddd, *J* = 4.9, 2.0, 0.9 Hz, 1H), 7.88 (d, *J* = 8.6 Hz, 2H), 7.80 (td, *J* = 7.7, 1.9 Hz, 1H), 7.72 (dt, *J* = 7.9, 1.1 Hz, 1H), 7.64 (d, *J* = 8.6 Hz, 2H), 7.35 (ddd, *J* = 7.5, 4.8, 1.2 Hz, 1H); **^{13}C NMR** (100 MHz, $CDCl_3$) δ 188.6, 151.0, 150.8, 137.4, 135.4, 132.3, 131.0, 129.2, 129.1, 123.9.

Methyl 4-((pyridin-2-ylthio)carbonyl)benzoate (S15): method **GP-I**; a white solid (105 mg,

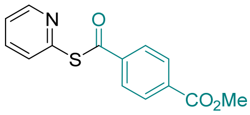

38%); **m.p.** 120 °C; **^{1}H NMR** (400 MHz, $CDCl_3$) δ 8.70 (ddd, *J* = 4.8, 1.9, 0.9 Hz, 1H), 8.16 (d, *J* = 8.7 Hz, 2H), 8.07 (d, *J* = 8.7 Hz, 2H), 7.82 (td, *J* = 7.7, 1.9 Hz, 1H), 7.74 (dt, *J* = 7.9, 1.1 Hz, 1H), 7.37 (ddd, *J* = 7.5,

4.9, 1.2 Hz, 1H), 3.96 (s, 3H); **^{13}C NMR** (100 MHz, $CDCl_3$) δ 189.1, 166.1, 150.9, 150.8, 140.0, 137.5, 134.8, 131.0, 130.2, 127.6, 124.0, 52.7; **HRMS (ESI):** m/z $[M+H]^+$ calcd for ($C_{14}H_{12}NO_3S^+$): 274.05324, found: 274.05301.

***S*-(Pyridin-2-yl) 4-(trifluoromethyl)benzothioate (S16)**[4]: method **GP-I**; a white solid (185 mg, 65%); **^{1}H NMR** (400 MHz, $CDCl_3$) δ 8.70 (ddd, *J* = 4.9, 1.9, 0.9 Hz, 1H), 8.13 (d, *J* = 8.1 Hz, 2H), 7.82 (td, *J* = 7.7, 1.9 Hz, 1H), 7.77 (d, *J* = 8.2 Hz, 2H), 7.74 (dt, *J* = 7.9, 1.0 Hz, 1H), 7.38 (ddd, *J* = 7.6, 4.8, 1.2 Hz, 1H); **^{13}C NMR** (100 MHz, $CDCl_3$) δ 188.8, 150.9, 150.7, 139.5, 137.5, 135.3 (q, *J* = 33.0 Hz), 131.0, 128.0, 126.1 (q, *J* = 3.8 Hz), 124.1, 123.6 (q, *J* = 272.9 Hz).

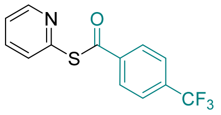

***S*-(Pyridin-2-yl) 3-methylbenzothioate (S17)**: method **GP-I**; a yellow oil (861 mg, 84%); **^{1}H NMR** (400 MHz, $CDCl_3$) δ 8.68 (ddd, *J* = 4.9, 1.9, 1.0 Hz, 1H), 7.84 (s, 1H), 7.82 – 7.81 (m, 1H), 7.79 (td, *J* = 7.6, 1.9 Hz, 1H), 7.73 (dt, *J* = 7.9, 1.1 Hz, 1H), 7.43 (d, *J* = 7.2 Hz, 1H), 7.40 – 7.35 (m, 1H), 7.33 (ddd, *J* = 7.4, 4.9, 1.3 Hz, 1H), 2.43 (s, 3H); **^{13}C NMR** (100 MHz, $CDCl_3$) δ 189.6, 151.6, 150.6, 138.9, 137.3, 136.7, 134.8, 131.0, 128.8, 128.1, 124.9, 123.7, 21.5; **HRMS (ESI):** m/z $[M+H]^+$ calcd for ($C_{13}H_{12}NOS^+$): 230.06341, found: 230.06331.

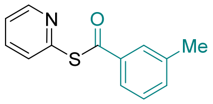

***S*-(Pyridin-2-yl) 3-methoxybenzothioate (S18)**[4]: method **GP-I**; a yellow oil (1.28 g, 97%); **^{1}H NMR** (400 MHz, $CDCl_3$) δ 8.68 (ddd, *J* = 4.9, 2.0, 0.9 Hz, 1H), 7.80 (td, *J* = 7.6, 1.9 Hz, 1H), 7.73 (dt, *J* = 8.0, 1.1 Hz, 1H), 7.64 (dt, *J* = 7.7, 1.1 Hz, 1H), 7.51 (dd, *J* = 2.6, 1.6 Hz, 1H), 7.40 (t, *J* = 8.0 Hz, 1H), 7.34 (ddd, *J* = 7.4, 4.8, 1.3 Hz, 1H), 7.16 (ddd, *J* = 8.3, 2.6, 0.9 Hz, 1H), 3.86 (s, 3H); **^{13}C NMR** (100 MHz, $CDCl_3$) δ 189.1, 159.7, 151.2, 150.4, 137.7, 137.1, 130.7, 129.8, 123.6, 120.2, 120.0, 111.8, 55.8.

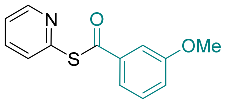

***S*-(Pyridin-2-yl) 3-ethoxybenzothioate (S19)**: method **GP-II**; a colorless oil (161 mg, 62%); **^{1}H NMR** (400 MHz, $CDCl_3$) δ 8.68 (d, *J* = 4.8 Hz, 1H), 7.79 (td, *J* = 7.6, 1.8 Hz, 1H), 7.72 (d, *J* = 7.9 Hz, 1H), 7.62 (d, *J* = 7.7 Hz, 1H), 7.49 (s, 1H), 7.39 (t, *J* = 8.0 Hz, 1H), 7.36 – 7.31 (m, 1H), 7.14 (dd, *J* = 8.3, 2.6 Hz, 1H), 4.08 (q, *J* = 6.9 Hz, 2H), 1.44 (t, *J* = 7.0 Hz, 3H); **^{13}C NMR** (100 MHz, $CDCl_3$) δ 189.4, 159.3,

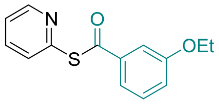

151.5, 150.7, 138.0, 137.3, 131.0, 130.0, 123.8, 121.0, 120.1, 112.5, 63.9, 14.9; **HRMS (ESI):** m/z $[M+H]^+$ calcd for ($C_{14}H_{14}NO_2S^+$): 260.07398, found: 260.07382.

***S*-(Pyridin-2-yl) 2-methoxybenzothioate (S20)**[4]: method **GP-I**; a yellow oil (472 mg, 71%);

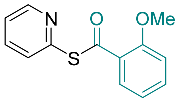

^{1}H NMR (400 MHz, $CDCl_3$) δ 8.65 (ddd, J = 4.8, 1.8, 1.1 Hz, 1H), 7.85 (dd, J = 8.0, 1.6 Hz, 1H), 7.77 – 7.71 (m, 2H), 7.54 – 7.47 (m, 1H), 7.29 (ddd, J = 6.7, 4.9, 2.0 Hz, 1H), 7.04 – 7.00 (m, 2H), 3.95 (s, 3H); **^{13}C NMR** (100 MHz, $CDCl_3$) δ 188.3, 158.4, 152.6, 150.5, 137.1, 134.4, 130.9, 130.1, 126.2, 123.5, 120.7, 112.3, 56.0.

***S*-(Pyridin-2-yl) 2-ethoxybenzothioate (S21)**: method **GP-II**; a yellow oil (227 mg, 87%);

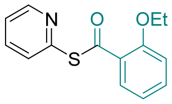

^{1}H NMR (400 MHz, $CDCl_3$) δ 8.67 (d, J = 6.6 Hz, 1H), 7.87 (dd, J = 8.2, 1.8 Hz, 1H), 7.77 (td, J = 7.5, 1.9 Hz, 1H), 7.72 (dt, J = 7.8, 1.2 Hz, 1H), 7.51 – 7.45 (m, 1H), 7.30 (ddd, J = 7.2, 4.9, 1.5 Hz, 1H), 7.04 – 6.96 (m, 2H), 4.23 (q, J = 7.0 Hz, 2H), 1.56 (t, J = 7.0 Hz, 3H); **^{13}C NMR** (100 MHz, $CDCl_3$) δ 188.3, 158.2, 152.9, 150.4, 137.1, 134.5, 131.1, 130.2, 126.2, 123.5, 120.6, 113.1, 65.1, 14.9; **HRMS (ESI):** m/z $[M+H]^+$ calcd for ($C_{14}H_{14}NO_2S^+$): 260.07398, found: 260.07391.

***S*-(Pyridin-2-yl) 2-methylbenzothioate (S22)**: method **GP-I**; a yellow oil (631 mg, 61%);

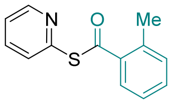

^{1}H NMR (400 MHz, $CDCl_3$) δ 8.70 (ddd, J = 4.9, 1.9, 0.9 Hz, 1H), 7.95 (dd, J = 7.8, 1.4 Hz, 1H), 7.80 (td, J = 7.7, 1.9 Hz, 1H), 7.73 (dt, J = 7.9, 1.1 Hz, 1H), 7.44 (td, J = 7.5, 1.3 Hz, 1H), 7.36 – 7.26 (m, 3H), 2.51 (s, 3H); **^{13}C NMR** (100 MHz, $CDCl_3$) δ 191.3, 152.0, 150.6, 137.8, 137.4, 136.6, 132.4, 132.0, 130.9, 129.0, 126.1, 123.8, 20.9; **HRMS (ESI):** m/z $[M+H]^+$ calcd for ($C_{13}H_{12}NOS^+$): 230.06341, found: 230.06334.

***S*-(Pyridin-2-yl) naphthalene-2-carbothioate (S23)**[4]: method **GP-I**; a white solid (794 mg, 67%);

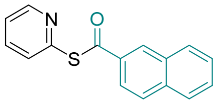

^{1}H NMR (400 MHz, $CDCl_3$) δ 8.71 (ddd, J = 4.9, 1.9, 1.0 Hz, 1H), 8.62 (s, 1H), 8.04 – 8.00 (m, 2H), 7.93 (d, J = 8.8 Hz, 1H), 7.90 (d, J = 8.3 Hz, 1H), 7.85 – 7.80 (m, 1H), 7.78 (dt, J = 7.9, 1.6 Hz, 1H), 7.66 – 7.57 (m, 2H), 7.36 (ddd, J = 7.0, 4.8, 1.6 Hz, 1H); **^{13}C NMR** (100 MHz, $CDCl_3$) δ 189.3, 151.5, 150.6, 137.3, 136.0, 133.9, 132.5, 131.0, 129.7, 129.4, 128.9, 128.8, 127.9, 127.1, 123.7, 123.2.

S-(Pyridin-2-yl) naphthalene-1-carbothioate (S24): method **GP-I**; a yellow solid (569 mg, 79%); **m.p.** 55 °C; **^{1}H NMR** (400 MHz, $CDCl_3$) δ 8.74 – 8.70 (m, 1H), 8.55 (d, J = 8.0 Hz, 1H), 8.24 (dd, J = 7.3, 1.2 Hz, 1H), 8.06 (d, J = 8.3 Hz, 1H), 7.90 (dd, J = 7.5, 2.0 Hz, 1H), 7.87 – 7.81 (m, 2H), 7.62 – 7.53 (m, 3H), 7.37 (ddd, J = 6.0, 4.9, 2.6 Hz, 1H); **^{13}C NMR** (100 MHz, $CDCl_3$) δ 191.4, 152.0, 150.7, 137.4, 134.5, 133.9, 133.7, 130.8, 129.4, 128.5, 128.5, 128.3, 126.9, 125.3, 124.6, 123.8; **HRMS (ESI):** m/z $[M+H]^+$ calcd for ($C_{16}H_{12}NOS^+$): 266.06341, found: 266.06320.

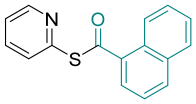

S-(Pyridin-2-yl) pyridine-2-carbothioate (S25)[4]: method **GP-II**; a white solid (101 mg, 38%); **^{1}H NMR** (400 MHz, $CDCl_3$) δ 8.66 (ddd, J = 4.9, 1.9, 1.0 Hz, 1H), 8.27 – 8.21 (m, 1H), 7.98 (s, 1H), 7.83 – 7.80 (m, 1H), 7.80 – 7.75 (m, 1H), 7.38 – 7.28 (m, 4H), 3.88 (s, 3H); **^{13}C NMR** (100 MHz, $CDCl_3$) δ 181.0, 152.3, 150.4, 137.5, 137.0, 135.3, 130.8, 125.8, 123.7, 123.2, 123.0, 122.1, 115.0, 110.0, 33.8.

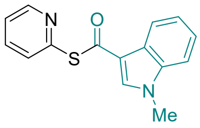

S-(Pyridin-2-yl) 2,2-dimethylpropanethioate (S26): method **GP-I**; a yellow oil (843 mg, 96%); **^{1}H NMR** (400 MHz, $CDCl_3$) δ 8.63 (ddd, J = 4.9, 1.9, 0.9 Hz, 1H), 7.73 (td, J = 7.7, 1.9 Hz, 1H), 7.56 (dt, J = 7.9, 1.0 Hz, 1H), 7.28 (ddd, J = 7.7, 5.0, 1.3 Hz, 1H), 1.33 (s, 9H); **^{13}C NMR** (100 MHz, $CDCl_3$) δ 203.9, 151.9, 150.5, 137.2, 131.0, 123.5, 47.5, 27.5; **HRMS (ESI):** m/z $[M+H]^+$ calcd for ($C_{10}H_{14}NOS^+$): 196.07906, found: 196.07903.

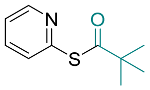

S-(Pyridin-2-yl) pentanethioate (S27)[7]: method **GP-I**; a yellow oil (483 mg, 55%); **^{1}H NMR** (400 MHz, $CDCl_3$) δ 8.62 (ddd, J = 4.8, 2.0, 0.9 Hz, 1H), 7.73 (td, J = 7.7, 1.9 Hz, 1H), 7.61 (dt, J = 7.9, 1.1 Hz, 1H), 7.32 – 7.24 (m, 1H), 2.70 (t, J = 7.4 Hz, 2H), 1.78 – 1.65 (m, 2H), 1.48 – 1.32 (m, 2H), 0.93 (t, J = 7.3 Hz, 3H); **^{13}C NMR** (100 MHz, $CDCl_3$) δ 196.6, 151.7, 150.4, 137.1, 130.2, 123.5, 44.0, 27.5, 22.2, 13.8.

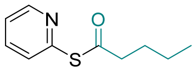

S-(Pyridin-2-yl) cyclopropanecarbothioate (S28)[2]: method **GP-II**; a yellow oil (148 mg, 83%); **^{1}H NMR** (400 MHz, $CDCl_3$) δ 8.62 (ddd, J = 4.9, 2.0, 0.9 Hz, 1H), 7.73 (td, J = 7.7, 1.9 Hz, 1H), 7.63 (dt, J = 7.9, 1.1 Hz, 1H), 7.29 – 7.26 (m, 1H), 2.13 (tt, J = 7.9, 4.5 Hz, 1H), 1.28 – 1.22 (m, 2H), 1.08 – 1.01 (m, 2H); **^{13}C NMR** (100 MHz, $CDCl_3$) δ 196.6, 151.7, 150.4, 137.1, 130.3, 123.5, 22.9, 11.5.

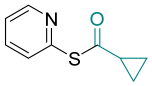

***S*-(Pyridin-2-yl) cyclopentanecarbothioate (S29)**: method **GP-II;** a yellow oil (177 mg, 85%); **^{1}H NMR** (400 MHz, $CDCl_3$) δ 8.61 (ddd, *J* = 4.9, 2.0, 0.9 Hz, 1H), 7.72 (td, *J* = 7.7, 1.9 Hz, 1H), 7.61 (dt, *J* = 7.9, 1.1 Hz, 1H), 7.29 – 7.24 (m, 1H), 3.12 (m, 1H), 2.02 – 1.86 (m, 4H), 1.77 – 1.58 (m, 4H); **^{13}C NMR** (100 MHz, $CDCl_3$) δ 200.1, 152.0, 150.5, 137.1, 130.3, 123.5, 53.6, 30.7, 26.0; **HRMS (ESI):** m/z $[M+H]^+$ calcd for ($C_{11}H_{14}NOS^+$): 208.07906, found: 208.07888.

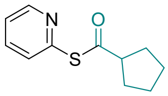

***S*-(Pyridin-2-yl) cyclohexanecarbothioate (S30)**[4]: method **GP-I**; a yellow solid (850 mg, 85%); **^{1}H NMR** (400 MHz, $CDCl_3$) δ 8.62 (ddd, *J* = 4.8, 1.9, 0.9 Hz, 1H), 7.72 (td, *J* = 7.7, 1.9 Hz, 1H), 7.60 (dt, *J* = 7.9, 1.0 Hz, 1H), 7.29 – 7.25 (m, 1H), 2.63 (tt, *J* = 11.4, 3.6 Hz, 1H), 2.07 – 1.99 (m, 2H), 1.86 – 1.78 (m, 2H), 1.71 – 1.64 (m, 1H), 1.59 – 1.48 (m, 2H), 1.38 – 1.21 (m, 3H); **^{13}C NMR** (100 MHz, $CDCl_3$) δ 199.9, 151.8, 150.4, 137.1, 130.4, 123.4, 53.0, 29.5, 25.6, 25.5.

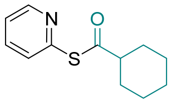

***S*-(Pyridin-2-yl) 4-(N,N-methylamino)benzenecarbothioate (S31)**: Thi thioester **S31** was prepared according to the literature procedure.[4]

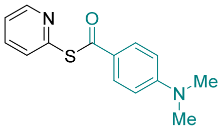

III. Optimisation studies

Supplementary Table 1. Reaction optimisations[a]

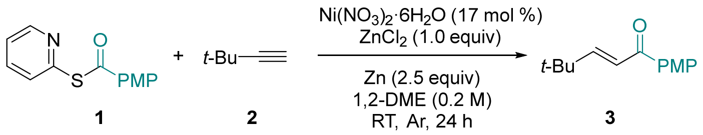

Entry	Change from above conditions	Yield (%)
1	None	69
2	[Ni] 5 mol %, alkyne (1.0 equiv), thioester (1.0 equiv)	13
3	[Ni] 10 mol %, alkyne (1.0 equiv), thioester (1.0 equiv)	43
4	[Ni] 10 mol %, alkyne (1.0 equiv), thioester (1.5 equiv)	57
5	[Ni] 10 mol %, alkyne (1.5 equiv), thioester (1.0 equiv)	64
6	Zn (1.0 equiv)	33
7	Zn (1.5 equiv)	48
8	Zn (2.0 equiv)	58
9	Zn (2.5 equiv)	60
10	$ZnCl_2$ (0.5 equiv)	55
11	$ZnCl_2$ (1.0 equiv)	58
12	$ZnCl_2$ (1.5 equiv)	52

[a]**1** (0.20 mmol), **2** (0.30 mmol), 1,2-DME (0.2 M), the product yields were determined by GC using dodecane as the internal standard. 1,2-DME = 1,2-dimethoxyethane; PMP = *para*-methoxyphenyl.

Supplementary Table 2. Screening of nickel catalysts

i) Without water[a]

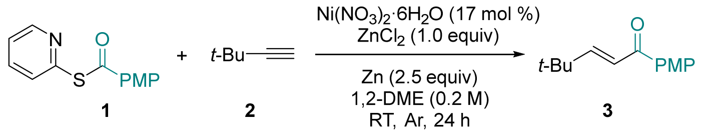

Entry	Change from above conditions	Yield (%)
1	None	69
2	$NiBr_2$·dme	23
3	$NiCl_2$·glyme	56
4	$Ni(ClO_4)_2$·$6H_2O$	(81)
5	$NiBr_2$·$3H_2O$	40
6	$Ni(OAc)_2$·$4H_2O$	72
7	NiO_2·xH_2O	0
8	$NiCl_2$·$6H_2O$	67
9	$NiBr_2$	15
10	$NiCl_2$	0

[a]**1** (0.20 mmol), **2** (0.30 mmol), 1,2-DME (0.2 M), the product yields were determined by GC using dodecane as the internal standard. Isolated yield is given in parentheses.

ii) With water (3.0 equiv)[b]

Entry	Change from above conditions	Yield (%)
1	$NiBr_2$·dme	54
2	$NiCl_2$·glyme	75
3	$Ni(ClO_4)_2$·$6H_2O$ (10 mol %)	71
4	$NiBr_2$·$3H_2O$	79
5	$Ni(OAc)_2$·$4H_2O$	73
6	$Ni(acac)_2$	80
7	$NiCl_2$·$6H_2O$	74
8	$NiBr_2$	66
9	$NiCl_2$	54
10	$Ni(OTf)_2$	69

[b]**1** (0.20 mmol), **2** (0.30 mmol), 1,2-DME (0.2 M).

Supplementary Table 3. Solvent screening[a]

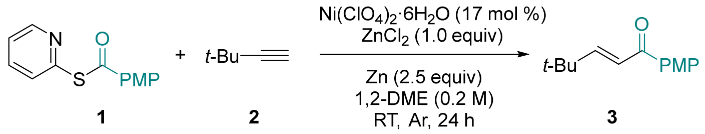

Entry	Change from above conditions	Yield (%)
1	H_2O	0
2	dioxane	64
3	1,2,-DME	87(81)
4	*N,N*-dimethylformamide	55
5	*N,N*-dimethylacetamide	52
6	DCM	0
7	2,2,2-trifluoroethanol	0
8	acetonitrile	59
9	1,1,1,3,3,3-hexafluoro-2-propanol	0
10	THF	84(78)
11	THF/chloroform = 1:1	<10

[a]**1** (0.20 mmol), **2** (0.30 mmol), 1,2-DME (0.2 M), the product yields were determined by GC using dodecane as the internal standard. Isolated yields were given in parentheses.

Supplementary Table 4. Additional screening[a]

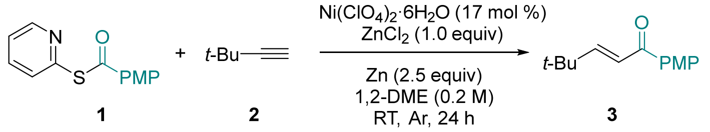

Entry	Change from above conditions	Yield (%)
1	$MgCl_2$ instead of $ZnCl_2$	18
2	ZnF_2 instead of $ZnCl_2$	13
3	$Zn(OTf)_2$ instead of $ZnCl_2$	4
4	$CoCl_2$ instead of $ZnCl_2$	38
5	Mn instead of Zn	49
6	50 °C instead of 25 °C	49
7	open atmosphere	66
8	[Ni] 10 mol %	68
9	12 h instead of 24 h	68

[a] **1** (0.20 mmol), **2** (0.30 mmol), 1,2-DME (0.2 M), the product yields were determined by GC using dodecane as the internal standard.

Supplementary Table 5. Additional screening for aryl alkyne **2´**[a]

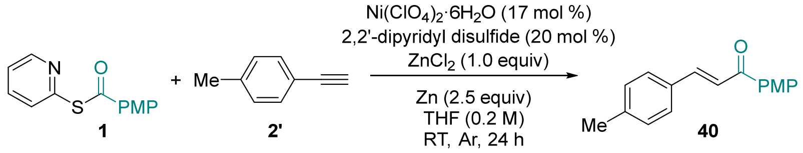

Entry	Change from above conditions	Yield (%)
1	None	69
2	1,2-DME instead of THF	55
3	w/o 2,2′-dipyridyl disulfide	50
4	1,2-DME instead of THF and w/o 2,2′-dipyridyl disulfide	39
5	$NiCl_2·6H_2O$ instead of $Ni(ClO_4)_2·6H_2O$	35
6	2-mercaptopyridine instead of 2,2′-dipyridyl disulfide	47
7	10 mol % of 2,2′-dipyridyl disulfide	53

[a]**1** (0.20 mmol), **2** (0.30 mmol), THF (0.2 M), isolated yields.

Supplementary Table 6. Ligand screening[a]

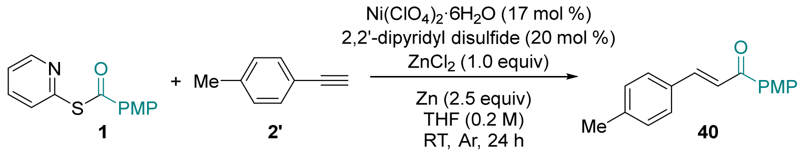

Entry	Change from above conditions	Yield (%)
1	None	69
2	**L1**	47
3	**L2**	36
4	**L3**	36
5	**L4**	41
6	**L5**	37
7	**L6**	39
8	**L7**	37
9	**L8**	26
10	**L9**	22

[a]**1** (0.20 mmol), **2** (0.30 mmol), THF (0.2 M), isolated yields.

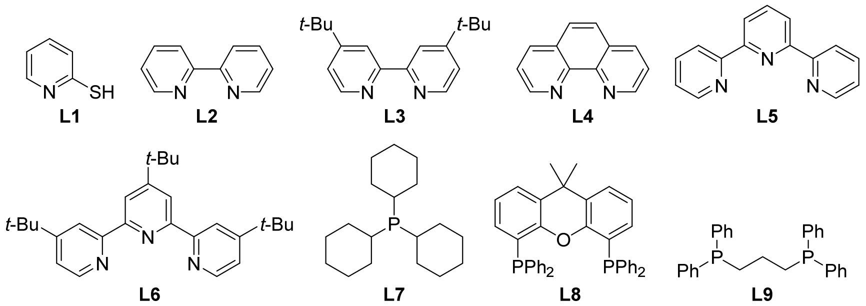

Supplementary Table 7. Screening of H-sources

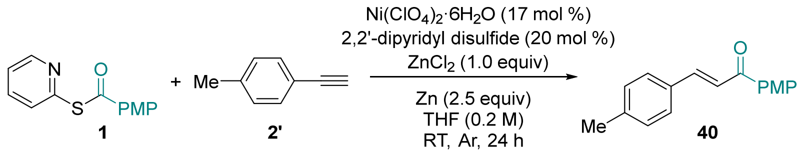

Entry	Change from above conditions	Yield (%)[a]
1	+ acetic acid (1.0 equiv)	47
2	+ NH_4Cl (1.0 equiv)	47
3	+ diphenyl acetic acid (1.0 equiv)	29
4	+ formic acid (1.0 equiv)	55
5	+ triethylsilane (1.0 equiv)	25

[a] **1** (0.20 mmol), **2** (0.30 mmol), THF (0.2 M), the product yields were determined by GC using dodecane as the internal standard.

Supplementary Discussion

IV. Experimental procedures

Supplementary Fig. 3. Synthesis of (*E*)-1-(4-methoxyphenyl)-4,4-dimethylpent-2-en-1-one (3)

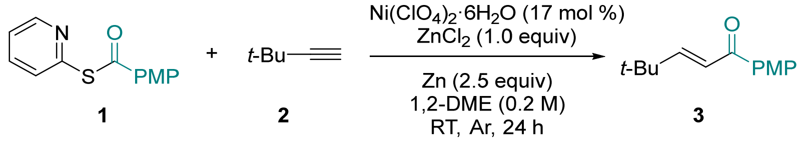

General procedure III (GP-III): A 1-dram screw-cap vial equipped with a magnetic stir bar was charged with *S*-(pyridin-2-yl) 4-methoxybenzothioate (**1**) (49 mg, 0.2 mmol, 1.0 equiv), Zn (33 mg, 0.5 mmol, 2.5 equiv), $ZnCl_2$ (27 mg, 0.2 mmol, 1.0 equiv), and $Ni(ClO_4)_2{\cdot}6H_2O$ (12 mg, 0.034 mmol, 17 mol %) inside a glove box. The mixture was dissolved in 1,2-DME (1 mL). Then, 3,3-dimethyl-1-butyne (**2**) (25 mg, 0.3 mmol, 1.5 equiv) was added. The reaction mixture was stirred for 24 h at room temperature. After completion of the reaction, the mixture was purified by flash column chromatography (hexane/EtOAc = 95:5) to afford title compound **3** (a colorless oil, 35 mg, 81%).

Supplementary Fig. 4. Synthesis of (*E*)-1-(4-methoxyphenyl)-3-(*p*-tolyl)prop-2-en-1-one (40)

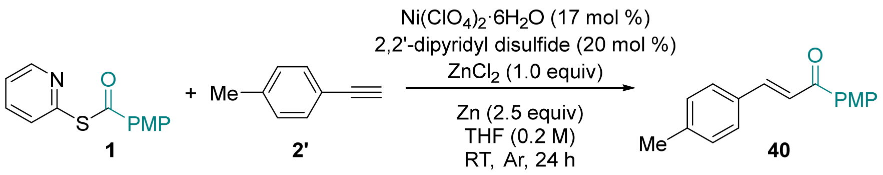

General procedure IV (GP-IV): A 1-dram screw-cap vial equipped with a magnetic stir bar was charged with *S*-(pyridin-2-yl) 4-methoxybenzothioate (**1**) (49 mg, 0.2 mmol, 1.0 equiv), 2,2′-dipyridiyl disulfide (9 mg, 0.04 mmol, 20 mol %), Zn (33 mg, 0.5 mmol, 2.5 equiv), $ZnCl_2$ (27 mg, 0.2 mmol, 1.0 equiv) and $Ni(ClO_4)_2 \cdot 6H_2O$ (12 mg, 0.034 mmol, 17 mol %) sequentially inside a glove box. The mixture was dissolved in THF (1 mL). Then, 4-ethynyltoluene (**2′**) (35 mg, 0.3 mmol, 1.5 equiv) was added. The reaction mixture was stirred for 24 h at room temperature. After completion of the reaction, the mixture was purified by flash column chromatography (hexane/EtOAc = 9:1) to afford the title compound **40** (a white solid, 35 mg, 69%).

Supplementary Fig. 5. 1.0 mmol scale synthesis of compound 58

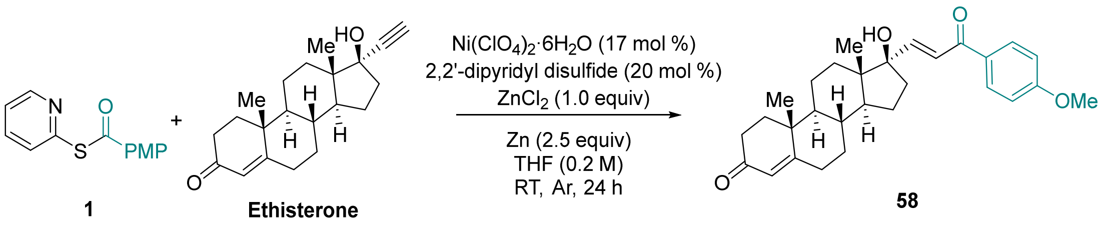

Experimental procedure (1.0 mmol): A 4-dram screw-cap vial equipped with a magnetic stir bar was charged with *S*-(pyridin-2-yl) 4-methoxybenzothioate (**1**) (245 mg, 1.0 mmol, 1.0 equiv), 2,2′-dipyridiyl disulfide (44 mg, 0.2 mmol, 20 mol %), Zn (163 mg, 2.5 mmol, 2.5 equiv), ethisterone (469 mg, 1.5 mmol, 1.5 equiv), $ZnCl_2$ (136 mg, 1.0 mmol, 1.0 equiv) and $Ni(ClO_4)_2 \cdot 6H_2O$ (62 mg, 0.17 mmol, 17 mol %) inside a glove box. The mixture was dissolved in THF (5 mL). The reaction mixture was vigorously stirred for 24 h at room temperature. After completion of the reaction, the mixture was purified by flash column chromatography (hexane/EtOAc = 1:1) to afford the title compound **58** (a white solid, 263 mg, 59%).

V. Deuterium labeling experiments

V-1. Preparation of $Ni(ClO_4)_2 \cdot 6D_2O$

Supplementary Fig. 6. Preparation of $Ni(ClO_4)_2 \cdot 6D_2O$

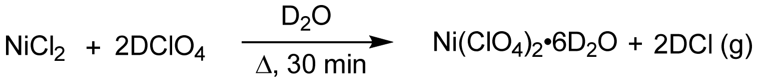

$Ni(ClO_4)_2 \cdot 6D_2O$ was prepared through the modified method from the described literature protocols.[8] In a round bottom flask, $NiCl_2$ (65 mg, 0.5 mmol, 1.0 equiv) was dissolved in D_2O (5 mL), and then $DClO_4$ (68%) (90 µL, 1.0 mmol, 2.0 equiv) was added to the mixture dropwise at 0 °C. The mixture was stirred at 80 °C until DCl gas is no longer released. The mixture was cooled down and concentrated *in vacuo*. The mixture was washed with anhydrous hexane (5 mL × 3) and dried inside a vacuum oven for 24 h at 50 °C to afford $Ni(ClO_4)_2 \cdot 6D_2O$ (a green crystal, 165 mg, 90%).

The FT-IR spectra of $Ni(ClO_4)_2 \cdot 6H_2O$ and $Ni(ClO_4)_2 \cdot 6D_2O$ are expected to show significant differences due to the difference in the frequency of the O–H stretch (~3490 cm^{-1}) and O–D stretch (~2580 cm^{-1}). The O–D stretch peak appears at a lower frequency than the O–H peak due to the higher mass of deuterium as shown in eq. 1:

$$\tilde{v} = (1/2\pi c)\sqrt{\frac{f(m1+m2)}{m1 \cdot m2}} \quad \text{(eq. 1)}$$

($\tilde{v}$ = wavenumber; f = force constant; c = the speed of light; m_1, m_2 = mass of the two atoms)

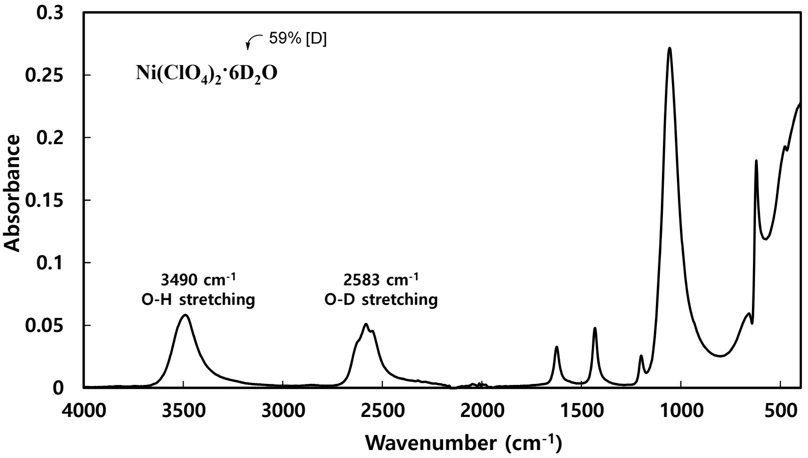

Supplementary Fig. 7. FT-IR spectrum of $Ni(ClO_4)_2 \cdot 6D_2O$.

In the FT-IR spectrum shown in Supplementary Fig. 7, the peaks at ~2580 cm^{-1} and at ~3490 cm^{-1} originate from the O–D stretch and the O–H stretch, respectively. When O–D and O–H stretch peaks coexist, they are not only from D_2O (for O–D) or H_2O (for O–H). Due to the

exposure of the sample to ambient air for the FT-IR measurement, the following equilibrium reaction (eq. 2) should be considered.

$$H_2O \rightleftharpoons HOD \rightleftharpoons D_2O \text{ (eq. 2)}$$

Therefore, the peak at ~2580 cm^{-1} originates from the O–D stretch of both D_2O and HOD. Likewise, the peak at ~3490 cm^{-1} arises from the O–H stretch of both H_2O and HOD. Based on the equilibrium shown in eq. 2, if the mole fraction of O–D ($x_{O\text{-}D} = n_{O\text{-}D}/(n_{O\text{-}D} + n_{O\text{-}H})$) is determined experimentally, the mole fractions of H_2O ($x_{H2O} = n_{H2O}/(n_{H2O} + n_{HOD} + n_{D2O})$), HOD ($x_{HOD} = n_{HOD}/(n_{H2O} + n_{HOD} + n_{D2O})$), and D_2O ($x_{D2O} = n_{D2O}/(n_{H2O} + n_{HOD} + n_{D2O})$) can also be determined. They are simply expressed as $(1 - x_{O\text{-}D})^2$, $2x_{O\text{-}D}(1 - x_{O\text{-}D})$, and $x_{O\text{-}D}^2$ for H_2O, HOD, and D_2O, respectively. $n_{O\text{-}D}$ and $n_{O\text{-}H}$ indicate the number of O–D and O–H groups in the sample, respectively, and n_{H2O}, n_{HOD}, and n_{D2O} represent the number of H_2O, HOD, and D_2O molecules in the sample, respectively. When the FT-IR spectrum is plotted as absorbance versus wavenumber, as shown in Supplementary Fig. 7, the integrated area of a particular peak is proportional to the product of the population and the integrated absorption coefficient (or oscillator strength) of the corresponding species (or functional group). Applying this relationship, $x_{O\text{-}D}$ in the measured sample can be estimated. To obtain $x_{O\text{-}D}$, the ratio between the integrated absorption coefficient of O–H and O–D is also required. Based on a previous study,[9] it was estimated to be ~1.76:1. In the sample, the ratio of the integrated area of the peak at ~3490 (for O–H) to that at ~2580 cm^{-1} (for O–D) is ~1.24:1. With these values, $x_{O\text{-}D}$ is estimated to be >0.59. Considering the dilution effect with atmospheric moisture over the course of measurement, the lower limit of the number percentage of D atoms in $Ni(ClO_4)_2{\cdot}6D_2O$ is 59%. The mole fractions of D_2O, HOD, and H_2O are estimated to be 0.35, 0.48, and 0.17, respectively. This set of mole fractions of three different water species (H_2O, HOD, and D_2O) indicates that the substitution reaction proceeded as expected.

V-2. Preparation of 3,3-dimethyl-1-butyne-1-*d* ([D]-2)

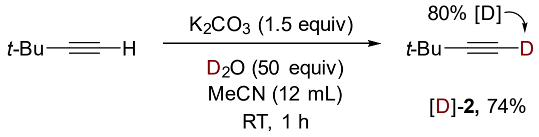

[D]-**2** was prepared according to the literature procedure with slight modifications:[10] To the solution of K_2CO_3 (5.0 g, 36.5 mmol, 1.5 equiv) in MeCN (12 mL), 3,3-dimethyl-1-butyne (2.0 g, 24.3 mmol, 1.0 equiv) and D_2O (24.3 g, 1.2 mol, 50 equiv) were added. The reaction mixture was stirred for 1 h at room temperature. After completion of the reaction, the mixture was distilled at 60 °C to afford the title compound [D]-**2** (a colorless liquid, 1.5 g, 74%); see also Supplementary Fig. 10.

V-3. ^{1}H NMR analysis for deuterated compounds

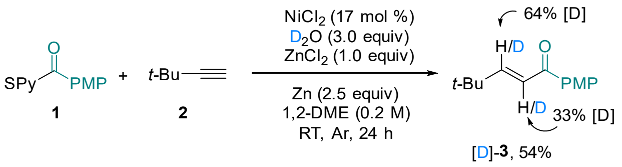

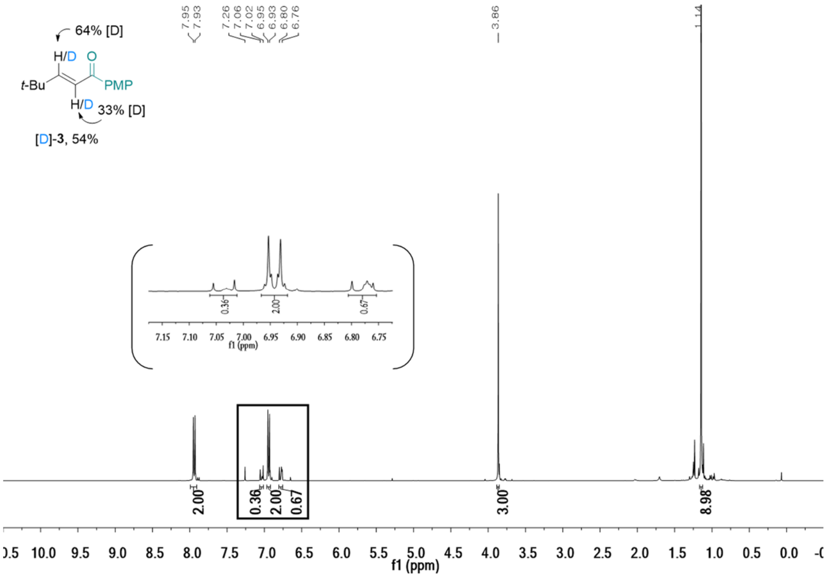

Supplementary Fig. 8. ^{1}H NMR (400 MHz, $CDCl_3$) of compound [D]-**3**.

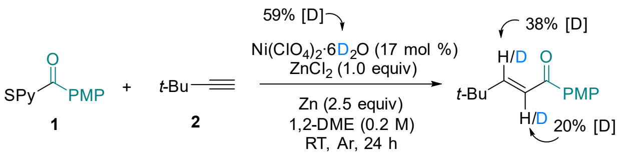

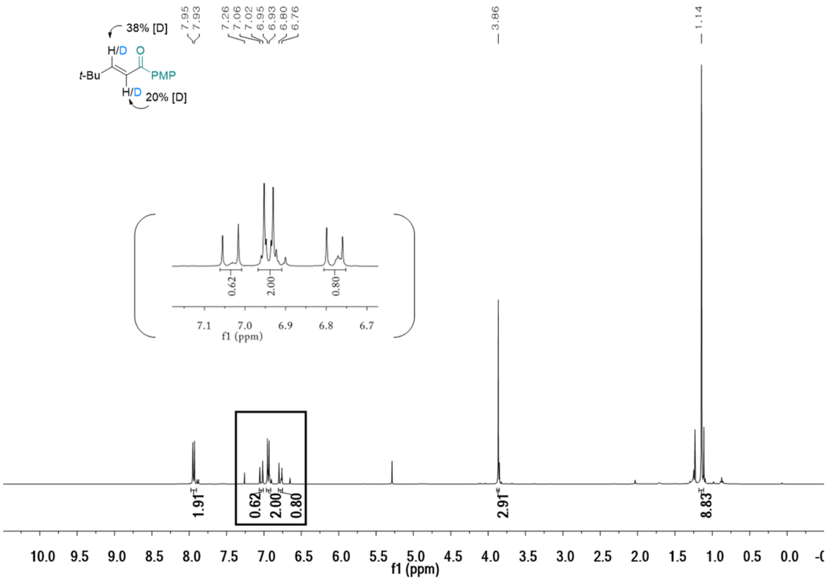

Supplementary Fig. 9. ^{1}H NMR (400 MHz, $CDCl_3$) of compound [D]-**3**.

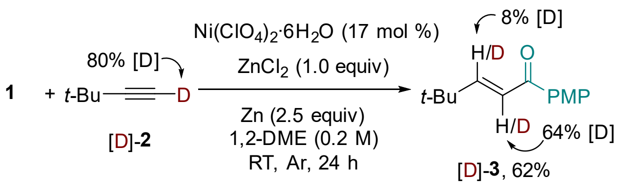

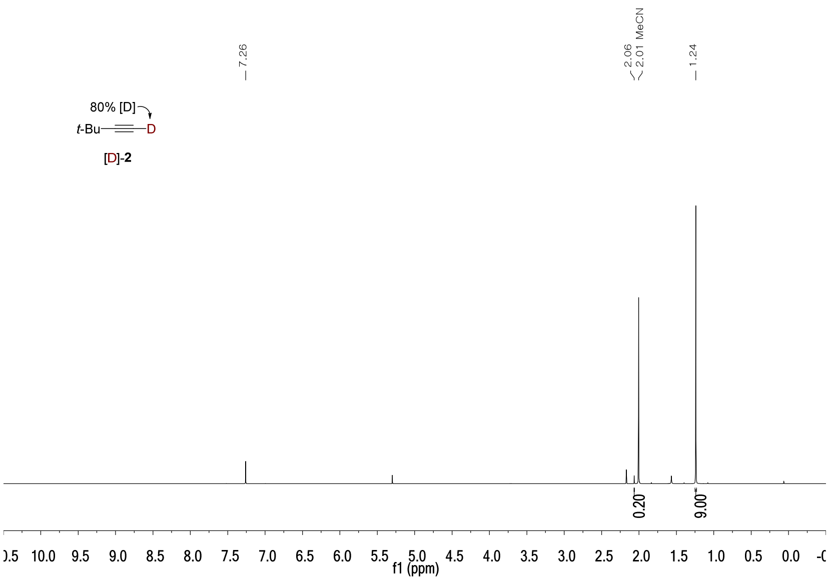

Supplementary Fig. 10. ^{1}H NMR (400 MHz, CDCl3) of compound [D]-**2**.

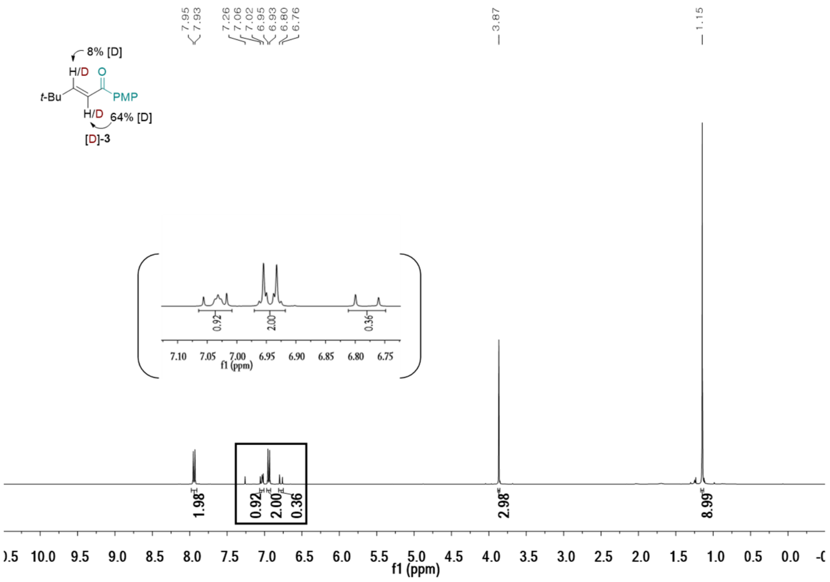

Supplementary Fig. 11. ^{1}H NMR (400 MHz, CDCl3) of compound [D]-**3**.

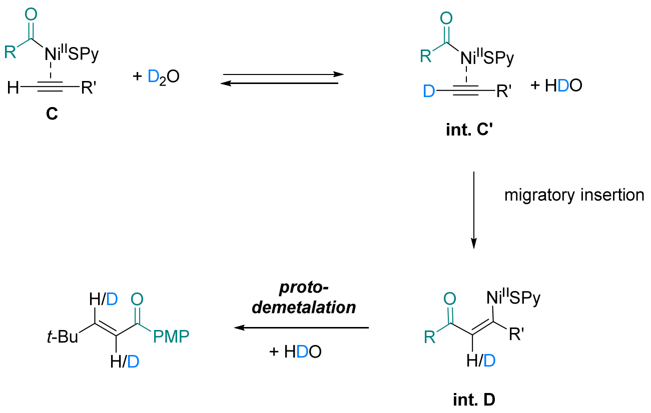

Supplementary Fig. 12. A schematic presentation of H/D scrambling.

VI. $ZnCl_2$ and ^{1}H NMR study

We have also compared the ^{1}H NMR spectra of thioester **1** before and after the addition of $ZnCl_2$. Supplementary Figs. 13 and 14 revealed the significant down-field shift of pyridyl CH moieties after the addition of $ZnCl_2$ indicating the deshielding effect of $ZnCl_2$-coordinated thioester **1**.

Experimental procedure: A 5-dram screw-cap vial equipped with a magnetic stir bar was charged with **1** (245 mg, 1.0 mmol, 1.0 equiv) and $ZnCl_2$ (135 mg, 1.0 mmol, 1.0 equiv) in a glove box. The mixture was dissolved in 1,2-DME (5 mL). The reaction mixture was stirred for 24 h at room temperature. After completion of the reaction, pentane (10 mL x 3 times) was added to the mixture. The precipitate was filtered and dried *in vacuo* to afford a white solid.

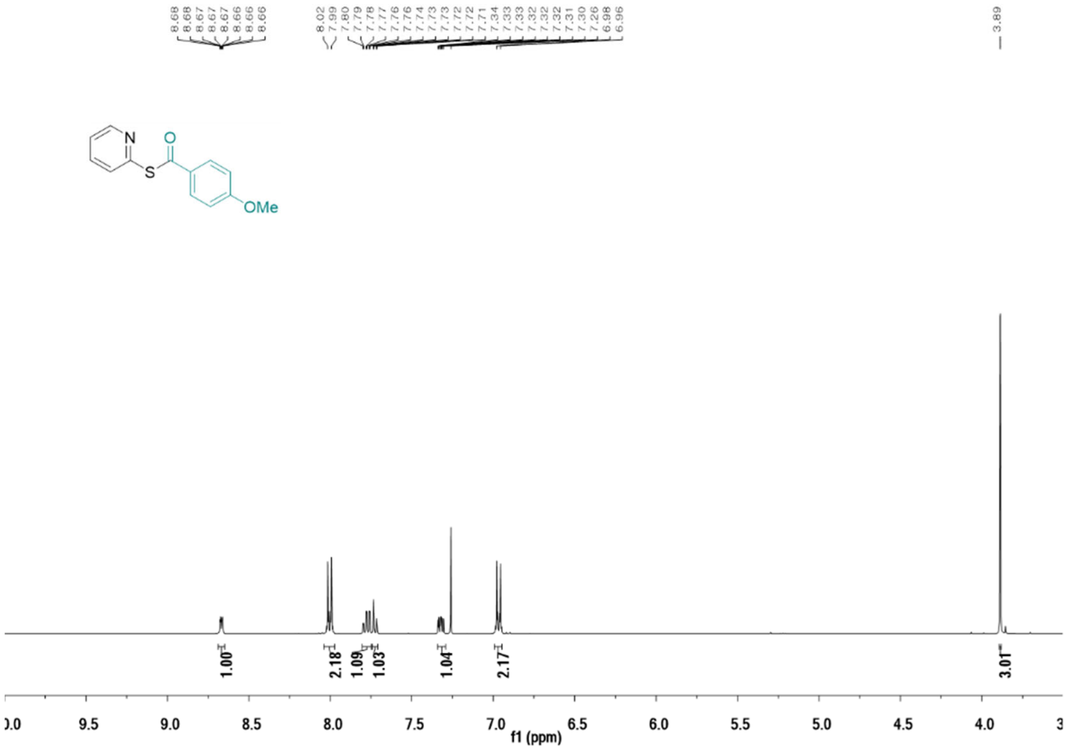

Supplementary Fig. 13. ^{1}H NMR (400 MHz, $CDCl_3$) of thioester **1** in the absence of $ZnCl_2$.

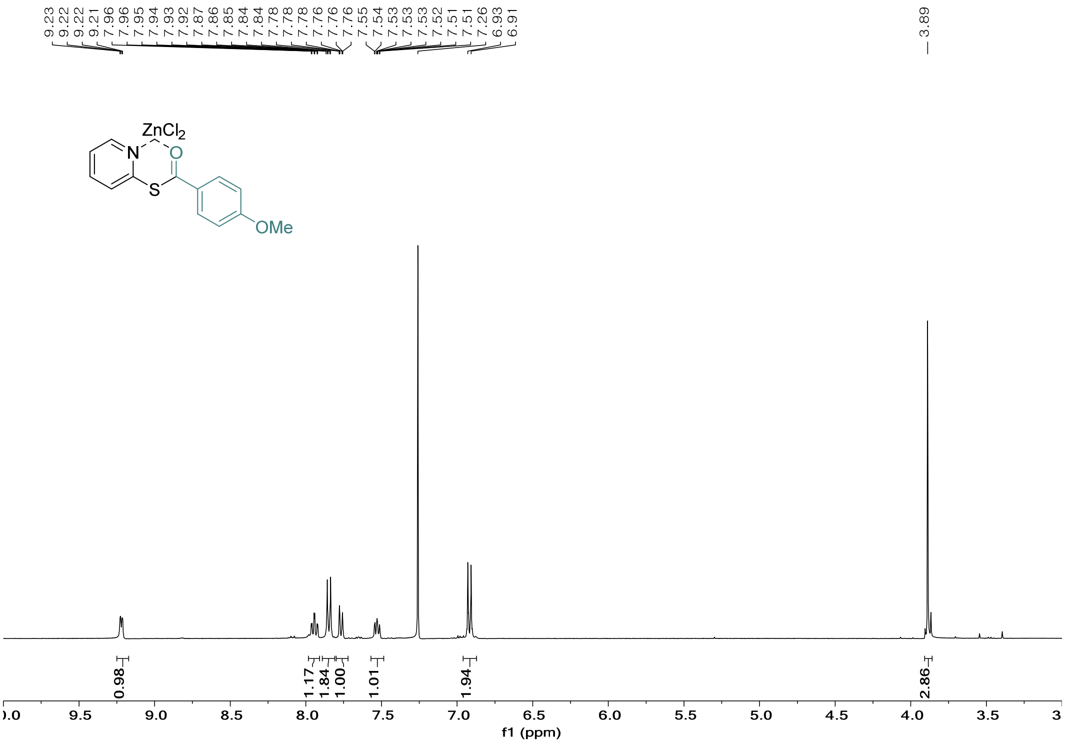

Supplementary Fig. 14. ^{1}H NMR (400 MHz, $CDCl_3$) of thioester **1** in the presence of $ZnCl_2$.

VII. X-ray single crystal diffraction data

X-ray diffraction data of single crystal of compound **27** were obtained at 100 K with monochromator (λ = 0.7 Å) synchrotron radiation source in Pohang Accelerator Laboratory (PAL) 2D beamline, Korea. X-ray diffraction data of single crystal of compound **66** were obtained at 173 K with Mo Kα radiation source using a Rigaku R-Axis Rapid II. Crystal structures of compound **27** and compound **66** were solved by the direct method and refined by full-matrix least-squares calculations using SHELXTL program package.[11] Thermal ellipsoids were shown at 50% probability. The data can be obtained free of charge from the Cambridge Crystallographic Data Centre: CCDC 2039192 (compound **27**) and 2036543 (compound **66**).

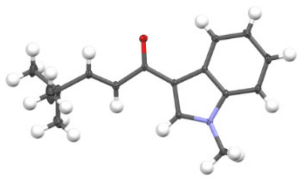

Supplementary Fig. 15. ORTEP diagram of compound **27**. Thermal ellipsoids are shown at 50% probability.

Supplementary Table 8. Crystallographic information of compound **27**

	Compound **27**	
Molecular formula	$C_{16}H_{19}NO$	
Temperature	100 K	
Crystal system	Monoclinic	
Space group	$P2_1/n$	
Unit cell dimensions	a = 10.497(2) Å	α = 90°
	b = 12.835(3) Å	β = 97.66(3)°
	c = 20.815(4) Å	γ = 90°
V (Å^3)	2779.4(10) Å^3	
Z	8	
ρ_{calc} (g·cm^{-3})	1.153	
μ (mm^{-1})	0.068	
R_1, $I > 2\sigma(I)$	0.0736	
wR_2, $I > 2\sigma(I)$	0.2178	

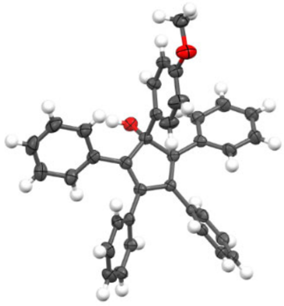

Supplementary Fig. 16. ORTEP diagram of compound **66**. Thermal ellipsoids are shown at 50% probability.

Supplementary Table 9. Crystallographic information of compound **66**

	Compound **66**	
Molecular formula	$C_{36}H_{28}O_2$	
Temperature	173 K	
Crystal system	Triclinic	
Space group	*P*-1	
Unit cell dimensions	a = 10.180(2) Å	α = 101.27(3)°
	b = 10.370(2) Å	β = 91.58(3)°
	c = 14.190(3) Å	γ = 115.56(3)°
V (Å^3)	1314.7(6) Å^3	
Z	2	
ρ_{calc} (g·cm^{-3})	1.244	
μ (mm^{-1})	0.076	
R_1, $I > 2\sigma(I)$	0.0823	
wR_2, $I > 2\sigma(I)$	0.1697	

VIII. Characterization data

VIII-1. Enone products

(*E*)-1-(4-Methoxyphenyl)-4,4-dimethylpent-2-en-1-one (3)[12]: method **GP-III**; a colorless oil

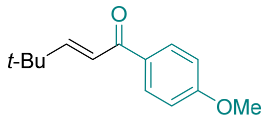

(35 mg, 81%); **^{1}H NMR** (400 MHz, $CDCl_3$) δ 7.95 (d, *J* = 8.9 Hz, 2H), 7.04 (d, *J* = 15.6 Hz, 1H), 6.95 (d, *J* = 8.9 Hz, 2H), 6.78 (d, *J* = 15.6 Hz, 1H), 3.87 (s, 3H), 1.15 (s, 9H); **^{13}C NMR** (100 MHz, $CDCl_3$) δ 190.0, 163.4, 158.8, 131.2, 130.9, 120.7, 113.8, 55.6, 34.2, 29.0.

(*E*)-4,4-Dimethyl-1-phenylpent-2-en-1-one (4)[12]: method **GP-III**; a colorless oil (17 mg,

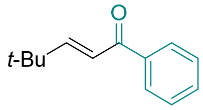

45%); **^{1}H NMR** (400 MHz, $CDCl_3$) δ 7.92 (d, *J* = 7.0 Hz, 2H), 7.55 (t, *J* = 7.4 Hz, 1H), 7.47 (t, *J* = 7.4 Hz, 2H), 7.06 (d, *J* = 15.7 Hz, 1H), 6.78 (d, *J* = 15.6 Hz, 1H), 1.16 (s, 9H); **^{13}C NMR** (100 MHz, $CDCl_3$) δ 191.8, 159.8, 138.4, 132.7, 128.7, 128.6, 121.1, 34.3, 28.9.

(*E*)-4,4-Dimethyl-1-(*p*-tolyl)pent-2-en-1-one (5): method **GP-III**; a yellow oil (28 mg, 70%);

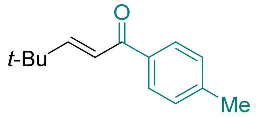

^{1}H NMR (400 MHz, $CDCl_3$) δ 7.84 (d, *J* = 7.9 Hz, 2H), 7.26 (d, *J* = 7.9 Hz, 2H), 7.05 (d, *J* = 15.6 Hz, 1H), 6.78 (d, *J* = 15.6 Hz, 1H), 2.41 (s, 3H), 1.15 (s, 9H); **^{13}C NMR** (100 MHz, $CDCl_3$) δ 191.2, 159.2, 143.4, 135.8, 129.3, 128.8, 121.0, 34.3, 28.9, 21.8; **HRMS (ESI):** m/z $[M+H]^+$ calcd for ($C_{14}H_{19}O^+$): 203.14304, found: 203.14302.

(*E*)-1-(4-Ethylphenyl)-4,4-dimethylpent-2-en-1-one (6): method **GP-III**; a yellow oil (27 mg,

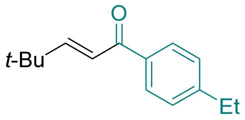

62%); **^{1}H NMR** (400 MHz, $CDCl_3$) δ 7.86 (d, *J* = 8.3 Hz, 2H), 7.29 (d, *J* = 8.3 Hz, 2H), 7.05 (d, *J* = 15.7 Hz, 1H), 6.78 (d, *J* = 15.7 Hz, 1H), 2.71 (q, *J* = 7.7 Hz, 2H), 1.26 (t, *J* = 7.6 Hz, 3H), 1.15 (s, 9H); **^{13}C NMR** (100 MHz, $CDCl_3$) δ 191.3, 159.3, 149.7, 136.0, 128.9, 128.1, 121.1, 34.3, 29.1, 28.9, 15.4; **HRMS (ESI):** m/z $[M+H]^+$ calcd for ($C_{15}H_{21}O^+$): 217.15869, found: 217.15868.

(*E*)-1-(4-Isopropylphenyl)-4,4-dimethylpent-2-en-1-one (7): method **GP-III**; a yellow oil

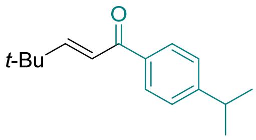

(29 mg, 63%); **^{1}H NMR** (400 MHz, $CDCl_3$) δ 7.87 (d, *J* = 8.3 Hz, 2H), 7.32 (d, *J* = 8.2 Hz, 2H), 7.04 (d, *J* = 15.7 Hz, 1H), 6.77 (d, *J* = 15.7 Hz, 1H), 2.97 (m,1H), 1.28 (d, *J* = 6.9 Hz, 6H), 1.15 (s, 9H); **^{13}C NMR** (100

MHz, $CDCl_3$) δ 191.3, 159.2, 154.2, 136.2, 128.9, 126.7, 121.1, 34.4, 34.3, 28.9, 23.9; **HRMS (ESI):** m/z $[M+H]^+$ calcd for ($C_{16}H_{23}O^+$): 231.17434, found: 231.17424.

(*E*)-1-(4-(*Tert*-butyl)phenyl)-4,4-dimethylpent-2-en-1-one (8): method **GP-III**; an orange oil (30 mg, 62%); **^{1}H NMR** (400 MHz, $CDCl_3$) δ 7.88 (d, *J* = 8.5 Hz, 2H), 7.48 (d, *J* = 8.5 Hz, 2H), 7.05 (d, *J* = 15.7 Hz, 1H), 6.78 (d, *J* = 15.7 Hz, 1H), 1.35 (s, 9H), 1.15 (s, 9H); **^{13}C NMR** (100 MHz, $CDCl_3$) δ 191.3, 159.2, 156.4, 135.7, 128.6, 125.6, 121.1, 35.2, 34.3, 31.3, 28.9; **HRMS (ESI):** m/z $[M+H]^+$ calcd for ($C_{17}H_{25}O^+$): 245.18999, found: 245.18990.

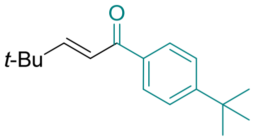

(*E*)-1-([1,1'-Biphenyl]-4-yl)-4,4-dimethylpent-2-en-1-one (9): method **GP-IV**; a white solid (21 mg, 40%); **m.p.** 127 °C; **^{1}H NMR** (400 MHz, $CDCl_3$) δ 8.01 (d, *J* = 8.4 Hz, 2H), 7.70 (d, *J* = 8.4 Hz, 2H), 7.64 (d, *J* = 7.0 Hz, 2H), 7.48 (t, *J* = 7.4 Hz, 2H), 7.40 (t, *J* = 7.3 Hz, 1H), 7.10 (d, *J* = 15.7 Hz, 1H), 6.83 (d, *J* = 15.7 Hz, 1H), 1.18 (s, 9H); **^{13}C NMR** (100 MHz, $CDCl_3$) δ 191.2, 159.7, 145.5, 140.2, 137.1, 129.3, 129.1, 128.3, 127.4, 127.3, 121.1, 34.4, 28.9; **HRMS (ESI):** m/z $[M+H]^+$ calcd for ($C_{19}H_{21}O^+$): 265.15869, found: 265.15851.

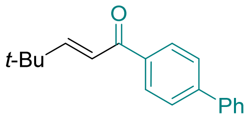

(*E*)-1-(4-Ethoxyphenyl)-4,4-dimethylpent-2-en-1-one (10): method **GP-III**; a colorless oil (37 mg, 79%); **^{1}H NMR** (400 MHz, $CDCl_3$) δ 7.93 (d, *J* = 8.9 Hz, 2H), 7.03 (d, *J* = 15.6 Hz, 1H), 6.93 (d, *J* = 8.9 Hz, 2H), 6.78 (d, *J* = 15.6 Hz, 1H), 4.09 (q, *J* = 7.0 Hz, 2H), 1.44 (t, *J* = 7.0 Hz, 3H), 1.14 (s, 9H); **^{13}C NMR** (100 MHz, $CDCl_3$) δ 189.9, 162.8, 158.6, 131.0, 130.9, 120.7, 114.3, 63.8, 34.2, 28.9, 14.8; **HRMS (ESI):** m/z $[M+H]^+$ calcd for ($C_{15}H_{21}O_2^+$): 233.15361, found: 233.15346.

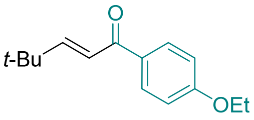

(*E*)-1-(4-(Benzyloxy)phenyl)-4,4-dimethylpent-2-en-1-one (11): method **GP-III**; a white solid (38 mg, 64%); **m.p.** 107 °C; **^{1}H NMR** (400 MHz, $CDCl_3$) δ 7.95 (d, *J* = 8.9 Hz, 2H), 7.46 – 7.32 (m, 5H), 7.04 (d, *J* = 15.6 Hz, 1H), 7.03 (d, *J* = 8.9 Hz, 2H), 6.78 (d, *J* = 15.6 Hz, 1H), 5.14 (s, 2H), 1.15 (s, 9H); **^{13}C NMR** (100 MHz, $CDCl_3$) δ 190.0, 162.5, 158.8, 136.4, 131.4, 131.0, 128.8, 128.4, 127.6, 120.7, 114.7, 70.3, 34.3, 29.0; **HRMS (ESI):** m/z $[M+H]^+$ calcd for ($C_{20}H_{23}O_2^+$): 295.16926, found: 295.16910.

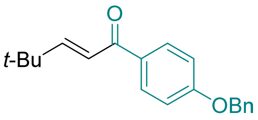

(*E*)-4,4-Dimethyl-1-(4-phenoxyphenyl)pent-2-en-1-one (12): method **GP-IV**; an orange solid (32 mg, 57%); **m.p.** 117 °C; **^{1}H NMR** (400 MHz, $CDCl_3$) δ 7.94 (d, *J* = 8.8 Hz, 2H), 7.43 – 7.36 (m, 2H), 7.19 (t, *J* = 7.4 Hz, 1H), 7.09 – 7.00 (m, 4H), 7.05 (d, *J* = 15.6 Hz, 1H), 6.77 (d, *J* = 15.6 Hz, 1H), 1.15 (s, 9H); **^{13}C NMR** (100 MHz, $CDCl_3$) δ 190.1, 161.8, 159.3, 155.8, 132.9, 130.9, 130.2, 124.7, 120.8, 120.2, 117.5, 34.3, 28.9; **HRMS (ESI):** m/z $[M+H]^+$ calcd for ($C_{19}H_{21}O_2^+$): 281.15361, found: 281.15341.

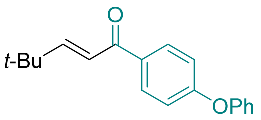

(*E*)-1-(4-Hydroxyphenyl)-4,4-dimethylpent-2-en-1-one (13): method **GP-III**; an orange oil (20 mg, 48%); **^{1}H NMR** (400 MHz, $CDCl_3$) δ 7.90 (d, *J* = 8.7 Hz, 2H), 7.09 (br, 1H), 7.06 (d, *J* = 15.6 Hz, 1H), 6.93 (d, *J* = 8.8 Hz, 2H), 6.79 (d, *J* = 15.6 Hz, 1H), 1.14 (s, 9H); **^{13}C NMR** (100 MHz, $CDCl_3$) δ 190.9, 160.8, 159.5, 131.4, 130.7, 120.7, 115.6, 34.3, 28.9; **HRMS (ESI):** m/z $[M+H]^+$ calcd for ($C_{13}H_{17}O_2^+$): 205.12231, found: 205.12222.

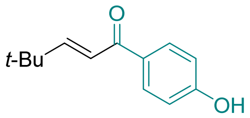

(*E*)-1-(4-Fluorophenyl)-4,4-dimethylpent-2-en-1-one (14)[12]: method **GP-III**; a white solid (10 mg, 25%); **^{1}H NMR** (400 MHz, $CDCl_3$) δ 7.96 (dd, *J* = 8.8, 5.5 Hz, 2H), 7.14 (t, *J* = 8.6 Hz, 2H), 7.06 (d, *J* = 15.7 Hz, 1H), 6.75 (d, *J* = 15.6 Hz, 1H), 1.16 (s, 9H); **^{13}C NMR** (100 MHz, $CDCl_3$) δ 189.9, 165.6 (d, *J* = 253.9 Hz), 159.9, 134.6 (d, *J* = 2.9 Hz), 131.2 (d, *J* = 9.2 Hz), 120.6, 115.7 (d, *J* = 21.8 Hz), 34.32, 28.85.

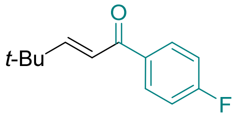

(*E*)-1-(4-Chlorophenyl)-4,4-dimethylpent-2-en-1-one (15)[12]: method **GP-IV**; a white solid (19 mg, 43%); **^{1}H NMR** (400 MHz, $CDCl_3$) δ 7.87 (d, *J* = 8.6 Hz, 2H), 7.44 (d, *J* = 8.6 Hz, 2H), 7.07 (d, *J* = 15.6 Hz, 1H), 6.73 (d, *J* = 15.6 Hz, 1H), 1.15 (s, 9H); **^{13}C NMR** (100 MHz, $CDCl_3$) δ 190.4, 160.4, 139.1, 136.6, 130.1, 128.9, 120.6, 34.4, 28.9.

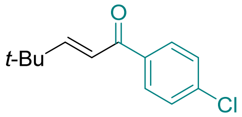

(*E*)-1-(4-Bromophenyl)-4,4-dimethylpent-2-en-1-one (16)[12]: method **GP-III**; a colorless oil (15 mg, 28%); **^{1}H NMR** (400 MHz, $CDCl_3$) δ 7.79 (d, *J* = 8.6 Hz, 2H), 7.61 (d, *J* = 8.6 Hz, 2H), 7.06 (d, *J* = 15.7 Hz, 1H), 6.72 (d, *J* = 15.7 Hz, 1H), 1.15 (s, 9H); **^{13}C NMR** (100 MHz, $CDCl_3$) δ 190.6, 160.5, 137.1, 131.9, 130.2, 127.8, 120.6, 34.4, 28.9.

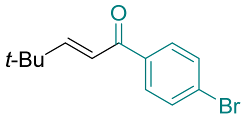

Methyl *(E)*-4-(4,4-dimethylpent-2-enoyl)benzoate (17): method **GP-IV**; a white solid (10 mg, 21%); **m.p.** 95 °C; **^{1}H NMR** (400 MHz, $CDCl_3$) δ 8.13 (d, *J* = 8.6 Hz, 2H), 7.95 (d, *J* = 8.6 Hz, 2H), 7.07 (d, *J* = 15.7 Hz, 1H), 6.75 (d, *J* = 15.7 Hz, 1H), 3.95 (s, 3H), 1.16 (s, 9H); **^{13}C NMR** (100 MHz, $CDCl_3$) δ 191.3, 166.5, 161.0, 141.9, 129.9, 129.7, 128.5, 121.1, 52.6, 34.5, 28.8; **HRMS (ESI):** m/z $[M+H]^+$ calcd for ($C_{15}H_{19}O_3^+$): 247.13287, found: 247.13271.

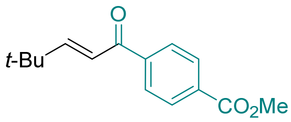

(*E*)-4,4-Dimethyl-1-(4-(trifluoromethyl)phenyl)pent-2-en-1-one (18)[13]: method **GP-IV**; a white solid (11 mg, 20%); **^{1}H NMR** (400 MHz, $CDCl_3$) δ 8.00 (d, *J* = 8.1 Hz, 2H), 7.73 (d, *J* = 8.2 Hz, 2H), 7.08 (d, *J* = 15.7 Hz, 1H), 6.74 (d, *J* = 15.8 Hz, 1H), 1.16 (s, 9H); **^{13}C NMR** (101 MHz, $CDCl_3$) δ 190.9, 161.3, 141.2, 133.9 (q, *J* = 32.6 Hz), 128.9, 125.7 (q, *J* = 3.8 Hz), 123.8 (q, *J* = 272.8 Hz), 120.9, 34.5, 28.8.

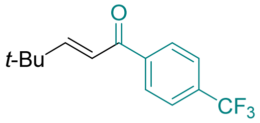

(*E*)-4,4-Dimethyl-1-(*m*-tolyl)pent-2-en-1-one (19): method **GP-III**; a yellow oil (22 mg, 54%); **^{1}H NMR** (400 MHz, $CDCl_3$) δ 7.72 (m, 2H), 7.36 (m, 2H), 7.05 (d, *J* = 15.7 Hz, 1H), 6.77 (d, *J* = 15.7 Hz, 1H), 2.42 (s, 3H), 1.16 (s, 9H); **^{13}C NMR** (100 MHz, $CDCl_3$) δ 191.9, 159.6, 138.4, 138.4, 133.5, 129.2, 128.5, 125.9, 121.2, 34.3, 28.9, 21.5; **HRMS (ESI):** m/z $[M+H]^+$ calcd for ($C_{14}H_{19}O^+$): 203.14304, found: 203.14308.

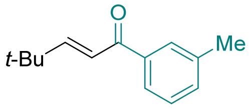

(*E*)-1-(3-Methoxyphenyl)-4,4-dimethylpent-2-en-1-one (20): method **GP-IV**; a colorless oil (18 mg, 42%); **^{1}H NMR** (400 MHz, $CDCl_3$) δ 7.49 (d, *J* = 7.7 Hz, 1H), 7.45 (s, 1H), 7.37 (t, *J* = 7.9 Hz, 1H), 7.10 (ddd, *J* = 8.2, 2.7, 0.9 Hz, 1H), 7.05 (d, *J* = 15.7 Hz, 1H), 6.75 (d, *J* = 15.7 Hz, 1H), 3.86 (s, 3H), 1.15 (s, 9H); **^{13}C NMR** (100 MHz, $CDCl_3$) δ 191.5, 159.9, 159.8, 139.8, 129.6, 121.2, 121.1, 119.1, 113.1, 55.6, 34.3, 28.9; **HRMS (ESI)**: m/z $[M+H]^+$ calcd for ($C_{14}H_{19}O_2^+$): 219.13796, found: 219.13788.

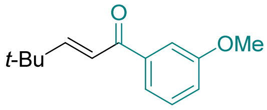

(*E*)-1-(3-Ethoxyphenyl)-4,4-dimethylpent-2-en-1-one (21): method **GP-IV**; a colorless oil (21 mg, 45%); **^{1}H NMR** (400 MHz, $CDCl_3$) δ 7.48 (d, *J* = 7.6 Hz, 1H), 7.44 (s, 1H), 7.36 (t, *J* = 7.9 Hz, 1H), 7.11 – 7.07 (m, 1H), 7.05 (d, *J* =

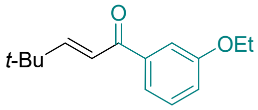

15.7 Hz, 1H), 6.75 (d, J = 15.7 Hz, 1H), 4.09 (q, J = 6.9 Hz, 2H), 1.43 (t, J = 7.0 Hz, 3H), 1.15 (s, 9H); **^{13}C NMR** (100 MHz, $CDCl_3$) δ 191.5, 159.7, 159.2, 139.7, 129.6, 121.1, 121.1, 119.5, 113.8, 63.8, 34.3, 28.9, 14.9; **HRMS (ESI):** m/z $[M+H]^+$ calcd for ($C_{15}H_{21}O_2^+$): 233.15361, found: 233.15364.

(*E*)-1-(2-Methoxyphenyl)-4,4-dimethylpent-2-en-1-one (22): method **GP-IV**; a colorless oil (26 mg, 58%); **^{1}H NMR** (400 MHz, $CDCl_3$) δ 7.50 (dd, J = 7.6, 1.8 Hz, 1H), 7.47 – 7.40 (m, 1H), 7.00 (t, J = 7.5 Hz, 1H), 6.96 (d, J = 8.3 Hz, 1H), 6.83 (d, J = 15.8 Hz, 1H), 6.58 (d, J = 15.8 Hz, 1H), 3.86 (s, 3H), 1.11 (s, 9H); **^{13}C NMR** (100 MHz, $CDCl_3$) δ 194.3, 158.4, 157.8, 132.5, 130.2, 129.4, 125.9, 120.6, 111.6, 55.6, 33.9, 28.8; **HRMS (ESI):** m/z $[M+H]^+$ calcd for ($C_{14}H_{19}O_2^+$): 219.13796, found: 219.13792.

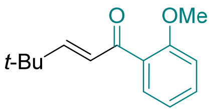

(*E*)-1-(2-Ethoxyphenyl)-4,4-dimethylpent-2-en-1-one (23): method **GP-IV**; a yellow oil (29 mg, 62%); **^{1}H NMR** (400 MHz, $CDCl_3$) δ 7.56 (dd, J = 7.6, 1.8 Hz, 1H), 7.43 – 7.37 (m, 1H), 6.98 (t, J = 7.5 Hz, 1H), 6.92 (d, J = 8.3 Hz, 1H), 6.88 (d, J = 15.8 Hz, 1H), 6.73 (d, J = 15.8 Hz, 1H), 4.08 (q, J = 7.0 Hz, 2H), 1.42 (t, J = 7.0 Hz, 3H), 1.11 (s, 9H); **^{13}C NMR** (100 MHz, $CDCl_3$) δ 194.0, 157.6, 157.4, 132.8, 130.5, 129.6, 126.1, 120.7, 112.5, 64.2, 34.0, 28.9, 15.0; **HRMS (ESI):** m/z $[M+H]^+$ calcd for ($C_{15}H_{21}O_2^+$): 233.15361, found: 233.15349.

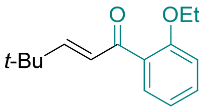

(*E*)-4,4-Dimethyl-1-(*o*-tolyl)pent-2-en-1-one (24): method **GP-III**; a yellow oil (26 mg, 64%); **^{1}H NMR** (400 MHz, $CDCl_3$) δ 7.38 (d, J = 7.5 Hz, 1H), 7.34 (d, J = 7.4 Hz, 1H), 7.25 – 7.21 (m, 2H), 6.71 (d, J = 16.0 Hz, 1H), 6.40 (d, J = 16.0 Hz, 1H), 2.39 (s, 3H), 1.11 (s, 9H); **^{13}C NMR** (100 MHz, $CDCl_3$) δ 197.8, 161.1, 139.4, 136.8, 131.3, 130.3, 128.2, 126.0, 125.5, 34.2, 28.8, 20.3; **HRMS (ESI):** m/z $[M+H]^+$ calcd for ($C_{14}H_{19}O^+$): 203.14304, found: 203.14302.

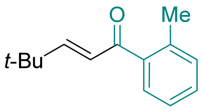

(*E*)-4,4-Dimethyl-1-(naphthalen-2-yl)pent-2-en-1-one (25)[12]: method **GP-III**; an orange oil (21 mg, 44%); **^{1}H NMR** (400 MHz, $CDCl_3$) δ 8.44 (s, 1H), 8.02 (d, J = 8.6 Hz, 1H), 7.98 (d, J = 8.3 Hz, 1H), δ 7.91 (d, J = 9.0 Hz, 1H), δ 7.89 (d, J = 8.0 Hz, 1H), 7.62 – 7.53 (m, 2H), 7.14 (d, J = 15.7 Hz, 1H), 6.94

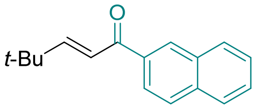

(d, J = 15.7 Hz, 1H), 1.20 (s, 9H); **^{13}C NMR** (100 MHz, $CDCl_3$) δ 191.4, 159.7, 135.7, 135.5, 132.6, 130.0, 129.6, 128.5, 128.4, 127.9, 126.8, 124.7, 121.0, 34.4, 28.9.

(*E*)-4,4-Dimethyl-1-(naphthalen-1-yl)pent-2-en-1-one (26)[13]: method **GP-III**; a white solid

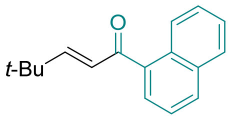

(15 mg, 32%); **^{1}H NMR** (400 MHz, $CDCl_3$) δ 8.26 (d, J = 7.4 Hz, 1H), 7.97 (d, J = 8.3 Hz, 1H), 7.89 (d, J = 7.0 Hz, 1H), 7.66 (d, J = 7.1 Hz, 1H), 7.56 – 7.50 (m, 3H), 6.86 (d, J = 16.0 Hz, 1H), 6.58 (d, J = 16.0 Hz, 1H), 1.12 (s, 9H); **^{13}C NMR** (100 MHz, $CDCl_3$) δ 196.9, 161.3, 137.2, 133.9, 131.5, 130.6, 128.5, 127.4, 127.2, 126.5, 126.3, 125.8, 124.5, 34.3, 28.8.

(*E*)-4,4-Dimethyl-1-(1-methyl-1*H*-indol-3-yl)pent-2-en-1-one (27): method **GP-IV**; a

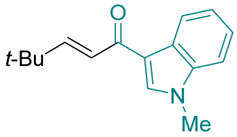

yellow solid (39 mg, 82%); **m.p.** 135 °C; **^{1}H NMR** (400 MHz, $CDCl_3$) δ 8.48 – 8.43 (m, 1H), 7.76 (s, 1H), 7.36 – 7.30 (m, 3H), 7.05 (d, J = 15.5 Hz, 1H), 6.65 (d, J = 15.5 Hz, 1H), 3.86 (s, 3H), 1.17 (s, 9H); **^{13}C NMR** (100 MHz, $CDCl_3$) δ 185.6, 155.5, 137.7, 135.4, 127.0, 123.6, 123.1, 122.7, 122.3, 117.5, 109.7, 33.9, 33.7, 29.1; **HRMS (ESI):** m/z $[M+H]^+$ calcd for ($C_{16}H_{20}NO^+$): 242.15394, found: 242.15375.

(*E*)-2,2,6,6-Tetramethylhept-4-en-3-one (28)[13]: method **GP-III**; a white solid (12 mg, 39%);

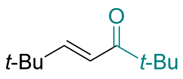

^{1}H NMR (400 MHz, $CDCl_3$) δ 6.93 (d, J = 15.4 Hz, 1H), 6.39 (d, J = 15.5 Hz, 1H), 1.15 (s, 9H), 1.08 (s, 9H); **^{13}C NMR** (100 MHz, $CDCl_3$) δ 205.0, 157.4, 119.1, 43.2, 33.9, 28.9, 26.4.

(*E*)-2,2-Dimethylnon-3-en-5-one (29)[14]: method **GP-III**; a yellow oil (8 mg, 24%); **^{1}H NMR**

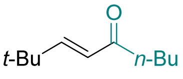

(400 MHz, $CDCl_3$) δ 6.81 (d, J = 16.1 Hz, 1H), 6.01 (d, J = 16.1 Hz, 1H), δ 2.54 (t, J = 7.7 Hz, 2H), 1.61 – 1.56 (m, 2H), 1.37 – 1.32 (m, 2H), 1.08 (s, 9H), 0.92 (t, J = 7.3 Hz, 3H); **^{13}C NMR** (100 MHz, $CDCl_3$) δ 201.5, 156.8, 125.5, 40.1, 33.7, 28.8, 26.5, 22.5, 14.0.

(*E*)-1-Cyclopropyl-4,4-dimethylpent-2-en-1-one (30): method **GP-III**; a yellow oil (17 mg,

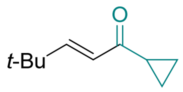

56%); **^{1}H NMR** (400 MHz, $CDCl_3$) δ 6.89 (d, J = 16.1 Hz, 1H), 6.15 (d, J = 16.1 Hz, 1H), 2.15 (tt, J = 7.9, 4.6 Hz, 1H), 1.11 (s, 9H), 1.08 (dt, J = 4.3, 3.1 Hz, 2H), 0.91 (dt, J = 8.1, 3.4 Hz, 2H); **^{13}C NMR** (100 MHz, $CDCl_3$) δ 201.1, 156.7, 125.8,

33.9, 28.9, 19.0, 11.3; **HRMS (ESI):** m/z $[M+H]^+$ calcd for ($C_{10}H_{17}O^+$): 153.12739, found: 153.12737.

(*E*)-1-Cyclopentyl-4,4-dimethylpent-2-en-1-one (31): method **GP-III**; an orange oil (14 mg, 40%); **^{1}H NMR** (400 MHz, $CDCl_3$) δ 6.84 (d, *J* = 16.0 Hz, 1H), 6.05 (d, *J* = 16.0 Hz, 1H), 3.10 (m, 1H), 1.85 – 1.71 (m, 4H), 1.64 – 1.54 (m, 4H), 1.08 (d, *J* = 1.3 Hz, 9H); **^{13}C NMR** (100 MHz, $CDCl_3$) δ 203.5, 157.0, 124.7, 49.2, 33.9, 29.5, 28.9, 26.3; **HRMS (ESI):** m/z $[M+H]^+$ calcd for ($C_{12}H_{21}O^+$): 181.15869, found: 181.15865.

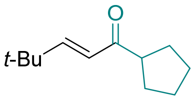

(*E*)-1-Cyclohexyl-4,4-dimethylpent-2-en-1-one (32)[15]: method **GP-IV**; a yellow oil (28 mg, 73%); **^{1}H NMR** (400 MHz, $CDCl_3$) δ 6.84 (d, *J* = 16.0 Hz, 1H), 6.06 (d, *J* = 16.0 Hz, 1H), 2.56 (m, 1H), 1.71 – 1.62 (m, 2H), 1.42 – 1.16 (m, 8H), 1.08 (d, *J* = 1.3 Hz, 9H); **^{13}C NMR** (100 MHz, $CDCl_3$) δ 204.2, 156.9, 123.7, 49.0, 33.9, 28.9, 28.9, 26.1, 25.9.

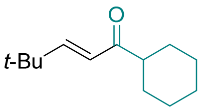

(*E*)-1-(4-Methoxyphenyl)-2-buten-1-one (33)[16]: method **GP-IV**; a colorless oil (24 mg, 68%); **^{1}H NMR** (400 MHz, $CDCl_3$) δ 7.94 (d, *J* = 8.9 Hz, 2H), 7.05 (dq, *J* = 15.3, 6.8 Hz, 1H), 6.96 – 6.89 (m, 3H), 3.87 (s, 3H), 1.99 (dd, *J* = 6.7, 1.5 Hz, 3H); **^{13}C NMR** (100 MHz, $CDCl_3$) δ 189.1, 163.4, 144.1, 130.9, 130.9, 127.3, 113.9, 55.6, 18.7.

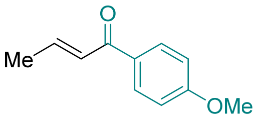

(*E*)-1-(4-Methoxyphenyl)-4-methylpent-2-en-1-one (34)[17]: method **GP-IV**; a yellow oil (20 mg, 48%); **^{1}H NMR** (400 MHz, $CDCl_3$) δ 7.94 (d, *J* = 8.8 Hz, 2H), 7.01 (dd, *J* = 15.4, 6.7 Hz, 1H), 6.94 (d, *J* = 8.8 Hz, 2H), 6.83 (dd, *J* = 15.4, 1.3 Hz, 1H), 3.87 (s, 3H), 2.56 (m, 1H), 1.13 (d, *J* = 6.8 Hz, 6H); **^{13}C NMR** (100 MHz, $CDCl_3$) δ 189.7, 163.4, 155.1, 131.1, 130.9, 122.8, 113.8, 55.6, 31.6, 21.6.

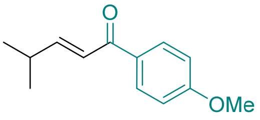

(*E*)-3-Cyclopropyl-1-(4-methoxyphenyl)prop-2-en-1-one (35): method **GP-IV**; a yellow oil (10 mg, 25%); **^{1}H NMR** (400 MHz, $CDCl_3$) δ 7.96 (d, *J* = 8.4 Hz, 2H), 7.03 (d, *J* = 15.0 Hz, 1H), 6.94 (d, *J* = 8.4 Hz, 2H), 6.54 (dd, *J* = 15.0, 10.2 Hz, 1H), 3.87 (s, 3H), 1.73 – 1.65 (m, 1H), 1.04 – 0.97 (m, 2H), 0.72 (m, 2H); **^{13}C NMR** (100 MHz, $CDCl_3$) δ 188.3, 163.3, 154.3, 131.1, 130.8, 122.6, 113.8,

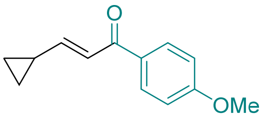

55.6, 15.4, 9.2; **HRMS (ESI):** m/z $[M+H]^+$ calcd for ($C_{13}H_{15}O_2^+$): 203.10666, found: 203.10660.

(*E*)-3-Cyclopentyl-1-(4-methoxyphenyl)prop-2-en-1-one (36): method **GP-IV**; a brown oil

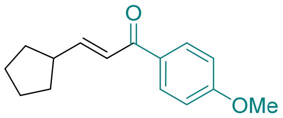

(15 mg, 33%); **^{1}H NMR** (400 MHz, $CDCl_3$) δ 7.94 (d, *J* = 8.9 Hz, 2H), 7.03 (dd, *J* = 15.3, 8.0 Hz, 1H), 6.94 (d, *J* = 8.9 Hz, 2H), 6.86 (dd, *J* = 15.3, 0.9 Hz, 1H), 3.87 (s, 3H), 2.70 (m, 1H), 1.95 – 1.83 (m, 2H), 1.78 – 1.67 (m, 2H), 1.69 – 1.57 (m, 2H), 1.55 – 1.40 (m, 2H); **^{13}C NMR** (100 MHz, $CDCl_3$) δ 189.5, 163.4, 153.4, 131.1, 130.9, 123.7, 113.8, 55.6, 43.6, 32.8, 25.5; **HRMS (ESI):** m/z $[M+H]^+$ calcd for ($C_{15}H_{19}O_2^+$): 231.13796, found: 231.13791.

(*E*)-3-Cyclohexyl-1-(4-methoxyphenyl)prop-2-en-1-one (37): method **GP-IV**; a yellow

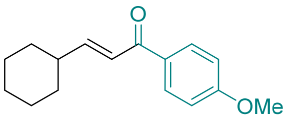

solid (30 mg, 62%); **m.p.** 64 °C; **^{1}H NMR** (400 MHz, $CDCl_3$) δ 7.94 (d, *J* = 8.9 Hz, 2H), 6.99 (dd, *J* = 15.5, 6.8 Hz, 1H), 6.94 (d, *J* = 8.9 Hz, 2H), 6.83 (dd, *J* = 15.5, 1.3 Hz, 1H), 3.87 (s, 3H), 2.24 (m, 1H), 1.87 – 1.75 (m, 4H), 1.37 – 1.19 (m, 6H); **^{13}C NMR** (100 MHz, $CDCl_3$) δ 189.7, 163.4, 154.0, 131.2, 130.9, 123.2, 113.8, 55.6, 41.2, 32.1, 26.1, 25.9; **HRMS (ESI):** m/z $[M+H]^+$ calcd for ($C_{16}H_{21}O_2^+$): 245.15361, found: 245.15356.

(*E*)-1-(4-Methoxyphenyl)-4-phenylbut-2-en-1-one (38): method **GP-IV**; a yellow oil (29 mg,

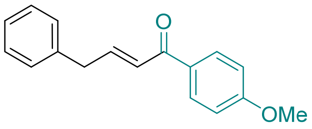

57%); **^{1}H NMR** (400 MHz, $CDCl_3$) δ 7.91 (d, *J* = 8.9 Hz, 2H), 7.38 – 7.29 (m, 2H), 7.26 – 7.21 (m, 3H), 7.20 – 7.14 (m, 1H), 6.93 (d, *J* = 8.9 Hz, 2H), 6.87 (dt, *J* = 15.3, 1.6 Hz, 1H), 3.87 (s, 3H), 3.64 (d, *J* = 6.8 Hz, 2H); **^{13}C NMR** (100 MHz, $CDCl_3$) δ 189.1, 163.5, 146.8, 131.0, 130.8, 129.0, 128.9, 126.8, 126.6, 113.9, 100.1, 55.6, 39.1; **HRMS (ESI):** m/z $[M+H]^+$ calcd for ($C_{17}H_{17}O_2^+$): 253.12231, found: 253.12215.

(*E*)-1-(4-Methoxyphenyl)-3-phenylprop-2-en-1-one (39)[18]: method **GP-IV**; a yellow solid

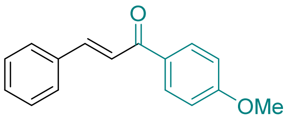

(32 mg, 67%); **^{1}H NMR** (400 MHz, $CDCl_3$) δ 8.05 (d, *J* = 8.8 Hz, 2H), 7.81 (d, *J* = 15.7 Hz, 1H), 7.68 – 7.62 (m, 2H), 7.55 (d, *J* = 15.7 Hz, 1H), 7.45 – 7.39 (m, 3H), 6.99 (d, *J* = 8.8 Hz, 2H), 3.90 (s, 3H); **^{13}C NMR** (100 MHz, $CDCl_3$) δ 188.9, 163.6, 144.1, 135.2, 131.3, 131.0, 130.5, 129.1, 128.5, 122.0, 114.0, 55.7.

(*E*)-1-(4-Methoxyphenyl)-3-(*p*-tolyl)prop-2-en-1-one (40)[19]: method **GP-IV**; a white solid

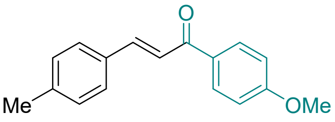

(35 mg, 69%); **^{1}H NMR** (400 MHz, $CDCl_3$) δ 8.04 (d, J = 8.9 Hz, 2H), 7.79 (d, *J* = 15.6 Hz, 1H), 7.58 – 7.45 (m, 3H), 7.22 (d, *J* = 7.9 Hz, 2H), 6.98 (d, *J* = 8.9 Hz, 2H), 3.89 (s, 3H), 2.39 (s, 3H); **^{13}C NMR** (100 MHz, $CDCl_3$) δ 188.9, 163.4, 144.1, 140.9, 132.4, 131.3, 130.9, 129.8, 128.5, 120.9, 113.9, 55.6, 21.6.

(*E*)-3-(4-Ethylphenyl)-1-(4-methoxyphenyl)prop-2-en-1-one (41)[18]: method **GP-IV**; a

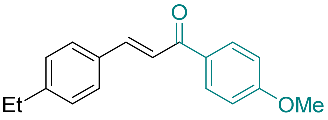

yellow solid (32 mg, 60%); **^{1}H NMR** (400 MHz, $CDCl_3$) δ 8.04 (d, *J* = 8.9 Hz, 2H), 7.80 (d, *J* = 15.6 Hz, 1H), 7.57 (d, *J* = 8.1 Hz, 2H), 7.51 (d, *J* = 15.6 Hz, 1H), 7.25 (d, *J* = 7.5 Hz, 2H), 6.99 (d, *J* = 8.9 Hz, 2H), 3.90 (s, 3H), 2.69 (q, *J* = 7.6 Hz, 2H), 1.26 (t, *J* = 7.6 Hz, 3H); **^{13}C NMR** (100 MHz, $CDCl_3$) δ 188.9, 163.5, 147.2, 144.2, 132.7, 131.4, 130.9, 128.6, 121.0, 113.9, 55.6, 29.0, 15.5.

(*E*)-3-(4-(*Tert*-butyl)phenyl)-1-(4-methoxyphenyl)prop-2-en-1-one (42)[19]: method **GP-IV**;

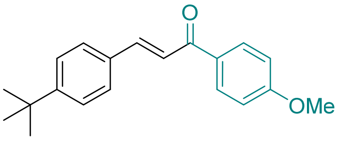

a yellow solid (41 mg, 69%); **^{1}H NMR** (400 MHz, $CDCl_3$) δ 8.04 (d, *J* = 8.9 Hz, 2H), 7.80 (d, *J* = 15.6 Hz, 1H), 7.59 (d, *J* = 8.2 Hz, 2H), 7.51 (d, *J* = 15.6 Hz, 1H), 7.44 (d, *J* = 8.4 Hz, 2H), 6.99 (d, *J* = 8.9 Hz, 2H), 3.90 (s, 3H), 1.35 (s, 9H); **^{13}C NMR** (100 MHz, $CDCl_3$) δ 189.0, 163.5, 154.1, 144.1, 132.4, 131.4, 130.9, 128.4, 126.0, 121.2, 113.9, 55.6, 35.1, 31.3.

(*E*)-1-(4-Methoxyphenyl)-3-(4-pentylphenyl)prop-2-en-1-one (43): method **GP-IV**; a

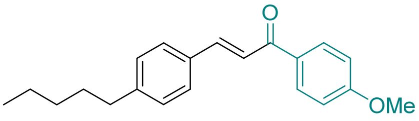

yellow solid (35 mg, 56%); **m.p.** 76 °C; **^{1}H NMR** (400 MHz, $CDCl_3$) δ 8.04 (d, *J* = 8.9 Hz, 2H), 7.79 (d, *J* = 15.6 Hz, 1H), 7.56 (d, *J* = 8.1 Hz, 2H), 7.51 (d, *J* = 15.6 Hz, 1H), 7.23 (d, *J* = 8.1 Hz, 2H), 6.98 (d, *J* = 8.8 Hz, 2H), 3.89 (s, 3H), 2.64 (t, *J* = 7.6 Hz, 2H), 1.68 – 1.61 (m, 2H), 1.37 – 1.29 (m, 4H), 0.90 (t, *J* = 7.0 Hz, 3H); **^{13}C NMR** (100 MHz, $CDCl_3$) δ 189.0, 163.5, 146.0, 144.3, 132.7, 131.4, 130.9, 129.2, 128.6, 121.1, 114.0, 55.6, 36.0, 31.6, 31.1, 22.7, 14.2; **HRMS (ESI)**: m/z $[M+H]^+$ calcd for ($C_{21}H_{25}O_2^+$): 309.18491, found: 309.18497.

(*E*)-3-([1,1'-Biphenyl]-4-yl)-1-(4-methoxyphenyl)prop-2-en-1-one (44)[20]: method **GP-IV**; a

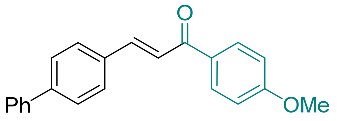

yellow solid (30 mg, 47%); **^{1}H NMR** (400 MHz, $CDCl_3$) δ 8.07 (d, J = 8.9 Hz, 2H), 7.85 (d, J = 15.6 Hz, 1H), 7.73 (d, J = 8.2 Hz, 2H), 7.66 (d, J = 8.4 Hz, 2H), 7.63 (d, J = 6.9 Hz, 2H), 7.59 (d, J = 15.6 Hz, 1H), 7.47 (t, J = 7.6 Hz, 2H), 7.38 (t, J = 7.3 Hz, 1H), 7.00 (d, J = 8.9 Hz, 2H), 3.90 (s, 3H); **^{13}C NMR** (100 MHz, $CDCl_3$) δ 188.6, 163.5, 143.5, 143.1, 140.2, 134.1, 131.2, 130.9, 129.0, 129.0, 127.9, 127.6, 127.1, 121.7, 113.9, 55.6.

(*E*)-1-(4-Methoxyphenyl)-3-(4-phenoxyphenyl)prop-2-en-1-one (45): method **GP-IV**; a

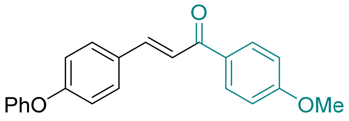

white solid (43 mg, 65%); **m.p.** 118 °C; **^{1}H NMR** (400 MHz, $CDCl_3$) δ 8.04 (d, J = 8.9 Hz, 2H), 7.79 (d, J = 15.6 Hz, 1H), 7.62 (d, J = 8.7 Hz, 2H), 7.46 (d, J = 15.6 Hz, 1H), 7.38 (dd, J = 8.6, 7.4 Hz, 2H), 7.17 (t, J = 7.4 Hz, 1H), 7.07 (d, J = 7.6 Hz, 2H), 7.02 (d, J = 8.8 Hz, 2H), 6.99 (d, J = 8.9 Hz, 2H), 3.89 (s, 3H); **^{13}C NMR** (100 MHz, $CDCl_3$) δ 188.8, 163.5, 159.7, 156.3, 143.5, 131.4, 130.9, 130.2, 130.1, 130.0, 124.3, 120.8, 119.8, 118.6, 114.0, 55.7; **HRMS (ESI):** m/z $[M+H]^+$ calcd for ($C_{22}H_{19}O_3^+$): 331.13287, found: 331.13263.

(*E*)-3-(2-Methoxyphenyl)-1-(4-methoxyphenyl)prop-2-en-1-one (46)[21]: method **GP-IV**; a

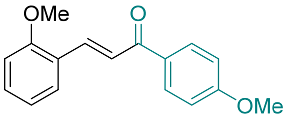

yellow oil (41 mg, 76%); **^{1}H NMR** (400 MHz, $CDCl_3$) δ 8.10 (d, J = 15.8 Hz, 1H), 8.04 (d, J = 8.8 Hz, 2H), 7.64 (dd, J = 7.7, 1.6 Hz, 1H), 7.63 (d, J = 15.8 Hz, 1H), 7.40 – 7.34 (m, 1H), 7.03 – 6.92 (m, 4H), 3.92 (s, 3H), 3.89 (s, 3H); **^{13}C NMR** (100 MHz, $CDCl_3$) δ 189.4, 163.3, 158.8, 139.6, 131.6, 131.5, 130.9, 129.3, 124.2, 122.8, 120.8, 113.9, 111.3, 55.7, 55.6.

(*E*)-3-(3-Methoxyphenyl)-1-(4-methoxyphenyl)prop-2-en-1-one (47)[21]: method **GP-IV**; a

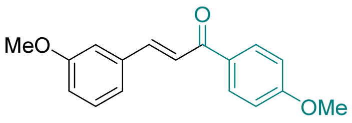

yellow solid (35 mg, 65%); **^{1}H NMR** (400 MHz, $CDCl_3$) δ 8.04 (d, J = 8.9 Hz, 2H), 7.76 (d, J = 15.6 Hz, 1H), 7.52 (d, J = 15.7 Hz, 1H), 7.34 (t, J = 7.9 Hz, 1H), 7.23 (d, J = 1.2 Hz, 1H), 7.16 (m, 1H), 7.03 – 6.92 (m, 3H), 3.90 (s, 3H), 3.86 (s, 3H); **^{13}C NMR** (100 MHz, $CDCl_3$) δ 188.8, 163.6, 160.4, 144.0, 136.6, 131.2, 131.0, 130.0, 122.3, 121.1, 116.2, 114.0, 113.5, 55.6, 55.5.

(*E*)-1,3-Bis(4-methoxyphenyl)prop-2-en-1-one (48)[21]: method **GP-IV**; a yellow solid (29 mg, 54%); **^{1}H NMR** (400 MHz, $CDCl_3$) δ 8.04 (d, *J* = 8.8 Hz, 2H), 7.78 (d, *J* = 15.6 Hz, 1H), 7.60 (d, *J* = 8.8 Hz, 2H), 7.43 (d, *J* = 15.5 Hz, 1H), 6.98 (d, *J* = 8.8 Hz, 2H), 6.94 (d, *J* = 8.7 Hz, 2H), 3.89 (s, 3H), 3.86 (s, 3H); **^{13}C NMR** (100 MHz, $CDCl_3$) δ 188.9, 163.4, 161.6, 144.0, 131.5, 130.8, 130.2, 128.0, 119.7, 114.5, 113.9, 55.6, 55.6.

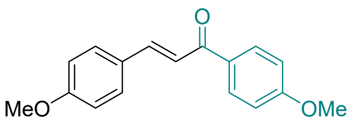

(*E*)-3-(4-Methoxy-2-methylphenyl)-1-(4-methoxyphenyl)prop-2-en-1-one (49): method **GP-IV**; a yellow solid (31 mg, 55%); **m.p.** 128 °C; **^{1}H NMR** (400 MHz, $CDCl_3$) δ 8.07 (d, *J* = 15.4 Hz, 1H), 8.04 (d, *J* = 8.7 Hz, 2H), 7.68 (d, *J* = 8.5 Hz, 1H), 7.39 (d, *J* = 15.4 Hz, 1H), 6.98 (d, *J* = 8.9 Hz, 2H), 6.83 – 6.73 (m, 2H), 3.89 (s, 3H), 3.84 (s, 3H), 2.48 (s, 3H); **^{13}C NMR** (100 MHz, $CDCl_3$) δ 188.9, 163.4, 161.3, 141.4, 140.7, 131.5, 130.9, 128.1, 126.9, 120.6, 116.0, 113.9, 112.3, 55.6, 55.5, 20.4; **HRMS (ESI):** m/z $[M+H]^+$ calcd for ($C_{18}H_{19}O_3^+$): 283.13287, found: 283.13281.

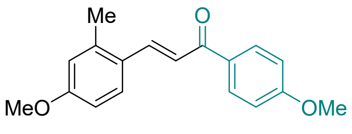

(*E*)-3-(4-Fluorophenyl)-1-(4-methoxyphenyl)prop-2-en-1-one (50)[20]: method **GP-IV**; a white solid (32 mg, 63%); **^{1}H NMR** (400 MHz, $CDCl_3$) δ 8.04 (d, *J* = 8.8 Hz, 2H), 7.77 (d, *J* = 15.6 Hz, 1H), 7.64 (dd, *J* = 8.7, 5.4 Hz, 2H), 7.47 (d, *J* = 15.7 Hz, 1H), 7.11 (t, *J* = 8.6 Hz, 2H), 6.99 (d, *J* = 8.9 Hz, 2H), 3.90 (s, 3H); **^{13}C NMR** (100 MHz, $CDCl_3$) δ 188.6, 164.0 (d, *J* = 251.4 Hz), 163.6, 142.7, 131.5 (d, *J* = 3.3 Hz), 131.1, 130.9, 130.3 (d, *J* = 8.4 Hz), 121.7 (d, *J* = 2.3 Hz), 116.2 (d, *J* = 21.9 Hz), 114.0, 55.6.

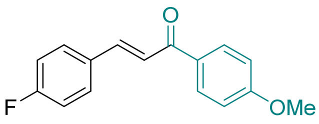

(*E*)-3-(4-Chlorophenyl)-1-(4-methoxyphenyl)prop-2-en-1-one (51)[20]: method **GP-IV**; a yellow solid (31 mg, 56%); **^{1}H NMR** (400 MHz, $CDCl_3$) δ 8.04 (d, *J* = 8.8 Hz, 2H), 7.75 (d, *J* = 15.7 Hz, 1H), 7.58 (d, *J* = 8.4 Hz, 2H), 7.52 (d, *J* = 15.6 Hz, 1H), 7.39 (d, *J* = 8.5 Hz, 2H), 6.99 (d, *J* = 8.9 Hz, 2H), 3.90 (s, 3H); **^{13}C NMR** (100 MHz, $CDCl_3$) δ 188.4, 163.6, 142.5, 136.3, 133.7, 131.0, 130.9, 129.6, 129.3, 122.4, 114.0, 55.6.

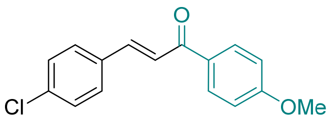

(*E*)-1-(4-Methoxyphenyl)-3-(4-(trifluoromethyl)phenyl)prop-2-en-1-one (52)[21]: method

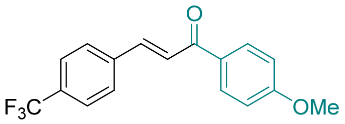

GP-III; a yellow solid (24 mg, 39%); **^{1}H NMR** (400 MHz, $CDCl_3$) δ 8.05 (d, *J* = 8.9 Hz, 2H), 7.80 (d, *J* = 15.7 Hz, 1H), 7.74 (d, *J* = 8.4 Hz, 2H), 7.67 (d, *J* = 8.2 Hz, 2H), 7.61 (d, *J* = 15.7 Hz, 1H), 7.00 (d, *J* = 8.9 Hz, 2H), 3.90 (s, 3H); **^{13}C NMR** (100 MHz, $CDCl_3$) δ 188.3, 163.8, 142.0, 138.6, 131.8 (q, *J* = 32.6 Hz), 131.0, 130.8, 128.5, 126.0 (q, *J* = 3.7 Hz), 124.2, 124.0 (q, *J* = 272.2 Hz), 114.1, 55.7.

(*E*)-3-(3,5-Bis(trifluoromethyl)phenyl)-1-(4-methoxyphenyl)prop-2-en-1-one (53)[22]:

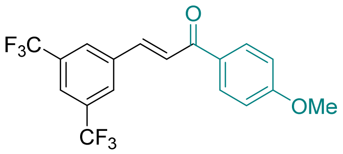

method **GP-IV**; a brown oil (17 mg, 23%); **^{1}H NMR** (400 MHz, $CDCl_3$) δ 8.07 (d, *J* = 8.9 Hz, 2H), 8.04 (s, 2H), 7.89 (s, 1H), 7.81 (d, *J* = 15.7 Hz, 1H), 7.65 (d, *J* = 15.7 Hz, 1H), 7.01 (d, *J* = 8.9 Hz, 2H), 3.91 (s, 3H); **^{13}C NMR** (100 MHz, $CDCl_3$) δ 187.7, 164.0, 140.2, 137.4, 132.6 (q, *J* = 33.5 Hz), 131.2, 130.5, 128.0 (m), 125.4, 123.4 (m), 123.2 (q, *J* = 272.9 Hz), 114.2, 55.7.

(*E*)-3-(6-Methoxynaphthalen-2-yl)-1-(4-methoxyphenyl)prop-2-en-1-one (54): method

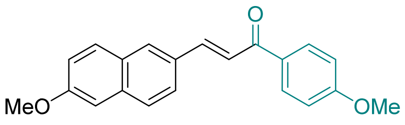

GP-IV; a yellow solid (48 mg, 76%); **m.p.** 167 °C; **^{1}H NMR** (400 MHz, $CDCl_3$) δ 8.08 (d, *J* = 8.9 Hz, 2H), 7.95 (d, *J* = 15.4 Hz, 2H), 7.80 – 7.73 (m, 3H), 7.62 (d, *J* = 15.6 Hz, 1H), 7.21 – 7.13 (m, 2H), 7.00 (d, *J* = 8.9 Hz, 2H), 3.95 (s, 3H), 3.91 (s, 3H); **^{13}C NMR** (100 MHz, $CDCl_3$) δ 188.9, 163.5, 159.0, 144.5, 135.9, 131.5, 130.9, 130.6, 130.4, 130.3, 128.9, 127.6, 124.6, 121.1, 119.6, 114.0, 106.2, 55.7, 55.6; **HRMS (ESI):** m/z $[M+H]^+$ calcd for ($C_{21}H_{19}O_3^+$): 319.13287, found: 319.13293.

(*E*)-1-(4-Methoxyphenyl)-3-(thiophen-2-yl)prop-2-en-1-one (55)[20]: method **GP-IV**; a

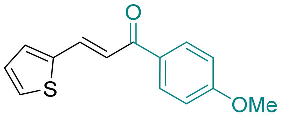

brown solid (19 mg, 39%); **^{1}H NMR** (400 MHz, $CDCl_3$) δ 8.02 (d, *J* = 8.9 Hz, 2H), 7.93 (d, *J* = 15.3 Hz, 1H), 7.40 (d, *J* = 5.1 Hz, 1H), δ 7.35 (d, *J* = 3.6 Hz, 1H), δ 7.35 (d, *J* = 15.3 Hz, 1H), 7.09 (dd, *J* = 5.1, 3.6 Hz, 1H), 6.98 (d, *J* = 8.9 Hz, 2H), 3.89 (s, 3H); **^{13}C NMR** (100 MHz, $CDCl_3$) δ 188.2, 163.6, 140.7, 136.5, 131.9, 131.1, 130.9, 128.6, 128.4, 120.8, 114.0, 55.6.

(*E*)-1-(4-Methoxyphenyl)-3-(thiophen-3-yl)prop-2-en-1-one (56)[23]: method **GP-IV**; an orange oil (28 mg, 58%); **^{1}H NMR** (400 MHz, $CDCl_3$) δ 8.02 (d, *J* = 8.9 Hz, 2H), 7.79 (d, *J* = 15.5 Hz, 1H), 7.59 (dd, *J* = 3.0, 1.3 Hz, 1H), 7.42 (dd, *J* = 5.1, 1.3 Hz, 1H), δ 7.38 – 7.36 (m, 1H), δ 7.36 (d, *J* = 15.6 Hz, 1H), 6.98 (d, *J* = 8.9 Hz, 2H), 3.89 (s, 3H); **^{13}C NMR** (100 MHz, $CDCl_3$) δ 189.1, 163.5, 138.5, 137.6, 131.3, 130.9, 128.9, 127.1, 125.4, 121.8, 55.7.

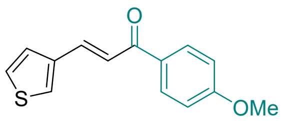

(*E*)-3-(3-Hydroxyphenyl)-1-(4-methoxyphenyl)prop-2-en-1-one (57)[24]: method **GP-IV**; a yellow solid (31 mg, 59%); **^{1}H NMR** (400 MHz, DMSO-d_6) δ 9.62 (s, 1H), 8.16 (d, *J* = 8.9 Hz, 2H), 7.84 (d, *J* = 15.6 Hz, 1H), 7.61 (d, *J* = 15.5 Hz, 1H), 7.31 (d, *J* = 7.7 Hz, 1H), 7.25 (t, *J* = 7.7 Hz, 1H), 7.22 (t, *J* = 2.0 Hz, 1H), 7.09 (d, *J* = 9.0 Hz, 2H), 6.87 (d, *J* = 7.8 Hz, 1H), 3.87 (s, 3H); **^{13}C NMR** (100 MHz, DMSO-d_6) δ 187.4, 163.2, 157.8, 143.4, 136.1, 130.9, 130.5, 129.9, 121.9, 119.8, 117.7, 115.2, 114.0, 55.6.

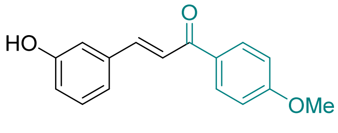

(8*R*,9*S*,10*R*,13*S*,14*S*,17*R*)-17-Hydroxy-17-((*E*)-3-(4-methoxyphenyl)-3-oxoprop-1-en-1-yl)-10,13-dimethyl-1,2,6,7,8,9,10,11,12,13,14,15,16,17-tetradecahydro-3*H*-cyclopenta[*a*]phenanthren-3-one (58): method **1 mmol scale**; a white solid (263 mg, 59%); **m.p.** 116 °C; **^{1}H NMR** (400 MHz, $CDCl_3$) δ 8.00 (d, *J* = 8.9 Hz, 2H), δ 7.23 (d, *J* = 15.2 Hz, 1H), 7.17 (d, *J* = 15.1 Hz, 1H), 6.95 (d, *J* = 8.9 Hz, 2H), 5.73 (s, 1H), 3.88 (s, 3H), 2.44 – 2.27 (m, 4H), 2.05 – 1.95 (m, 2H), 1.94 – 1.82 (m, 2H), 1.71 – 1.36 (m, 10H), 1.19 (s, 3H), 1.12 – 1.06 (m, 1H), 1.01 (s, 3H), 0.97 – 0.92 (m, 1H); **^{13}C NMR** (100 MHz, $CDCl_3$) δ 199.6, 188.7, 170.9, 163.7, 151.8, 131.1, 124.2, 121.7, 114.0, 84.6, 55.6, 53.5, 50.4, 47.7, 38.7, 37.7, 36.5, 35.8, 34.1, 32.9, 32.7, 31.7, 29.9, 24.0, 20.8, 17.6, 14.0; **HRMS (ESI):** m/z $[M+H]^+$ calcd for ($C_{29}H_{37}O_4^+$): 449.26864, found: 449.26852.

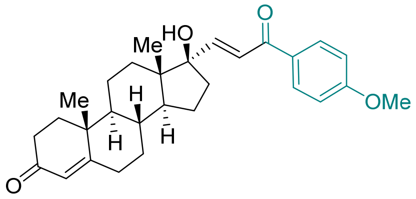

(*E*)-1-(4-Methoxyphenyl)-2-methyl-3-phenylprop-2-en-1-one (59)[25]: method **GP-III**; a colorless oil (15 mg, 30%); **^{1}H NMR** (400 MHz, $CDCl_3$) δ 7.81 (d, *J* = 8.8 Hz, 2H), 7.44 – 7.39 (m, 4H), 7.36 – 7.31 (m, 1H), 7.11 (d, *J* = 1.5 Hz, 1H), 6.95 (d, *J* = 8.8 Hz, 2H), 3.88 (s, 3H), 2.26 (d, *J* = 1.5 Hz, 3H); **^{13}C NMR** (100 MHz, $CDCl_3$) δ 198.3, 162.8, 140.1, 136.9, 135.9, 132.0, 130.6, 129.6, 128.5, 128.4, 113.5, 55.4, 14.9.

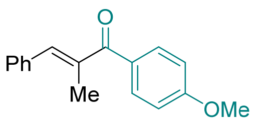

(*E*)-1-(4-Methoxyphenyl)-2,3-diphenylprop-2-en-1-one (60)[26]: method **GP-III**; a colorless oil (8 mg, 13%); **^{1}H NMR** (400 MHz, $CDCl_3$) δ 7.90 (d, *J* = 8.9 Hz, 2H), 7.34 – 7.28 (m, 5H), 7.22 – 7.16 (m, 3H), 7.14 (s, 1H), 7.11 (d, *J* = 7.5 Hz, 2H), 6.92 (d, *J* = 8.8 Hz, 2H), 3.87 (s, 3H); ^{13}C NMR (100 MHz, $CDCl_3$) δ 196.4, 163.2, 141.1, 138.0, 136.9, 135.1, 132.5, 130.6, 130.3, 129.7, 128.9, 128.8, 128.4, 128.0, 113.7, 55.6.

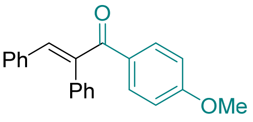

(*E*)-1-(4-Methoxyphenyl)-2-propylhex-2-en-1-one (61)[25]: method **GP-III**; a colorless oil (5 mg, 10%); **^{1}H NMR** (400 MHz, $CDCl_3$) δ 7.71 (d, *J* = 8.8 Hz, 2H), 6.91 (d, *J* = 8.8 Hz, 2H), 6.10 (t, *J* = 7.3 Hz, 1H), 3.86 (s, 3H), 2.50 – 2.39 (m, 2H), 2.26 (m, 2H), 1.46 (m, 4H), 1.00 – 0.90 (m, 6H); **^{13}C NMR** (100 MHz, $CDCl_3$) δ 198.2, 162.7, 143.3, 141.3, 131.9, 131.5, 113.4, 55.6, 30.8, 29.3, 22.5, 22.3, 14.3, 14.2.

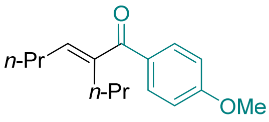

Methyl 4-(4-methoxyphenyl)-4-oxobutanoate (62)[27]: method **GP-III**; a white solid (29 mg, 66%); **^{1}H NMR** (400 MHz, $CDCl_3$) δ 7.95 (d, *J* = 8.9 Hz, 2H), 6.92 (d, *J* = 8.9 Hz, 2H), 3.86 (s, 3H), 3.69 (s, 3H), 3.26 (t, *J* = 6.7 Hz, 2H), 2.74 (t, *J* = 6.7 Hz, 2H); **^{13}C NMR** (100 MHz, $CDCl_3$) δ 196.7, 173.7, 163.7, 130.4, 129.8, 113.9, 55.6, 52.0, 33.2, 28.3.

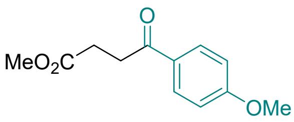

Ethyl 4-(4-methoxyphenyl)-4-oxobutanoate (63)[28]: method **GP-III**; a white solid (31 mg, 65%); **^{1}H NMR** (400 MHz, $CDCl_3$) δ 7.96 (d, *J* = 8.9 Hz, 2H), 6.93 (d, *J* = 8.9 Hz, 2H), 4.15 (q, *J* = 7.1 Hz, 2H), 3.86 (s, 3H), 3.26 (t, *J* = 6.7 Hz, 2H), 2.73 (t, *J* = 6.7 Hz, 2H), 1.25 (t, *J* = 7.1 Hz, 3H); **^{13}C NMR** (100 MHz, $CDCl_3$) δ 196.7, 173.2, 163.7, 130.4, 129.8, 113.8, 60.7, 55.6, 33.1, 28.5, 14.3.

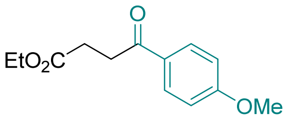

4-(4-Methoxyphenyl)-4-oxobutanenitrile (64)[4]: method **GP-III**; a white solid (28 mg, 74%); **^{1}H NMR** (400 MHz, $CDCl_3$) δ 7.92 (d, *J* = 9.0 Hz, 2H), 6.95 (d, *J* = 9.0 Hz, 2H), 3.87 (s, 3H), 3.36 – 3.27 (t, *J* = 7.2 Hz, 2H), 2.79 – 2.70 (t, *J* = 7.2 Hz, 2H); **^{13}C NMR** (100 MHz, $CDCl_3$) δ 193.9, 164.1, 130.4, 128.7, 119.5, 114.0, 55.6, 33.9, 11.9.

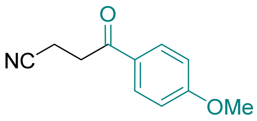

(2*Z*,4*E*)-1-(4-Methoxyphenyl)-2,3,4,5-tetraphenylpenta-2,4-dien-1-one (65): a yellow solid

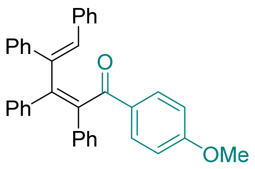

(16 mg, 16%); **m.p.** 70 °C; **^{1}H NMR** (400 MHz, $CDCl_3$) δ 7.61 (d, *J* = 7.2 Hz, 2H), 7.47 (d, *J* = 6.2 Hz, 2H), 7.33 – 7.30 (m, 2H), 7.25 – 7.07 (m, 16H), 6.91 (s, 1H), 6.35 (d, *J* = 9.0 Hz, 2H), 3.68 (s, 3H); **^{13}C NMR** (100 MHz, $CDCl_3$) δ 195.5, 162.2, 143.0, 141.8, 140.9, 140.7, 138.0, 137.9, 137.8, 132.0, 131.2, 130.6, 129.5, 128.8, 128.8, 128.4, 128.2, 128.2, 128.1, 128.0, 127.9, 127.7, 127.7, 127.3, 113.0, 55.3; **HRMS (ESI):** m/z $[M+H]^+$ calcd for ($C_{36}H_{29}O_2^+$): 493.21621, found: 493.21655.

1-(4-Methoxyphenyl)-2,3,4,5-tetraphenylcyclopenta-2,4-dien-1-ol (66): a white solid (39

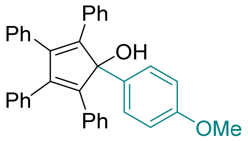

mg, 40%); **m.p.** 200 °C; **^{1}H NMR** (400 MHz, $CDCl_3$) δ 7.47 (d, *J* = 8.8 Hz, 2H), 7.19 – 6.93 (m, 20H), 6.80 (d, *J* = 8.9 Hz, 2H), 3.77 (s, 3H); **^{13}C NMR** (100 MHz, $CDCl_3$) δ 158.6, 148.0, 142.3, 135.2 134.1, 132.1, 130.1, 129.7, 128.0, 127.9, 127.2, 127.1, 126.4, 114.0, 90.1, 55.3; **HRMS (ESI):** m/z $[M+H]^+$ calcd for ($C_{36}H_{29}O_2^+$): 493.21621, found: 493.21616.

VIII-2. Additional enone products from Lewis-basic moiety bearing thioester S31

(*E*)-1-(4-(Dimethylamino)phenyl)-4,4-dimethylpent-2-en-1-one (13’): method **GP-IV**; a

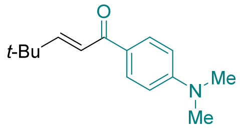

yellow solid (17 mg, 38%); **m.p.** 90 °C; **^{1}H NMR** (400 MHz, $CDCl_3$) δ 7.91 (d, *J* = 9.1 Hz, 2H), 7.00 (d, *J* = 15.5 Hz, 1H), 6.81 (d, *J* = 15.5 Hz, 1H), 6.65 (d, *J* = 9.1 Hz, 2H), 3.04 (s, 6H), 1.14 (s, 9H); **^{13}C NMR** (100 MHz, $CDCl_3$) δ 189.0, 157.0, 153.3, 130.8, 125.9, 120.6, 110.8, 40.1, 34.0, 29.0; **HRMS (ESI):** m/z $[M+H]^+$ calcd for ($C_{15}H_{22}NO^+$): 232.1696, found: 232.16919.

(*E*)-1-(4-(Dimethylamino)phenyl)-3-phenylprop-2-en-1-one (13’’)[29]: method **GP-IV**; a

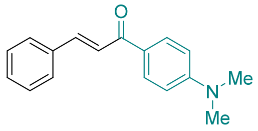

yellow solid (14 mg, 28%); **^{1}H NMR** (400 MHz, $CDCl_3$) δ 8.02 (d, *J* = 8.5 Hz, 2H), 7.79 (d, *J* = 15.6 Hz, 1H), 7.64 (d, *J* = 7.2 Hz, 2H), 7.60 (d, *J* = 15.8 Hz, 1H), 7.48 – 7.31 (m, 3H), 6.70 (d, *J* = 8.5 Hz, 2H), 3.07 (s, 6H); **^{13}C NMR** (100 MHz, $CDCl_3$) δ 187.8, 153.5, 142.5, 135.6, 130.9, 130.0, 128.9, 128.3, 126.0, 122.2, 110.9, 40.1.

Supplementary Note
IX. NMR spectra data

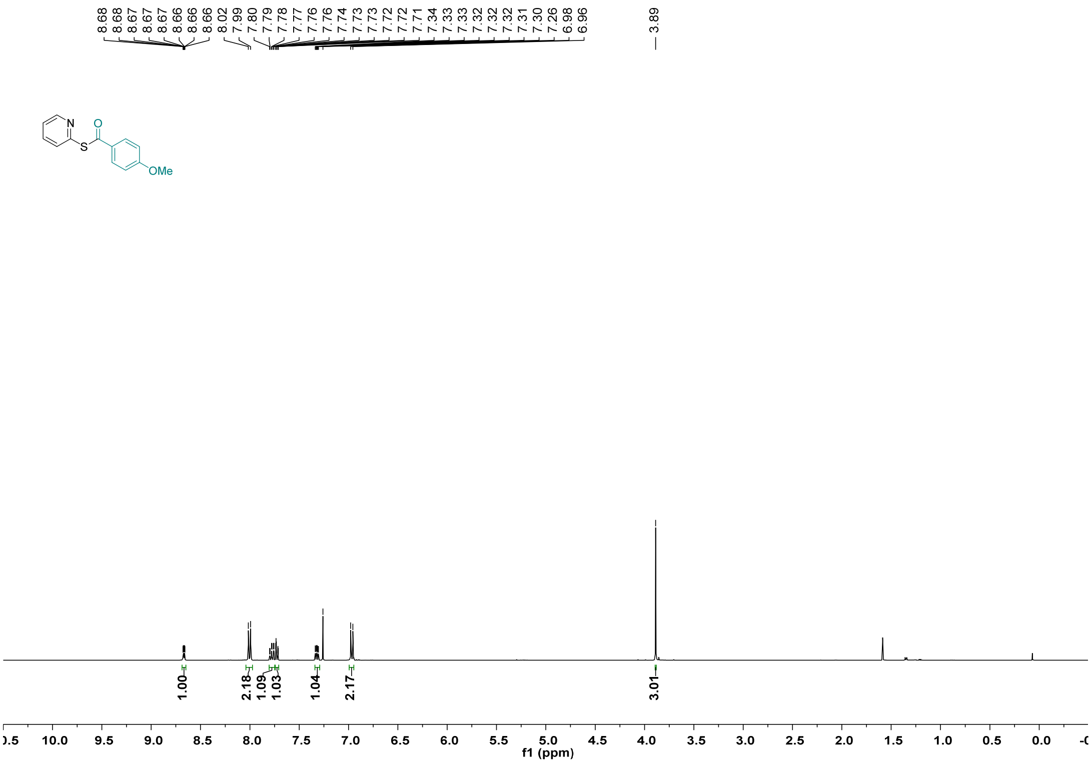

^1H NMR (400 MHz, $CDCl_3$) of compound **1**

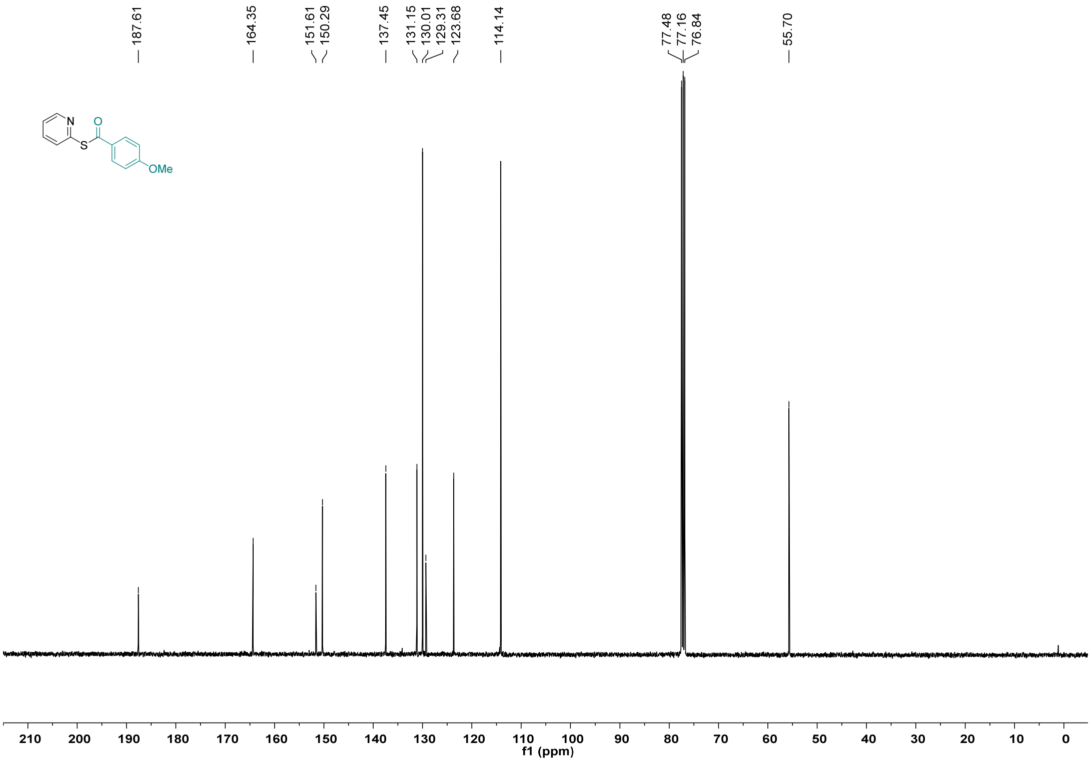

^{13}C NMR (100 MHz, $CDCl_3$) of compound **1**

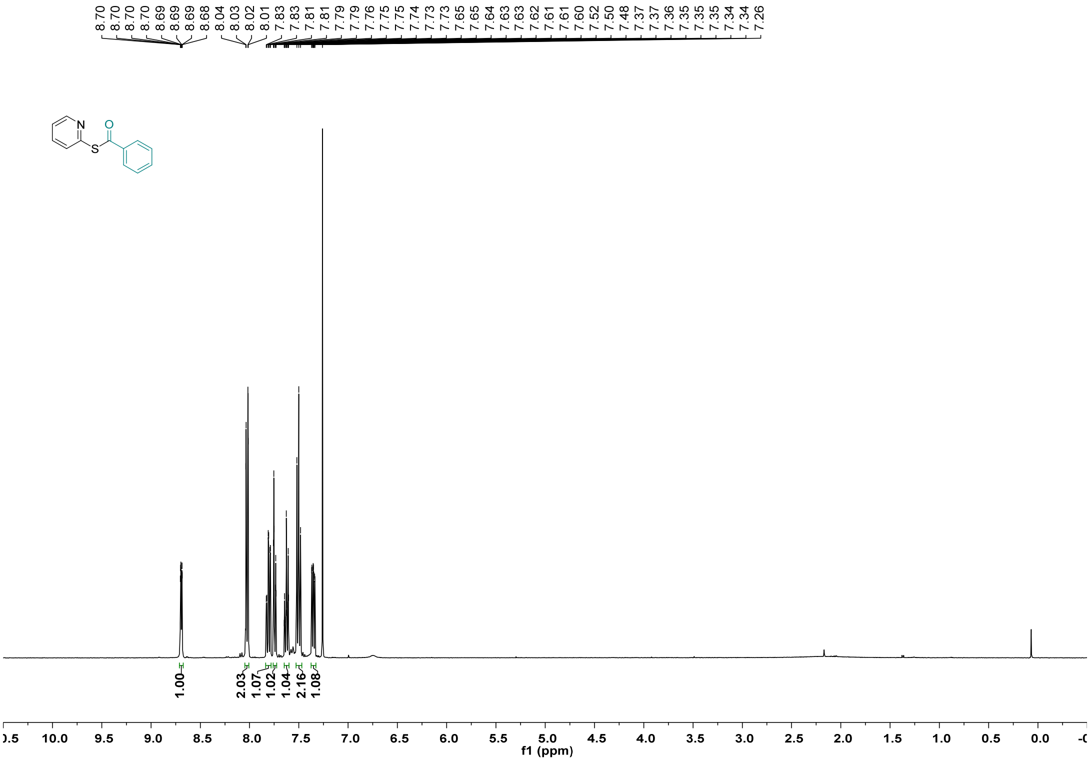

^{1}H NMR (400 MHz, $CDCl_3$) of compound **S2**

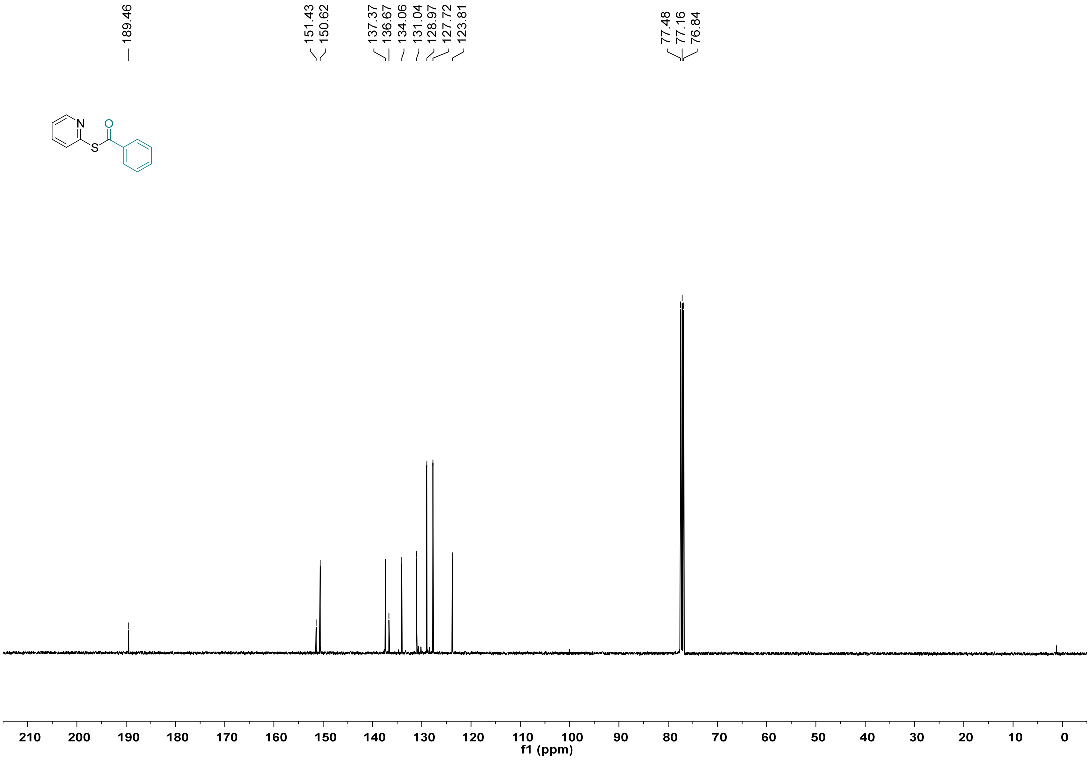

^{13}C NMR (100 MHz, $CDCl_3$) of compound **S2**

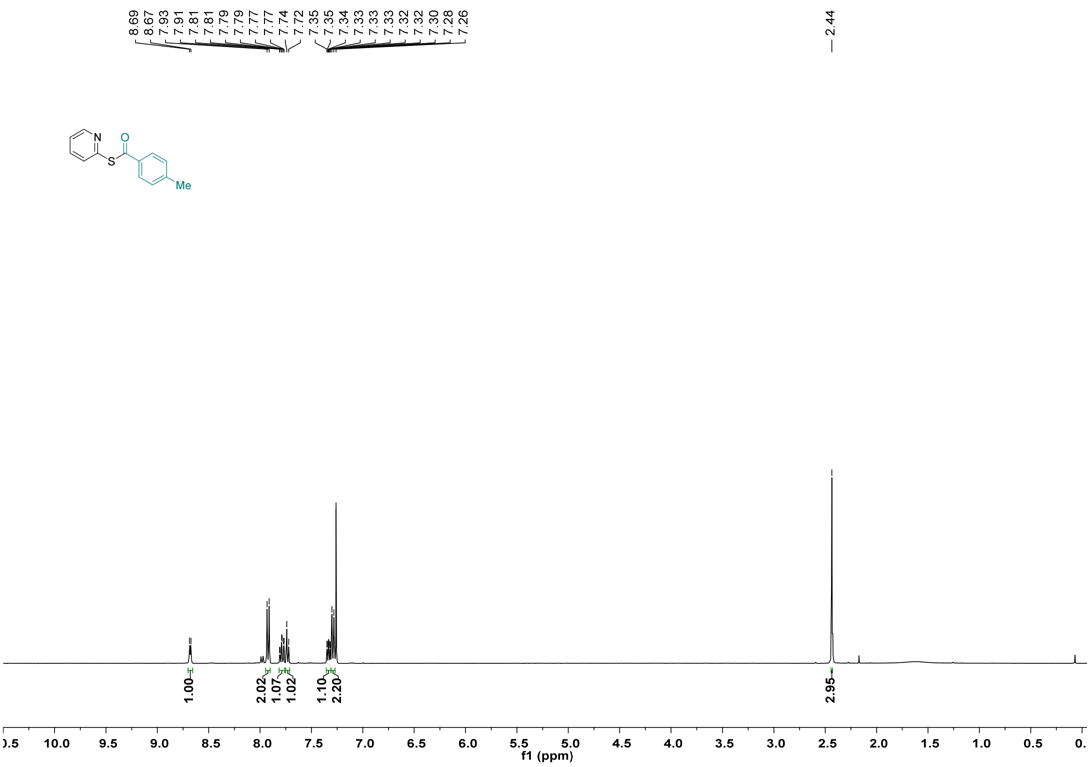

^{1}H NMR (400 MHz, $CDCl_3$) of compound **S3**

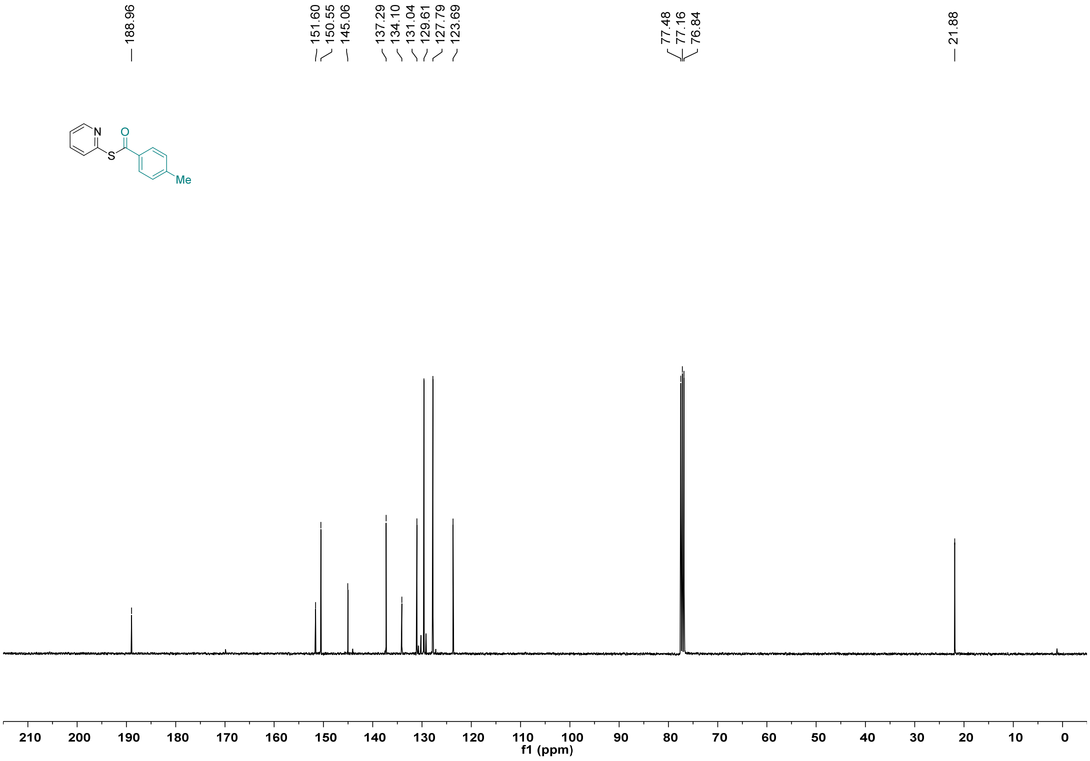

^{13}C NMR (100 MHz, $CDCl_3$) of compound **S3**

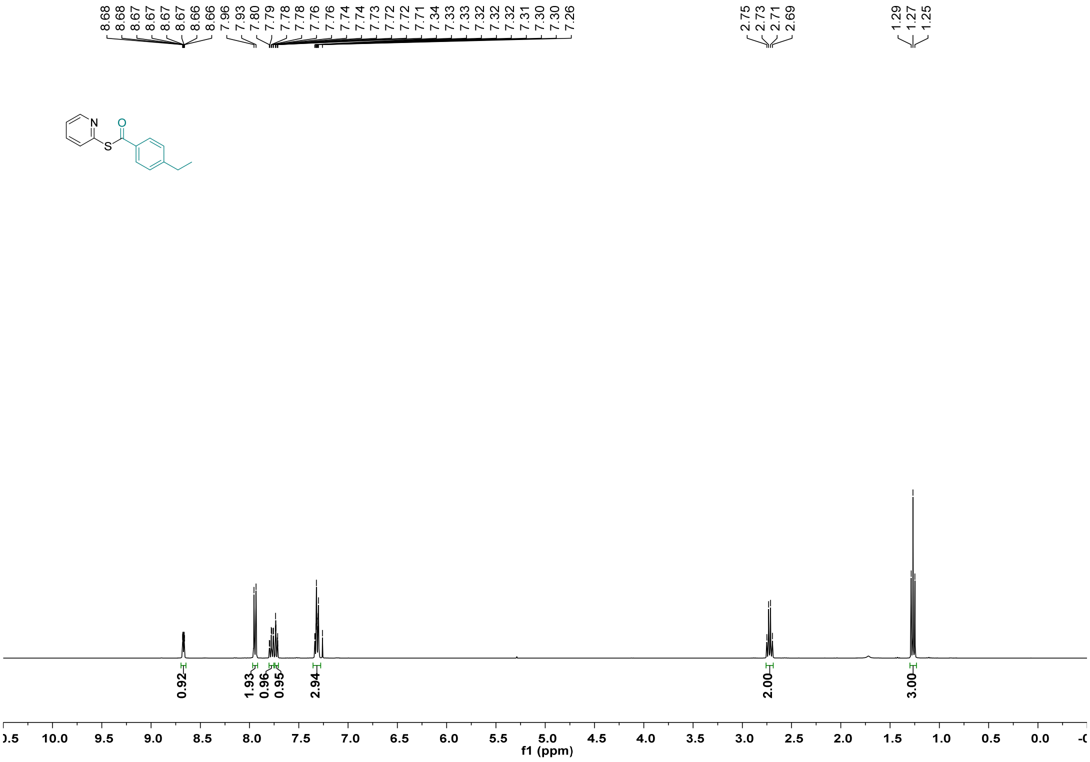

^{1}H NMR (400 MHz, $CDCl_3$) of compound **S4**

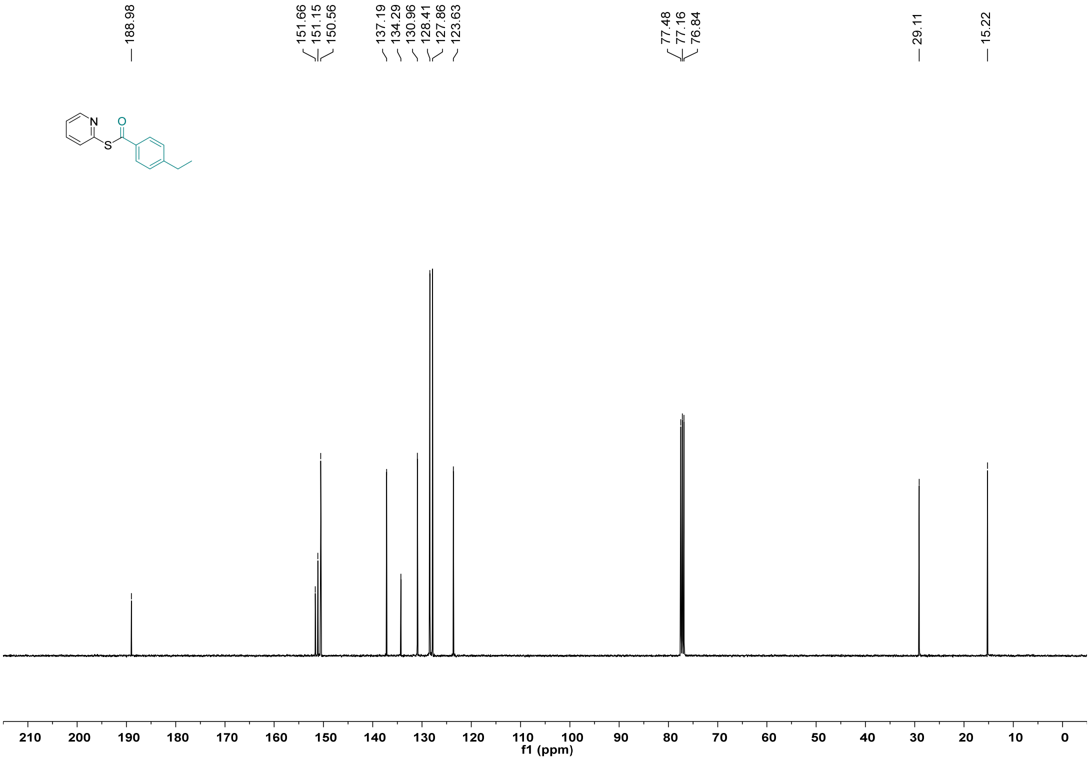

^{13}C NMR (100 MHz, $CDCl_3$) of compound **S4**

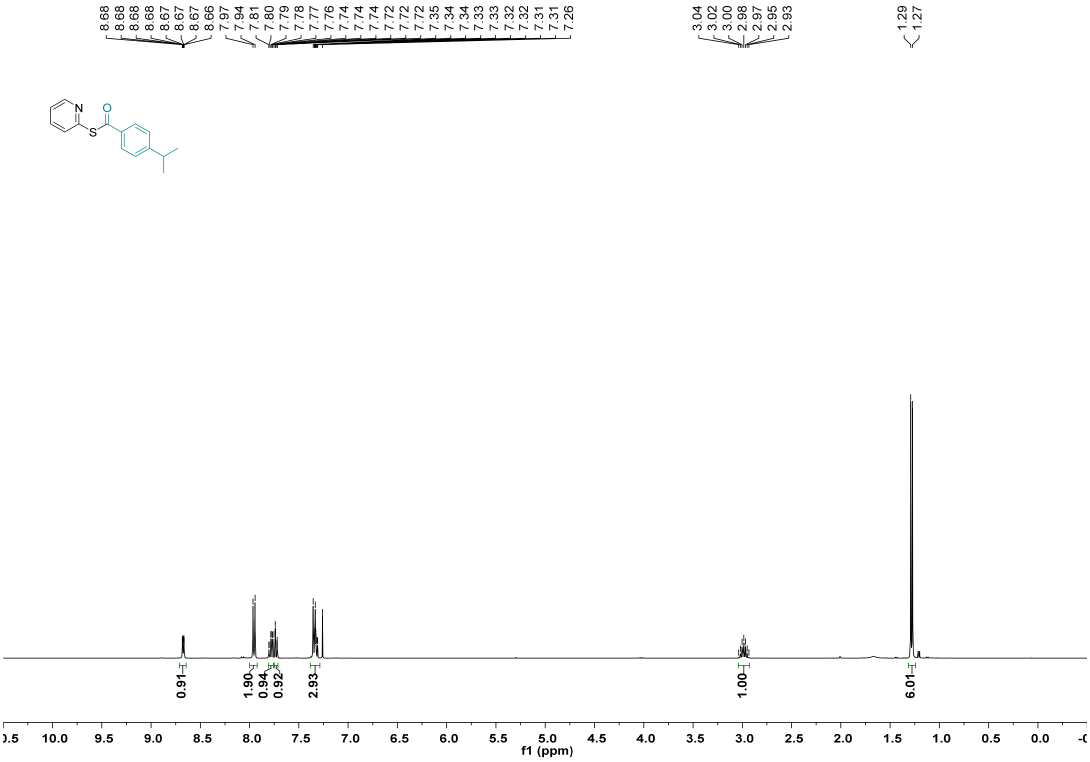

^{1}H NMR (400 MHz, $CDCl_3$) of compound **S5**

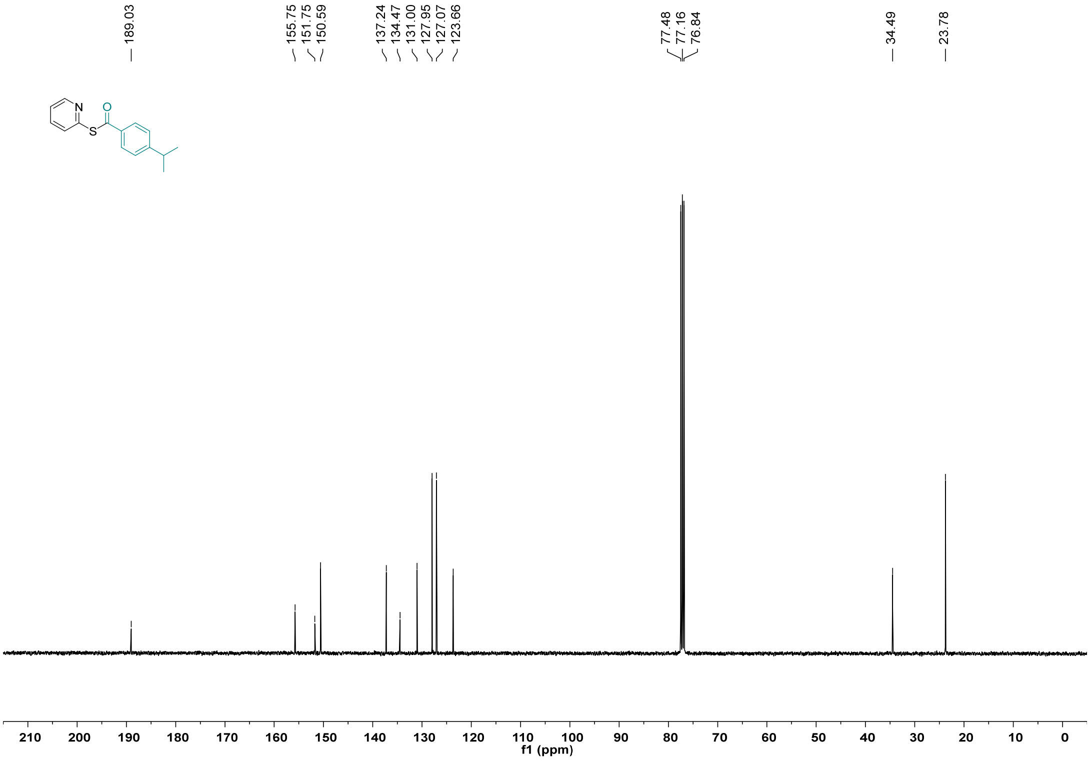

^{13}C NMR (100 MHz, $CDCl_3$) of compound **S5**

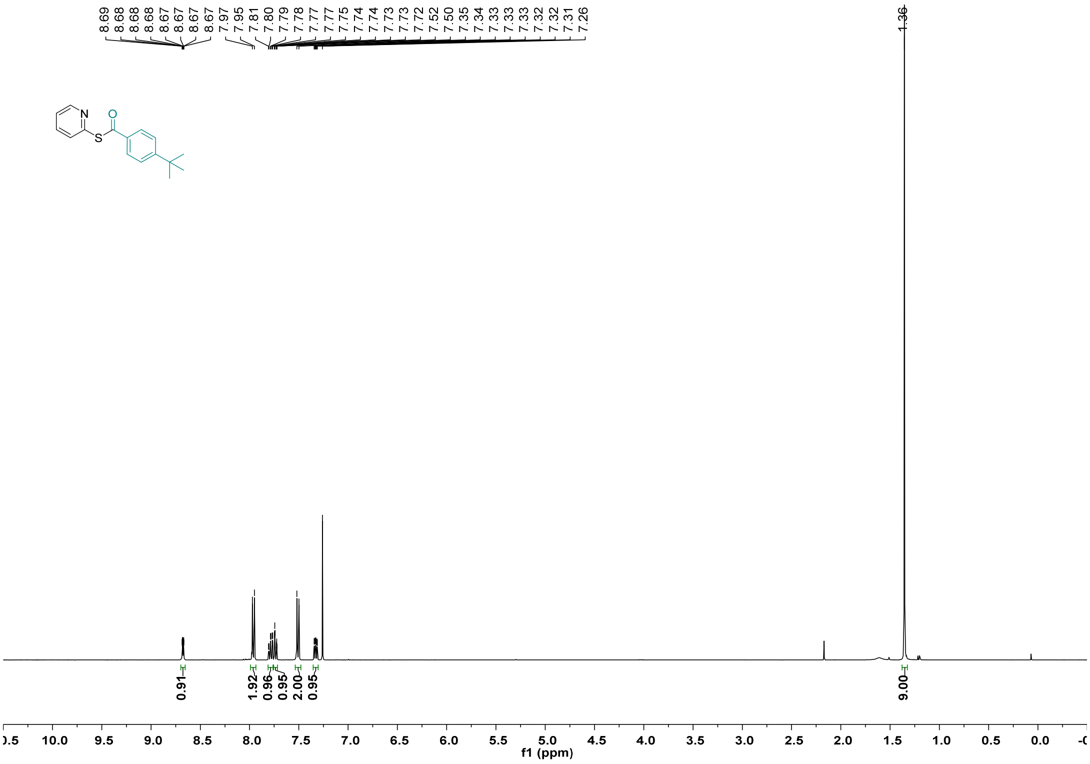

^{1}H NMR (400 MHz, CDCl$_3$) of compound **S6**

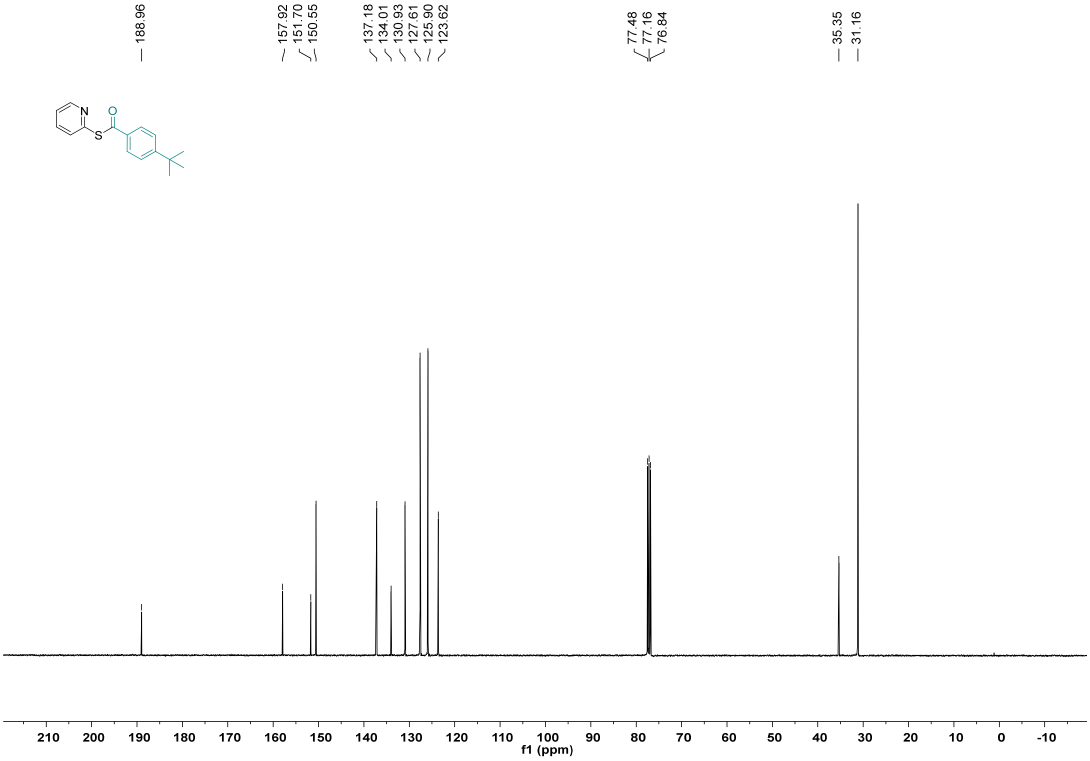

^{13}C NMR (100 MHz, CDCl$_3$) of compound **S6**

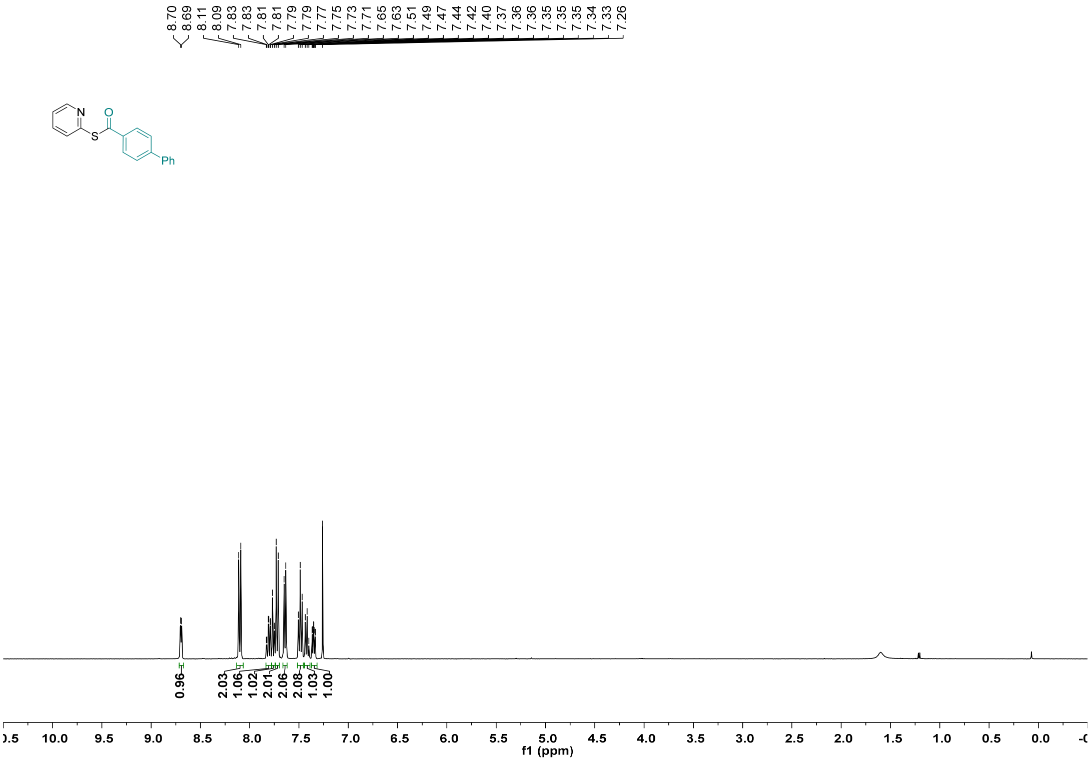

^{1}H NMR (400 MHz, $CDCl_3$) of compound **S7**

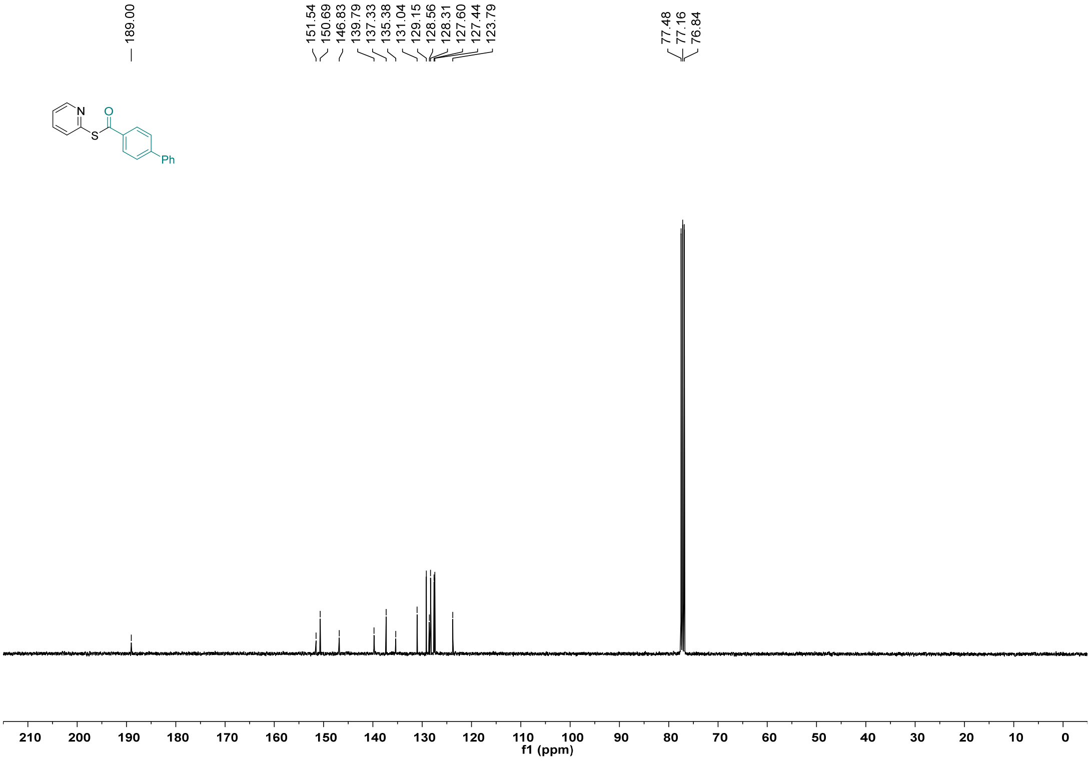

^{13}C NMR (100 MHz, $CDCl_3$) of compound **S7**

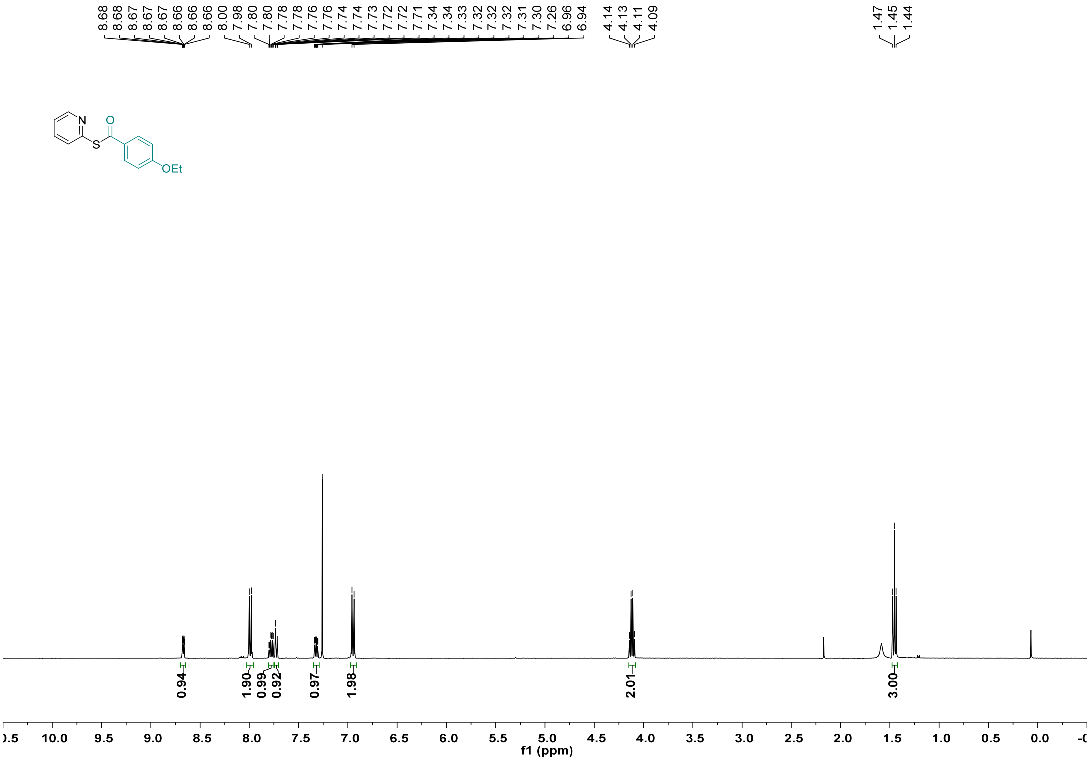

^{1}H NMR (400 MHz, $CDCl_3$) of compound **S8**

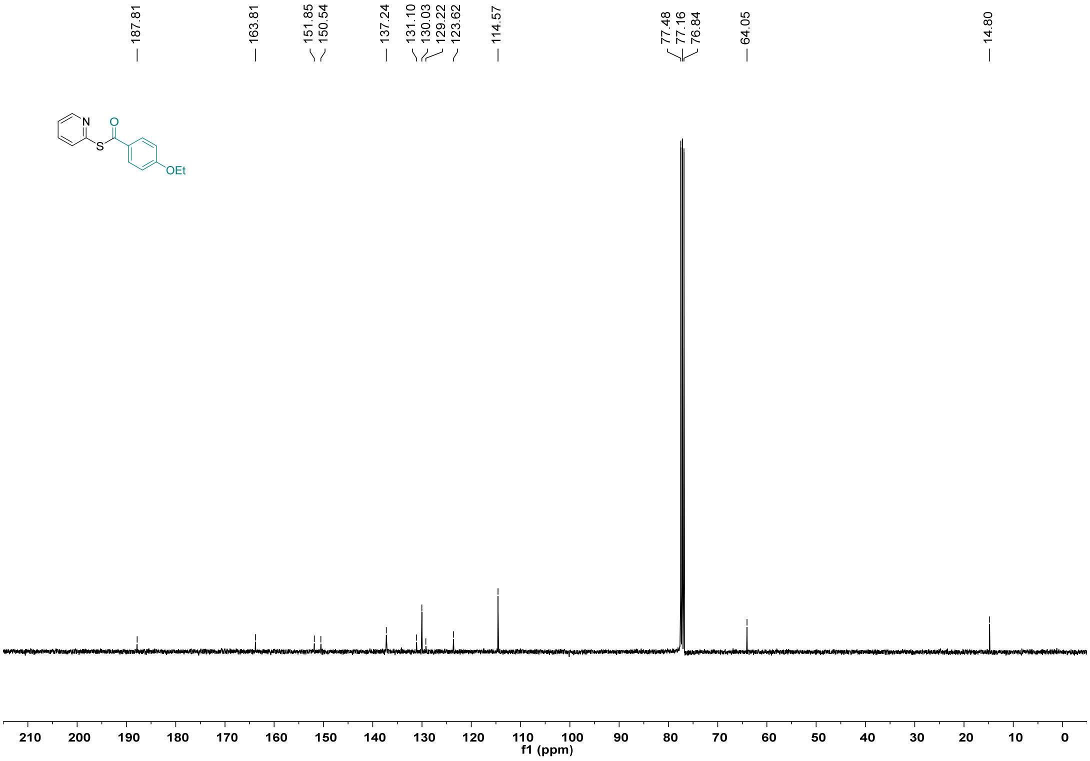

^{13}C NMR (100 MHz, $CDCl_3$) of compound **S8**

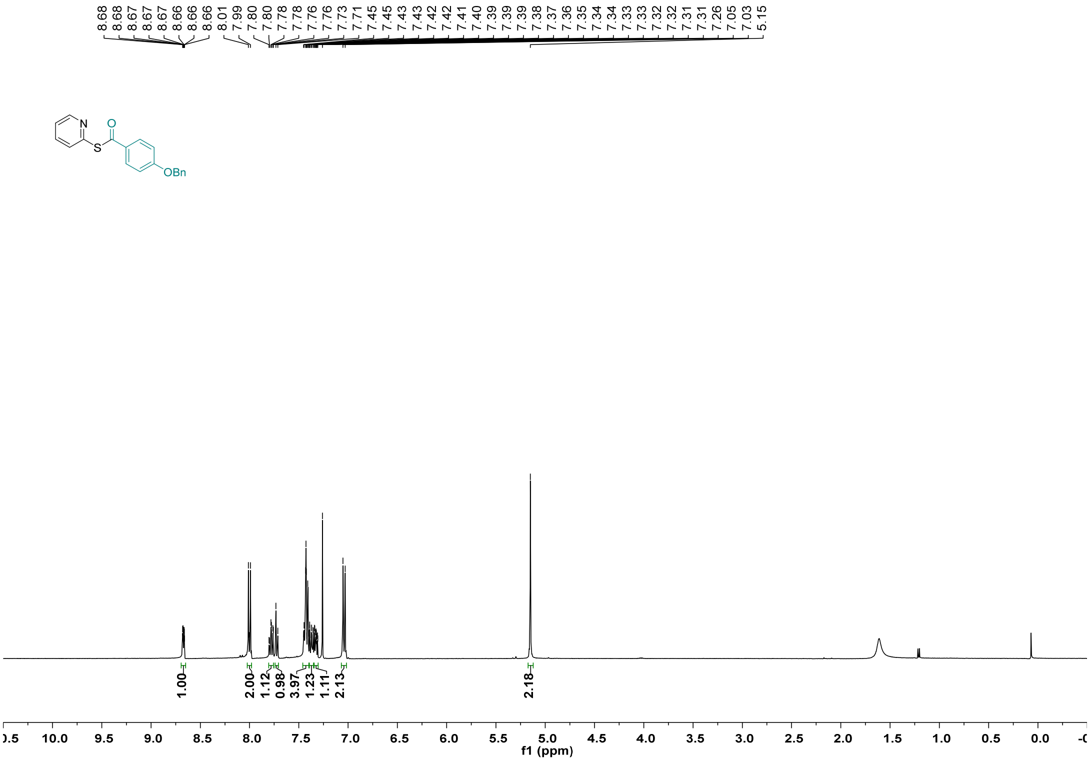

^{1}H NMR (400 MHz, $CDCl_3$) of compound **S9**

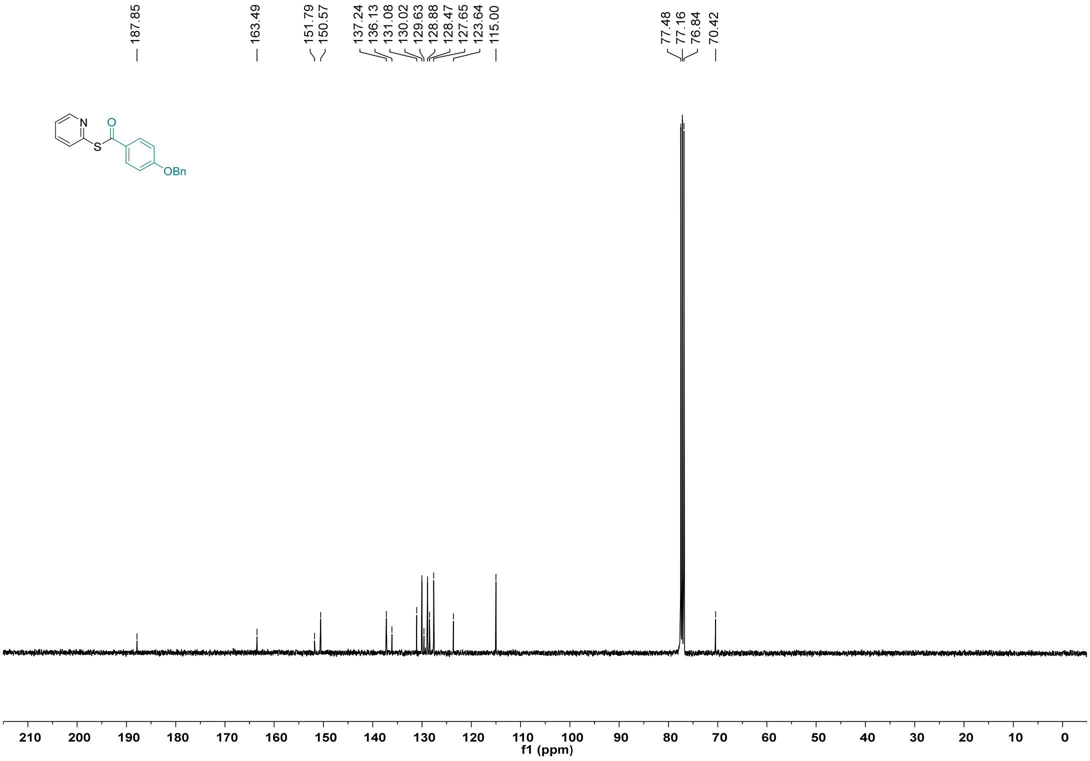

^{13}C NMR (100 MHz, $CDCl_3$) of compound **S9**

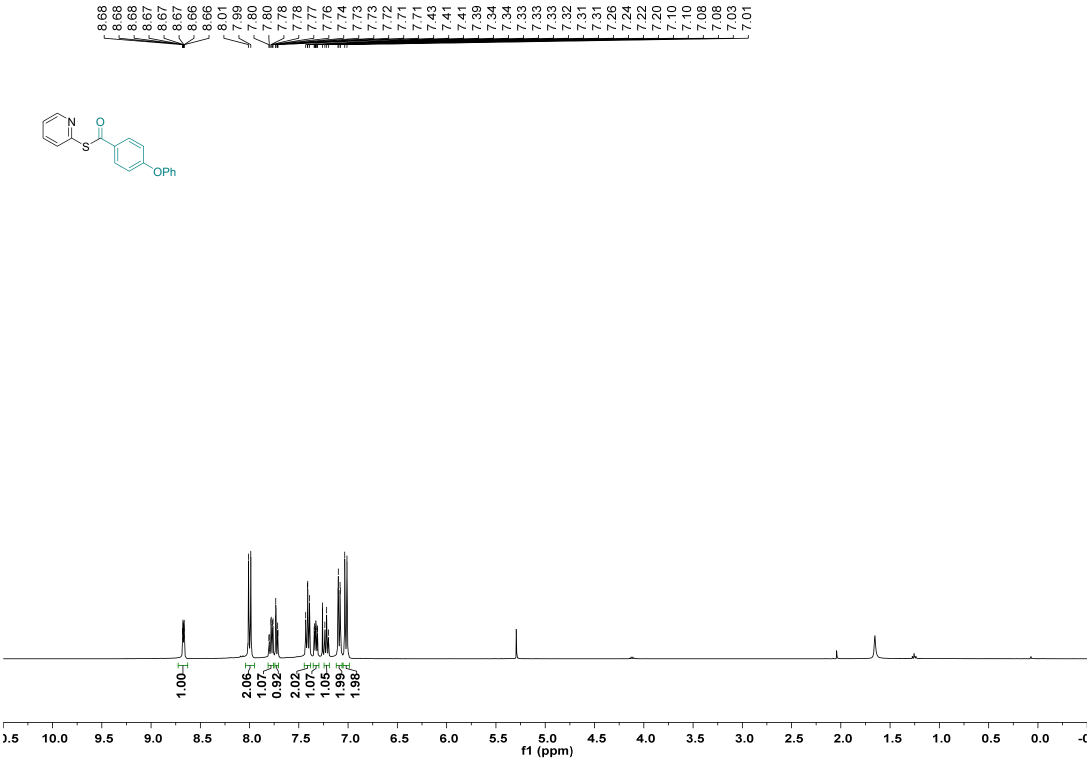

^{1}H NMR (400 MHz, $CDCl_3$) of compound **S10**

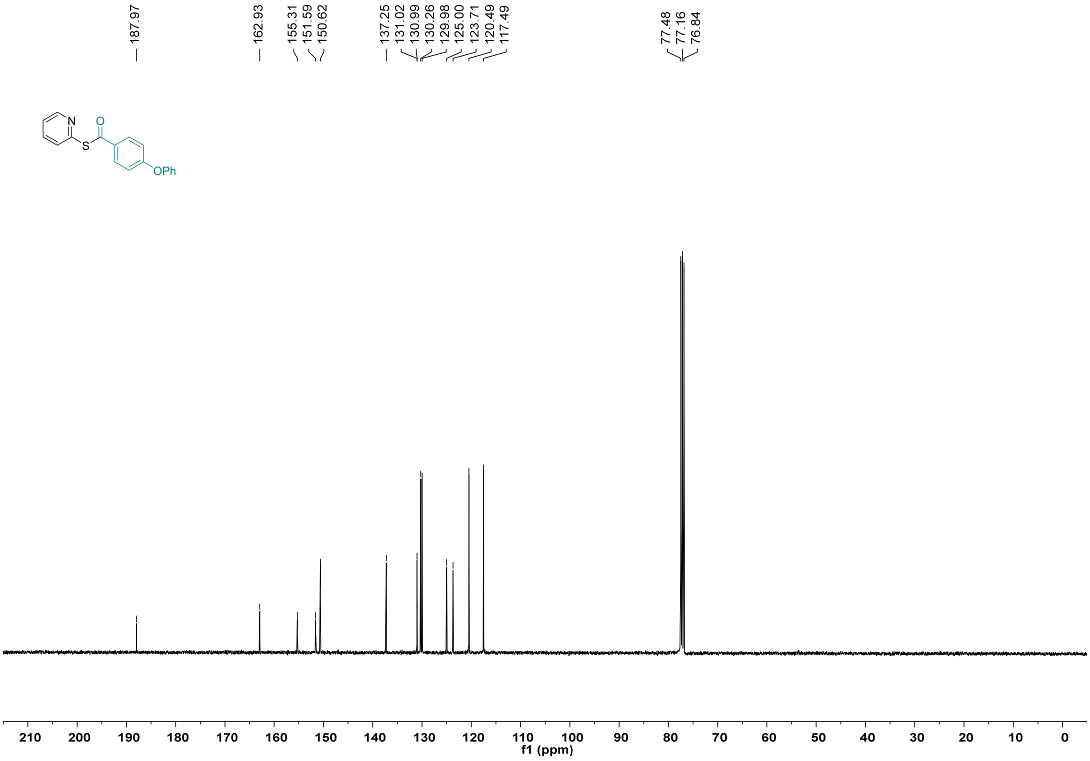

^{13}C NMR (100 MHz, $CDCl_3$) of compound **S10**

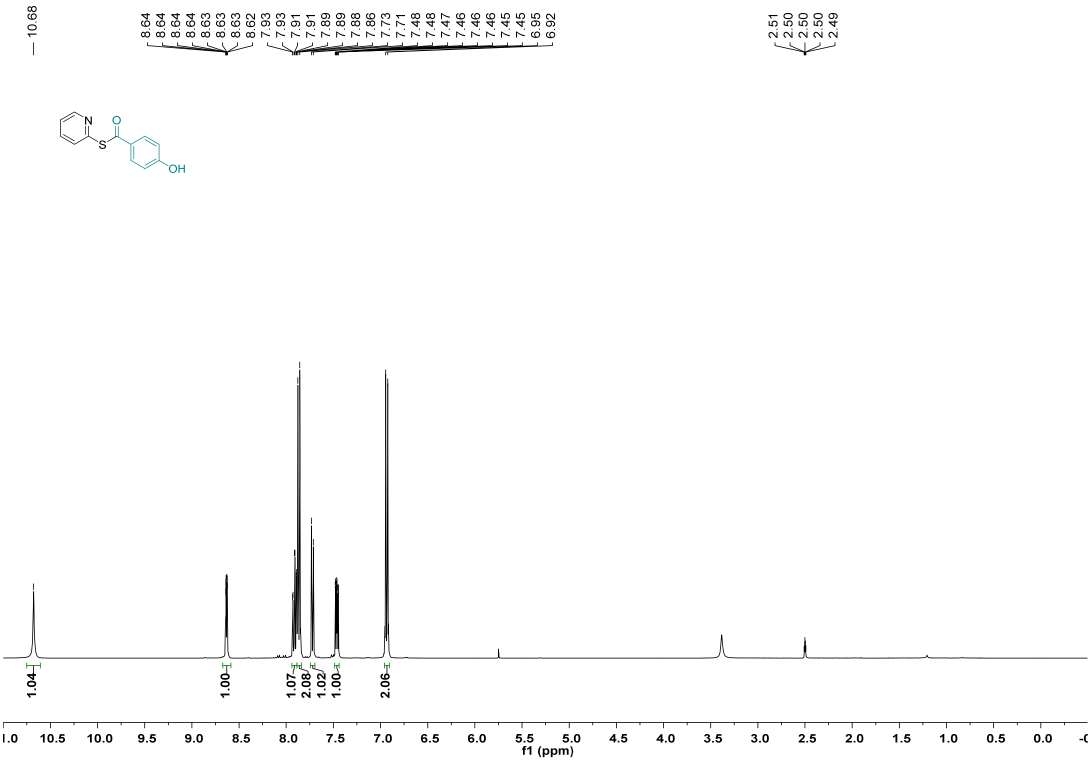

^{1}H NMR (400 MHz, DMSO-d_6) of compound **S11**

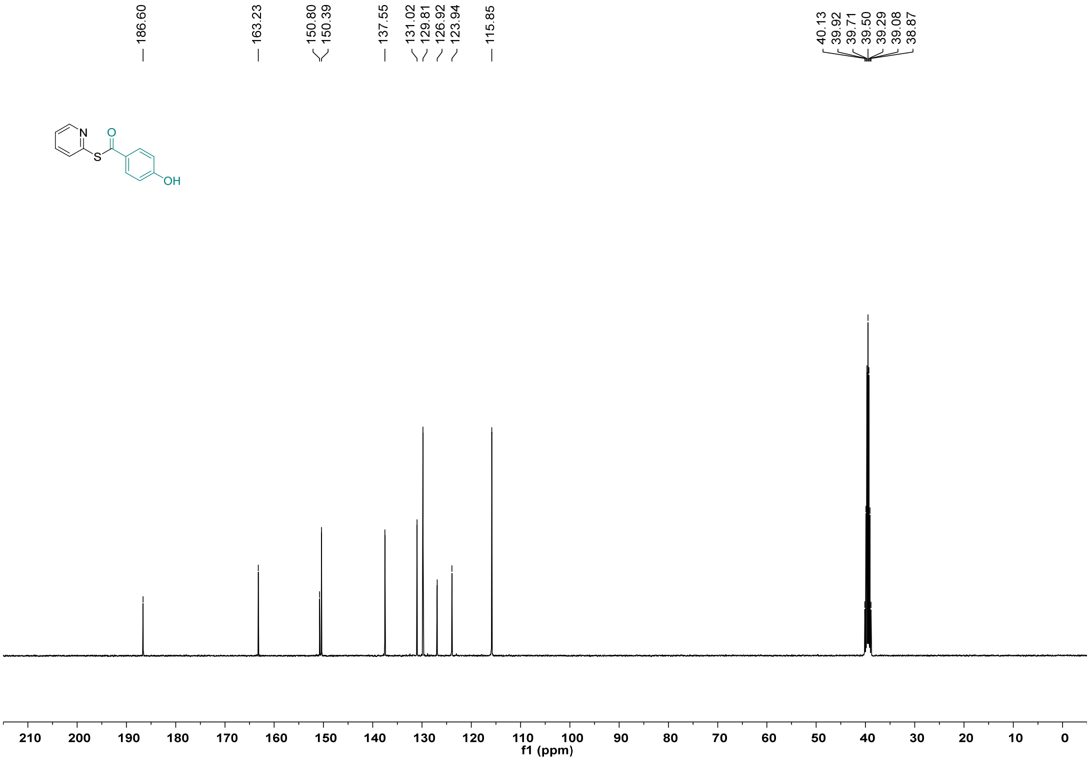

^{13}C NMR (100 MHz, DMSO-d_6) of compound **S11**

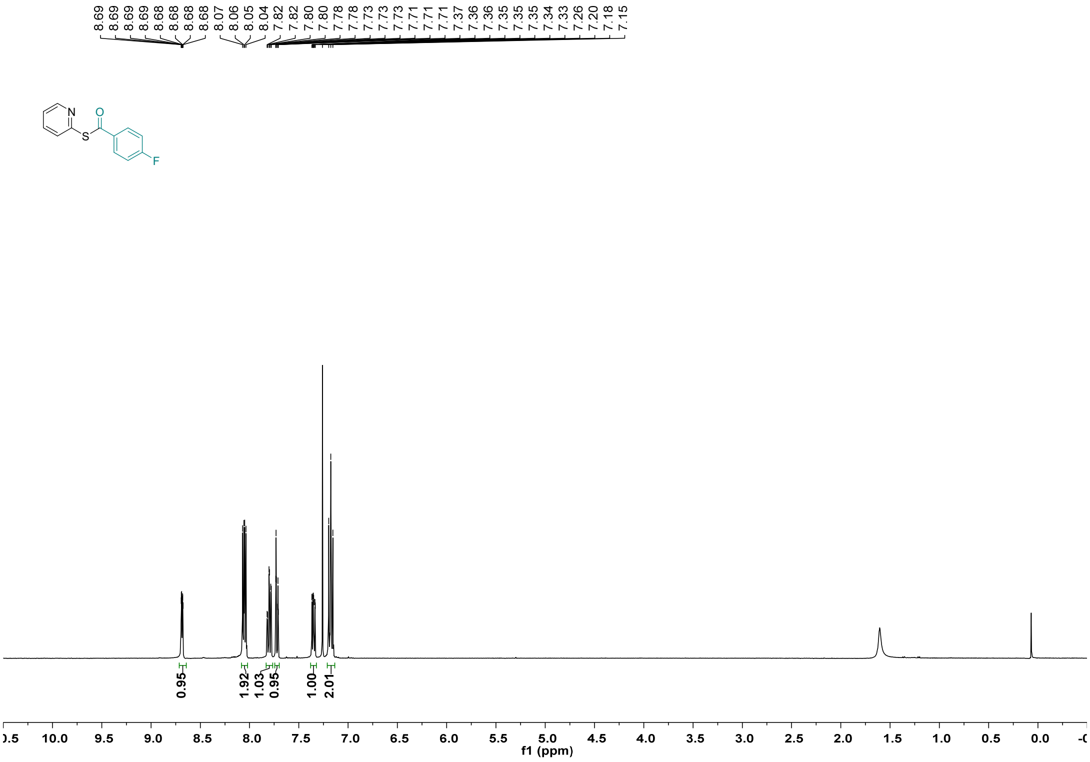

^{1}H NMR (400 MHz, $CDCl_3$) of compound **S12**

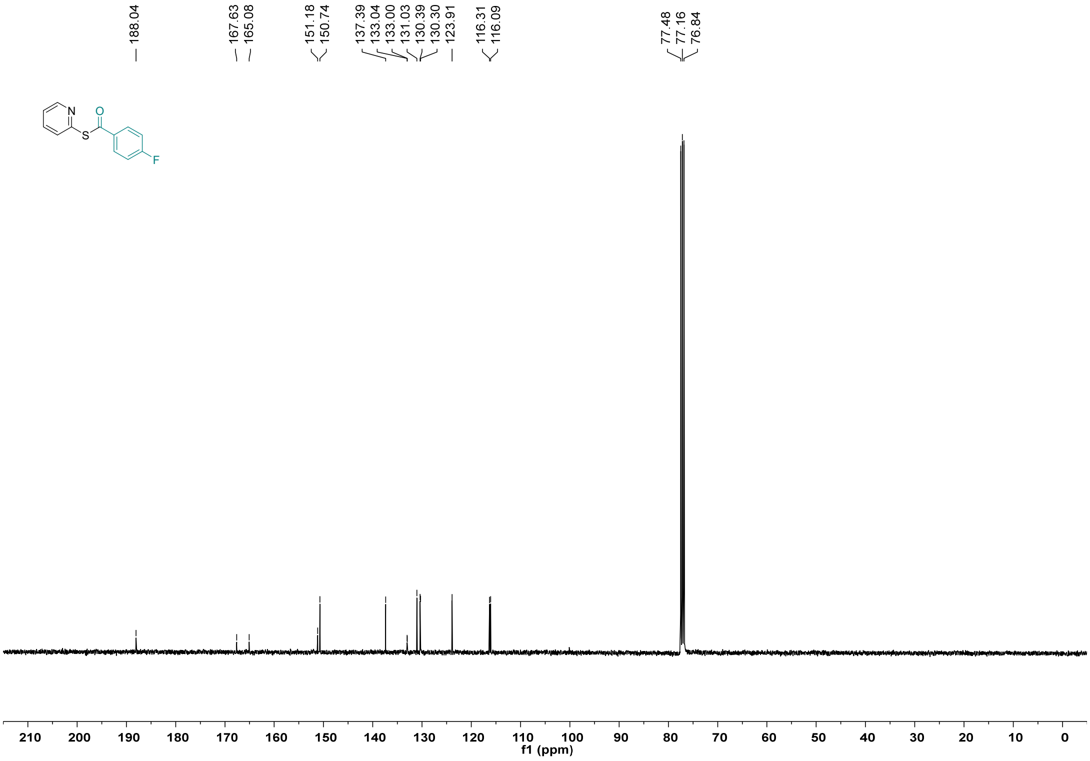

^{13}C NMR (100 MHz, $CDCl_3$) of compound **S12**

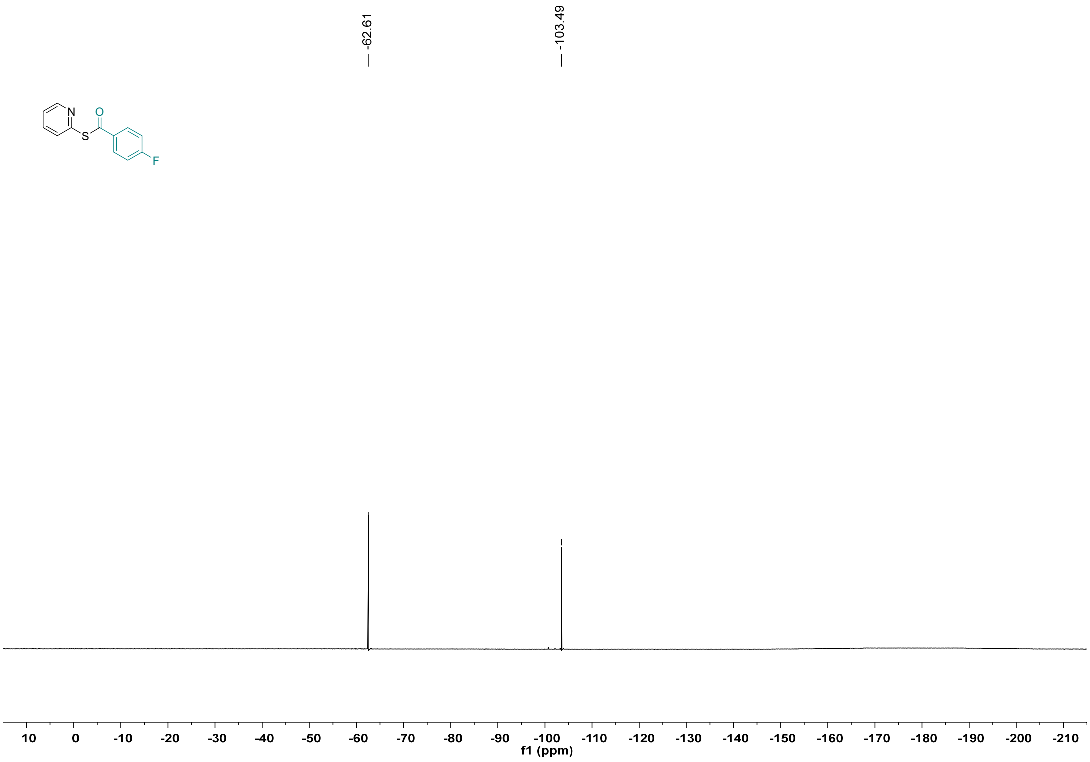

^{19}F NMR (377 MHz, $CDCl_3$) of compound **S12**

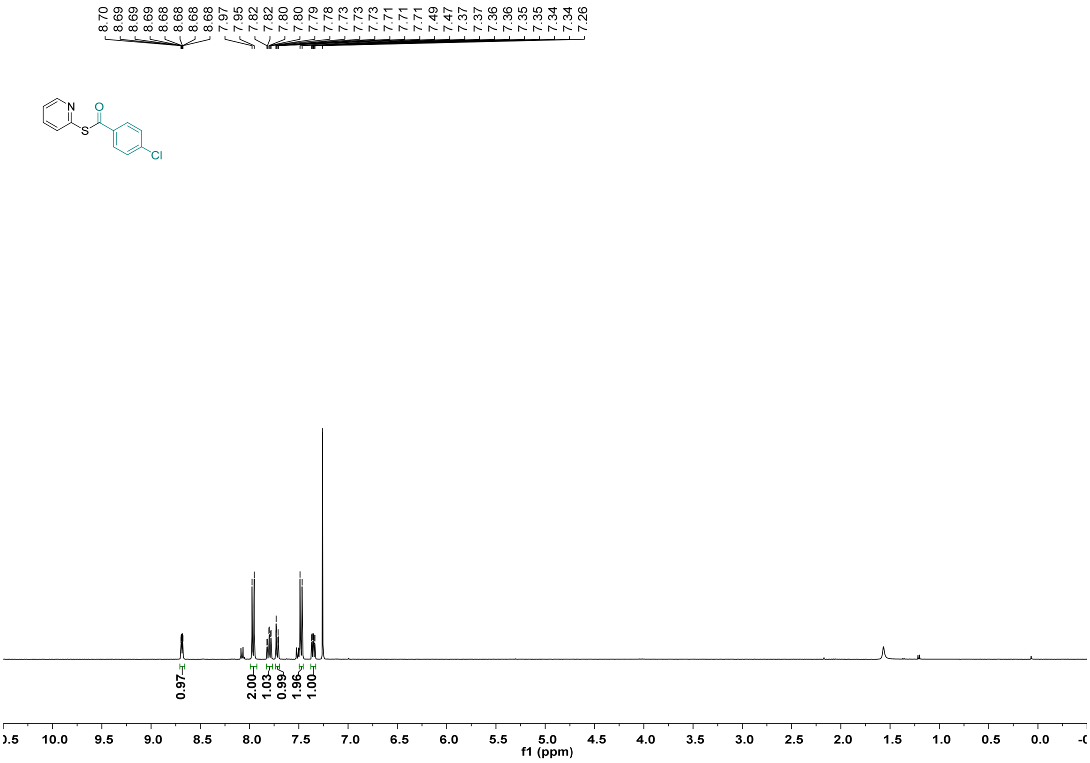

^{1}H NMR (400 MHz, $CDCl_3$) of compound **S13**

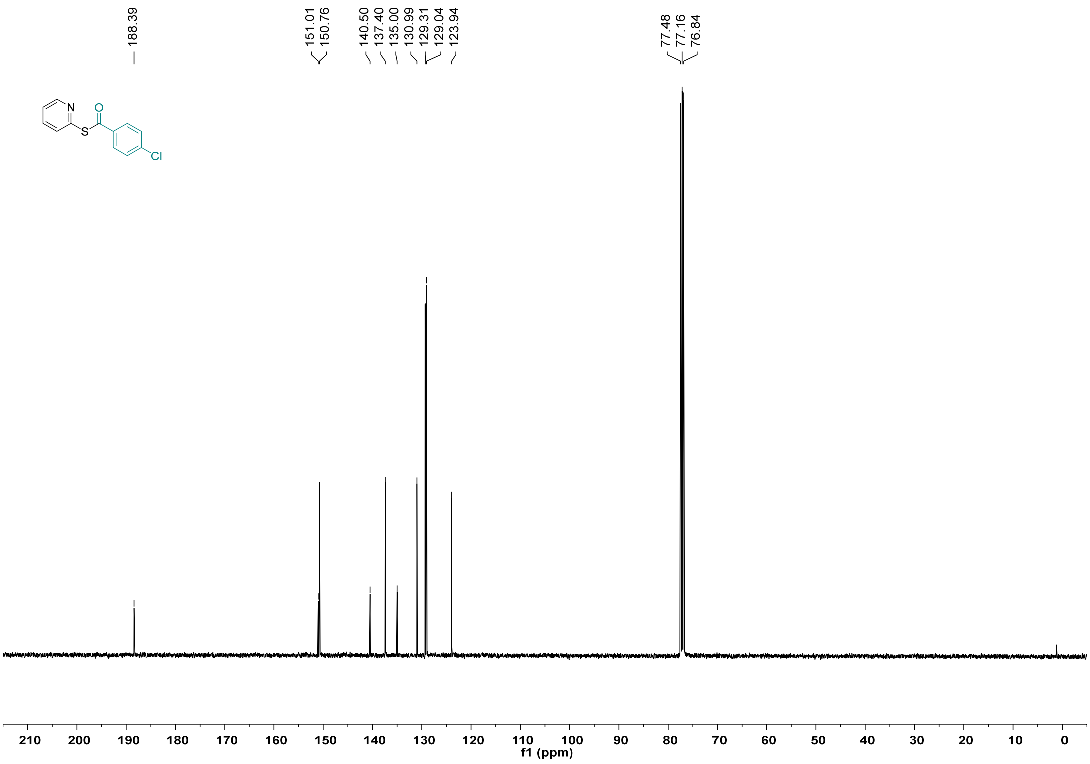

^{13}C NMR (100 MHz, $CDCl_3$) of compound **S13**

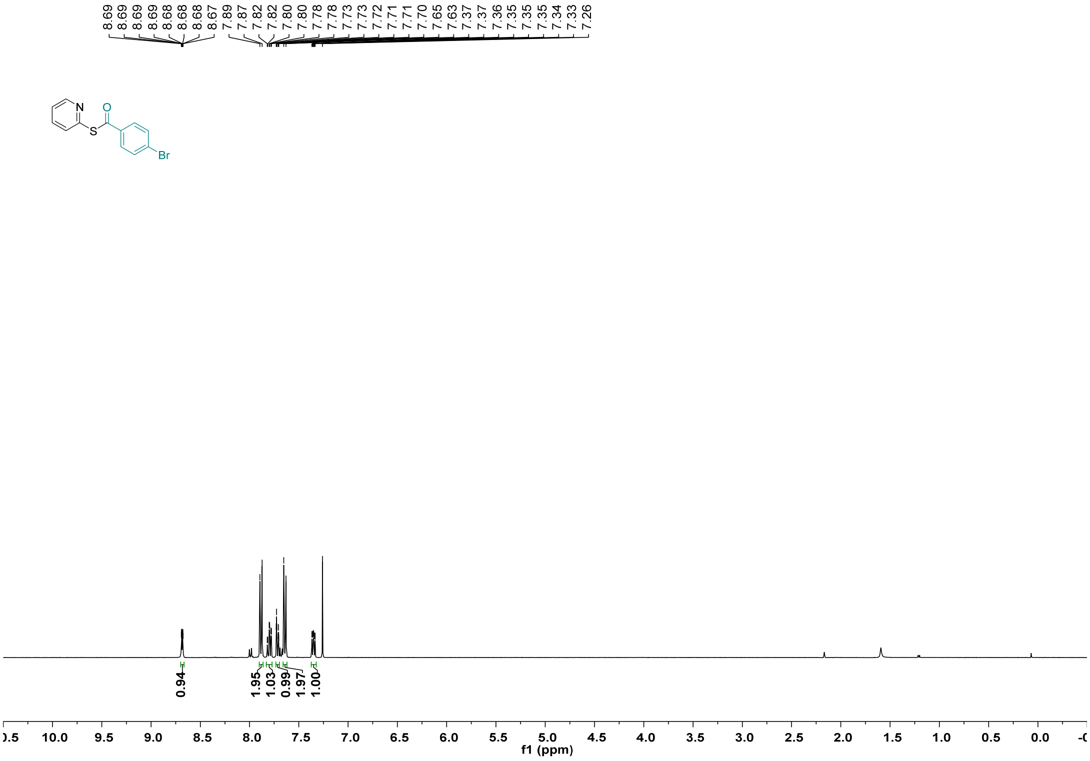

^{1}H NMR (400 MHz, $CDCl_3$) of compound **S14**

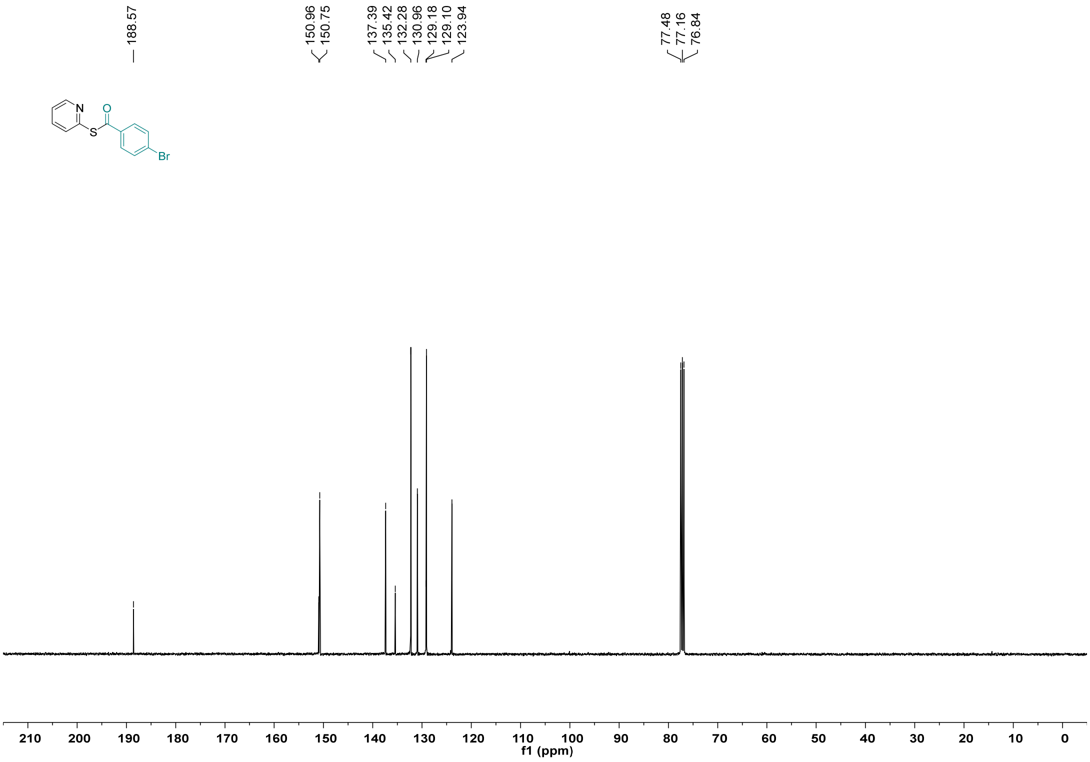

^{13}C NMR (100 MHz, $CDCl_3$) of compound **S14**

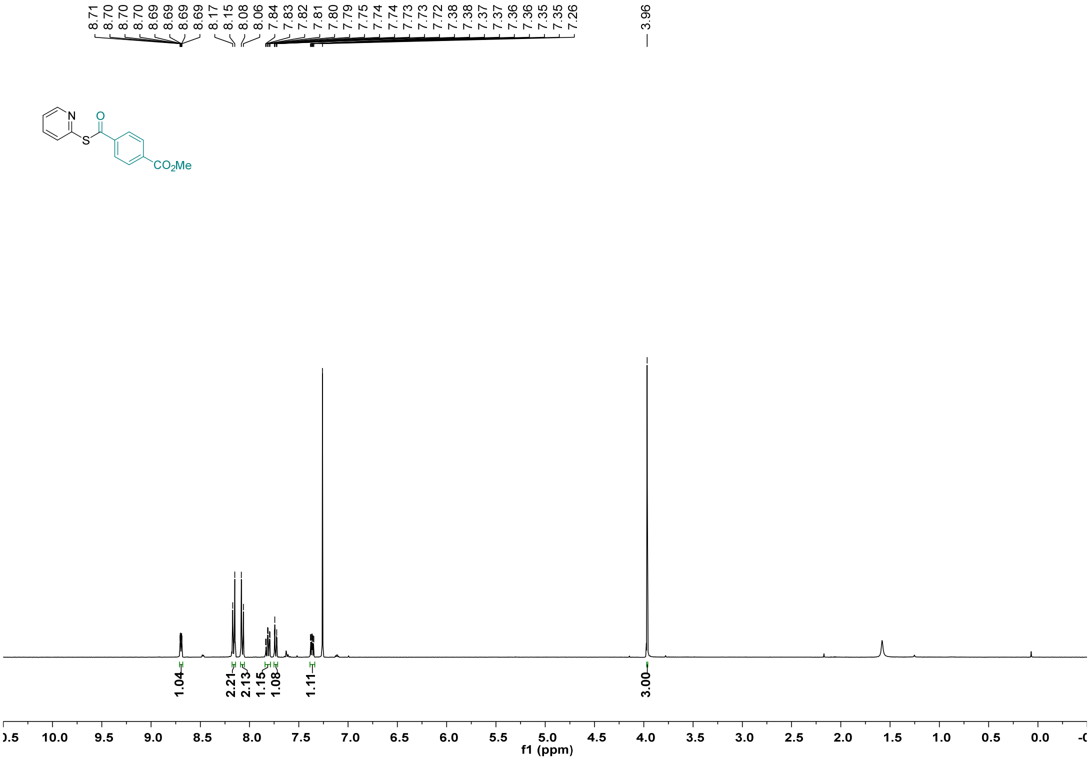

^{1}H NMR (400 MHz, $CDCl_3$) of compound **S15**

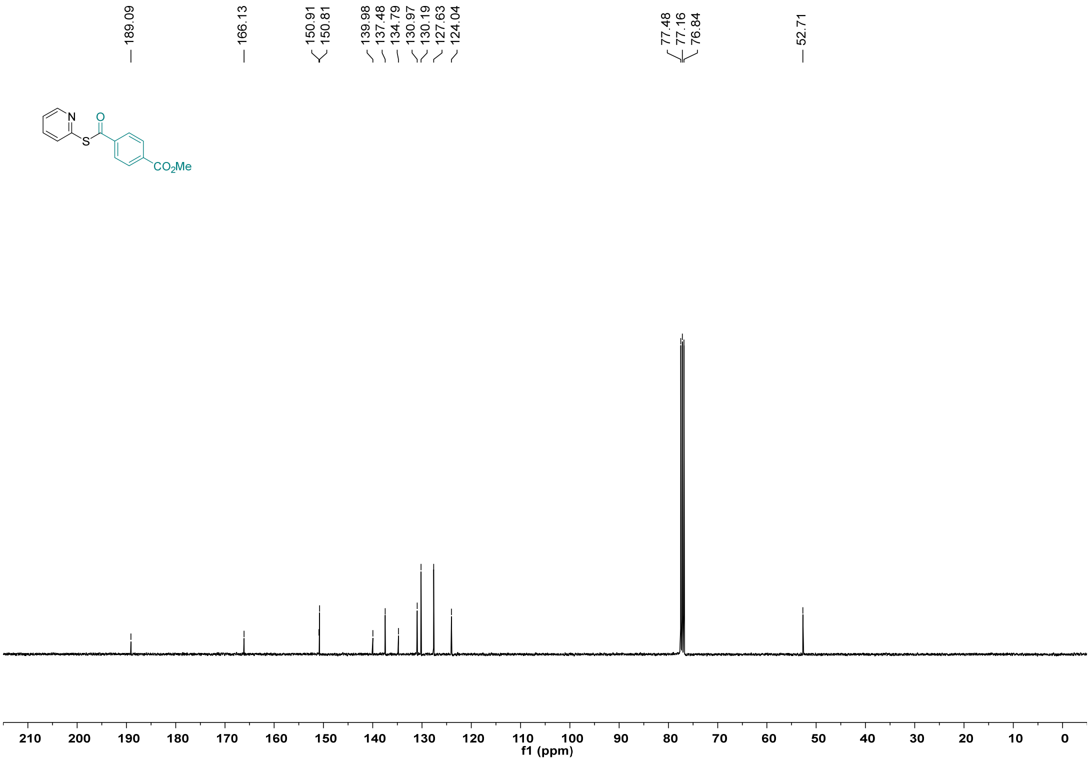

^{13}C NMR (100 MHz, $CDCl_3$) of compound **S15**

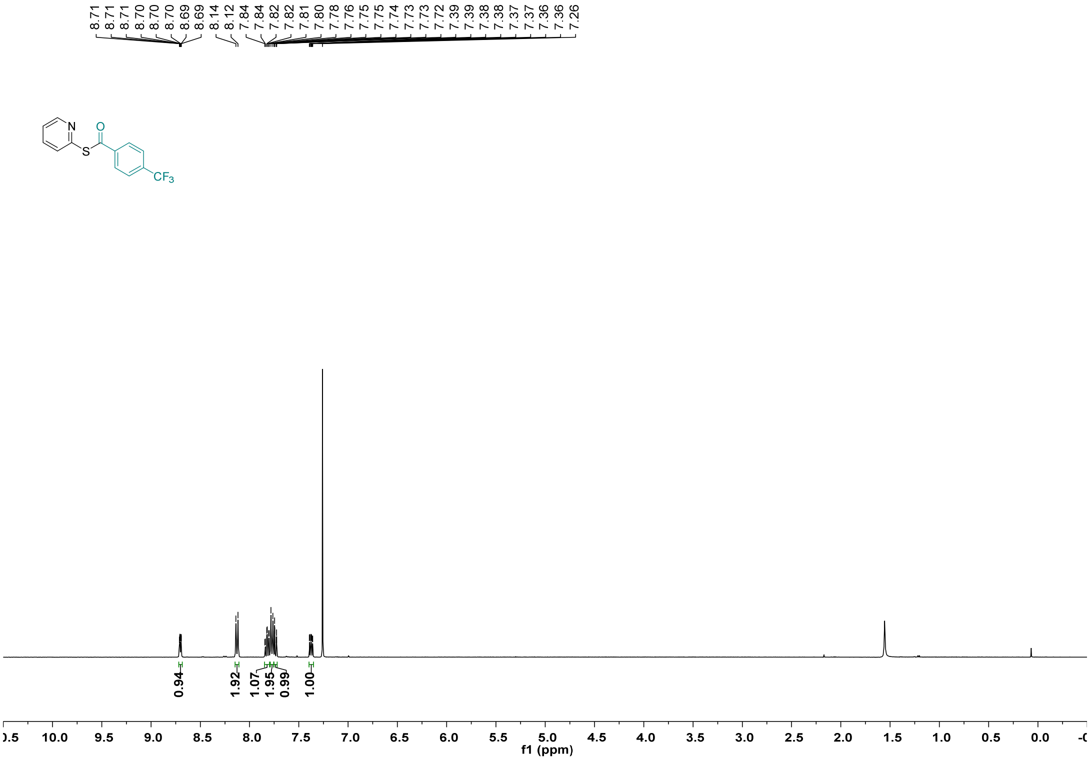

^{1}H NMR (400 MHz, $CDCl_3$) of compound **S16**

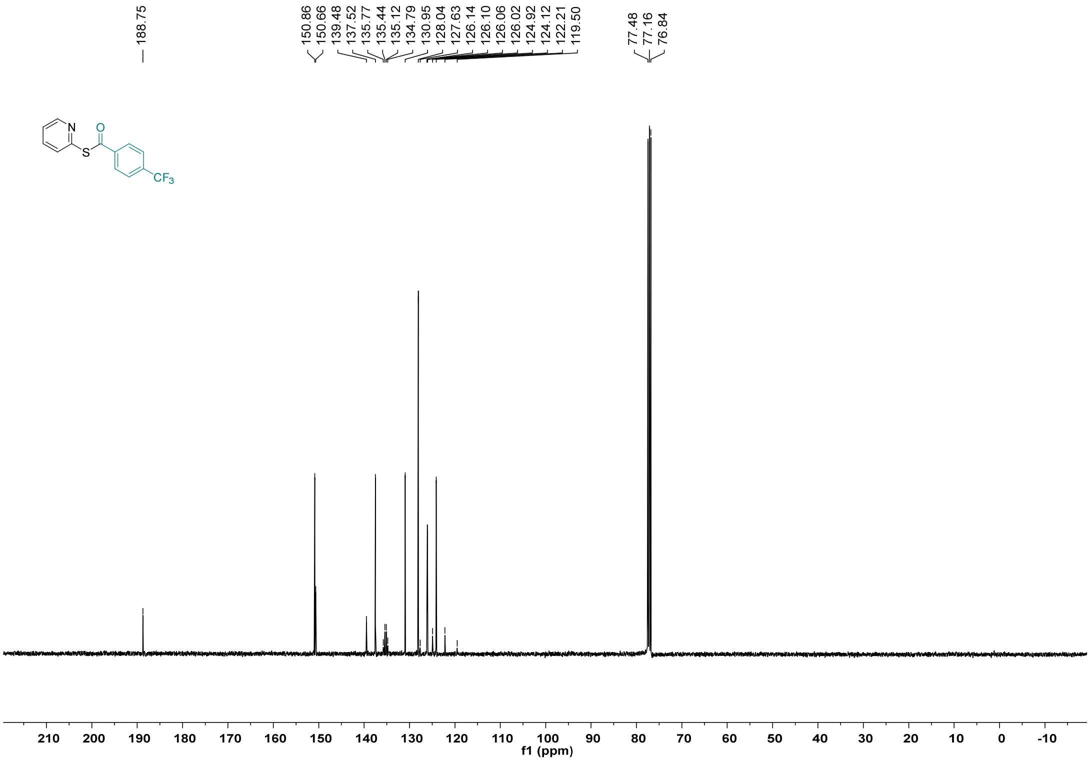

^{13}C NMR (100 MHz, $CDCl_3$) of compound **S16**

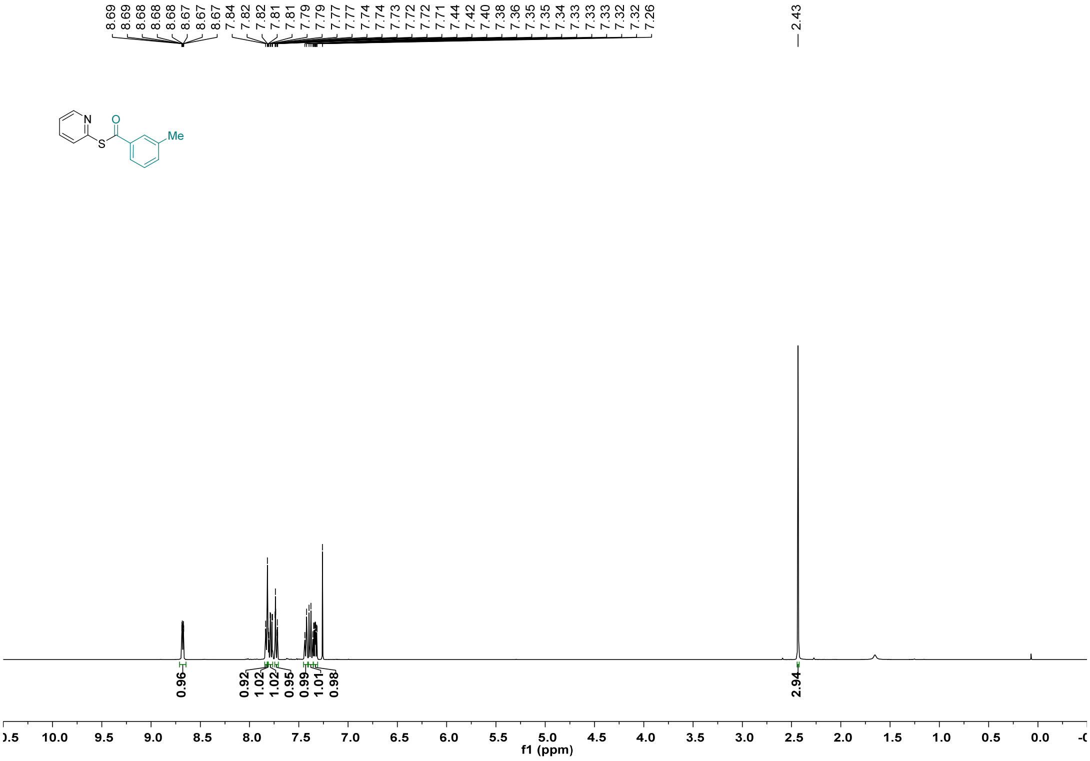

^{1}H NMR (400 MHz, $CDCl_3$) of compound **S17**

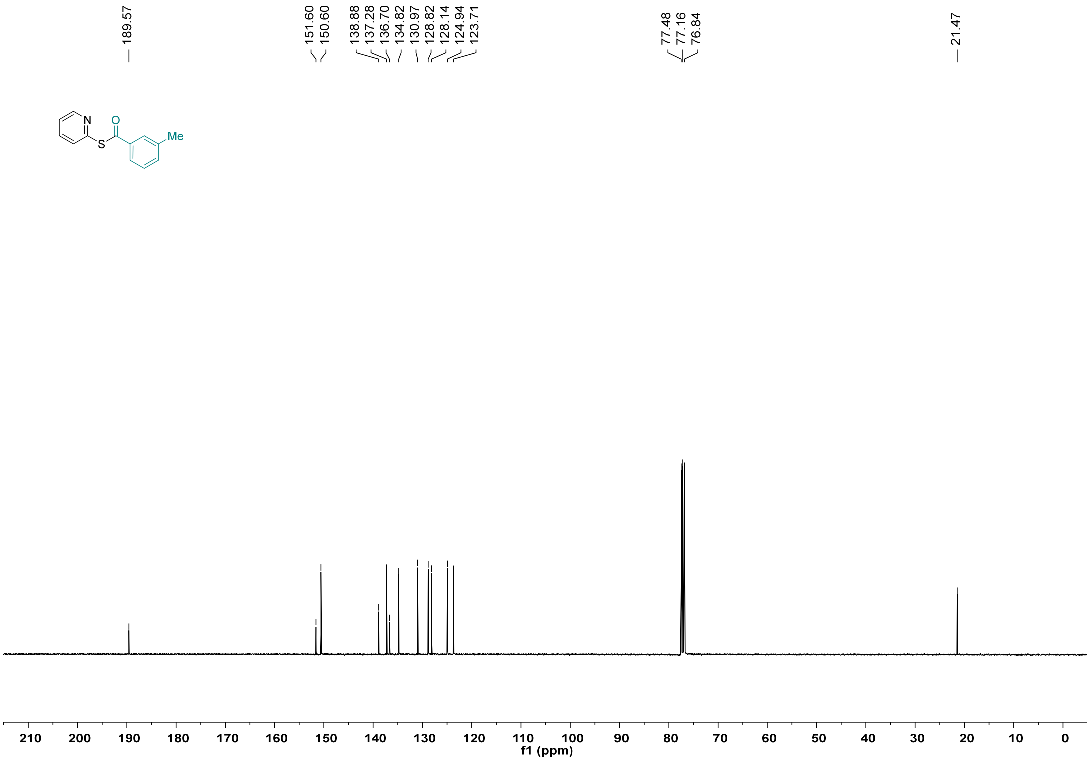

^{13}C NMR (100 MHz, $CDCl_3$) of compound **S17**

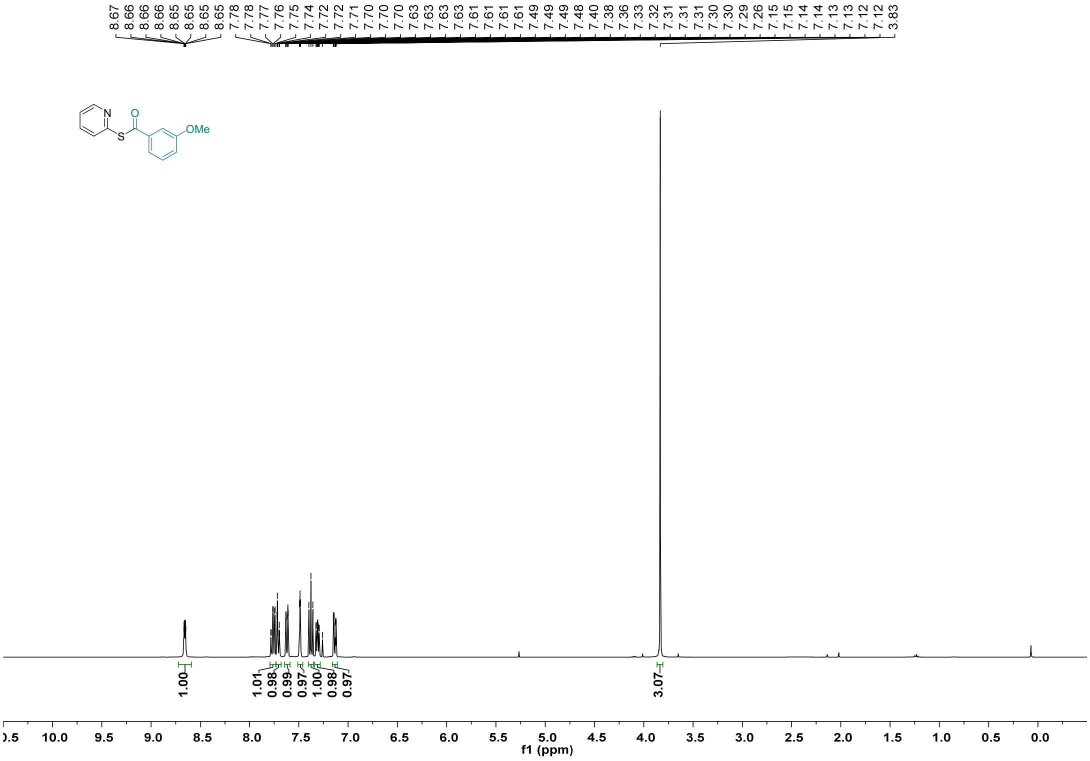

^{1}H NMR (400 MHz, $CDCl_3$) of compound **S18**

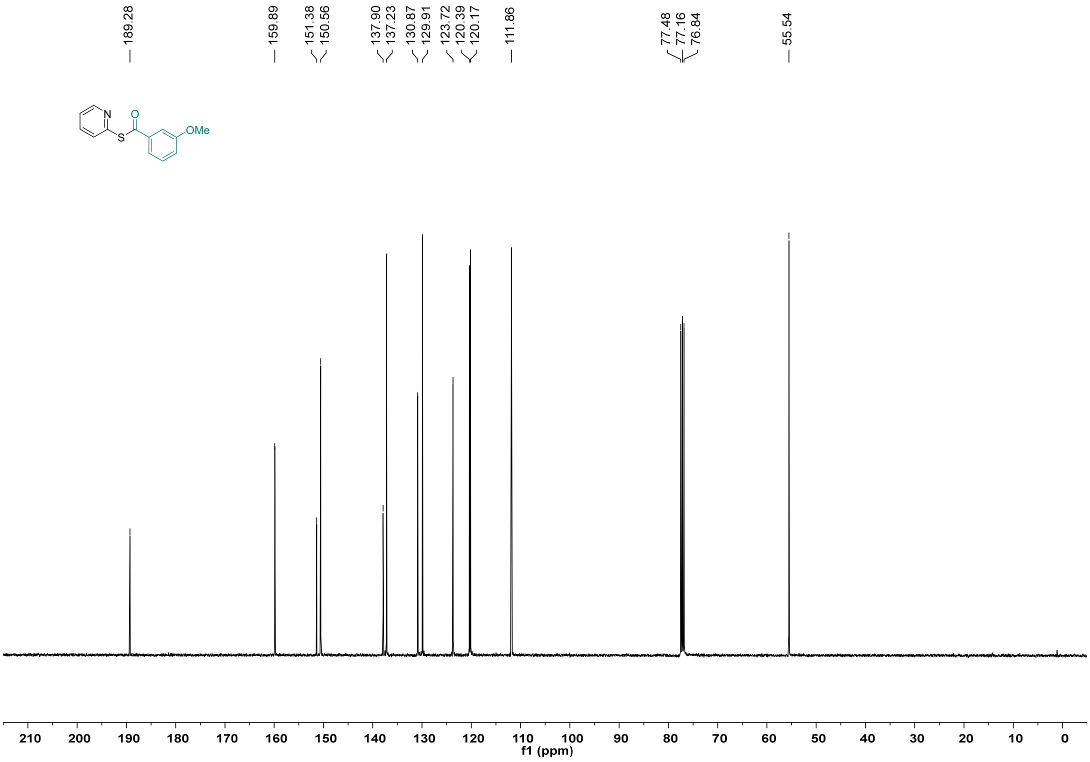

^{13}C NMR (100 MHz, CDCl$_3$) of compound **S18**

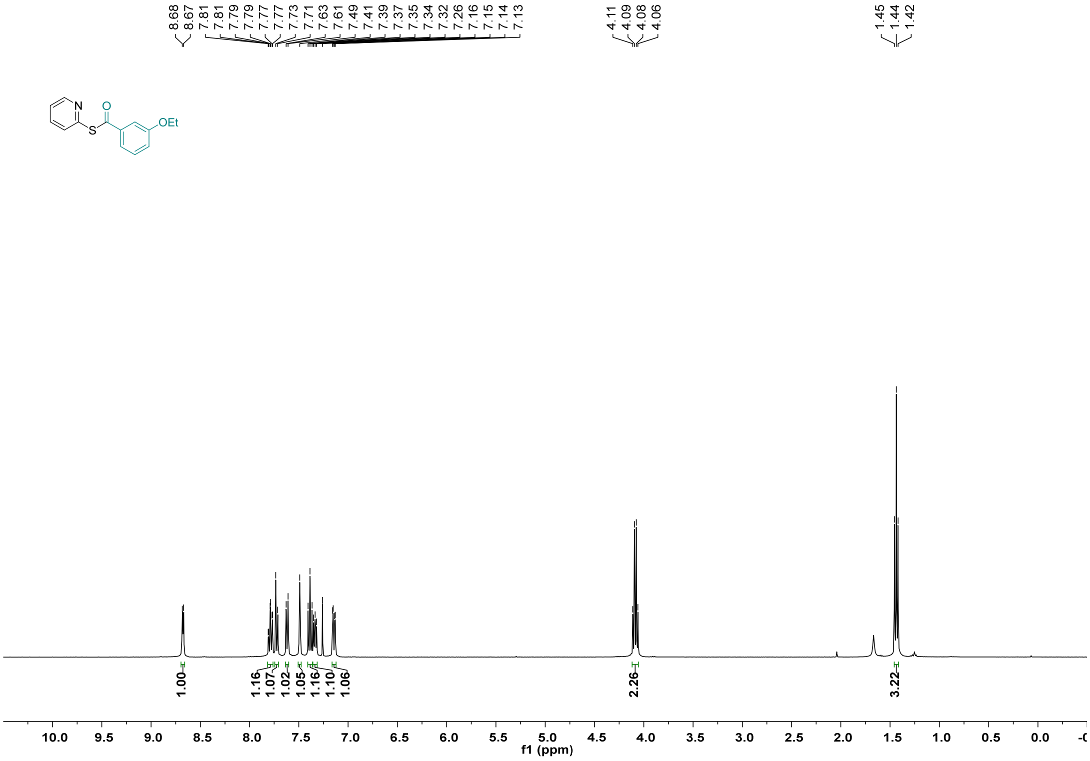

^{1}H NMR (400 MHz, CDCl$_3$) of compound **S19**

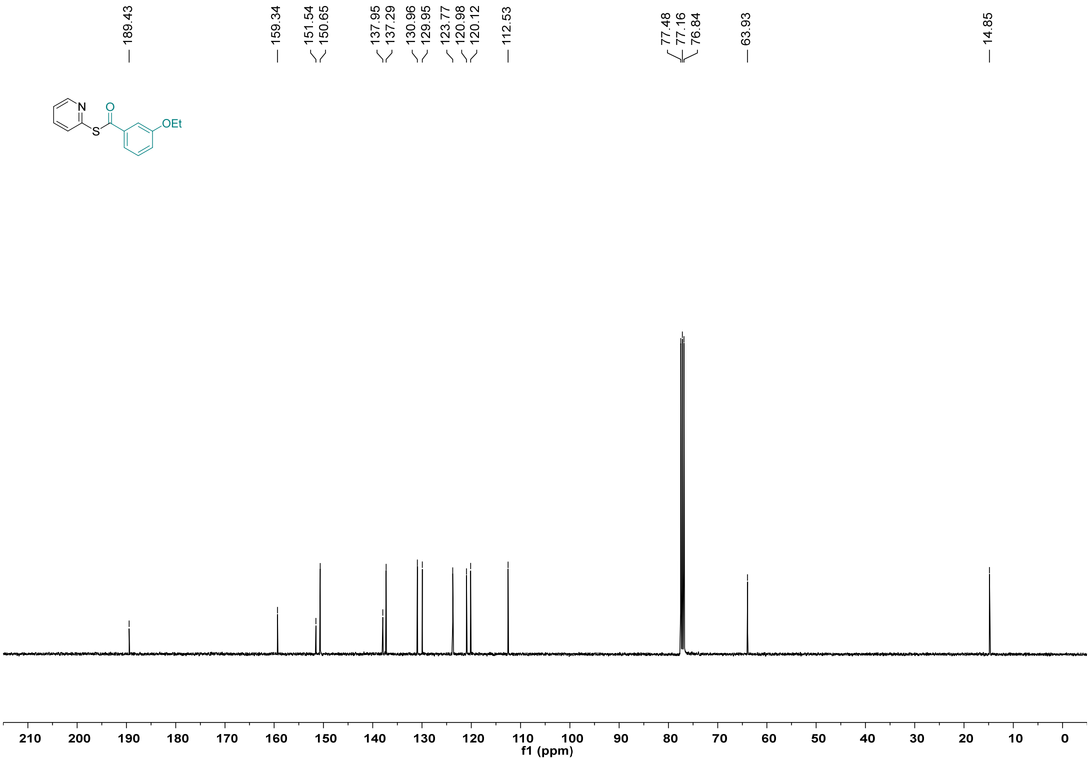

^{13}C NMR (100 MHz, $CDCl_3$) of compound **S19**

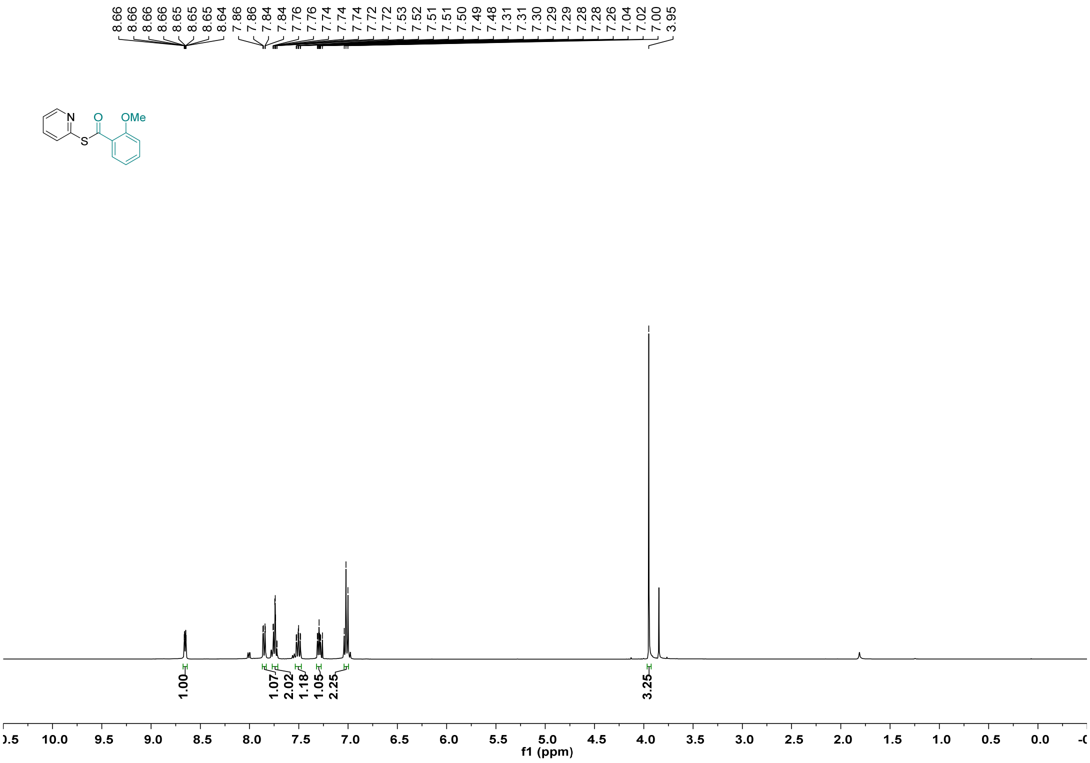

^{1}H NMR (400 MHz, $CDCl_3$) of compound **S20**

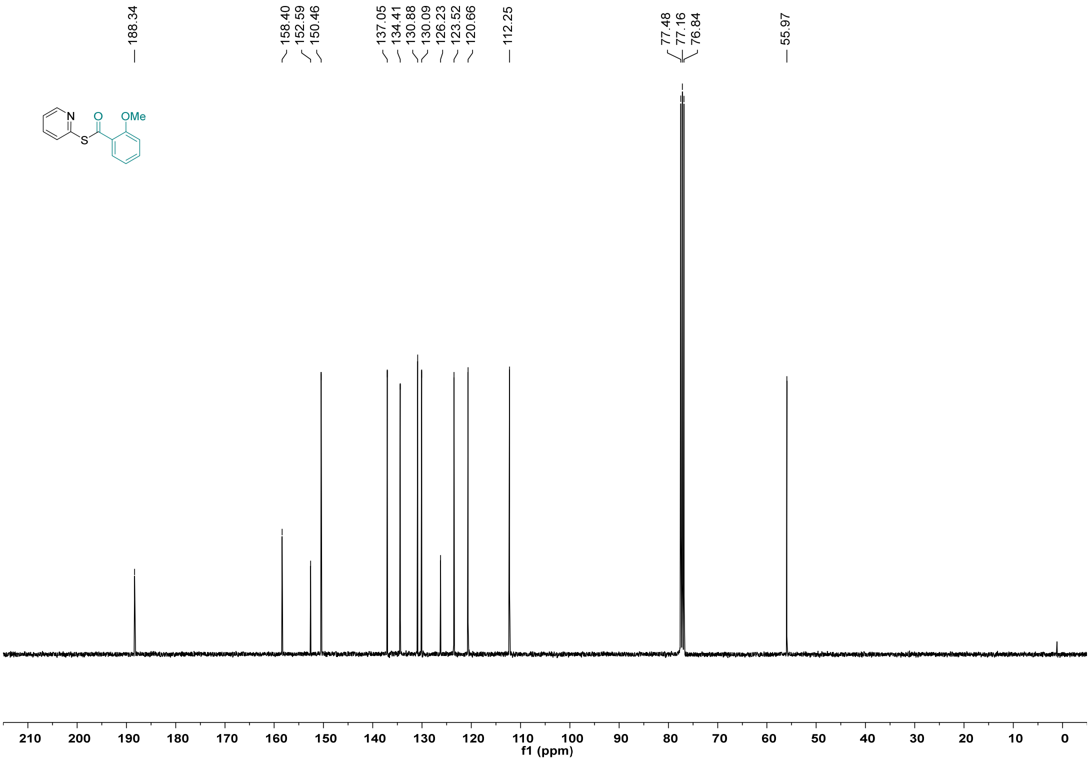

^{13}C NMR (100 MHz, $CDCl_3$) of compound **S20**

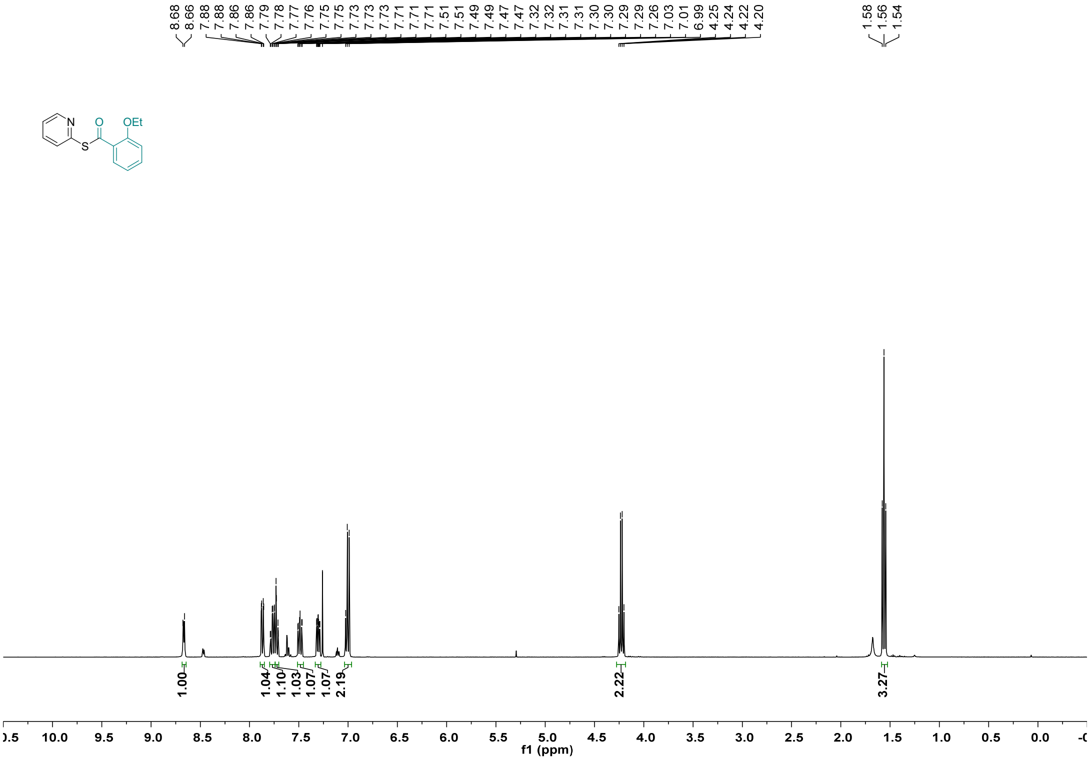

^{1}H NMR (400 MHz, $CDCl_3$) of compound **S21**

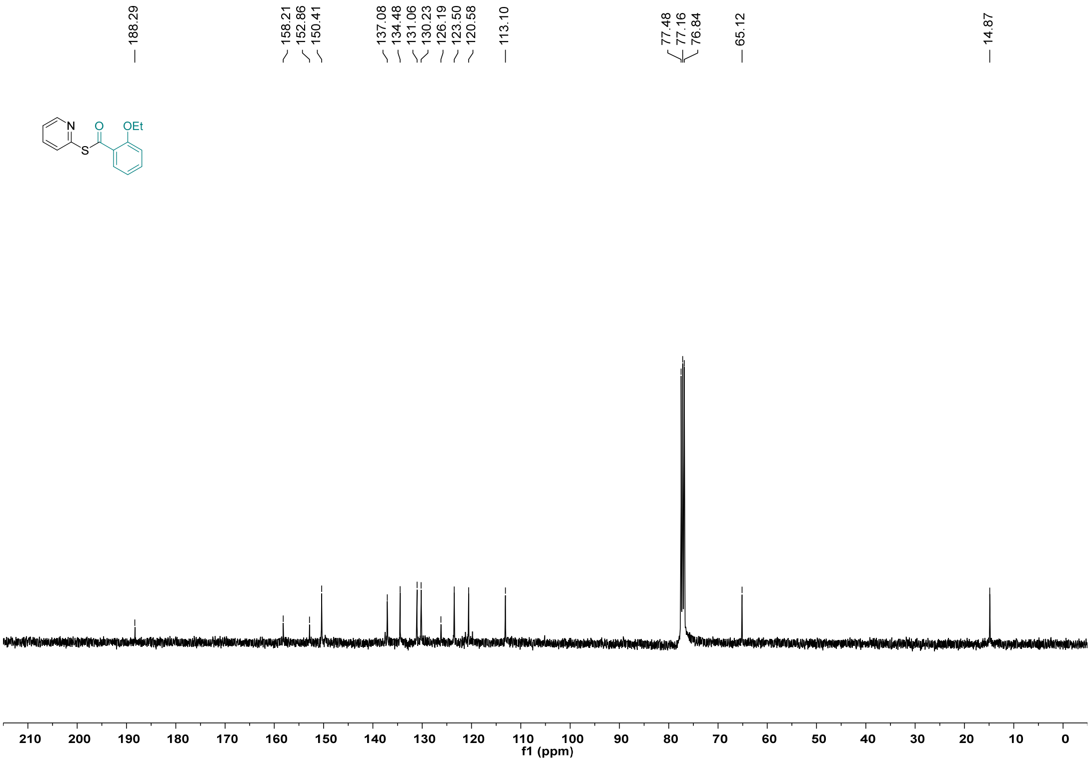

^{13}C NMR (100 MHz, $CDCl_3$) of compound **S21**

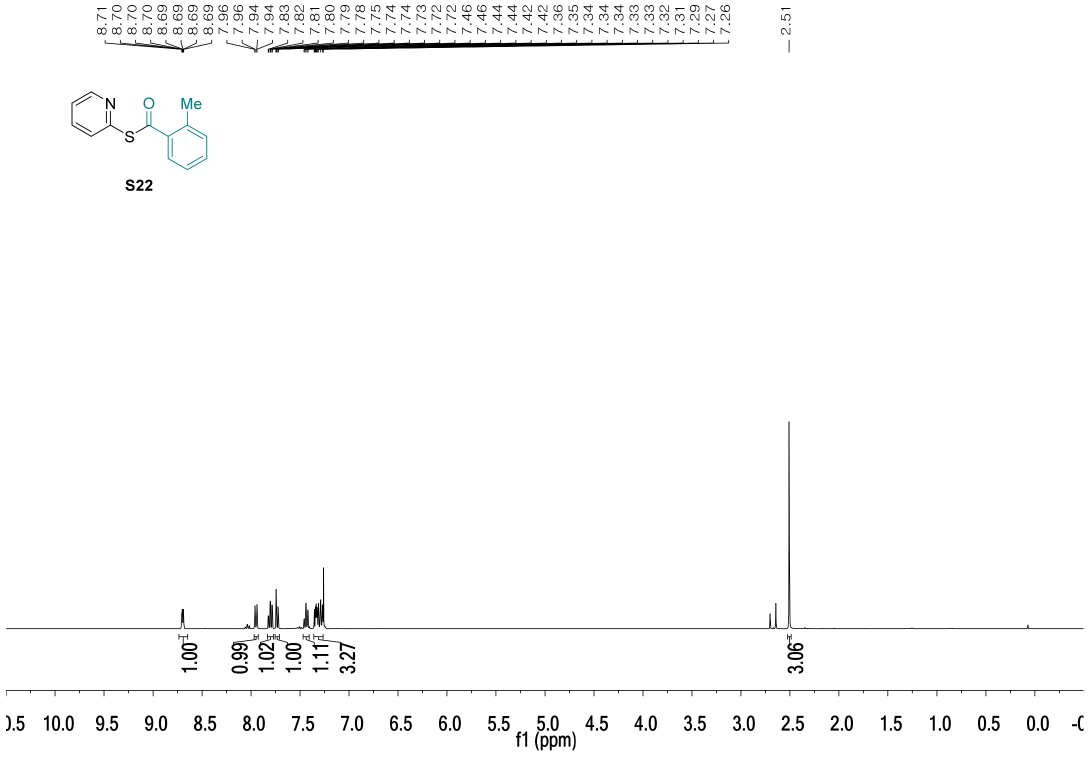

^{1}H NMR (400 MHz, $CDCl_3$) of compound **S22**

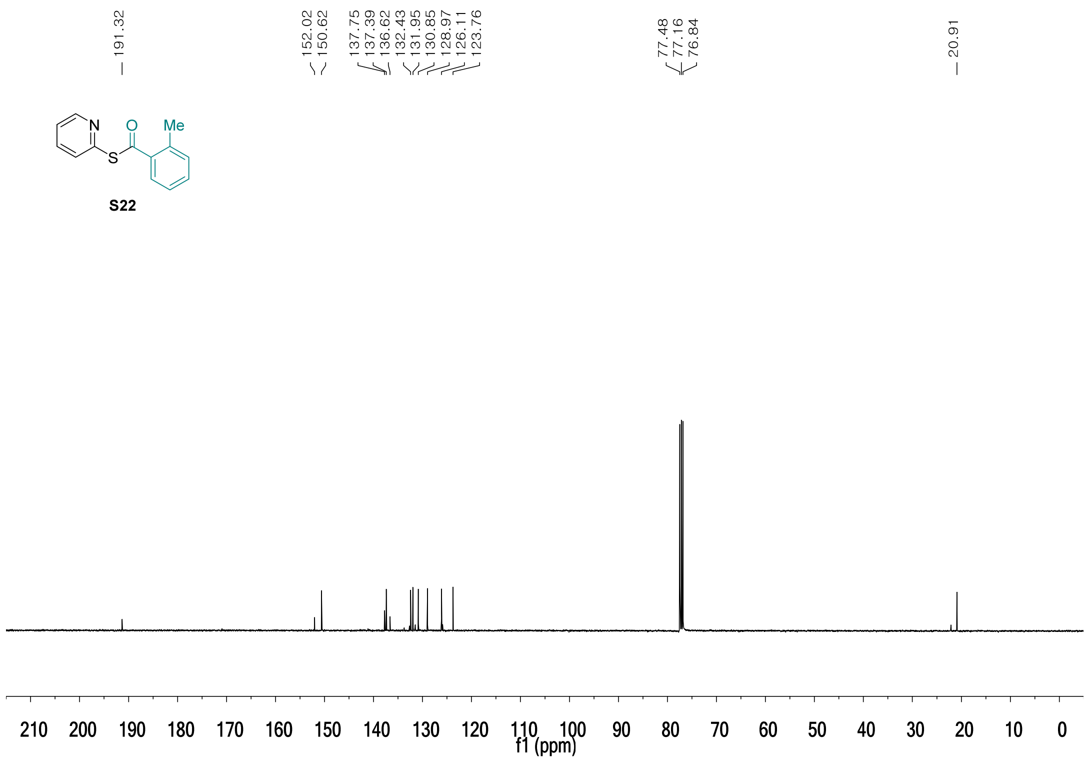

^{13}C NMR (100 MHz, $CDCl_3$) of compound **S22**

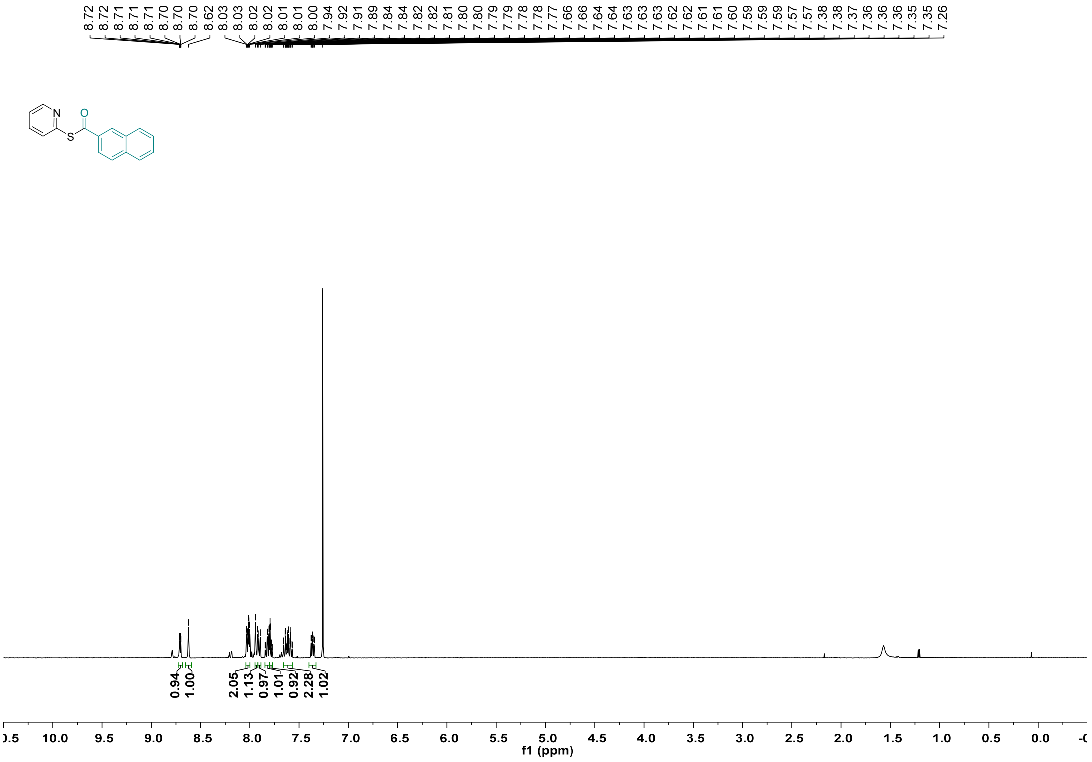

^{1}H NMR (400 MHz, $CDCl_3$) of compound **S23**

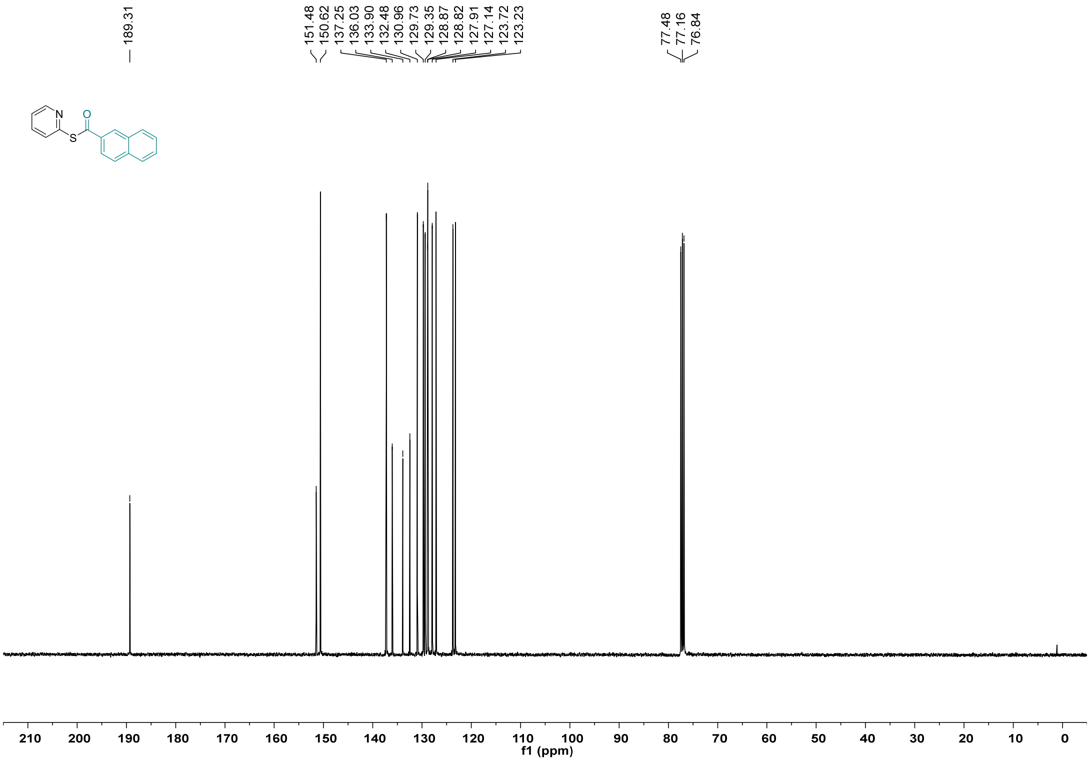

^{13}C NMR (100 MHz, CDCl$_3$) of compound **S23**

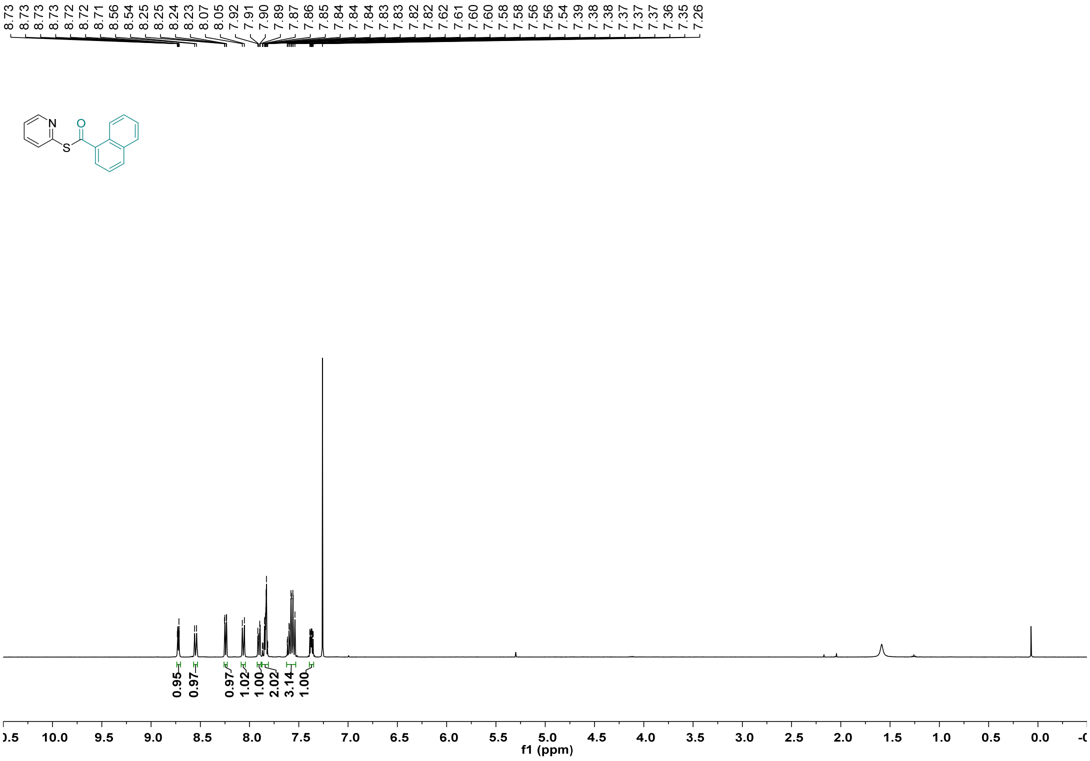

^{1}H NMR (400 MHz, CDCl$_3$) of compound **S24**

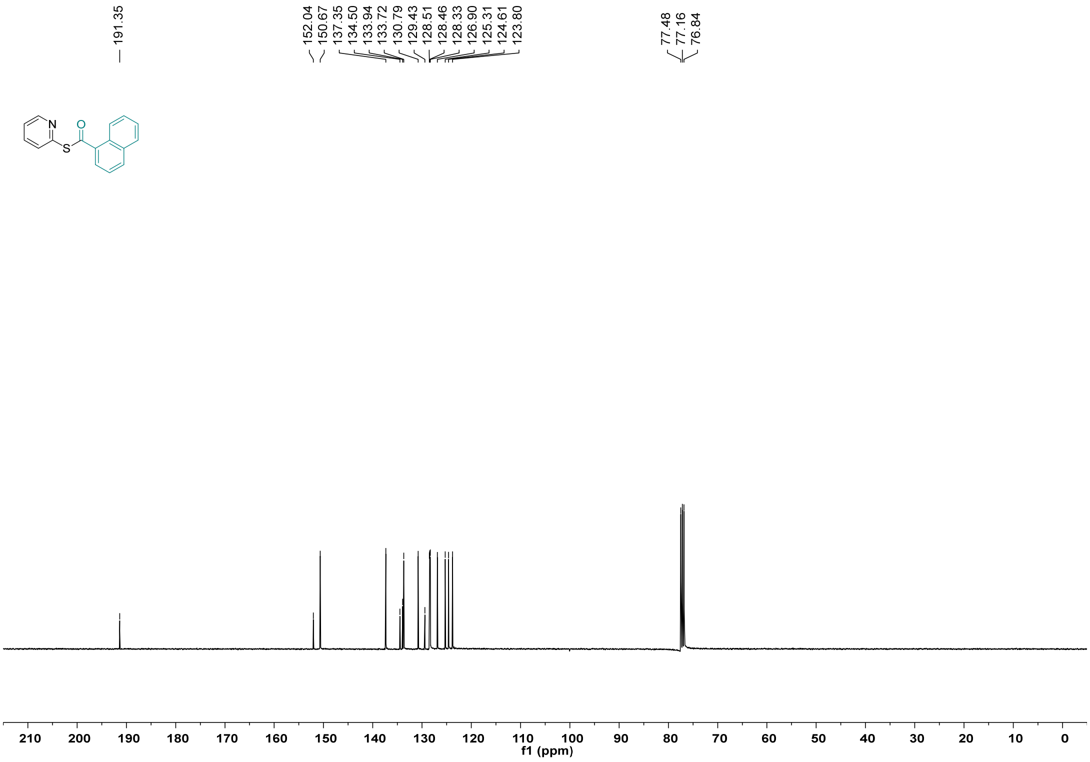

^{13}C NMR (100 MHz, $CDCl_3$) of compound **S24**

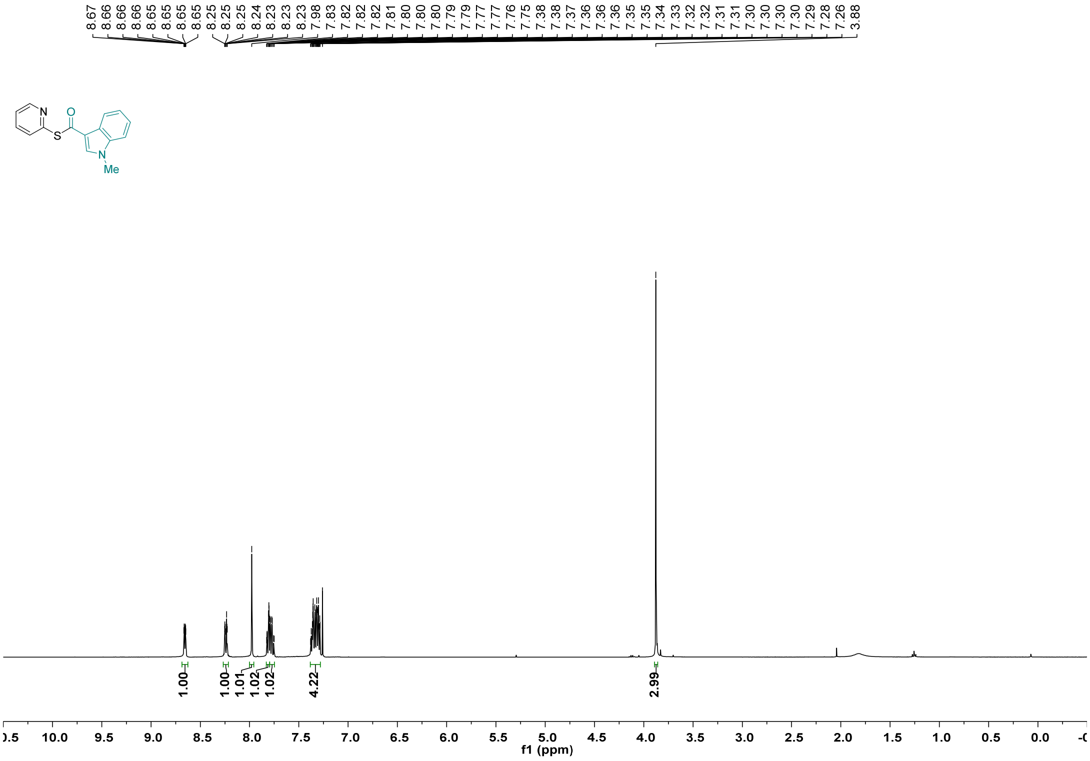

^{1}H NMR (400 MHz, $CDCl_3$) of compound **S25**

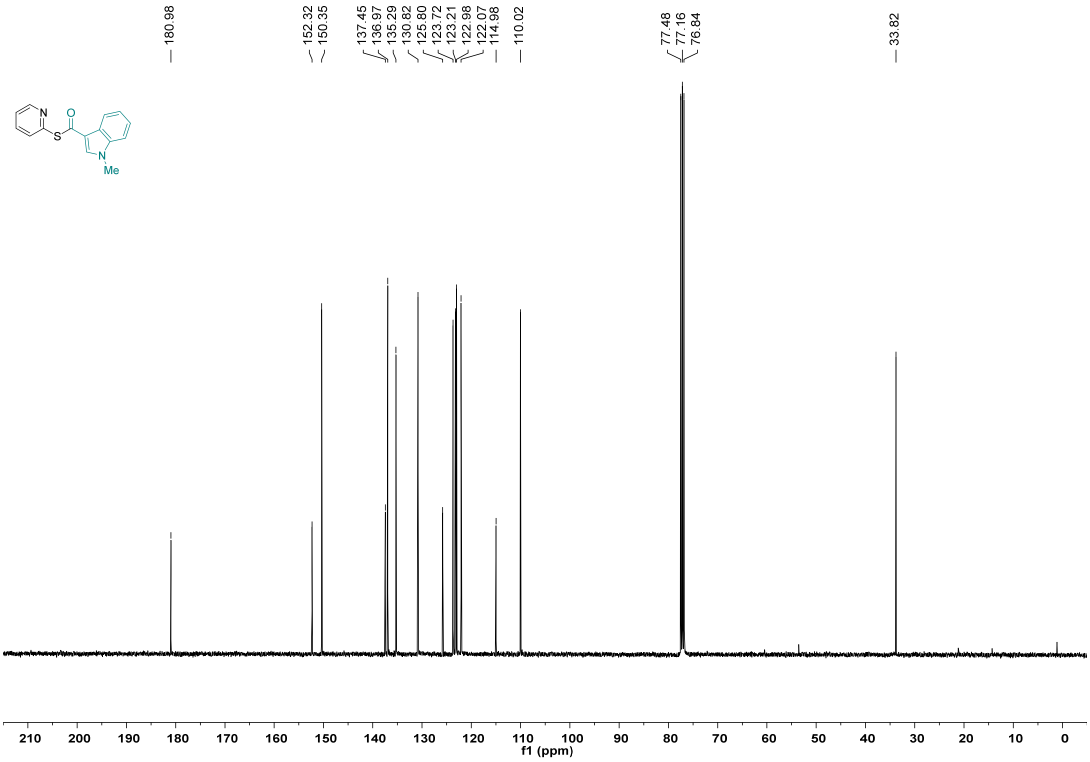

^{13}C NMR (100 MHz, $CDCl_3$) of compound **S25**

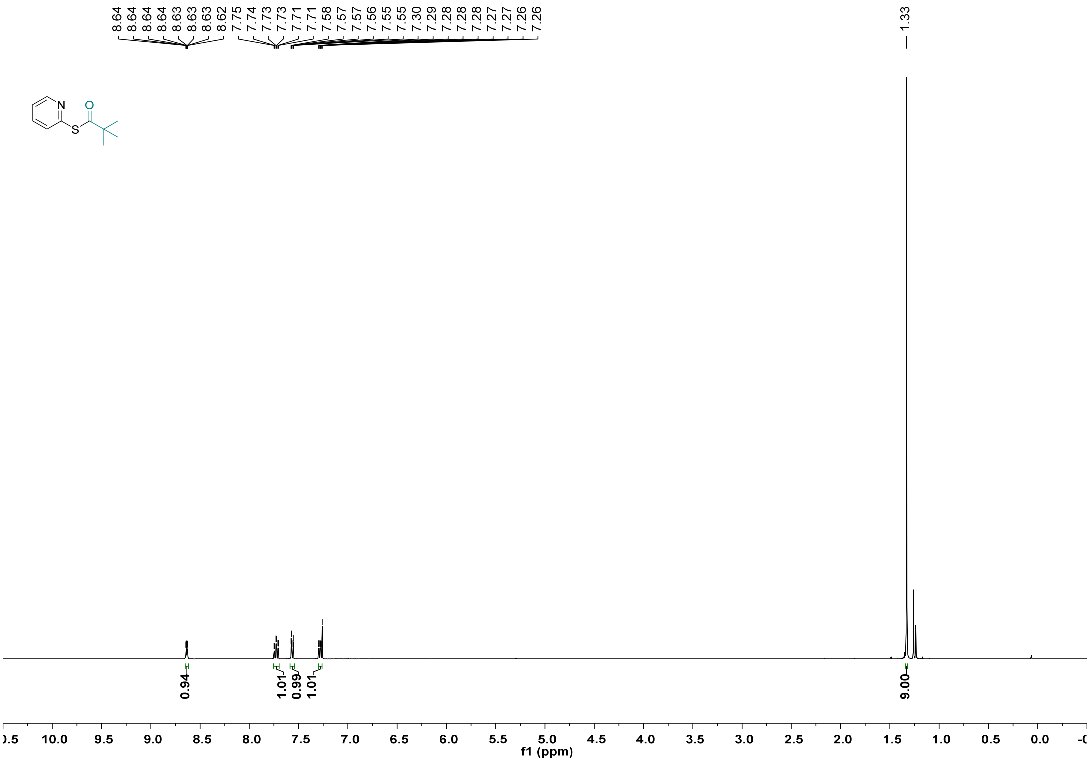

^{1}H NMR (400 MHz, $CDCl_3$) of compound **S26**

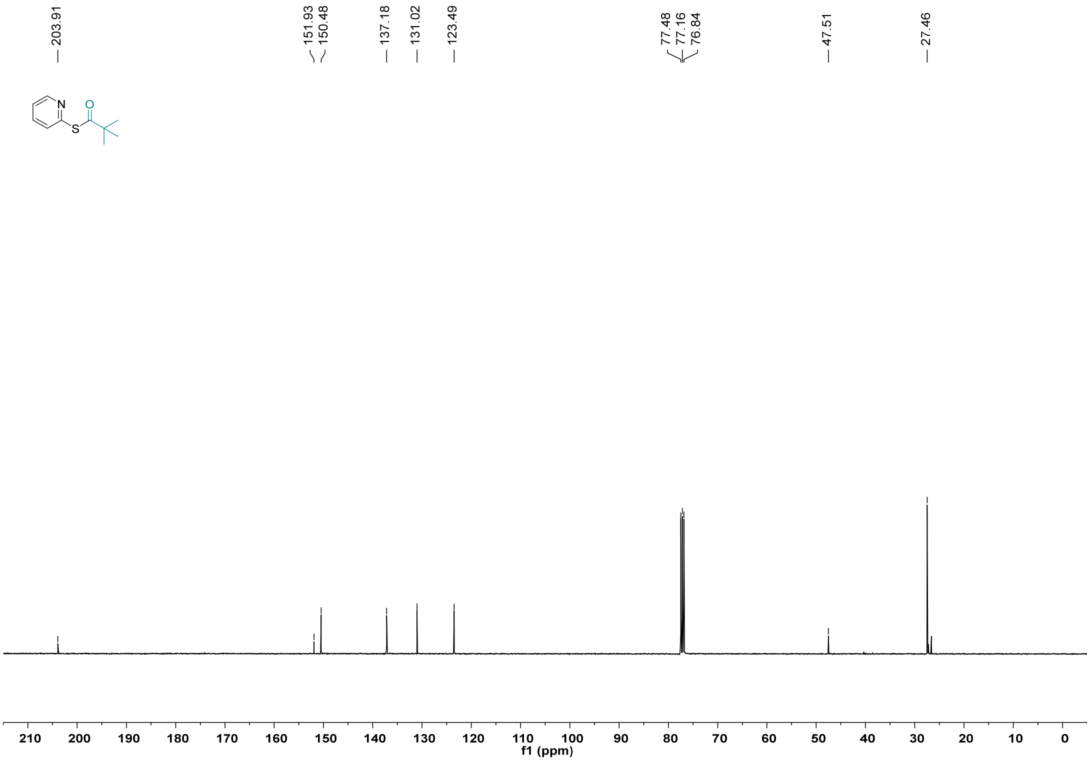

^{13}C NMR (100 MHz, $CDCl_3$) of compound **S26**

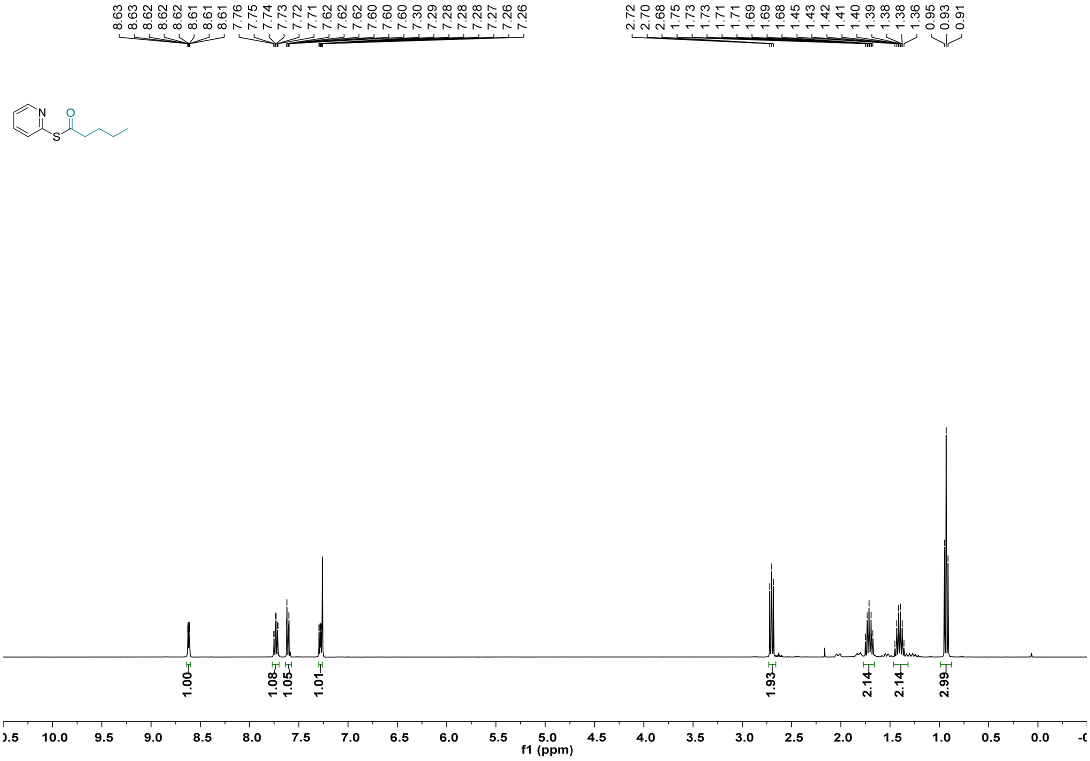

^{1}H NMR (400 MHz, $CDCl_3$) of compound **S27**

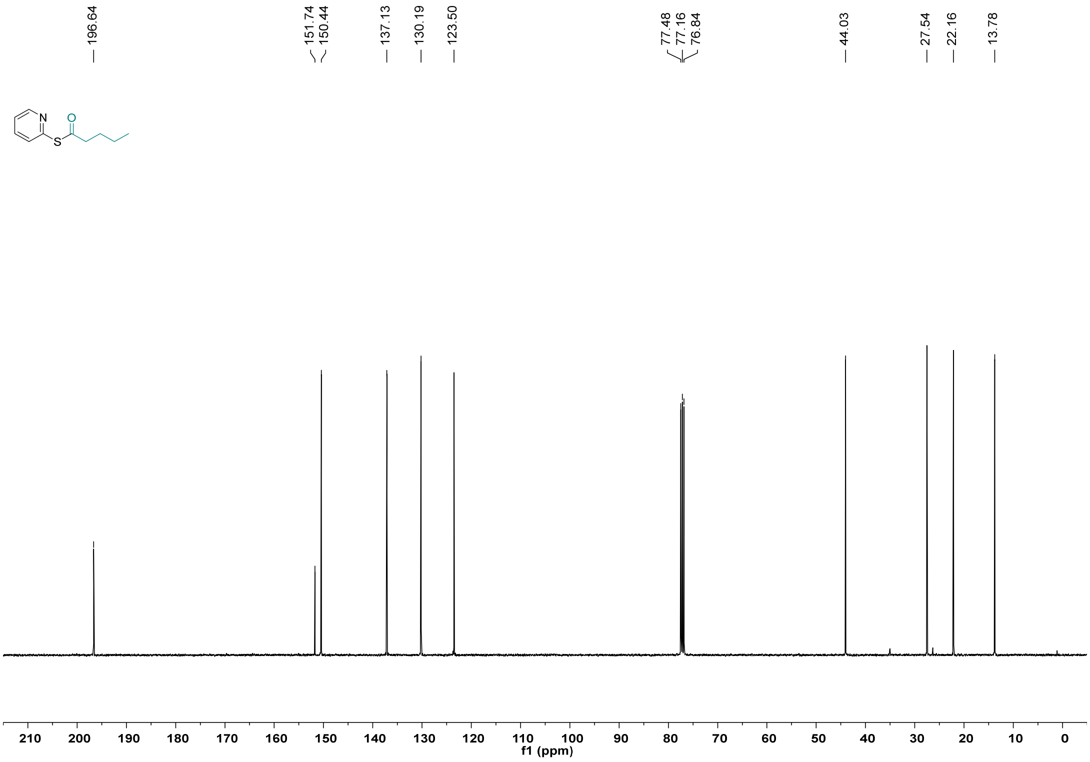

^{13}C NMR (100 MHz, $CDCl_3$) of compound **S27**

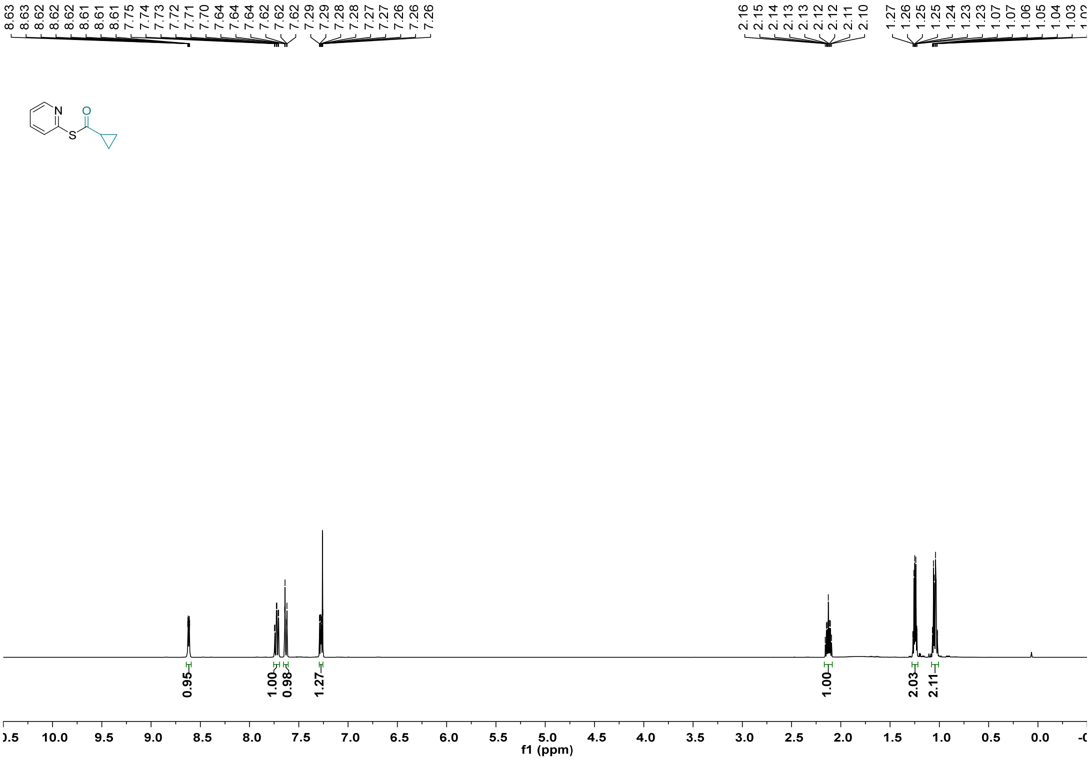

^{1}H NMR (400 MHz, $CDCl_3$) of compound **S28**

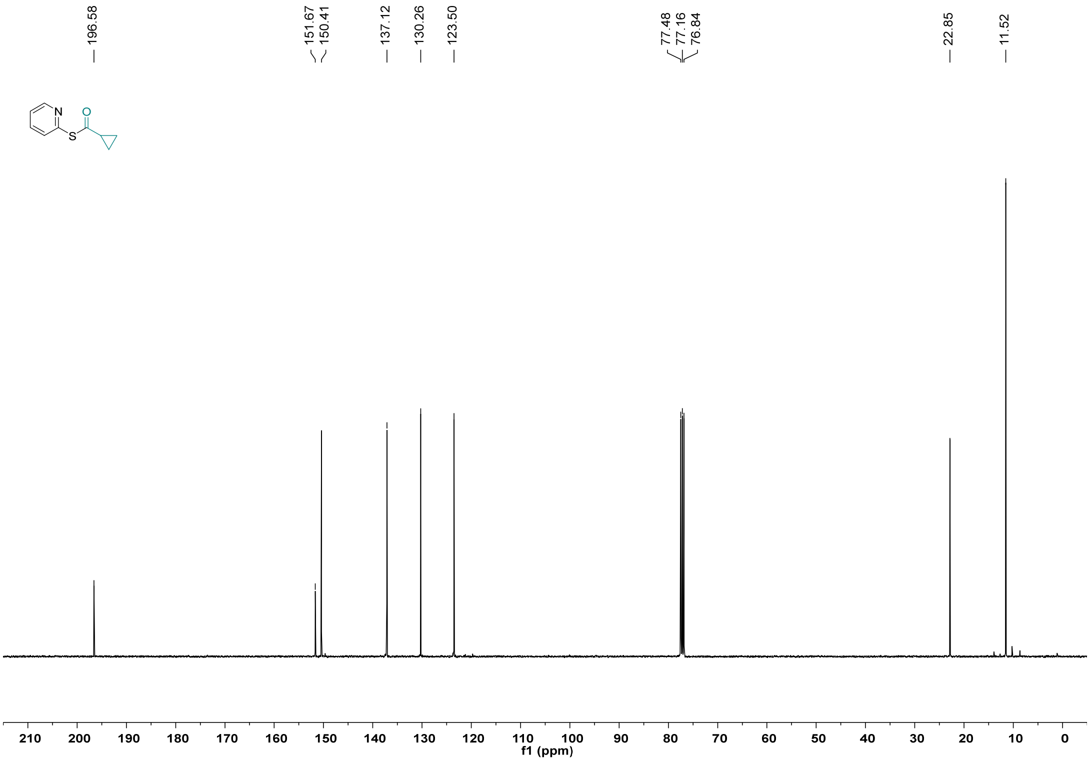

^{13}C NMR (100 MHz, CDCl$_3$) of compound **S28**

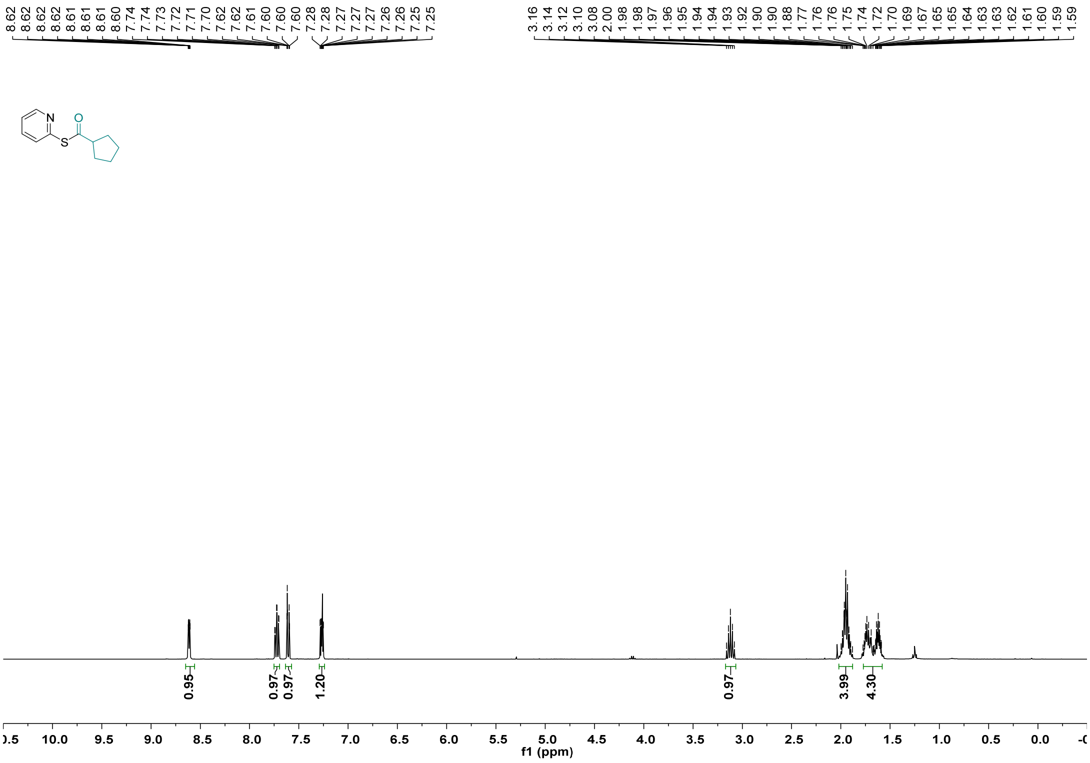

^{1}H NMR (400 MHz, CDCl$_3$) of compound **S29**

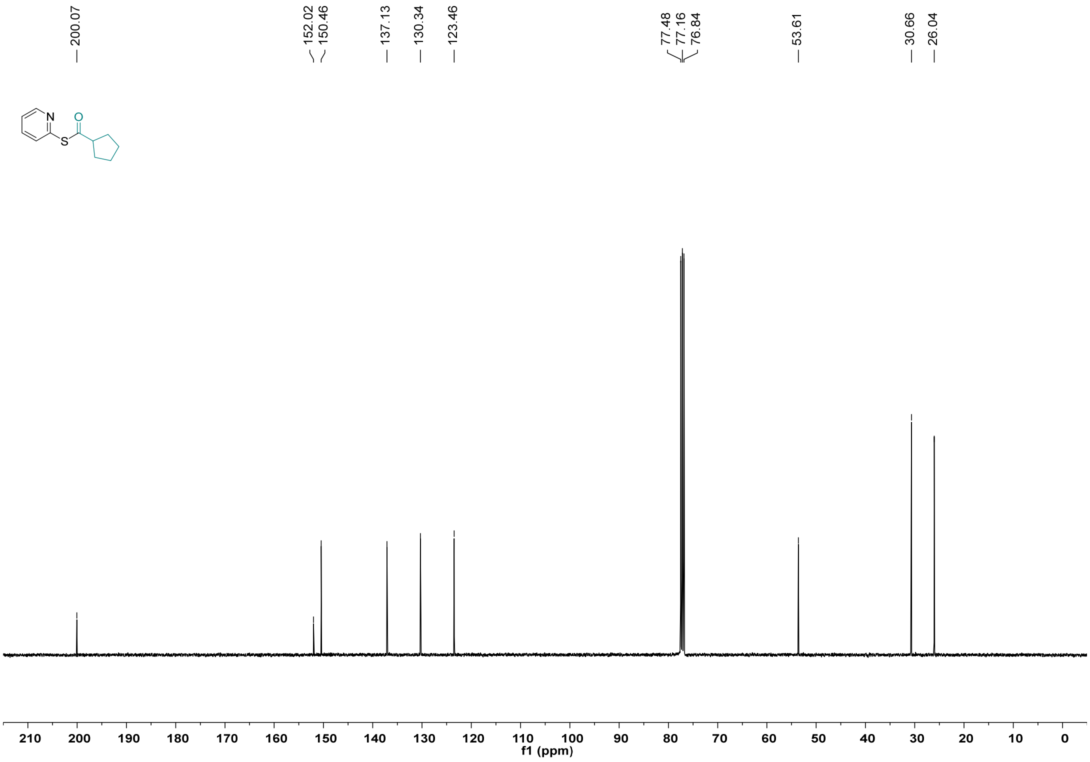

^{13}C NMR (100 MHz, $CDCl_3$) of compound **S29**

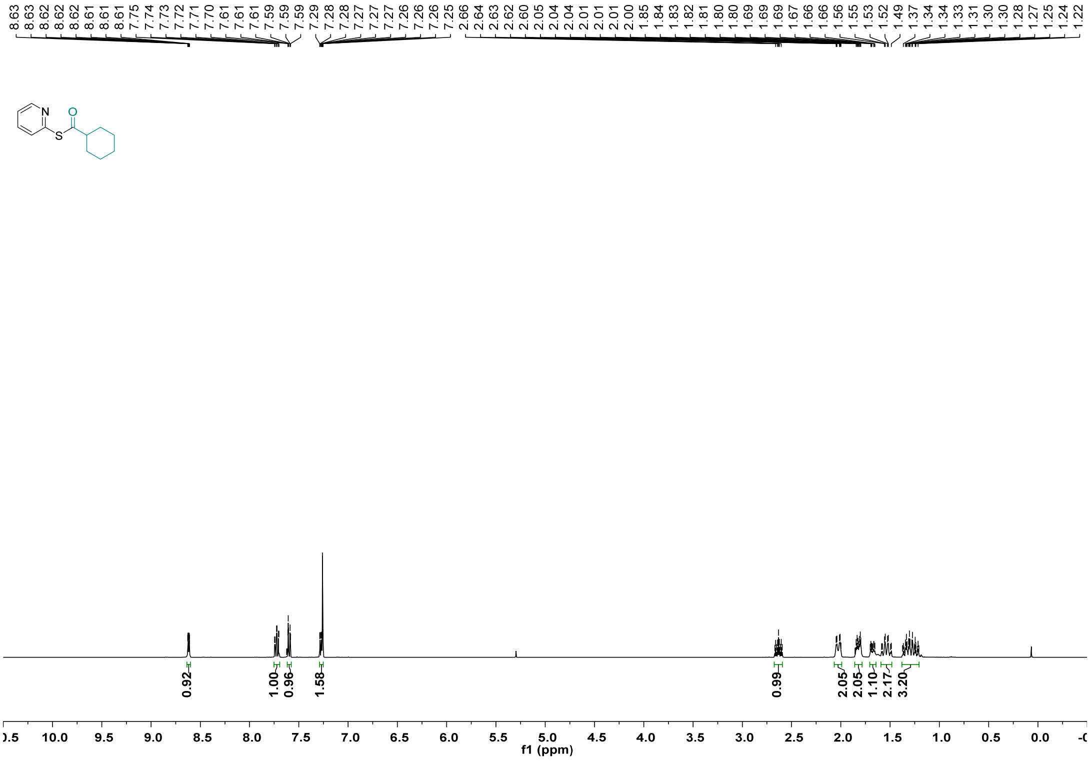

^{1}H NMR (400 MHz, $CDCl_3$) of compound **S30**

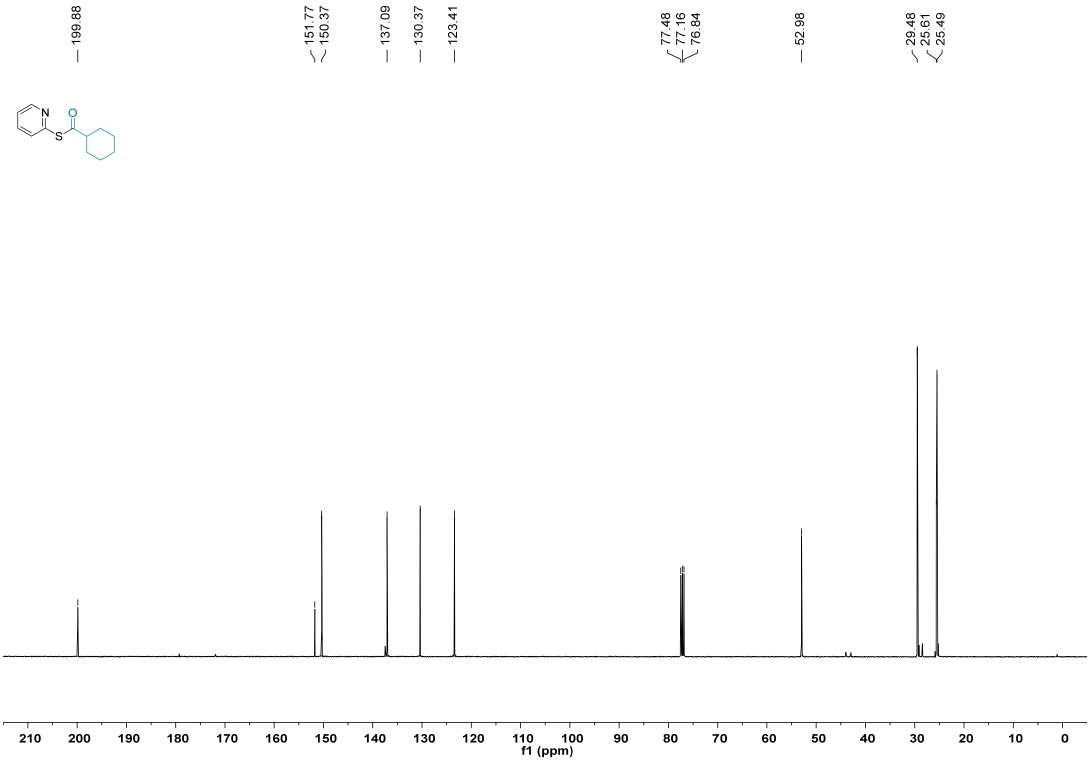

^{13}C NMR (100 MHz, $CDCl_3$) of compound **S30**

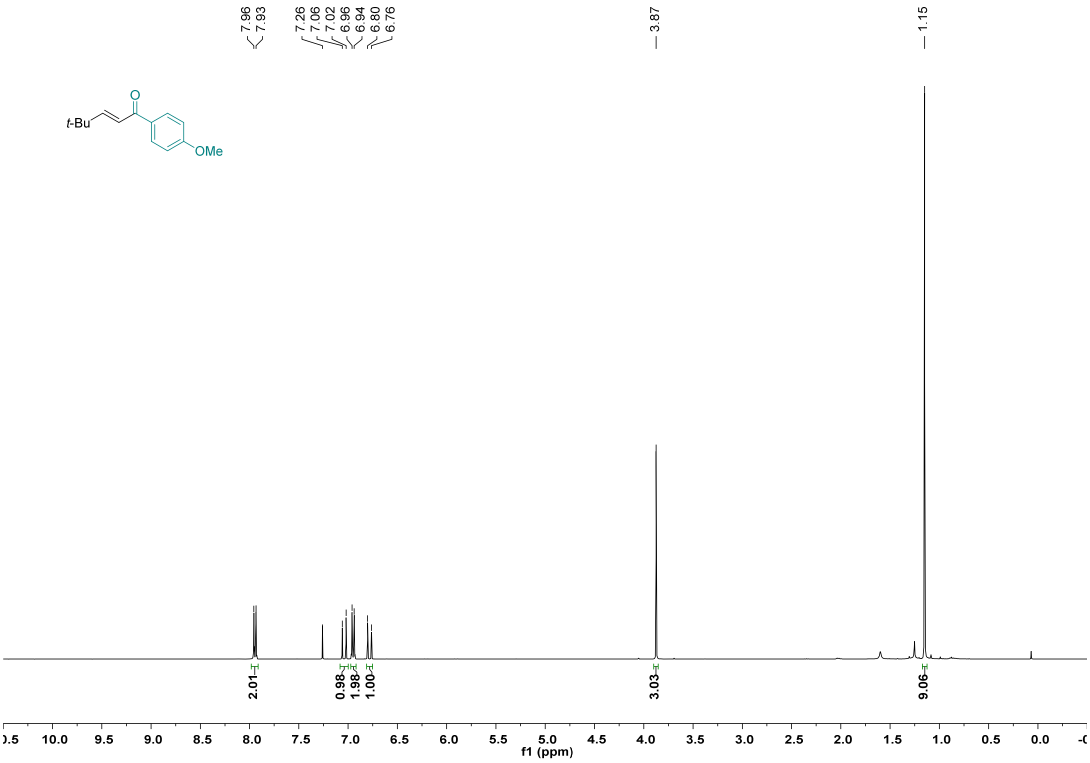

^{1}H NMR (400 MHz, $CDCl_3$) of compound **3**

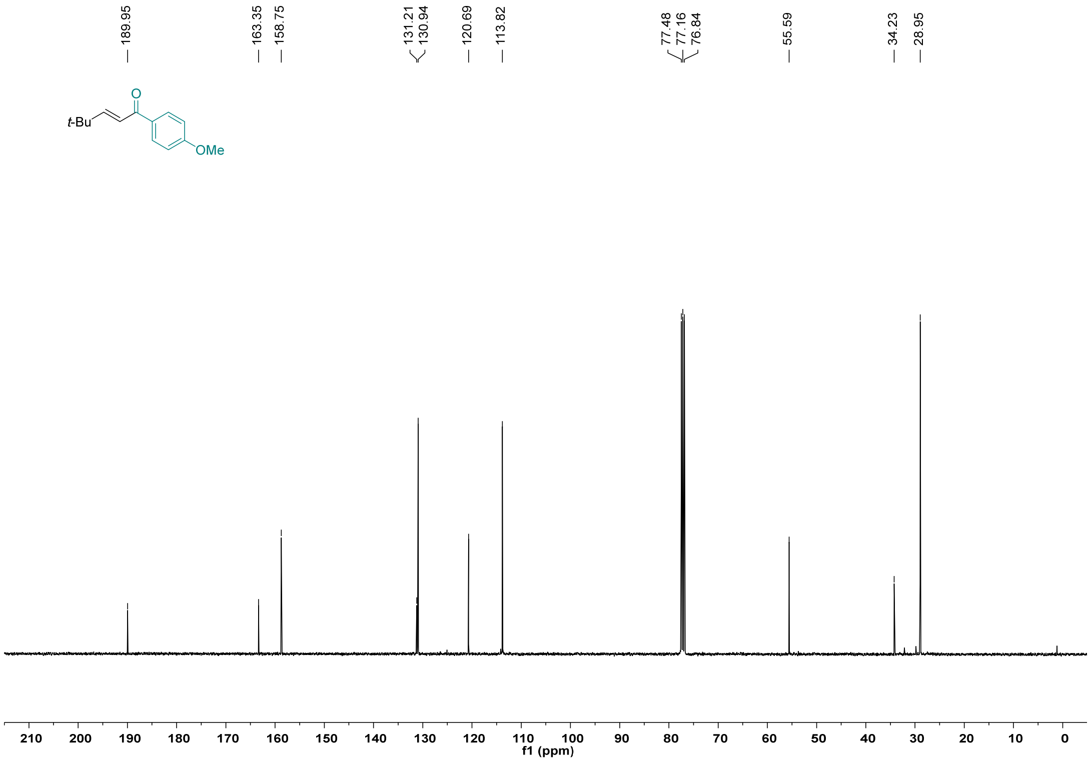

^{13}C NMR (100 MHz, $CDCl_3$) of compound **3**

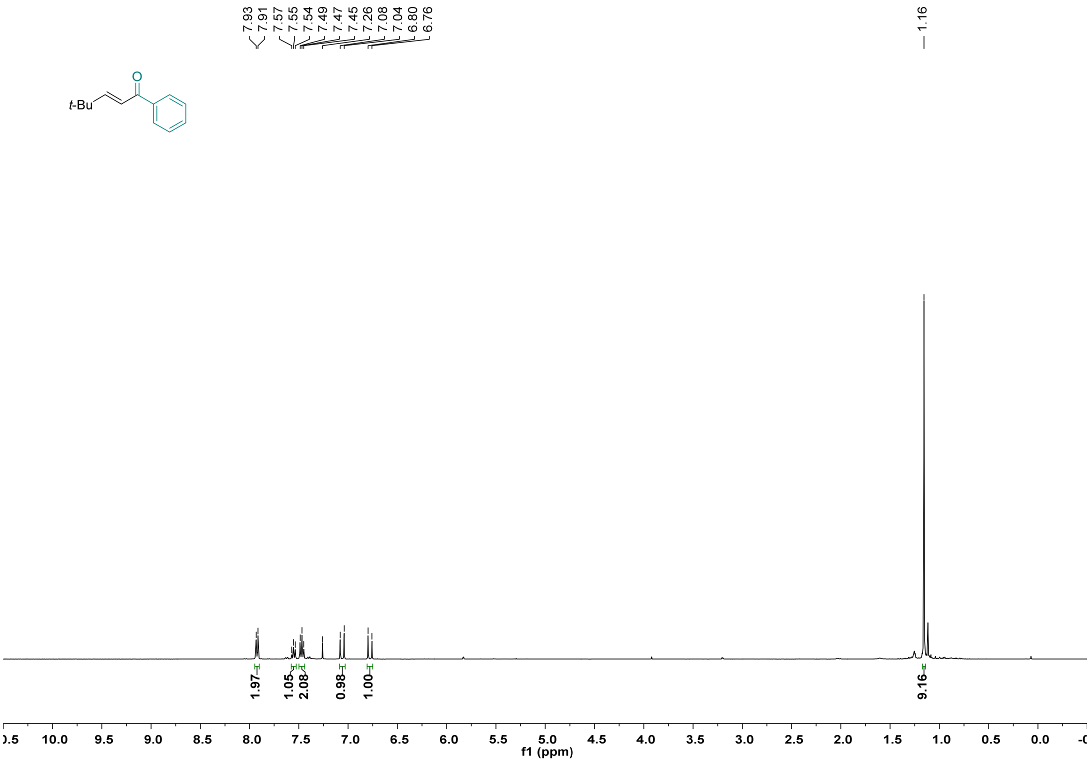

^{1}H NMR (400 MHz, $CDCl_3$) of compound **4**

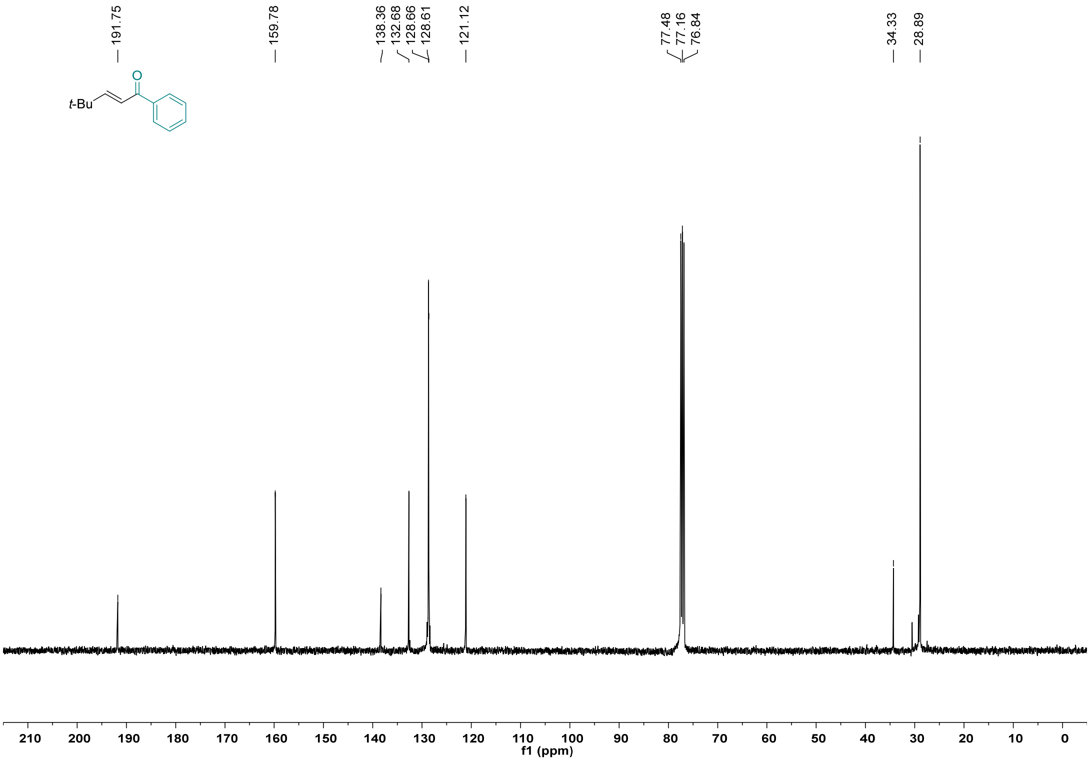

^{13}C NMR (100 MHz, CDCl$_3$) of compound **4**

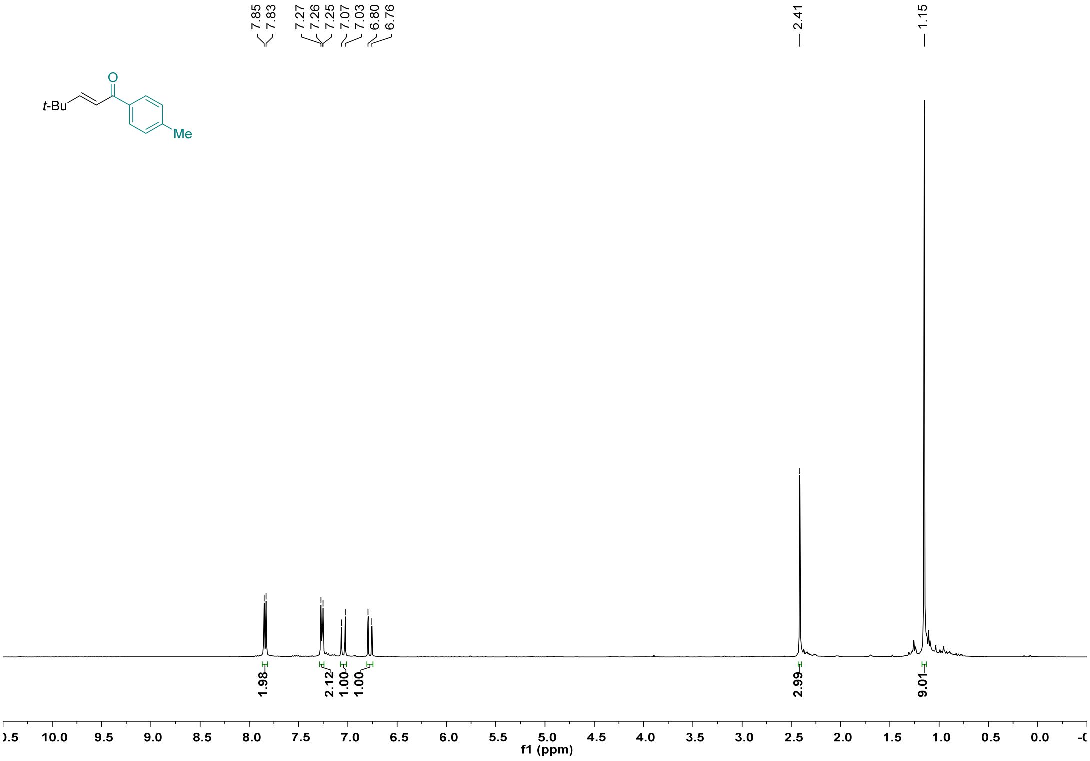

^{1}H NMR (400 MHz, CDCl$_3$) of compound **5**

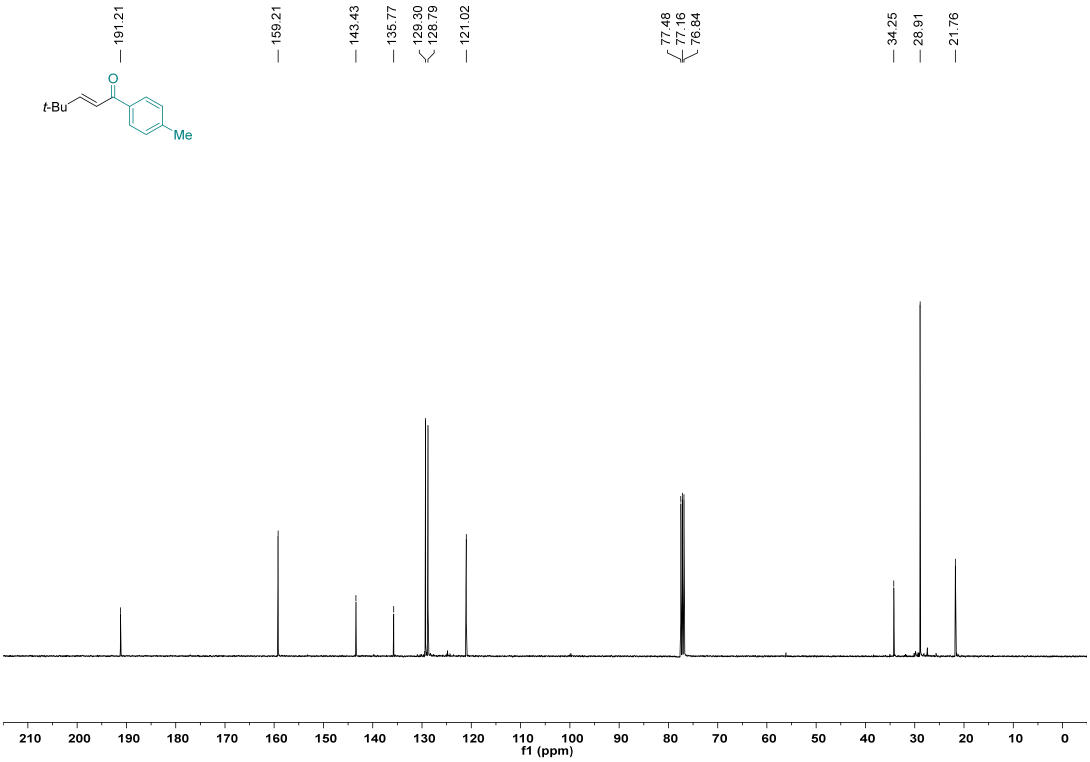

^{13}C NMR (100 MHz, CDCl$_3$) of compound **5**

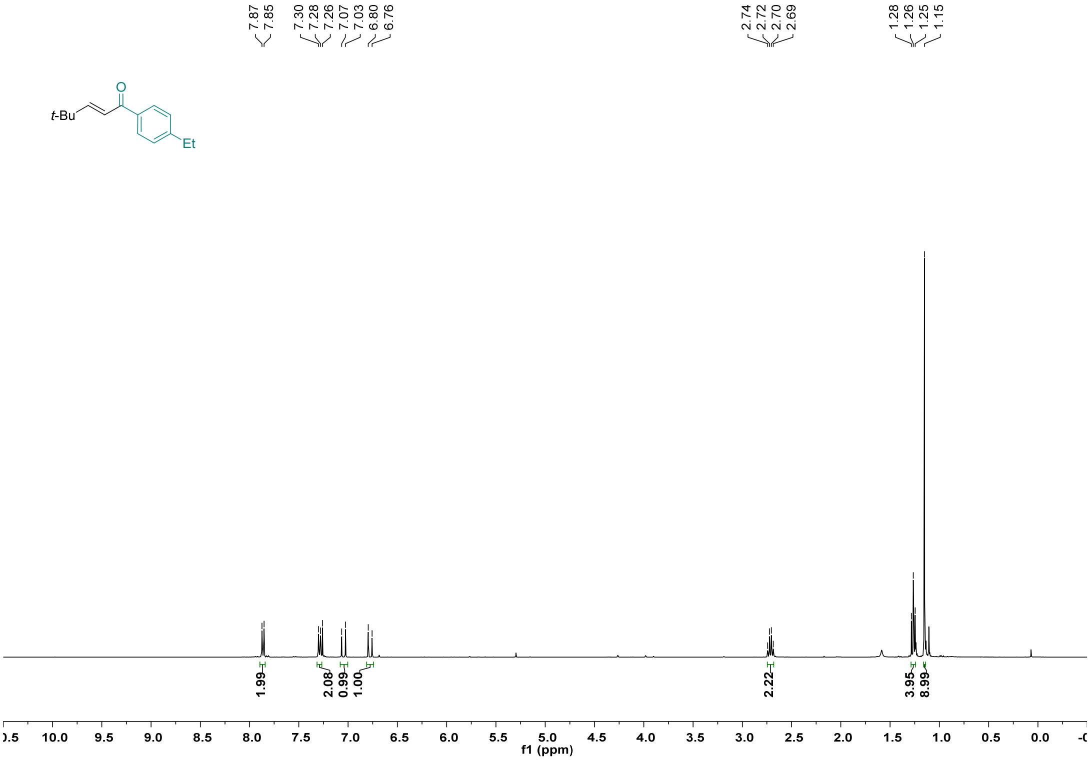

^{1}H NMR (400 MHz, CDCl$_3$) of compound **6**

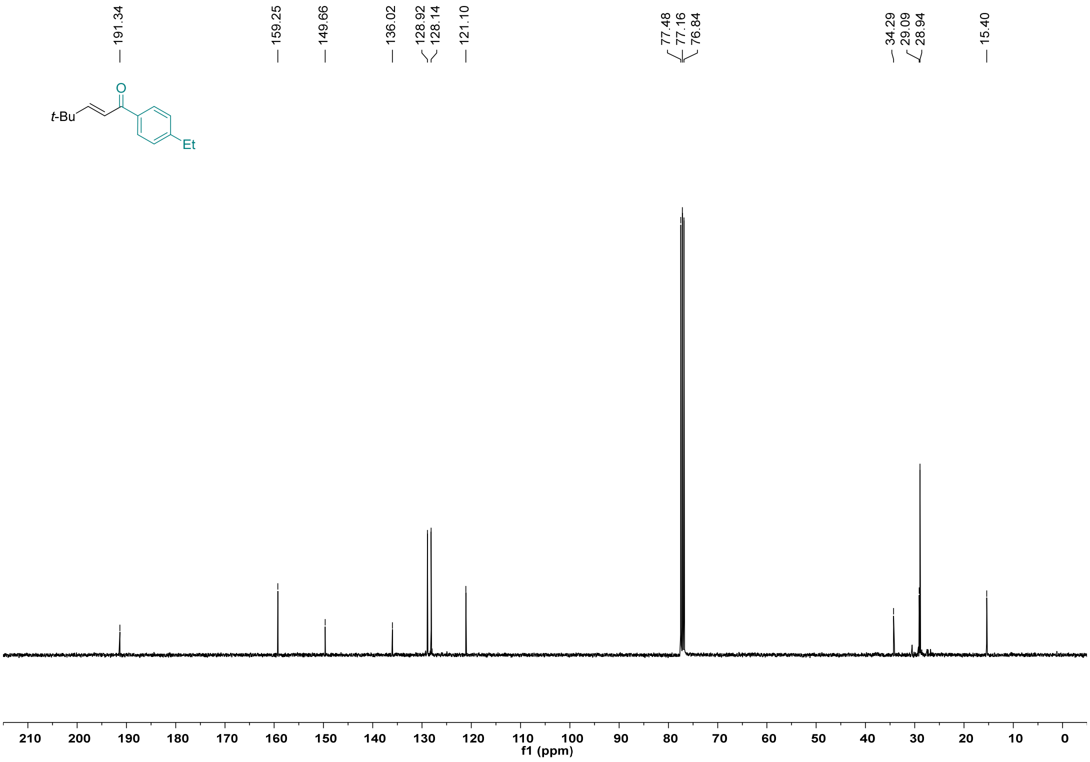

^{13}C NMR (100 MHz, $CDCl_3$) of compound **6**

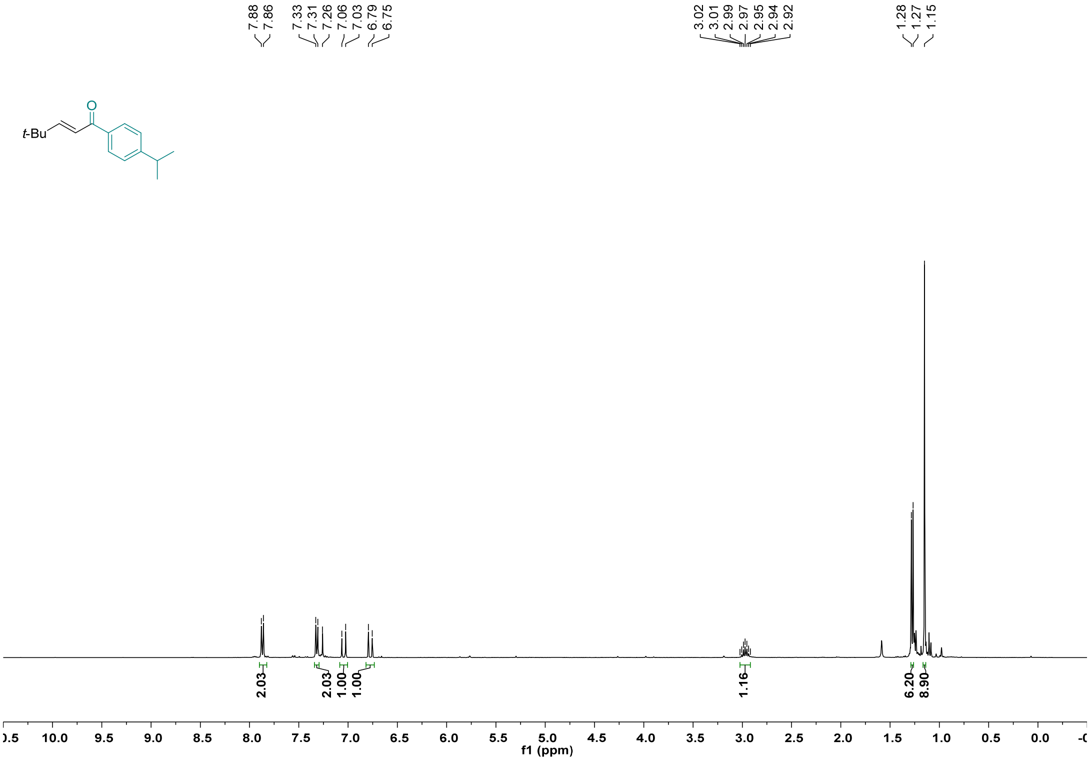

^{1}H NMR (400 MHz, $CDCl_3$) of compound **7**

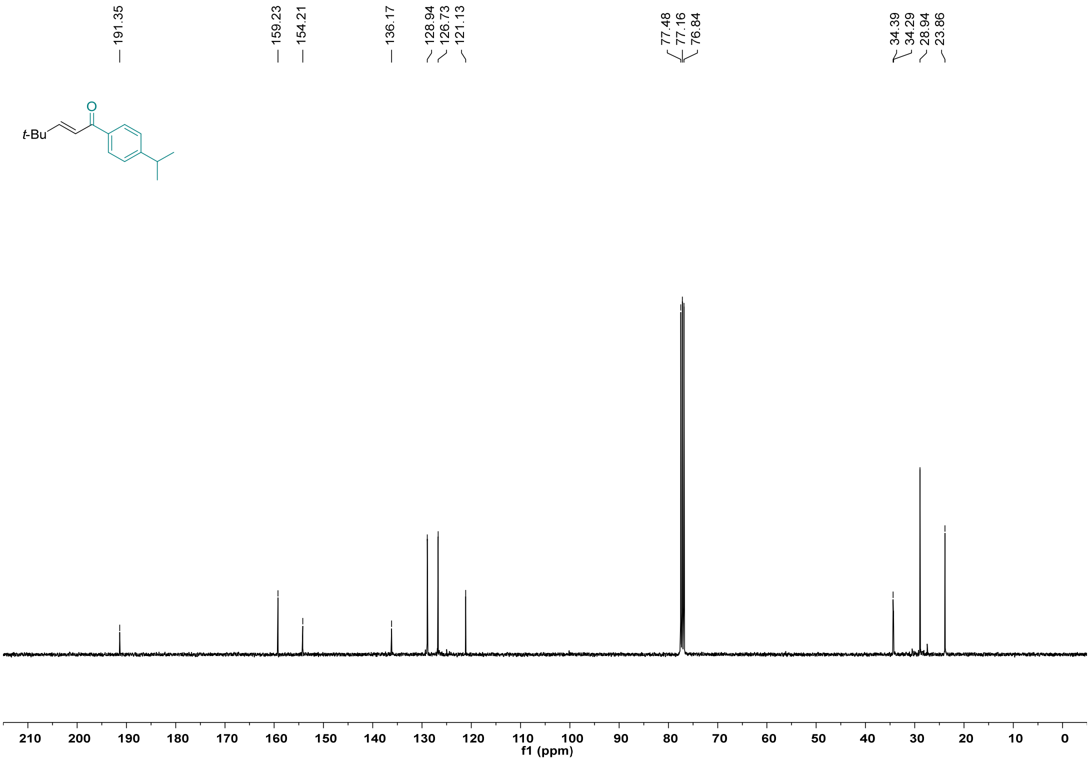

^{13}C NMR (100 MHz, $CDCl_3$) of compound **7**

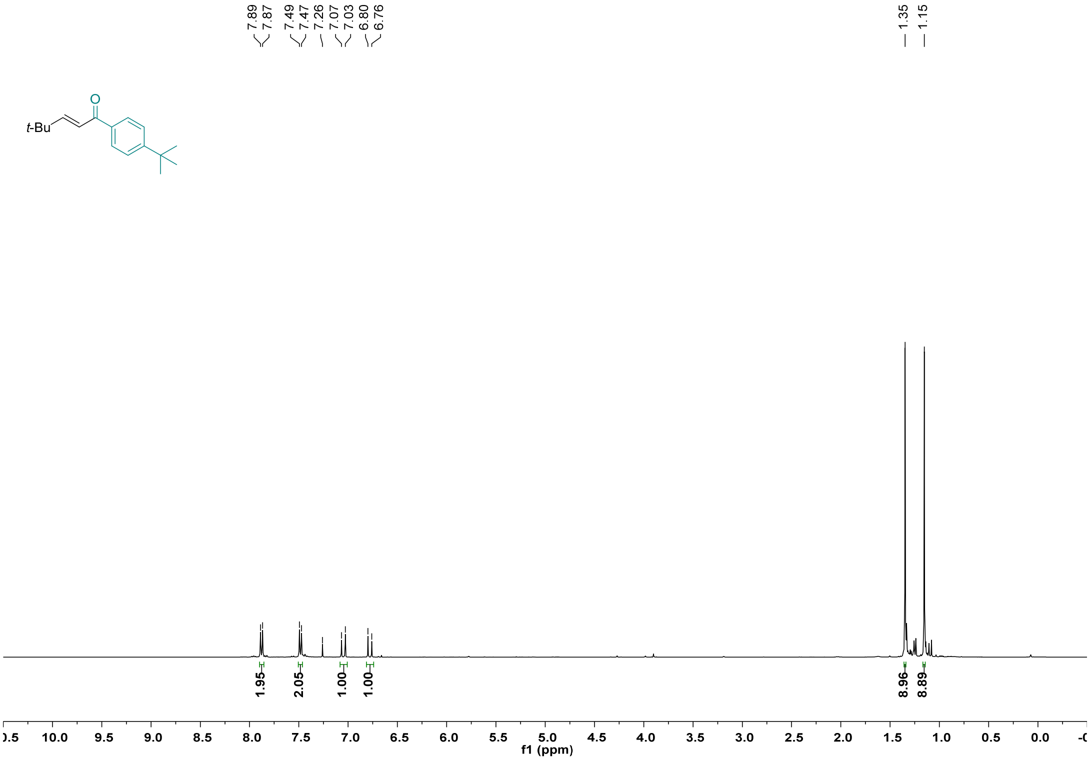

^{1}H NMR (400 MHz, $CDCl_3$) of compound **8**

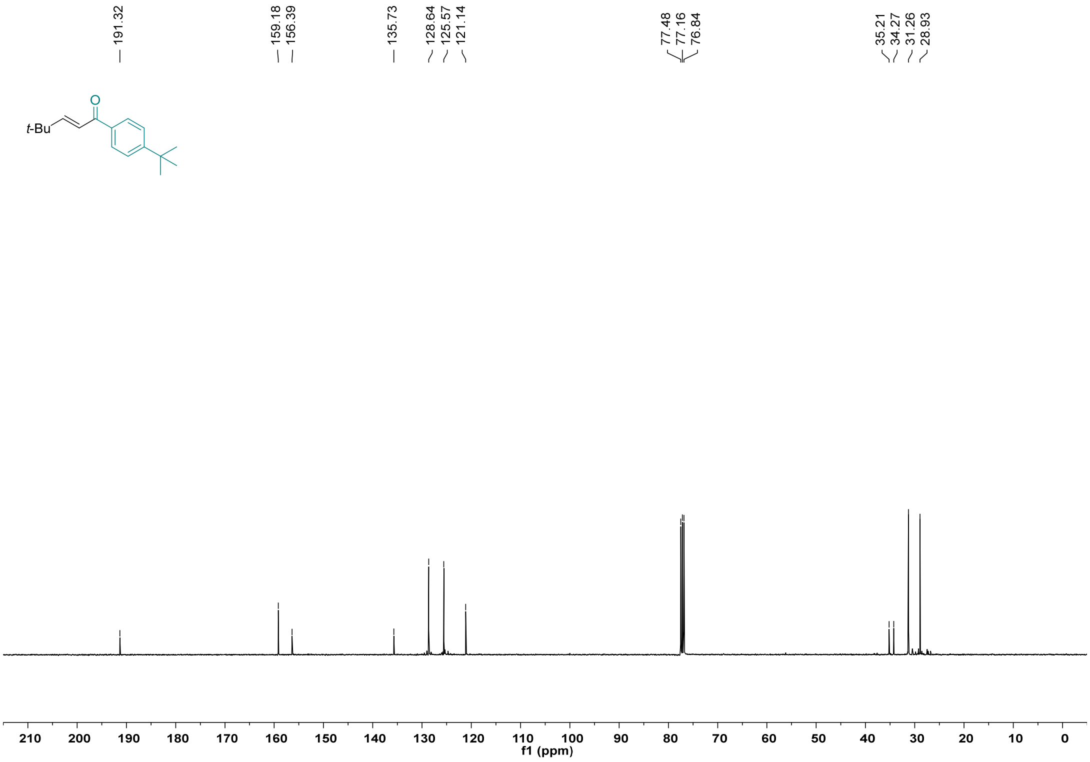

^{13}C NMR (100 MHz, $CDCl_3$) of compound **8**

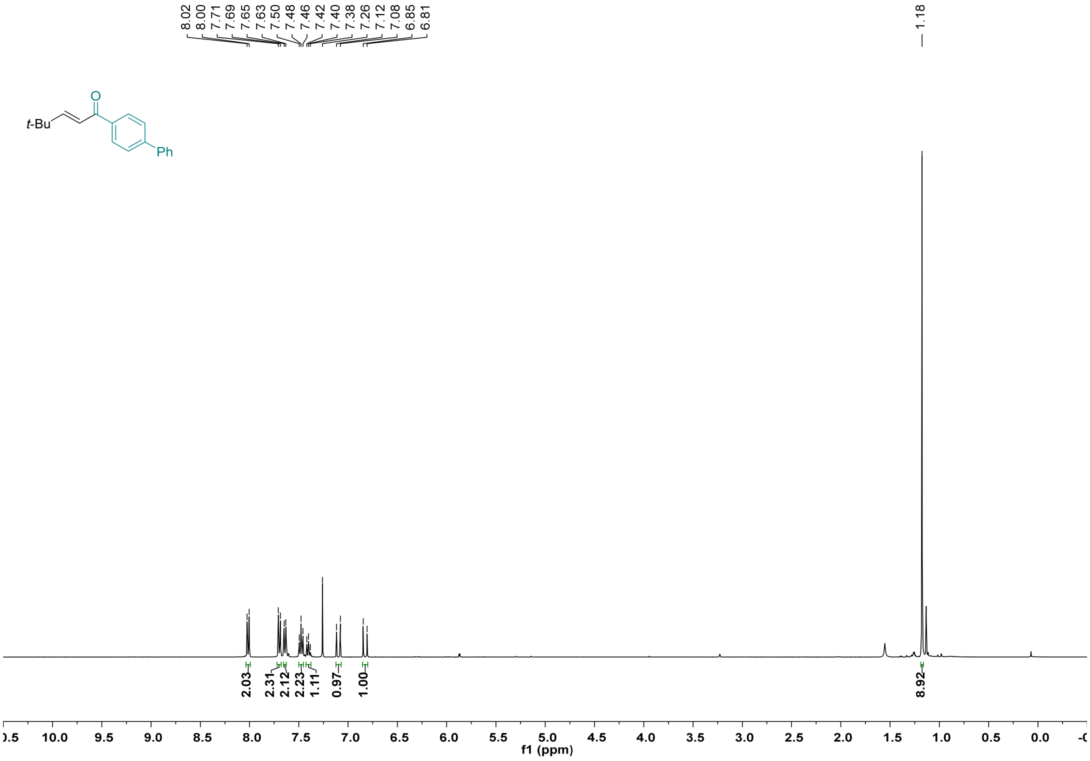

^{1}H NMR (400 MHz, $CDCl_3$) of compound **9**

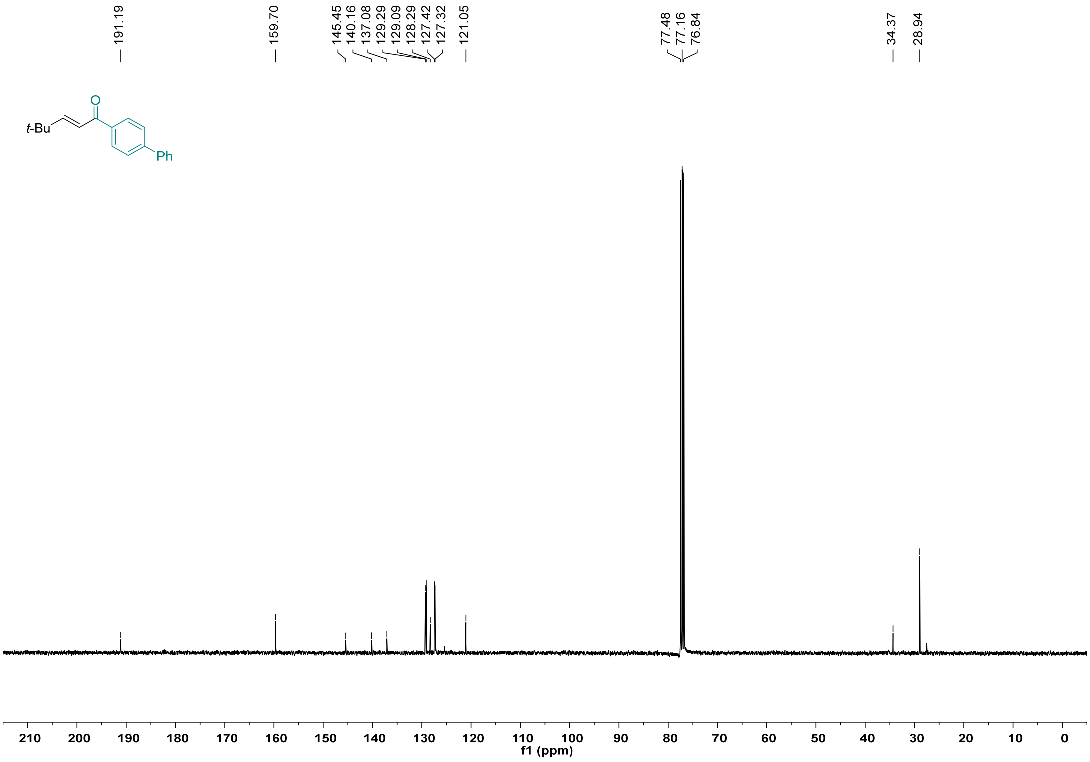

^{13}C NMR (100 MHz, CDCl$_3$) of compound **9**

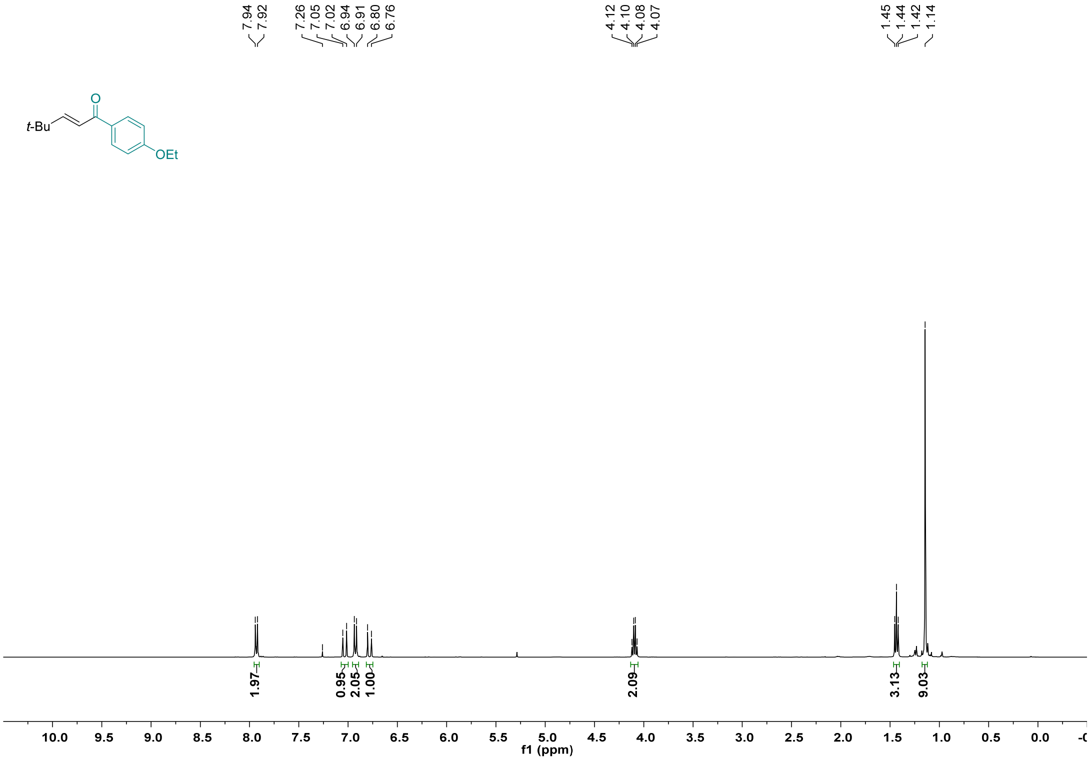

^{1}H NMR (400 MHz, CDCl$_3$) of compound **10**

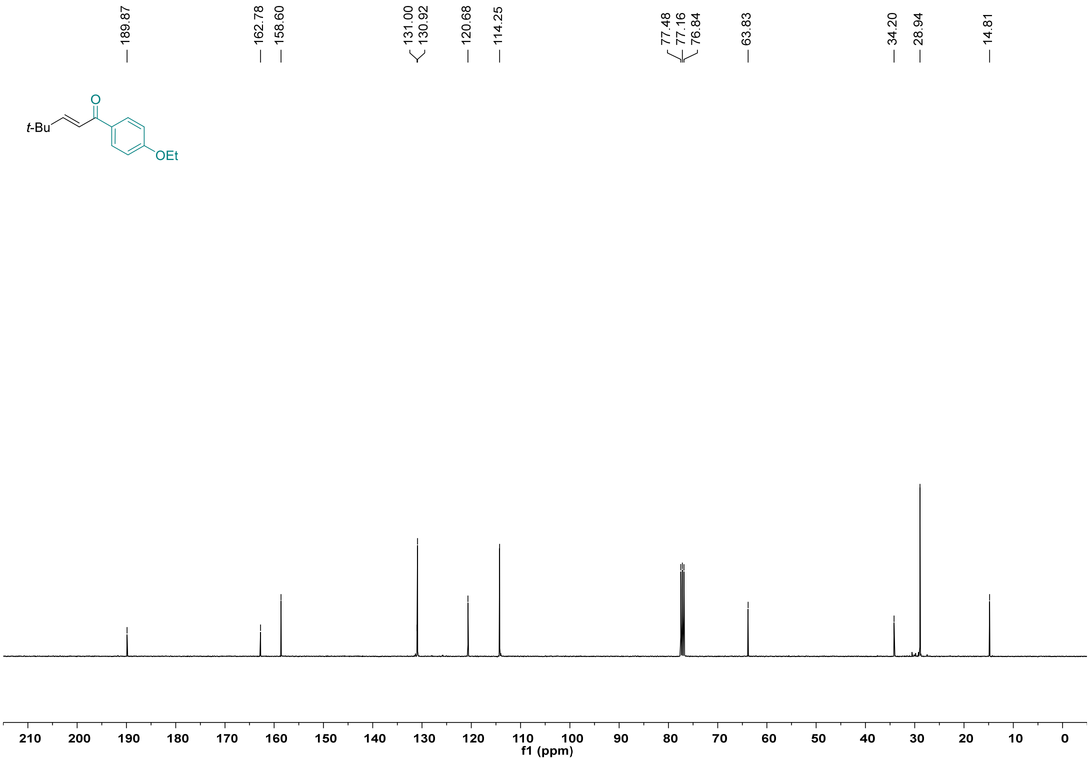

^{13}C NMR (100 MHz, CDCl$_3$) of compound **10**

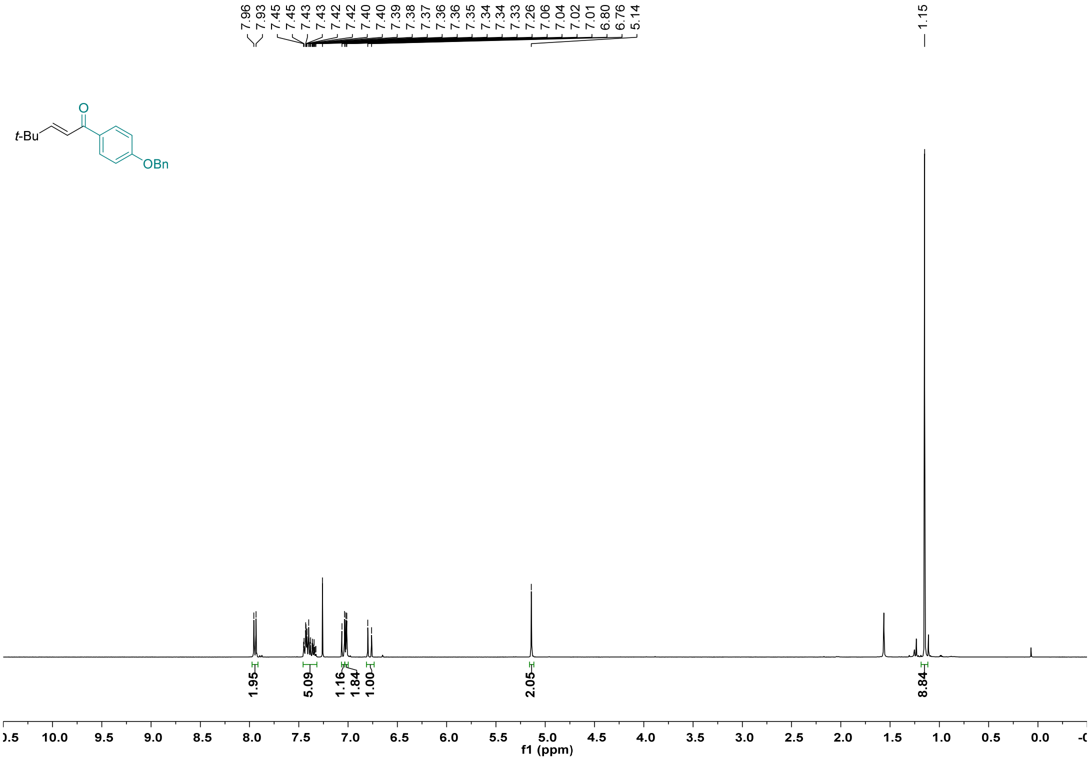

^{1}H NMR (400 MHz, CDCl$_3$) of compound **11**

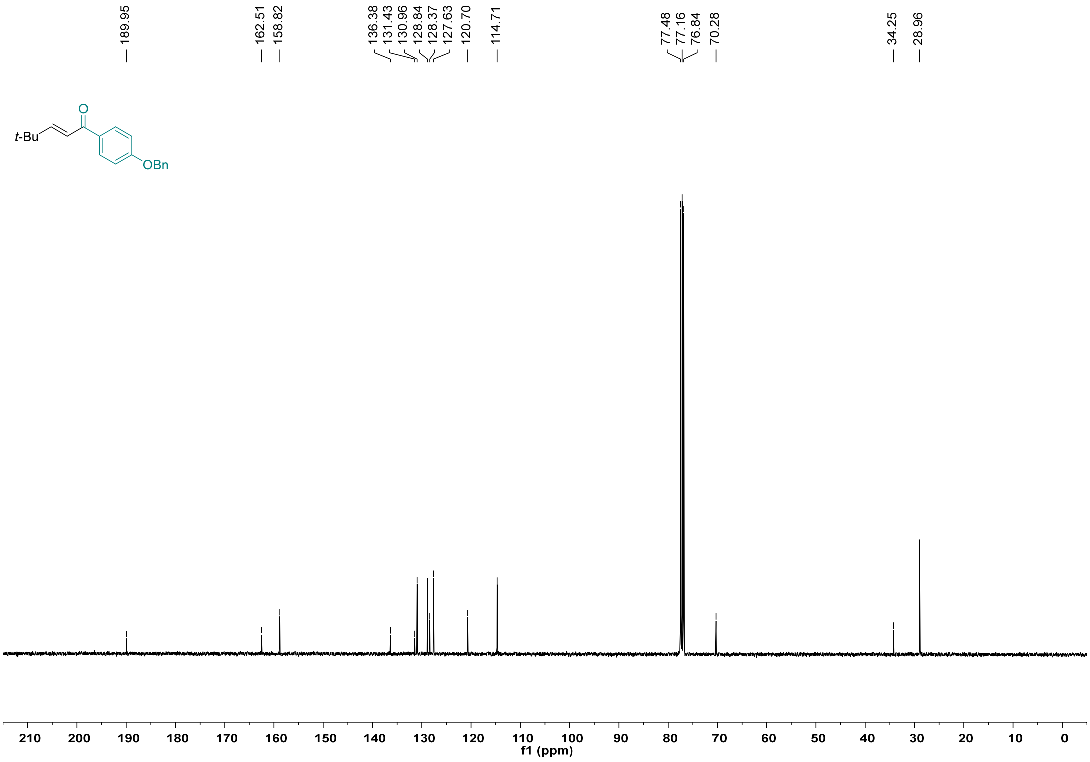

^{13}C NMR (100 MHz, CDCl$_3$) of compound **11**

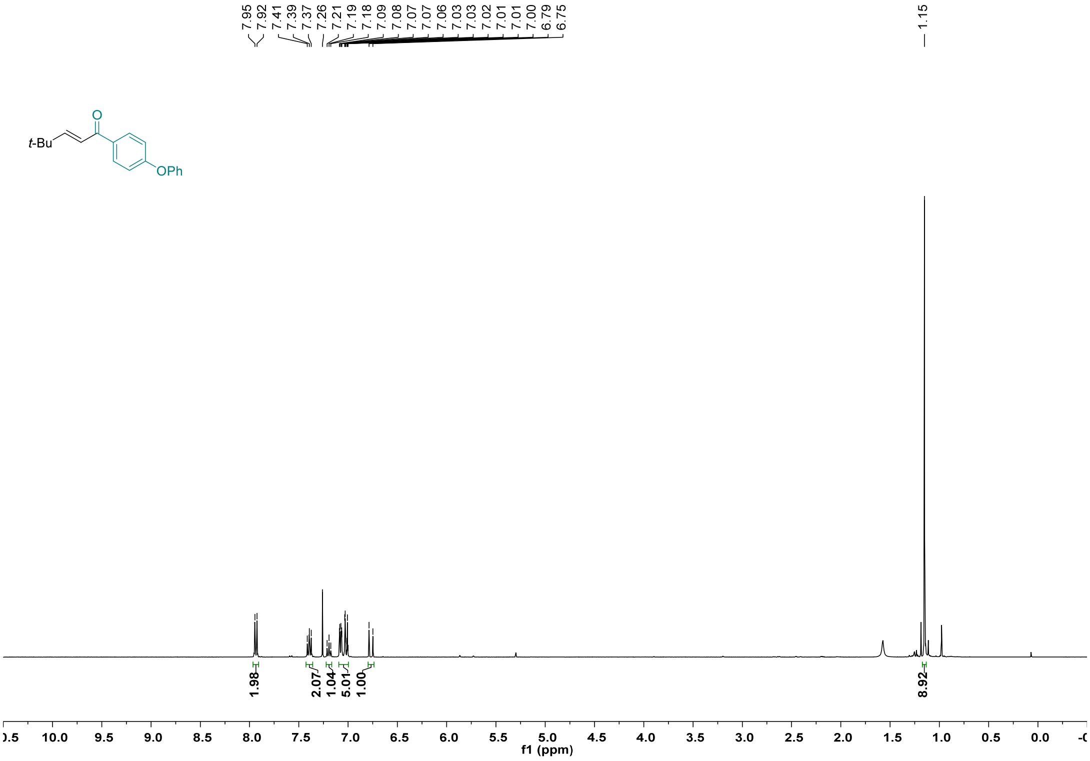

^{1}H NMR (400 MHz, CDCl$_3$) of compound **12**

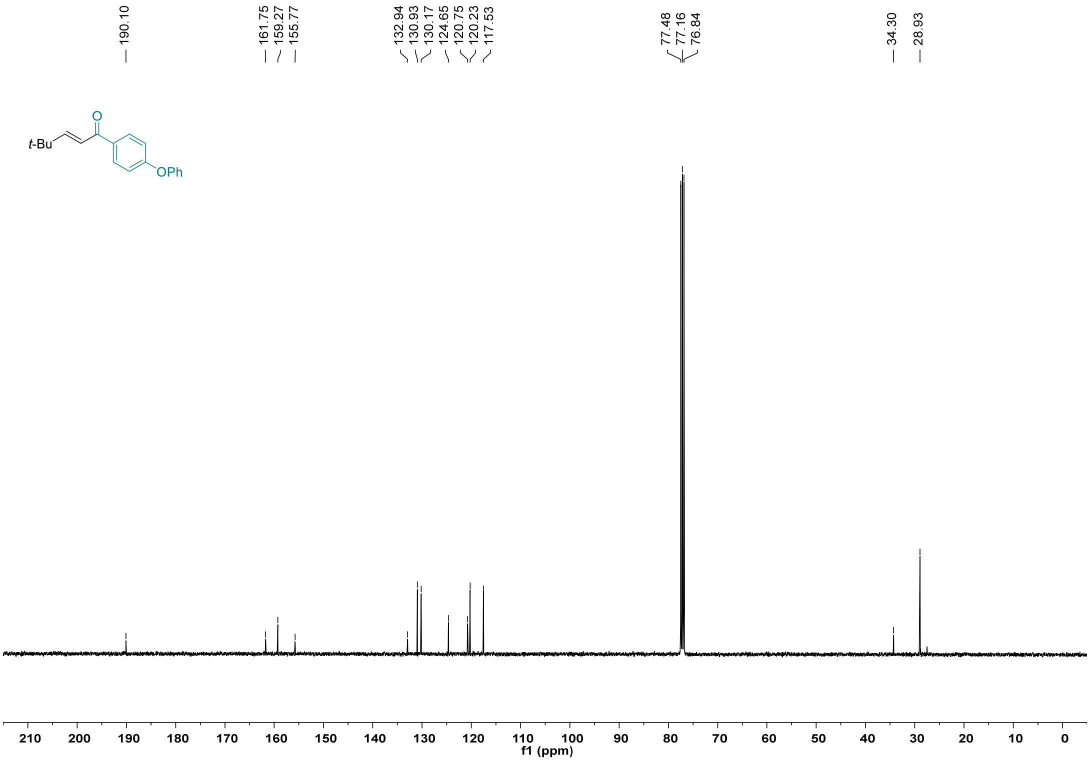

^{13}C NMR (100 MHz, $CDCl_3$) of compound **12**

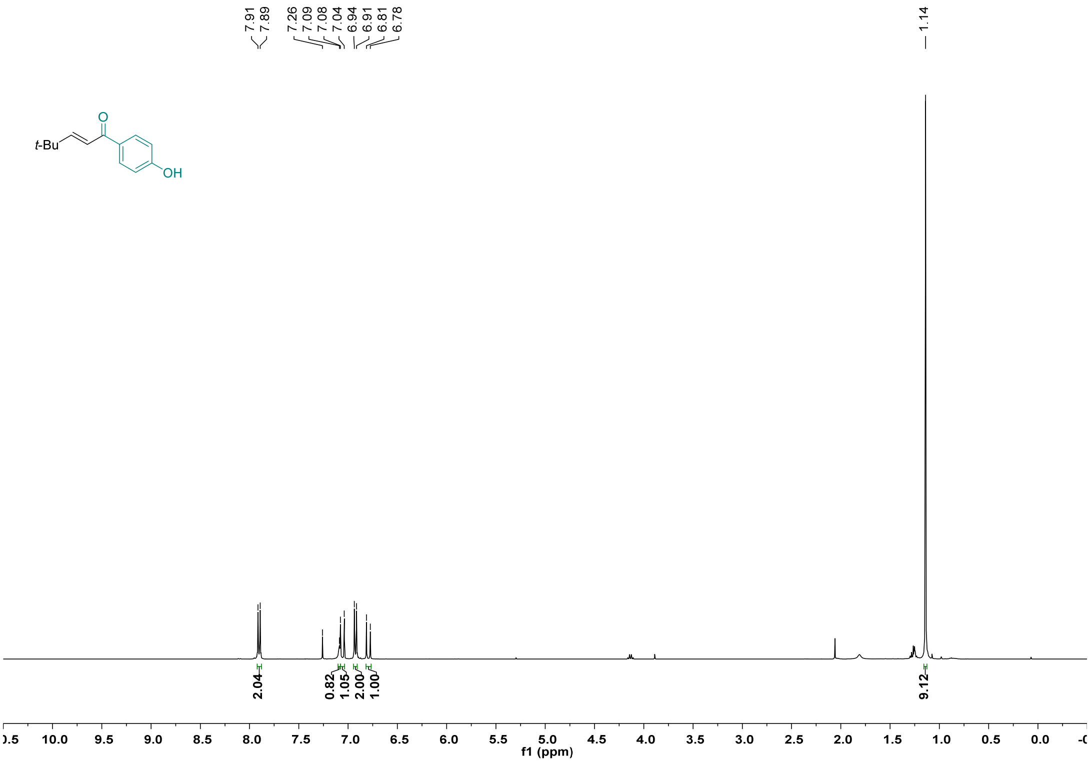

^{1}H NMR (400 MHz, $CDCl_3$) of compound **13**

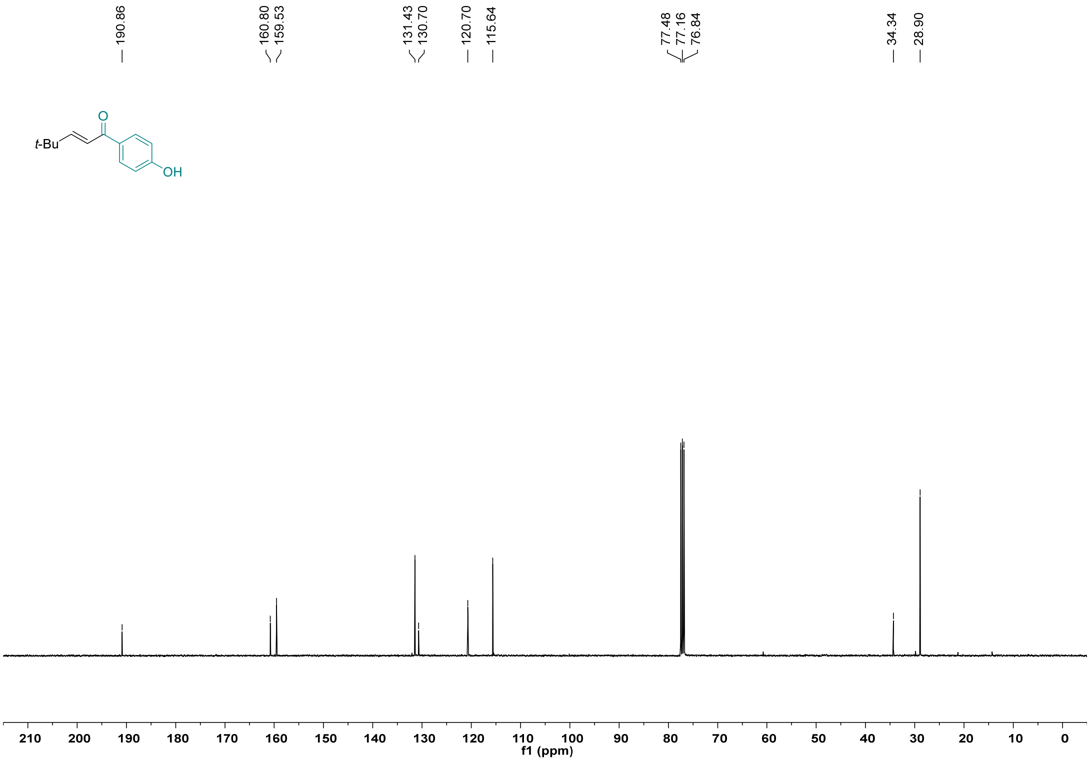

^{13}C NMR (100 MHz, $CDCl_3$) of compound **13**

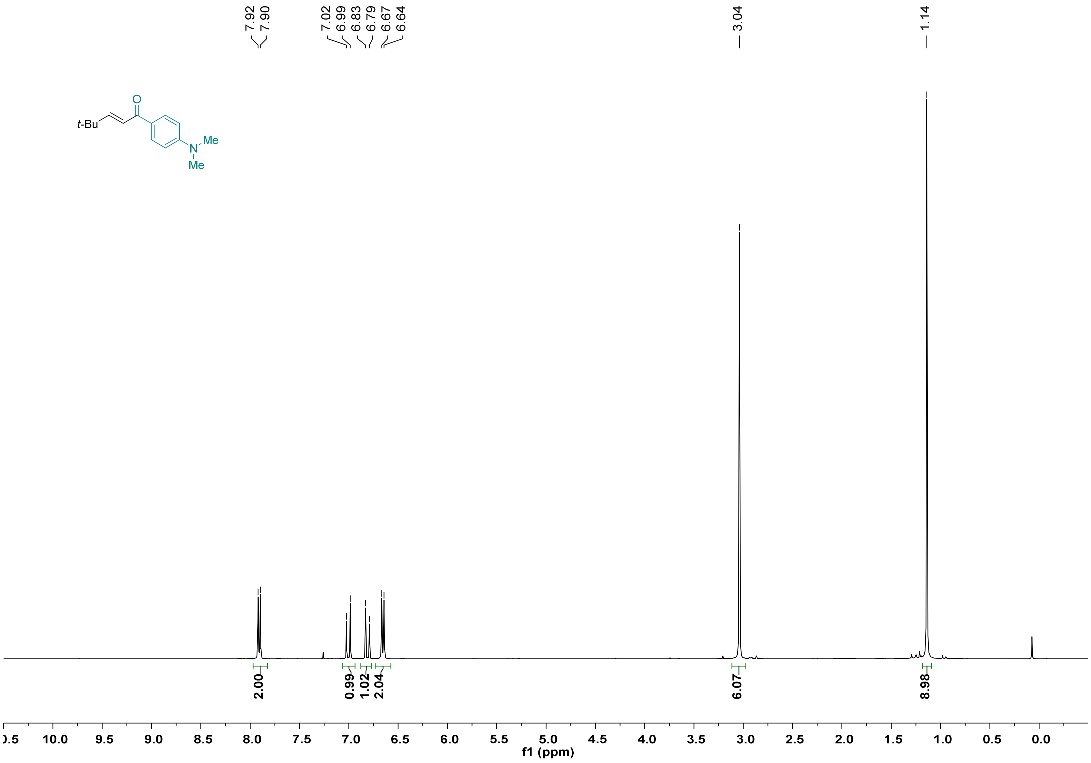

^{1}H NMR (400 MHz, $CDCl_3$) of compound **13’**

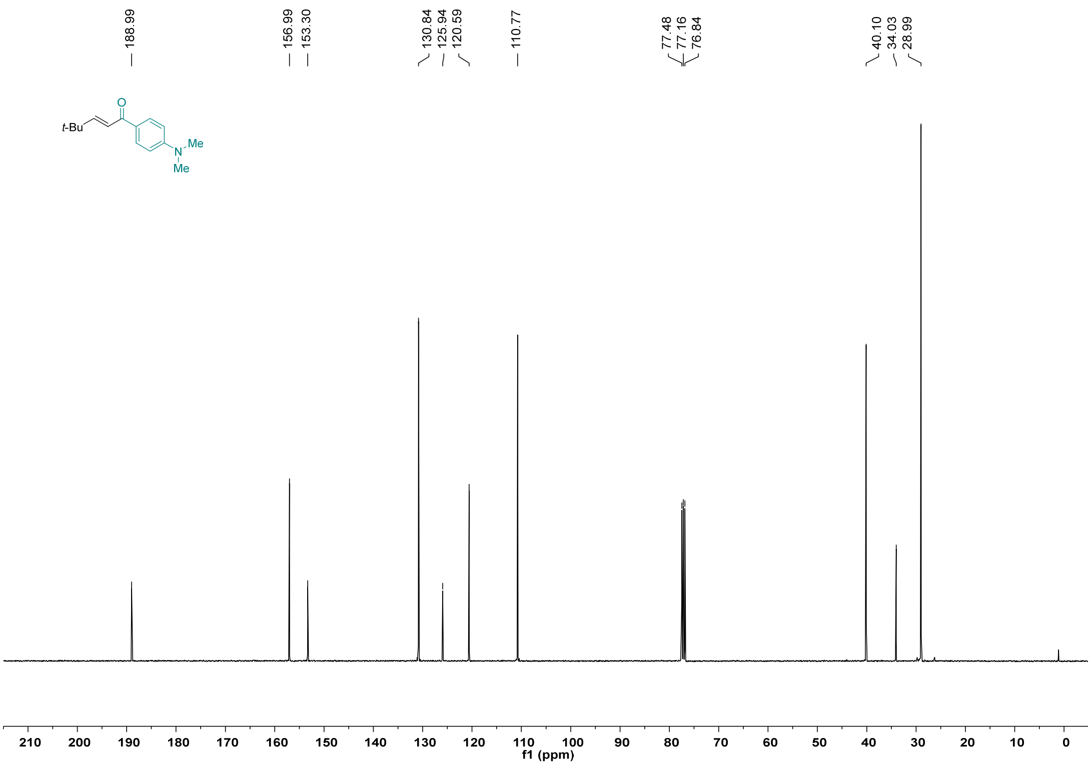

^{13}C NMR (100 MHz, CDCl$_3$) of compound **13’**

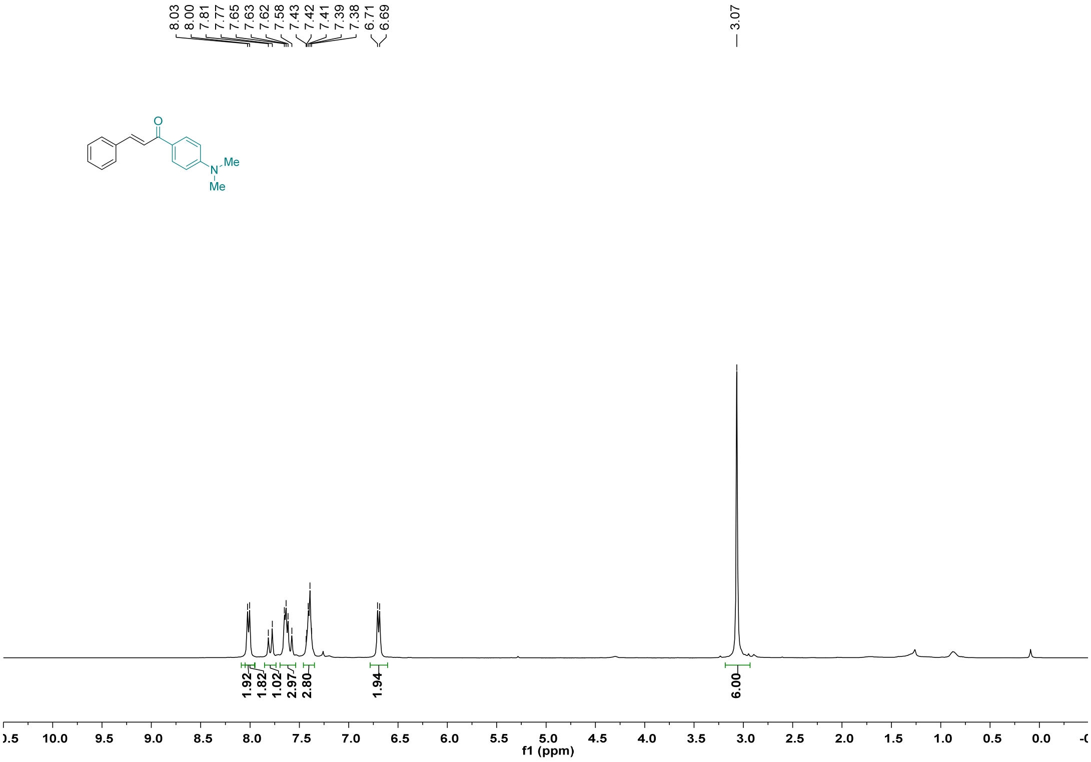

^{1}H NMR (400 MHz, CDCl$_3$) of compound **13’’**

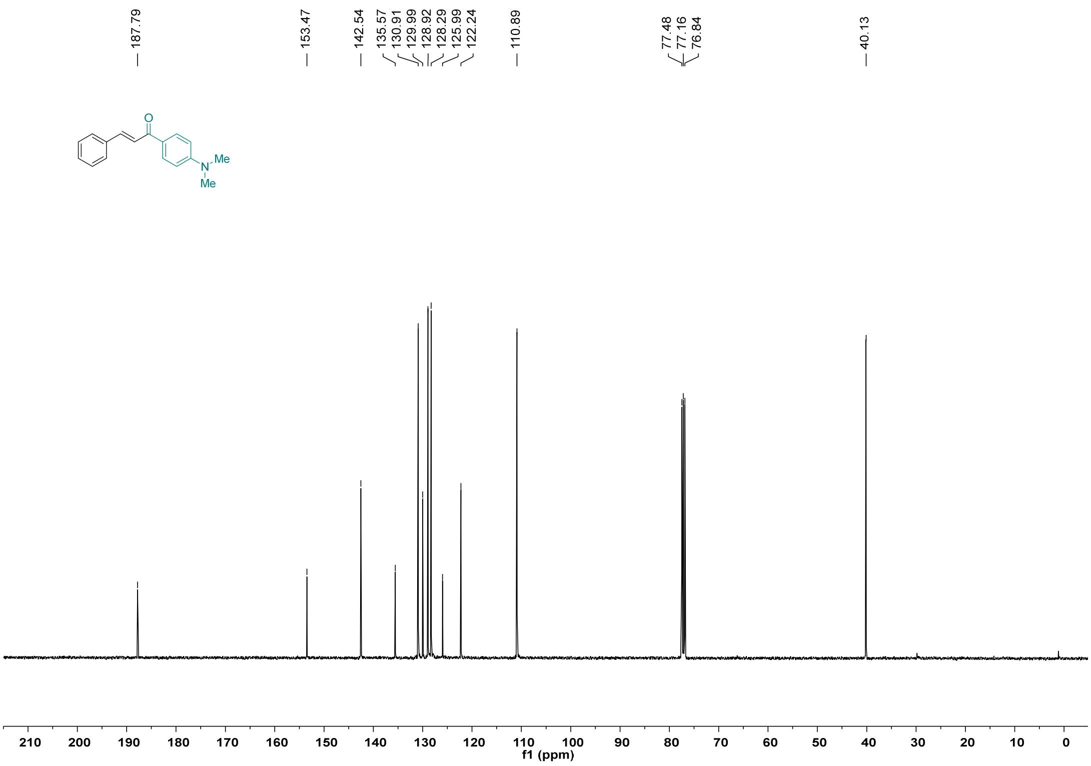

^{13}C NMR (100 MHz, $CDCl_3$) of compound **13''**

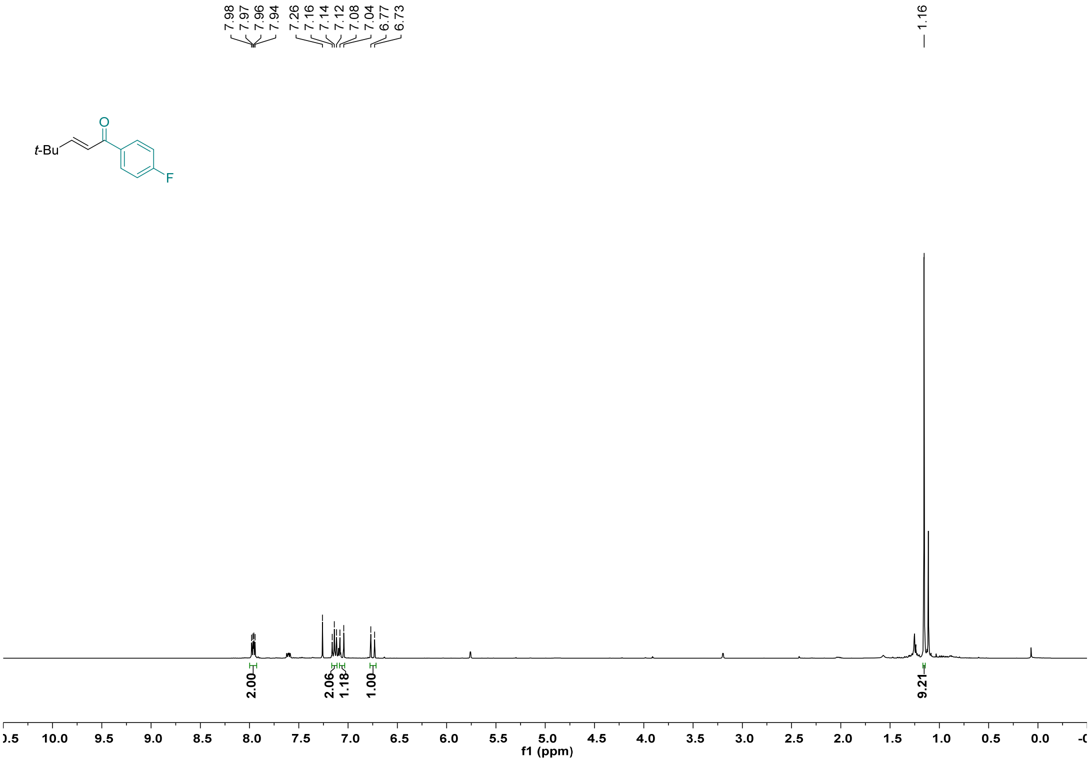

^{1}H NMR (400 MHz, $CDCl_3$) of compound **14**

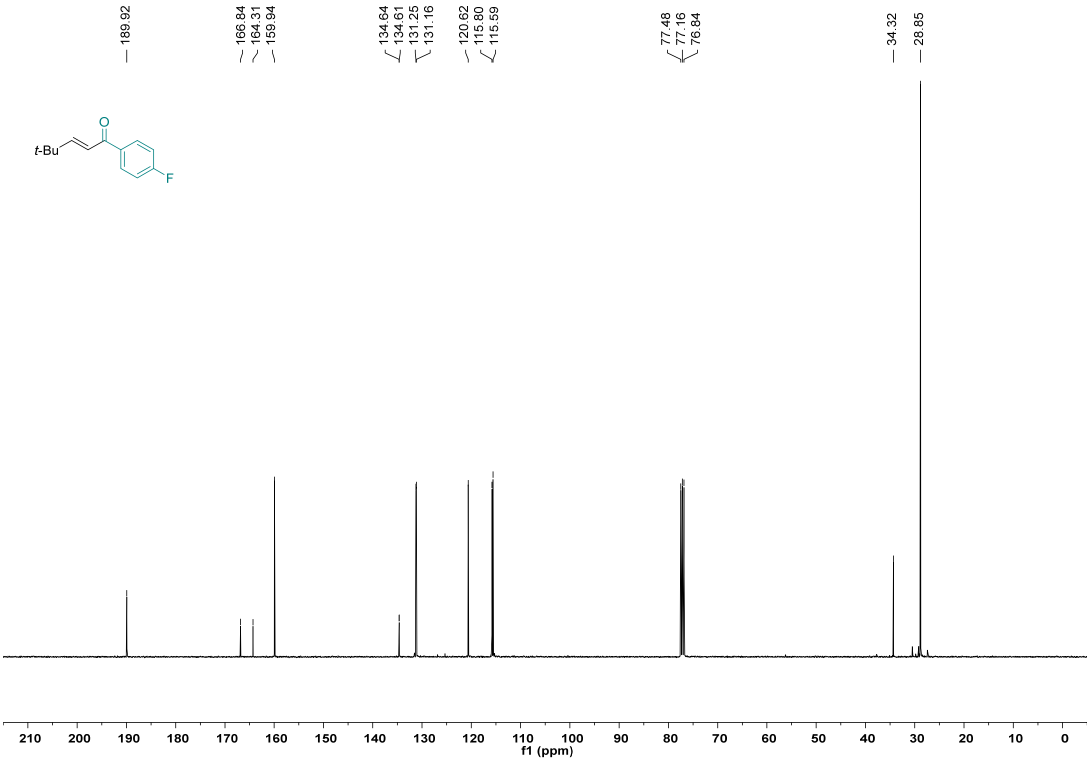

^{13}C NMR (100 MHz, $CDCl_3$) of compound **14**

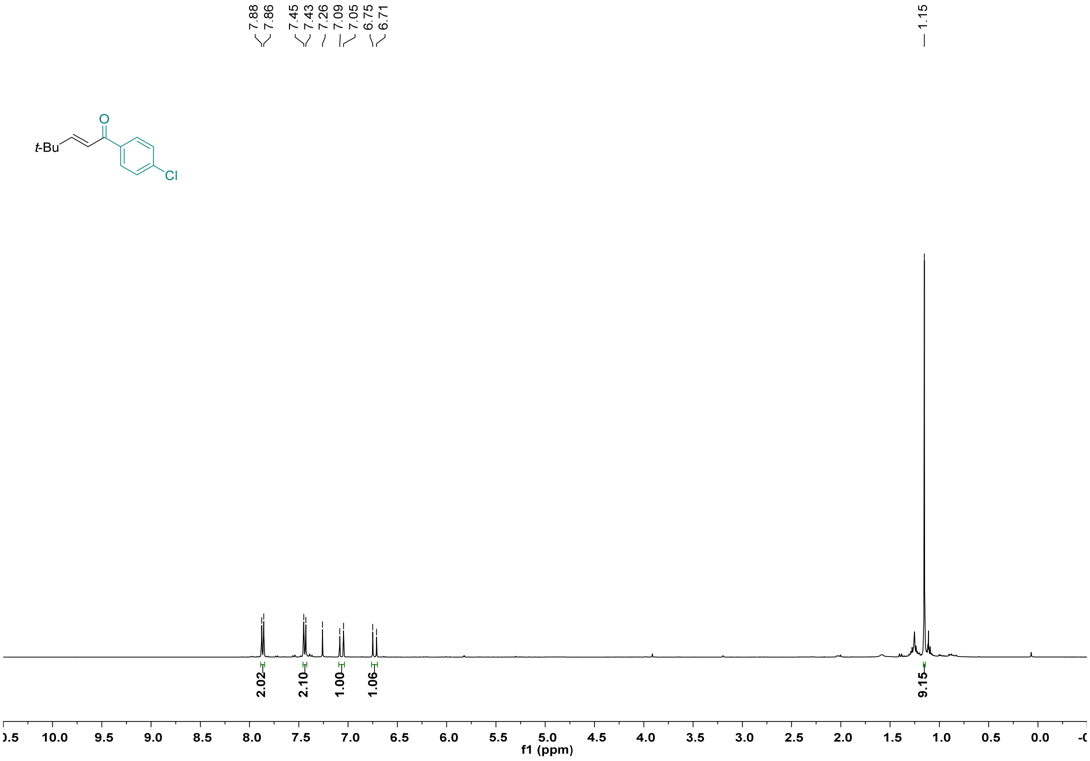

^{1}H NMR (400 MHz, $CDCl_3$) of compound **15**

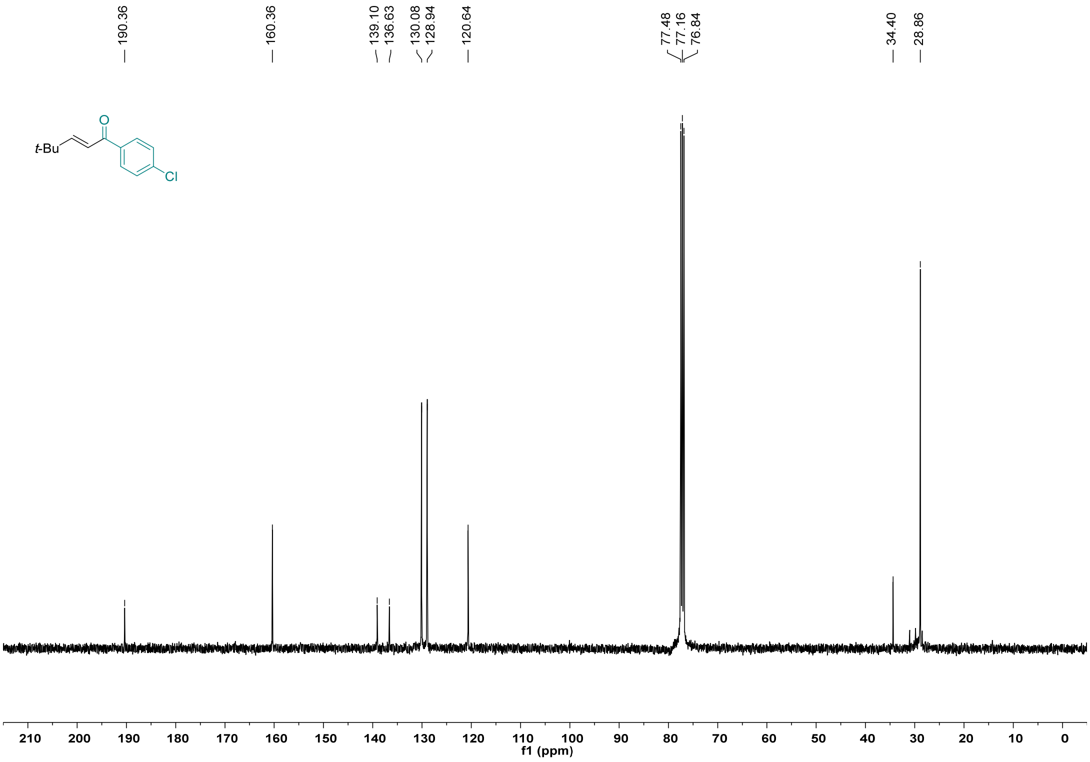

^{13}C NMR (100 MHz, $CDCl_3$) of compound **15**

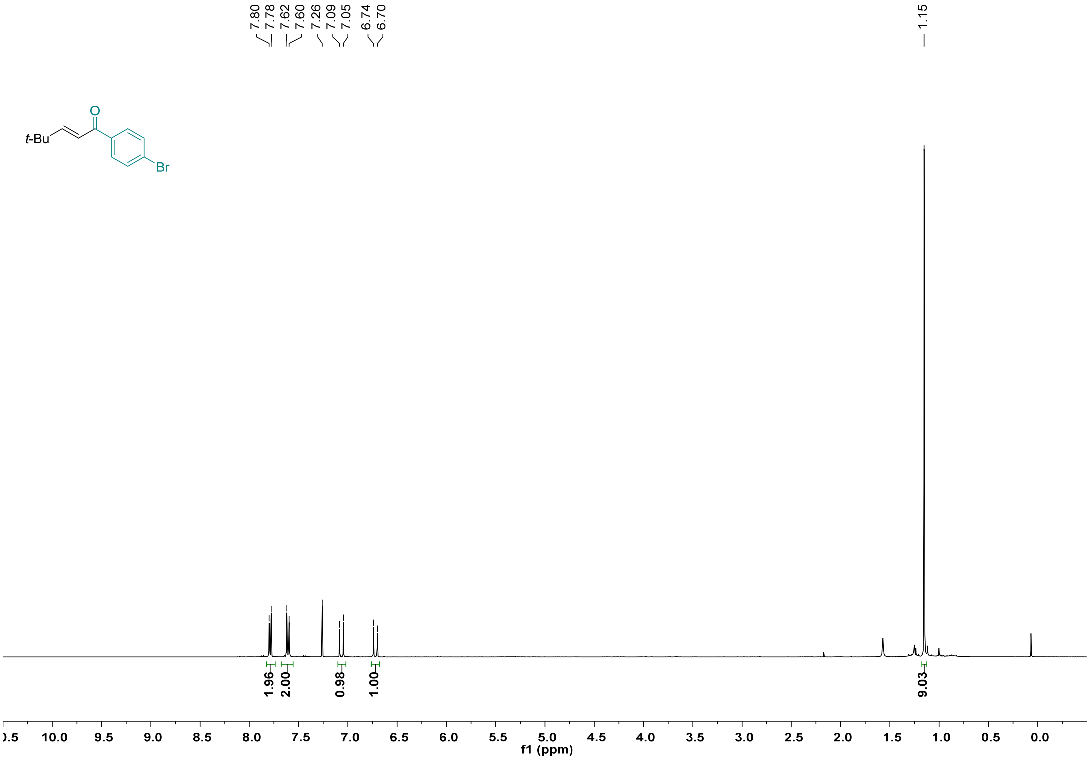

^{1}H NMR (400 MHz, $CDCl_3$) of compound **16**

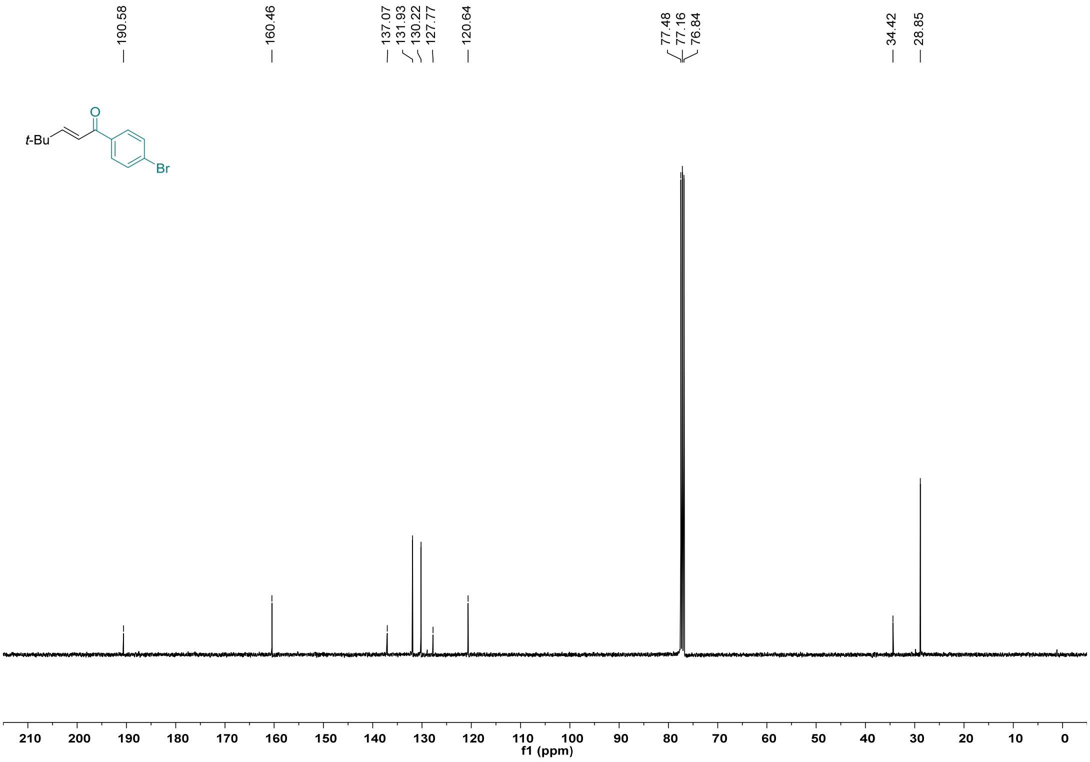

^{13}C NMR (100 MHz, $CDCl_3$) of compound **16**

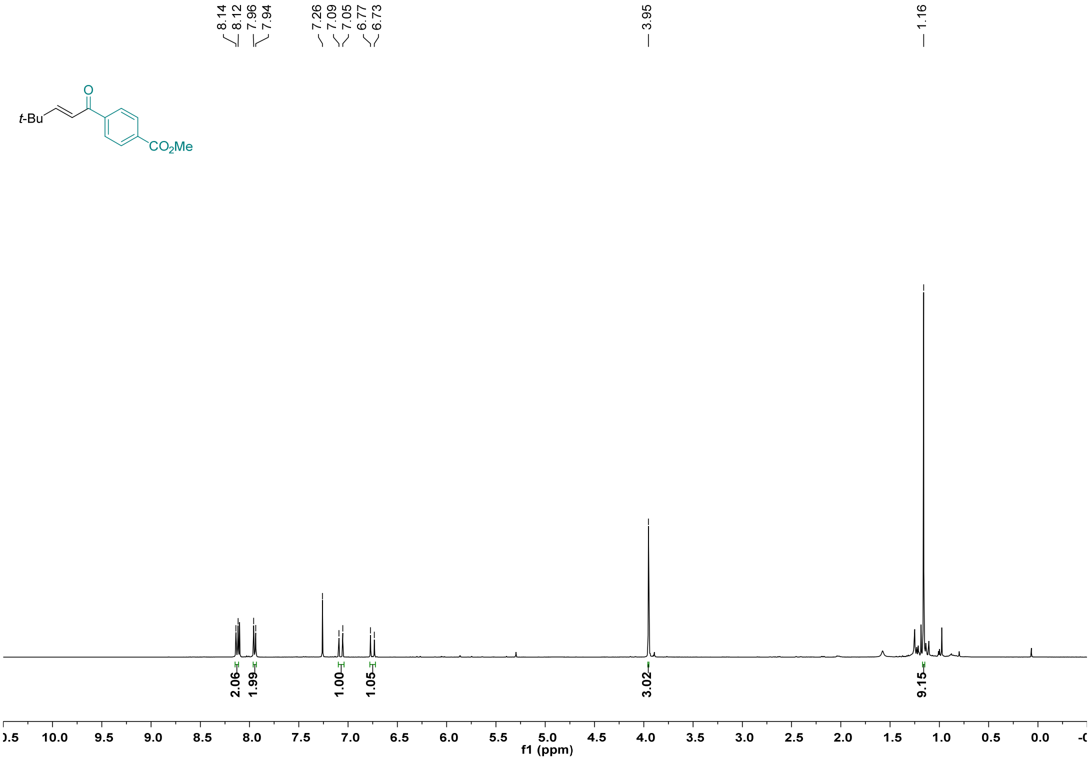

^{1}H NMR (400 MHz, $CDCl_3$) of compound **17**

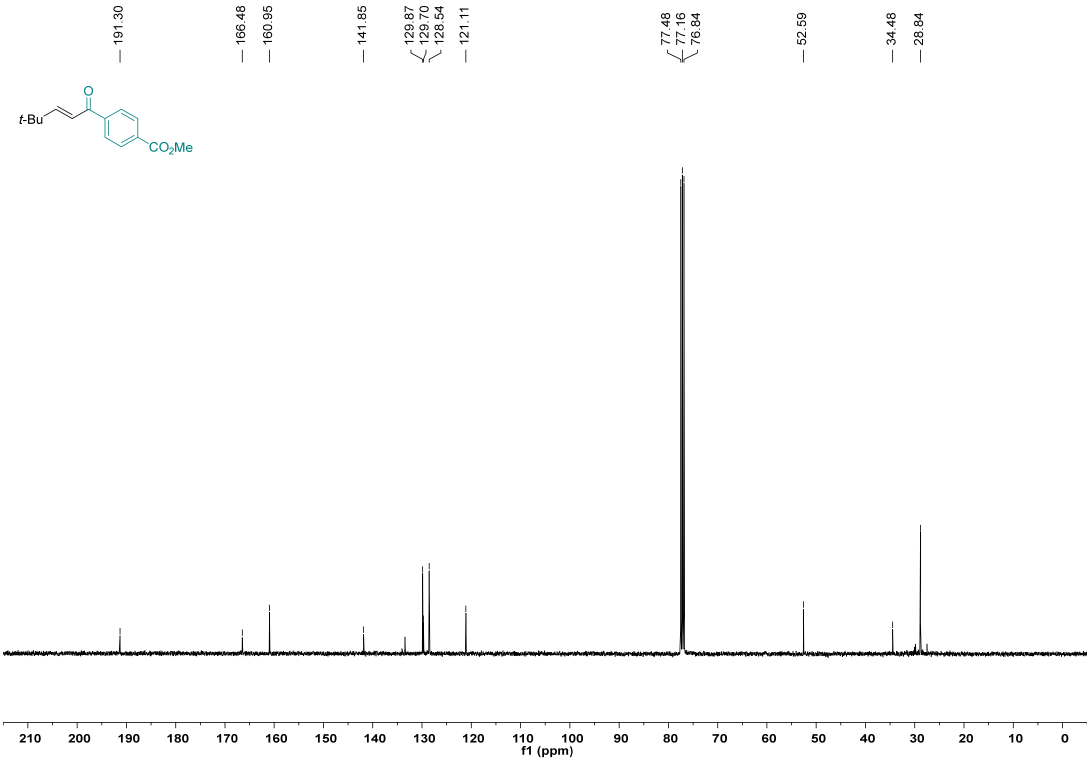

^{13}C NMR (100 MHz, $CDCl_3$) of compound **17**

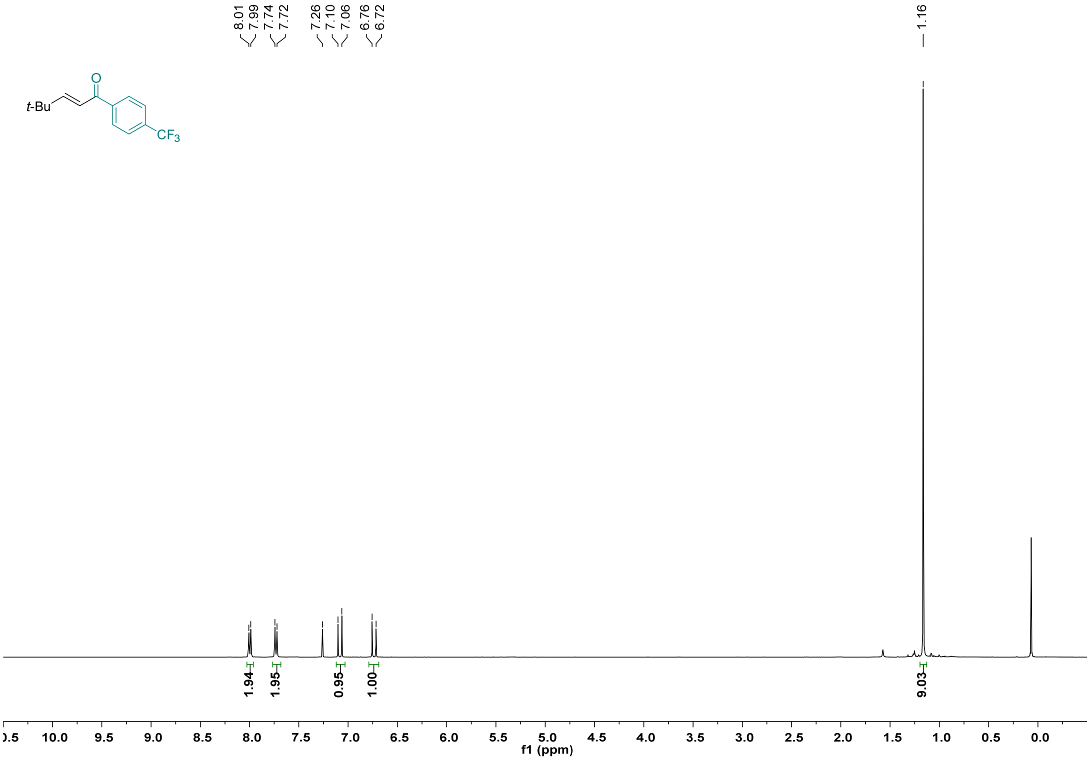

^{1}H NMR (400 MHz, $CDCl_3$) of compound **18**

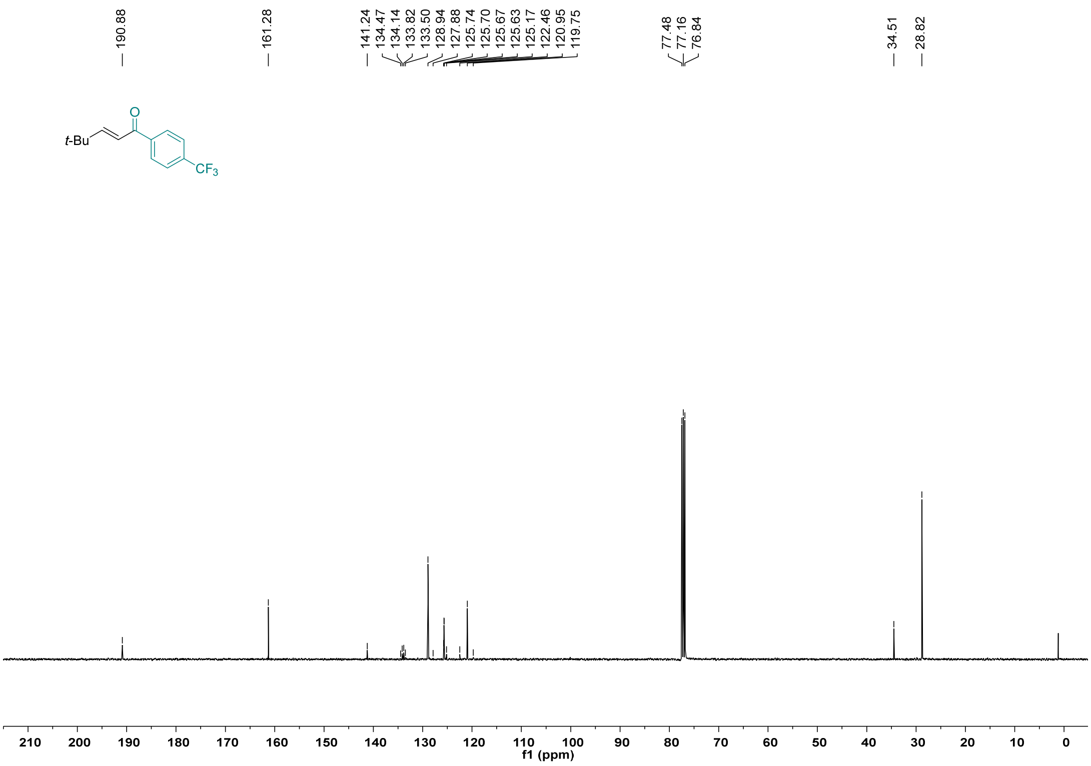

^{13}C NMR (100 MHz, $CDCl_3$) of compound **18**

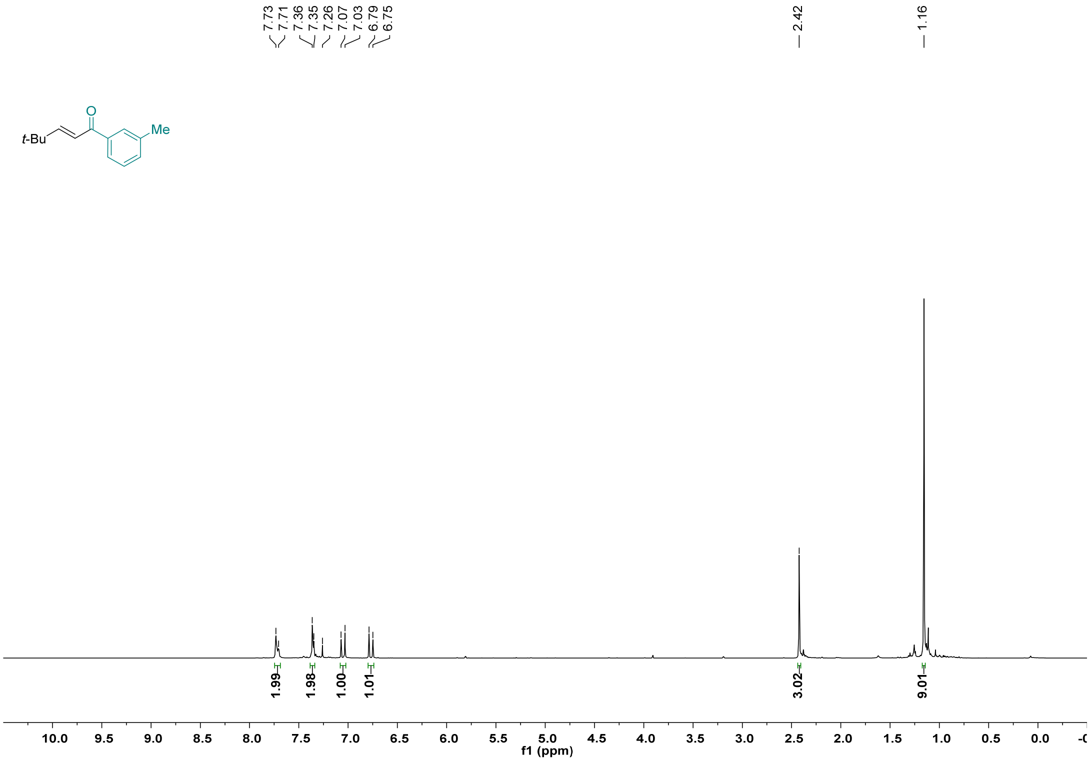

^{1}H NMR (400 MHz, $CDCl_3$) of compound **19**

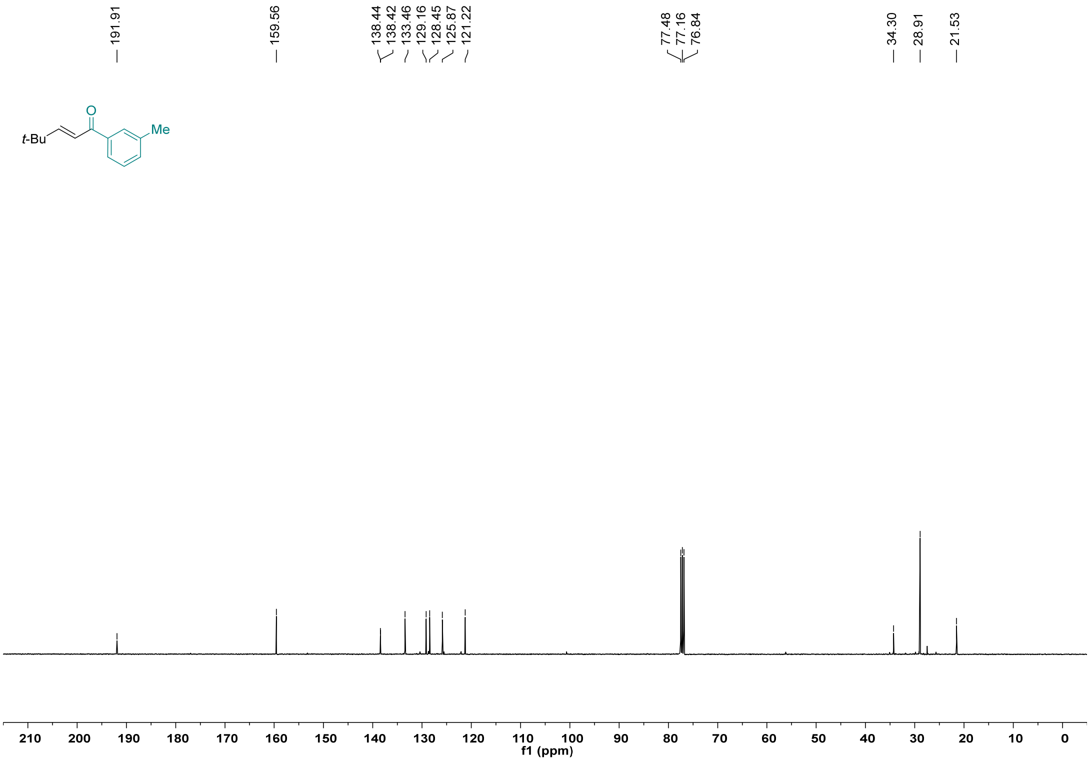

^{13}C NMR (100 MHz, CDCl$_3$) of compound **19**

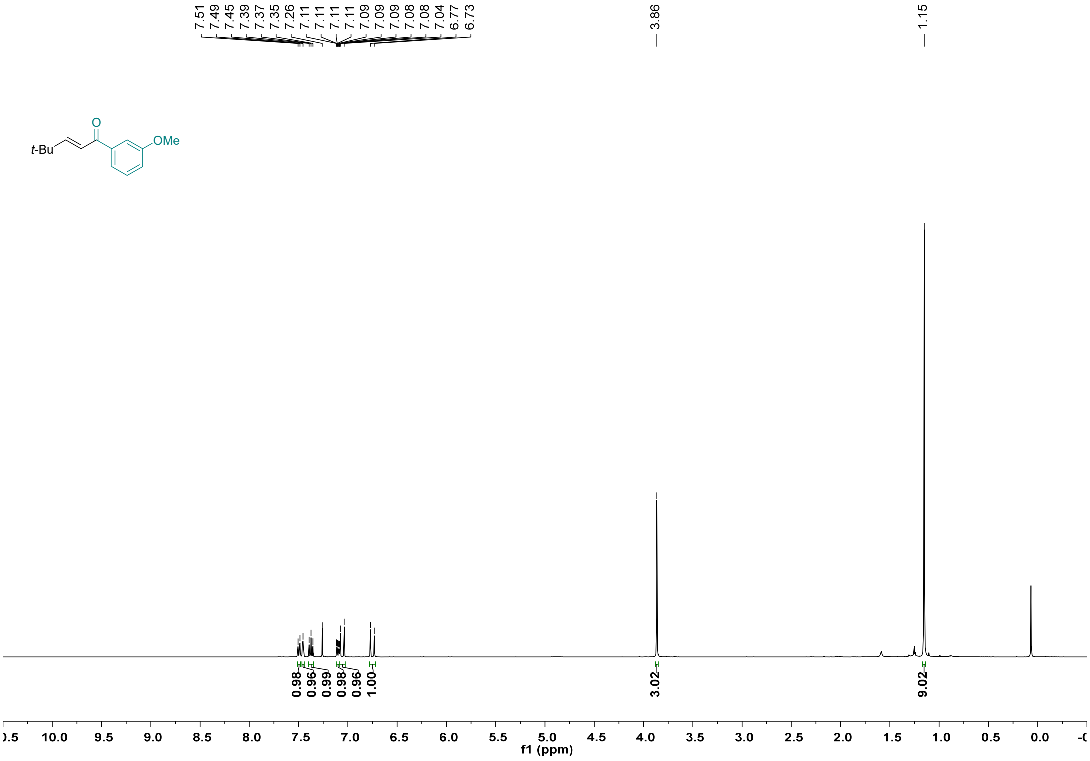

^{1}H NMR (400 MHz, CDCl$_3$) of compound **20**

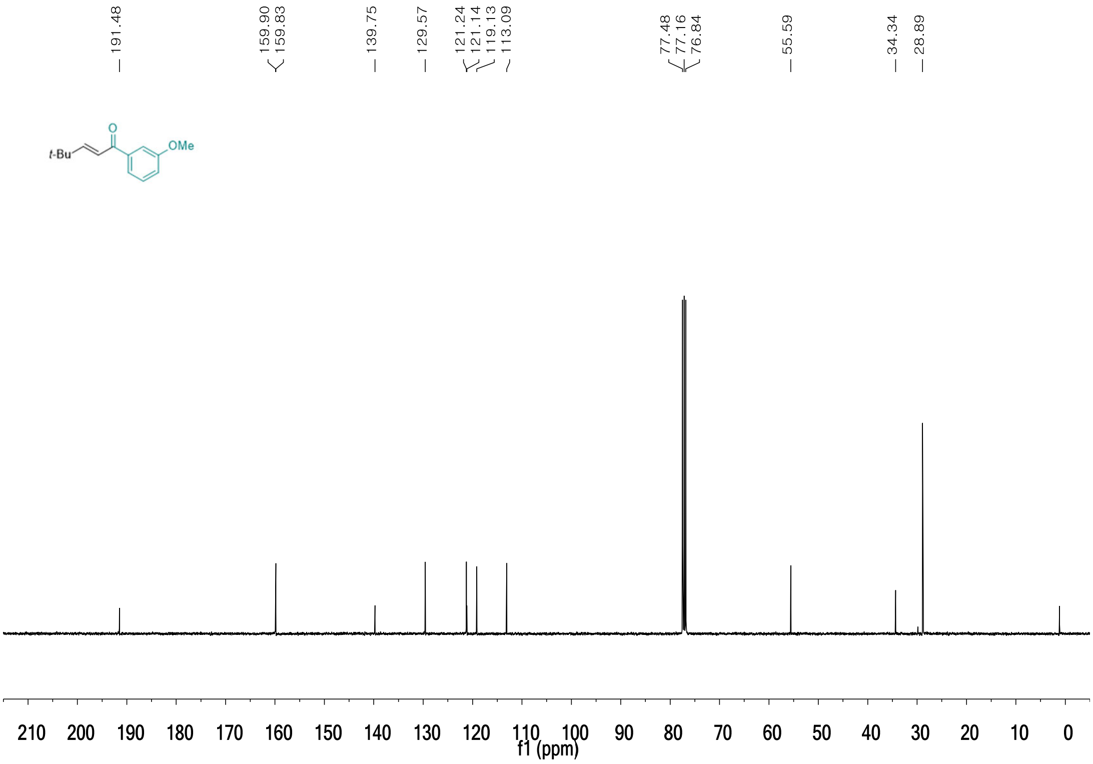

^{13}C NMR (100 MHz, $CDCl_3$) of compound **20**

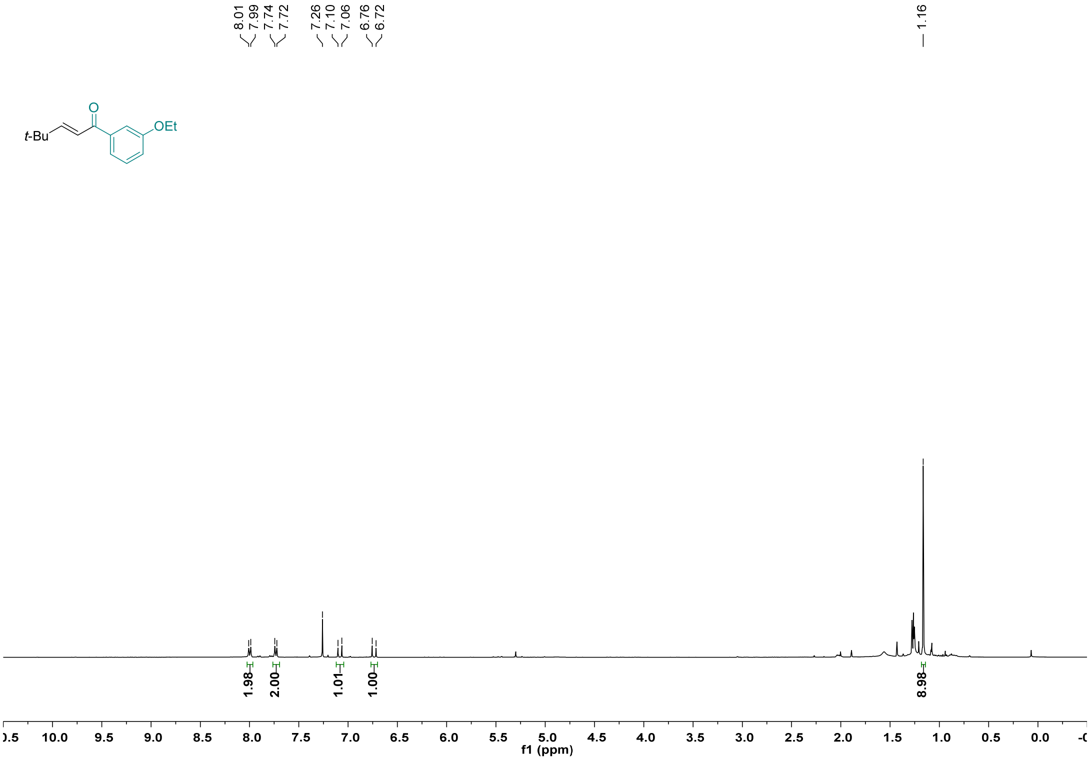

^{1}H NMR (400 MHz, $CDCl_3$) of compound **21**

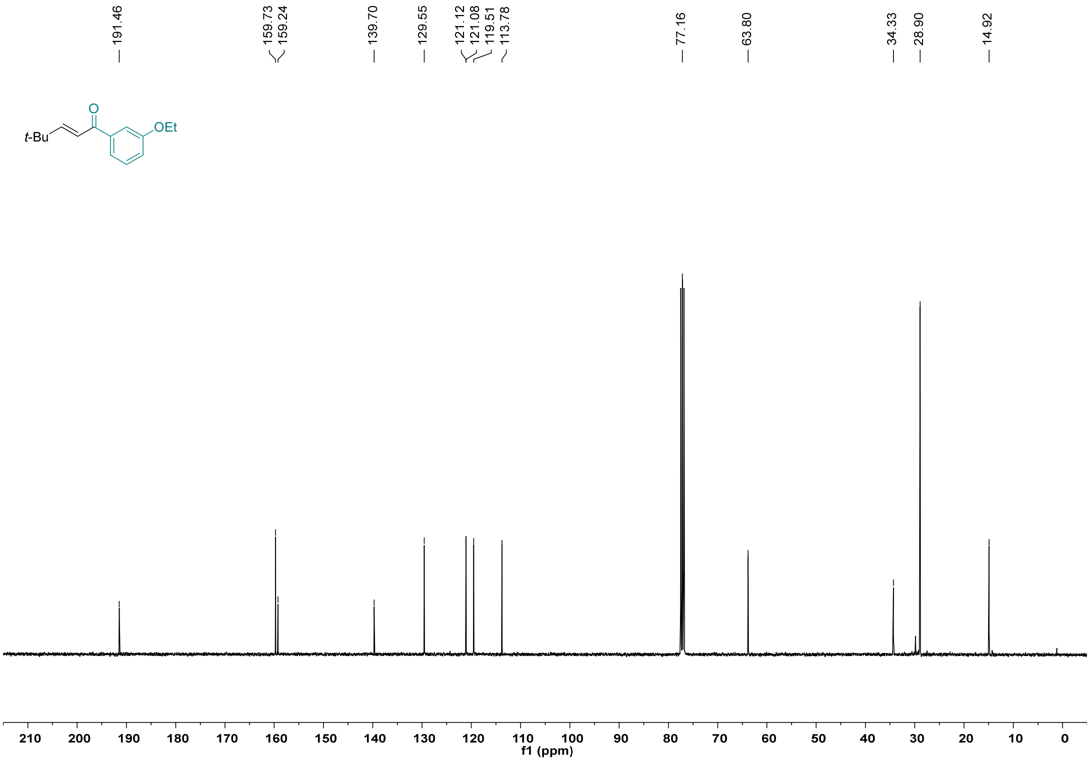

^{13}C NMR (100 MHz, $CDCl_3$) of compound **21**

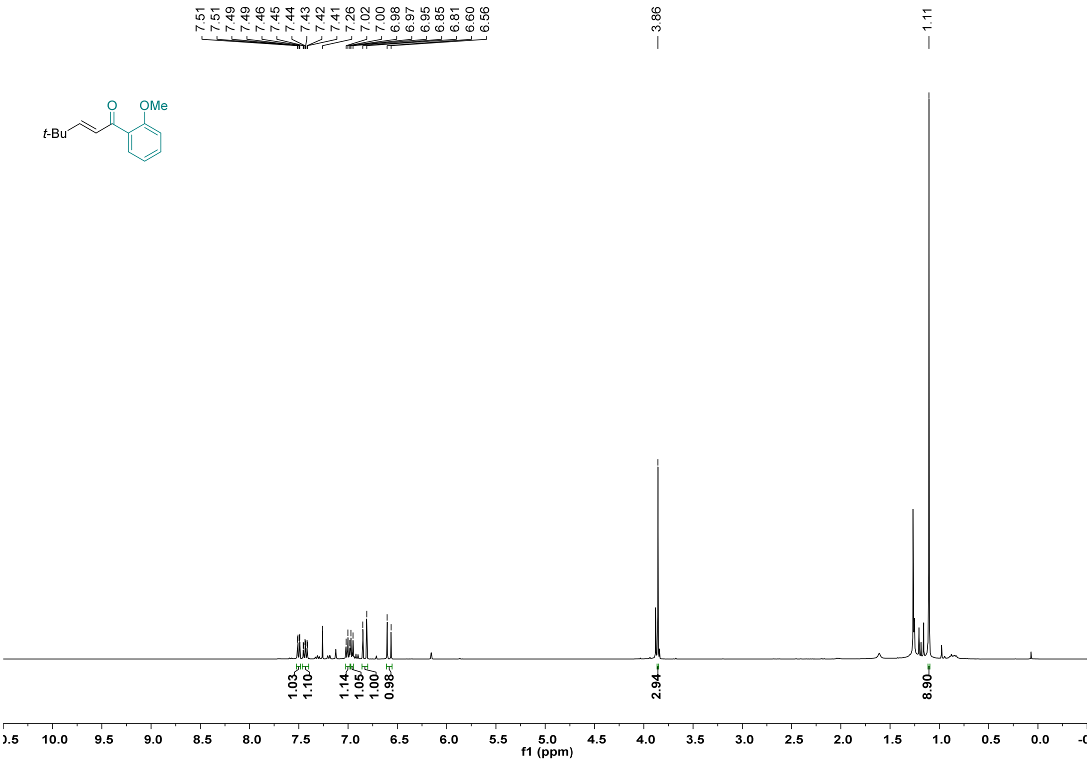

^{1}H NMR (400 MHz, $CDCl_3$) of compound **22**

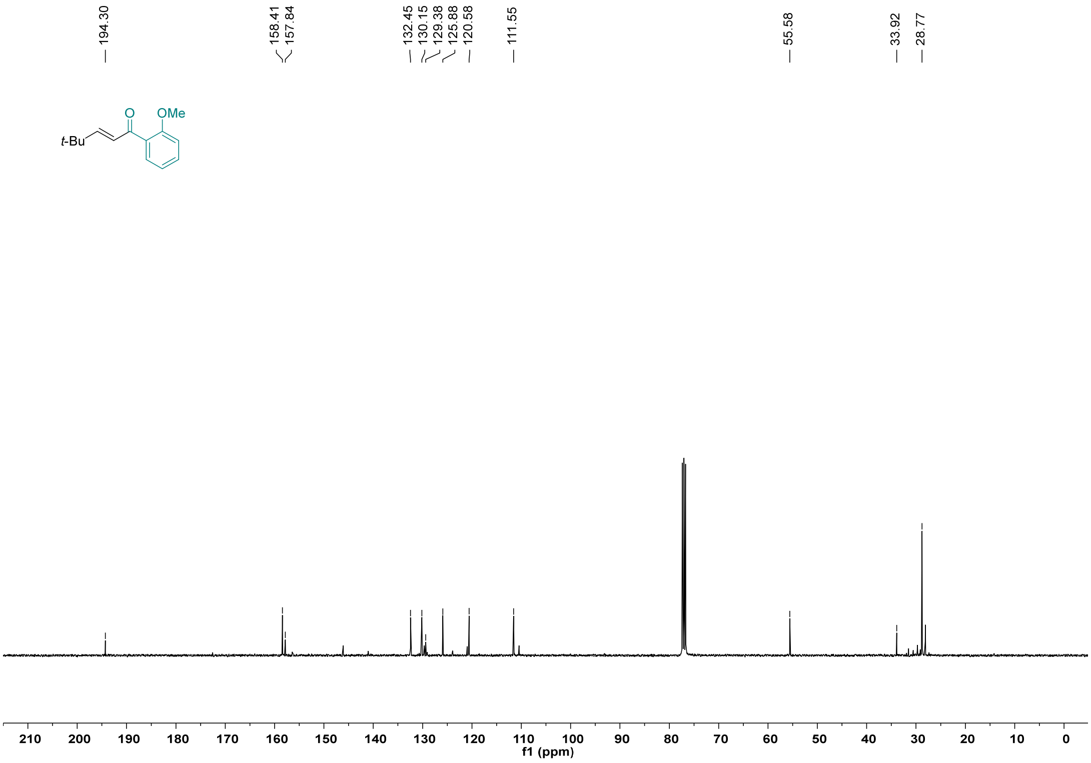

^{13}C NMR (100 MHz, CDCl$_3$) of compound **22**

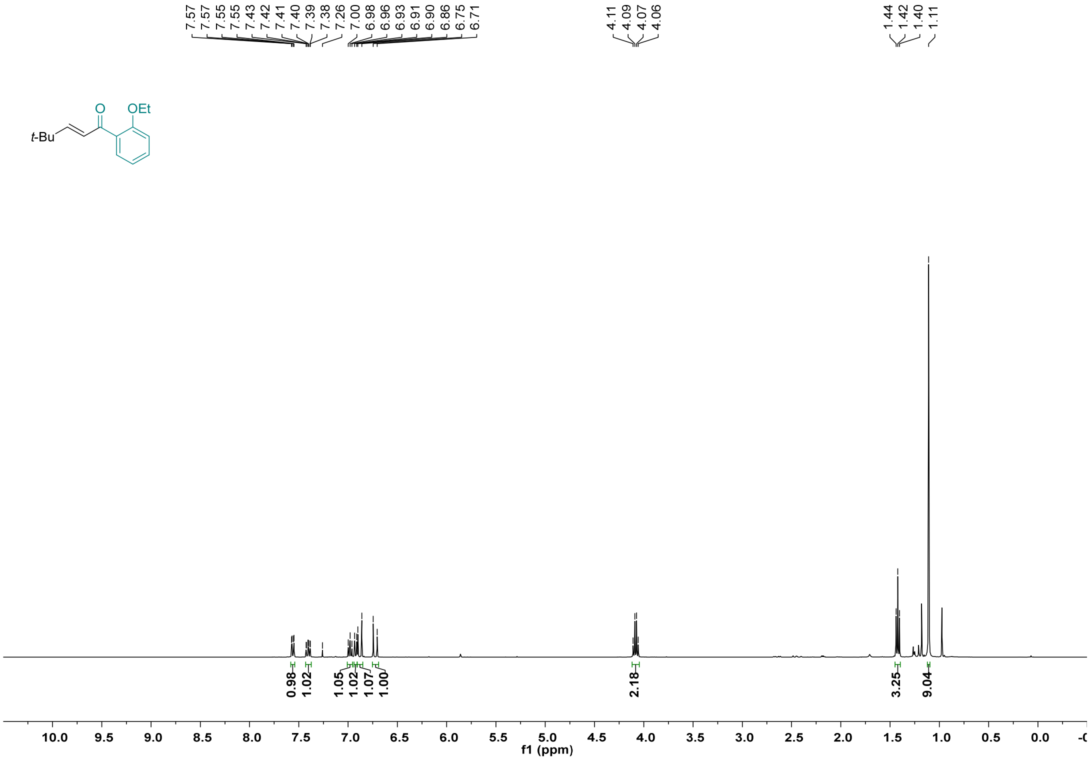

^{1}H NMR (400 MHz, CDCl$_3$) of compound **23**

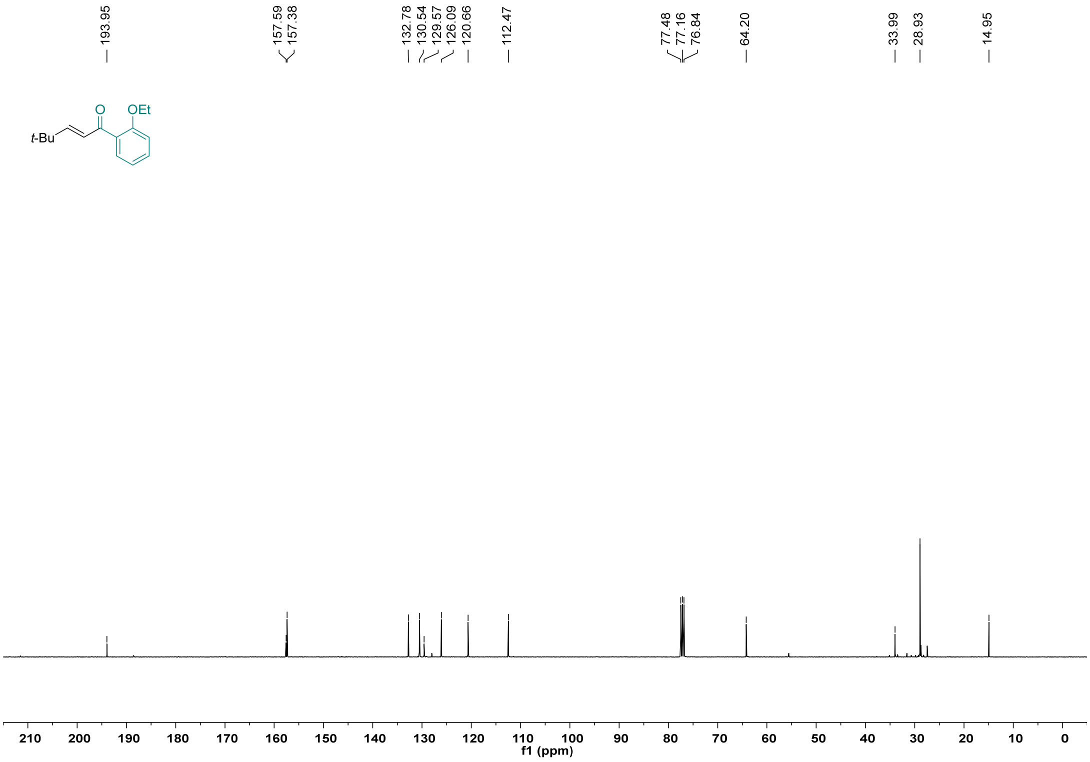

^{13}C NMR (100 MHz, CDCl$_3$) of compound **23**

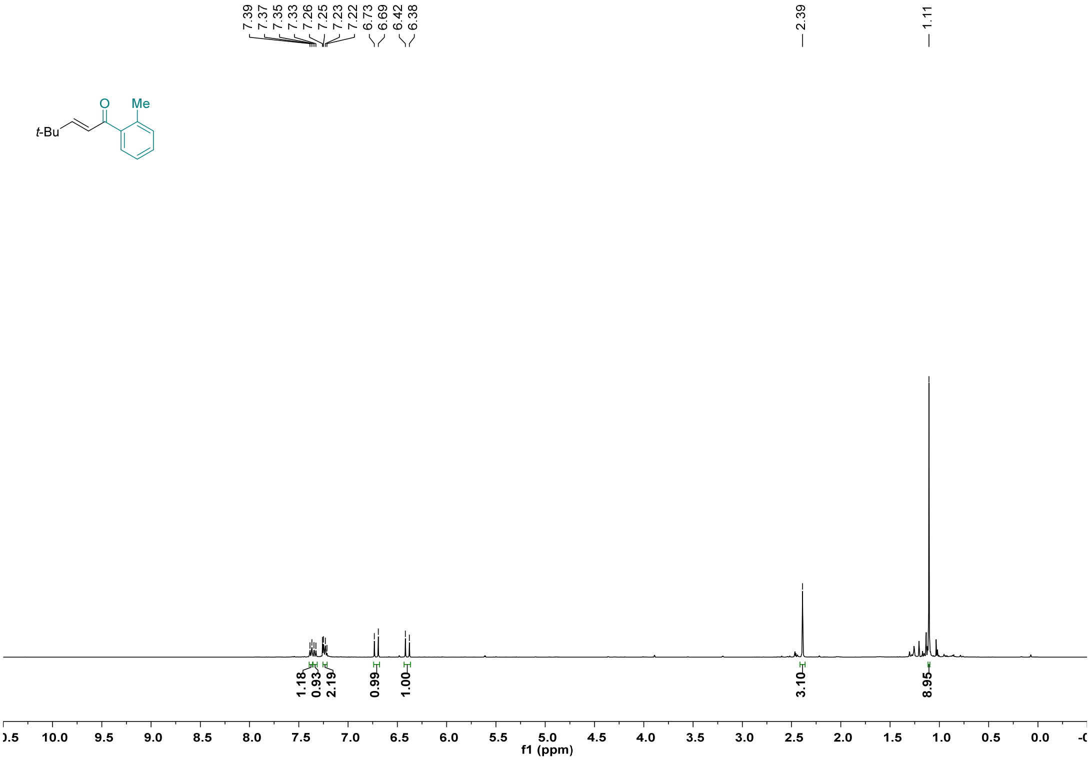

^{1}H NMR (400 MHz, CDCl$_3$) of compound **24**

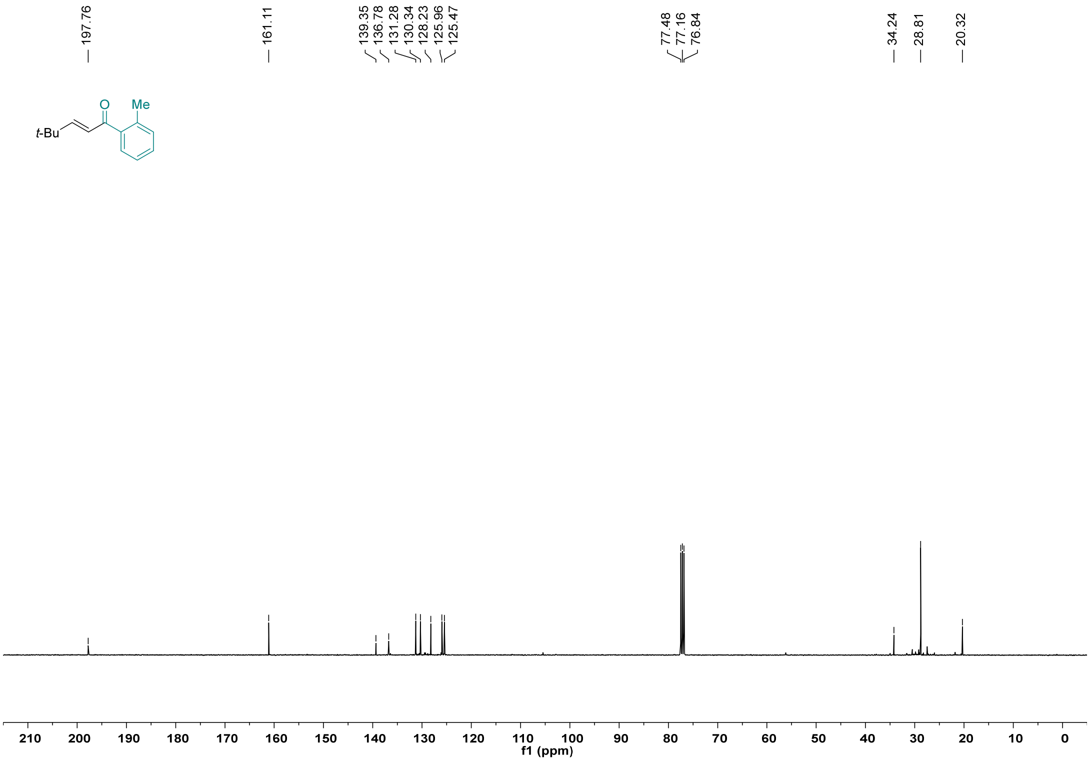

^{13}C NMR (100 MHz, $CDCl_3$) of compound **24**

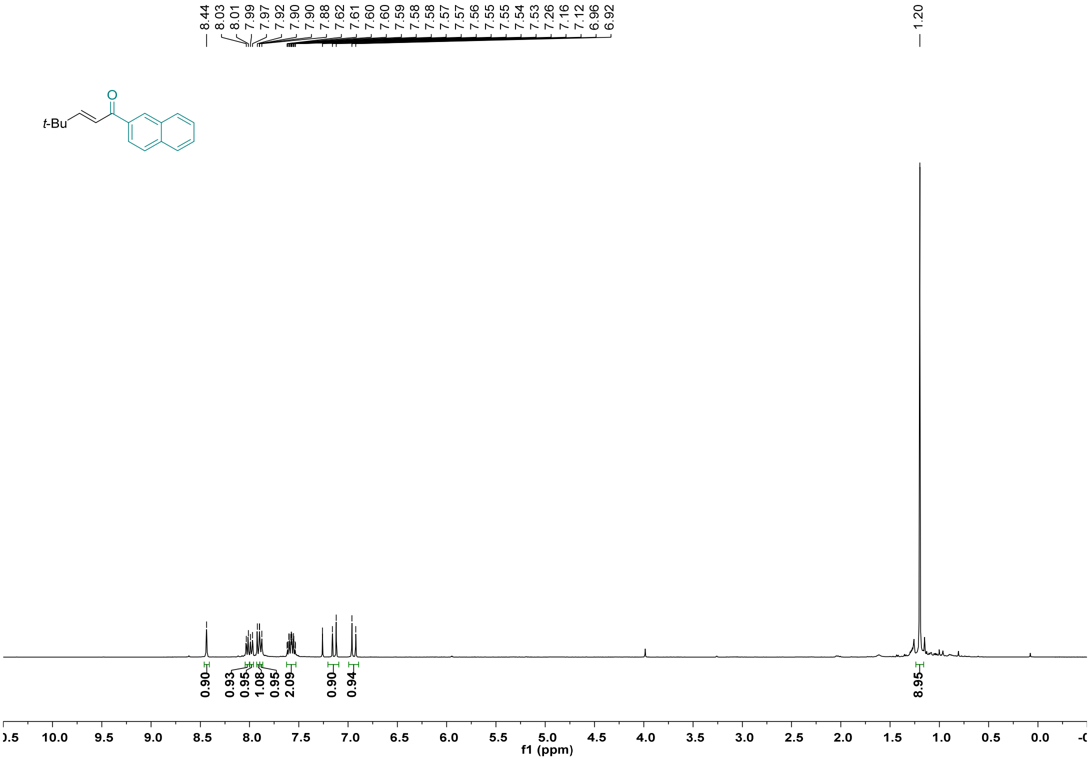

^{1}H NMR (400 MHz, $CDCl_3$) of compound **25**

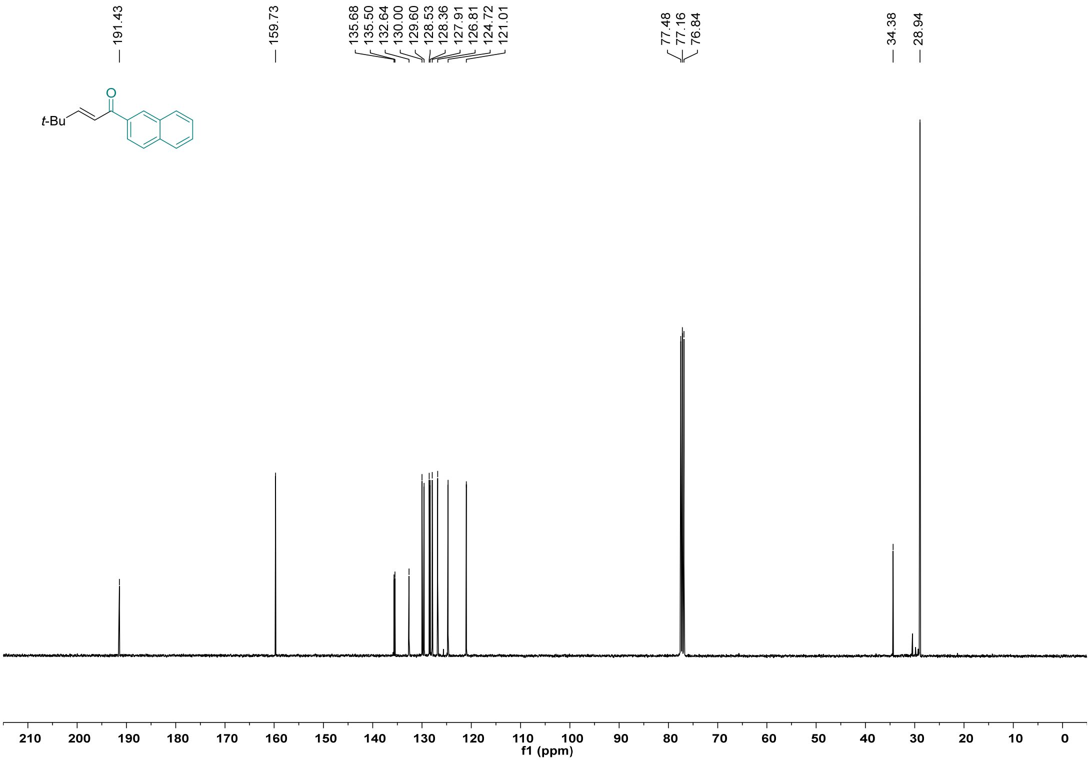

^{13}C NMR (100 MHz, CDCl$_3$) of compound **25**

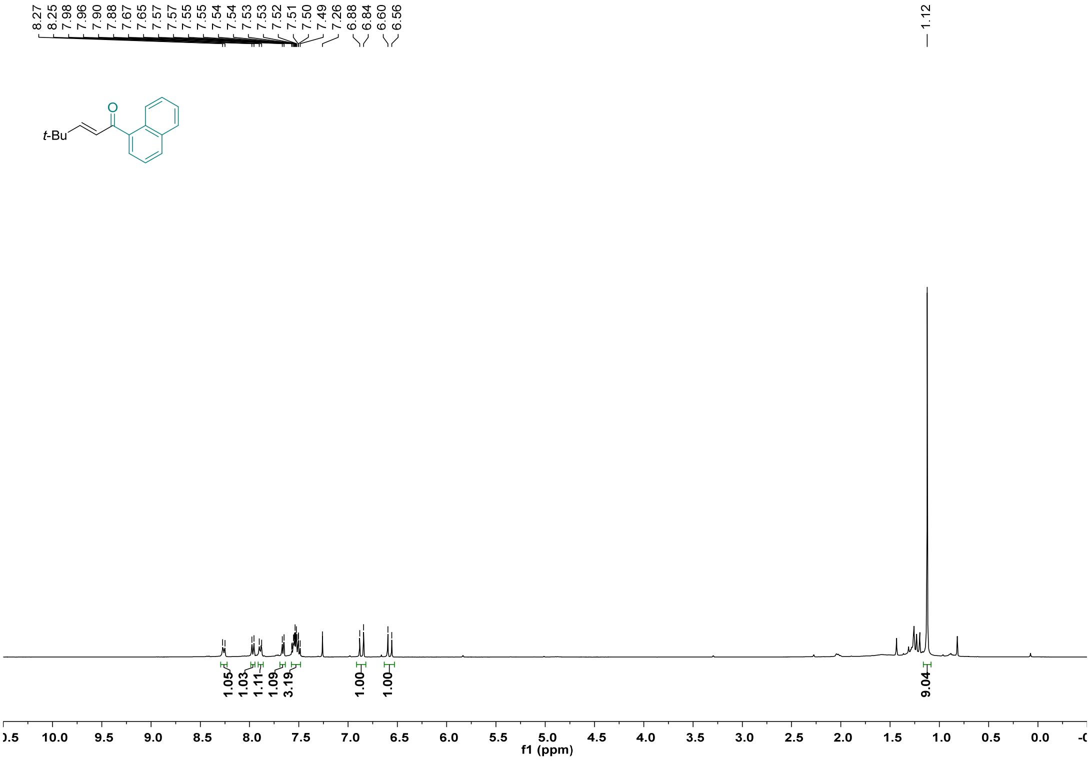

^{1}H NMR (400 MHz, CDCl$_3$) of compound **26**

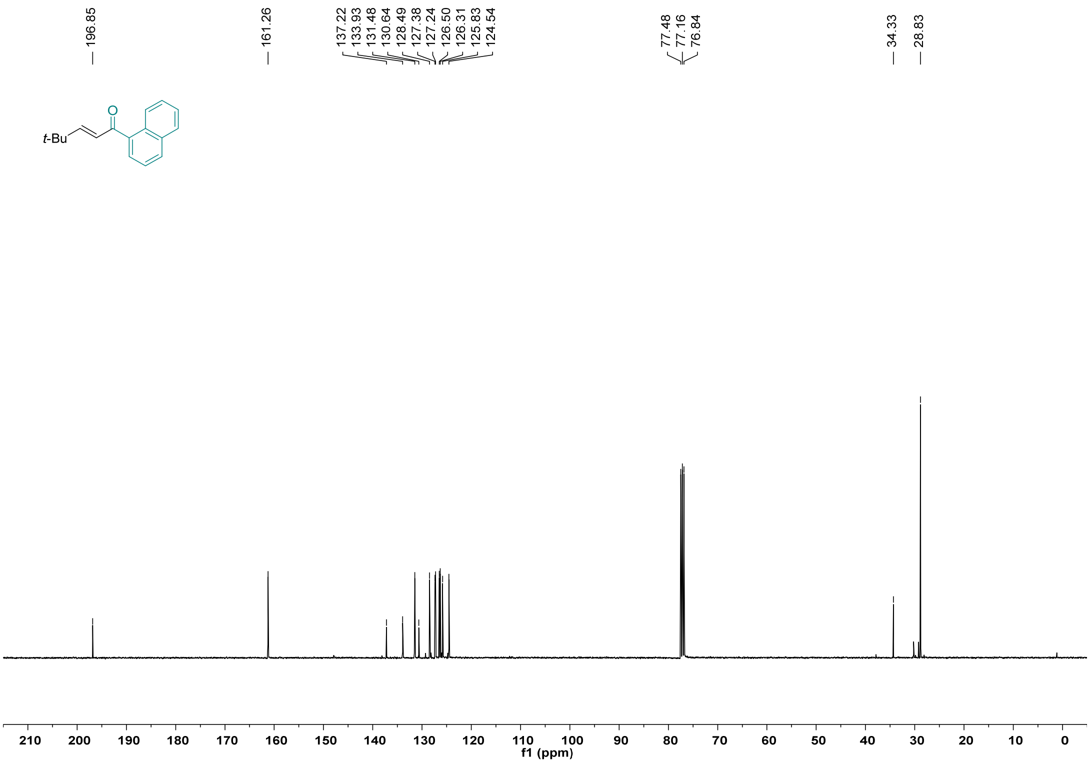

^{13}C NMR (100 MHz, $CDCl_3$) of compound **26**

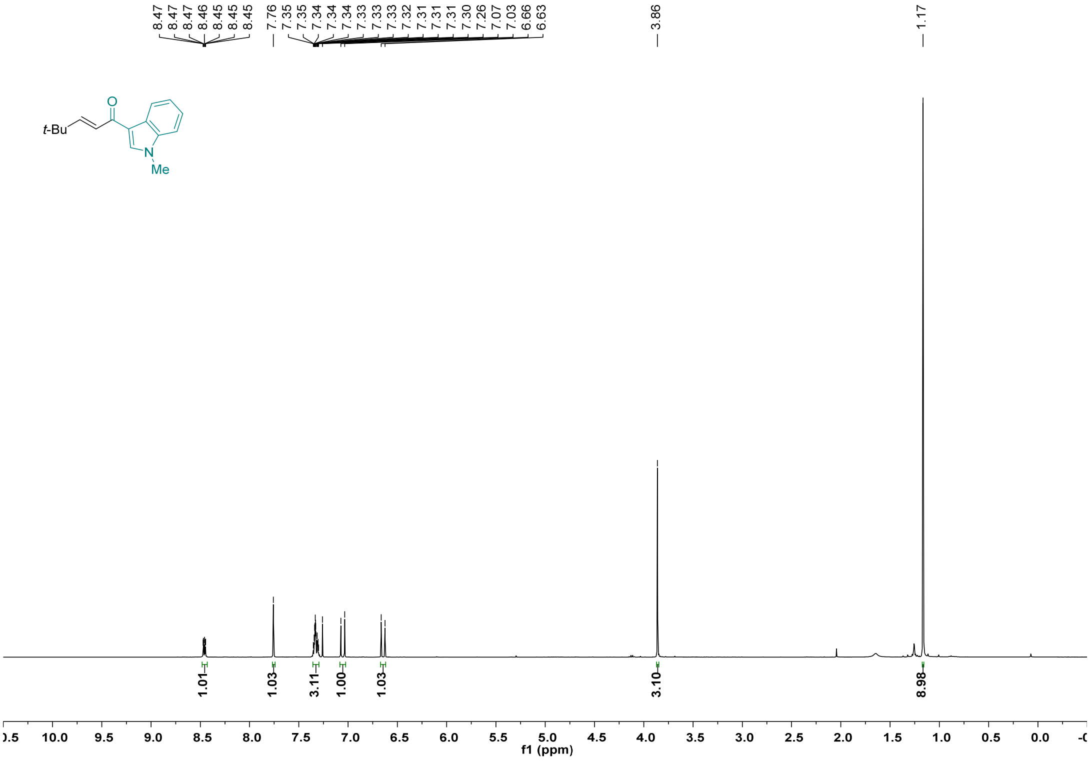

^{1}H NMR (400 MHz, $CDCl_3$) of compound **27**

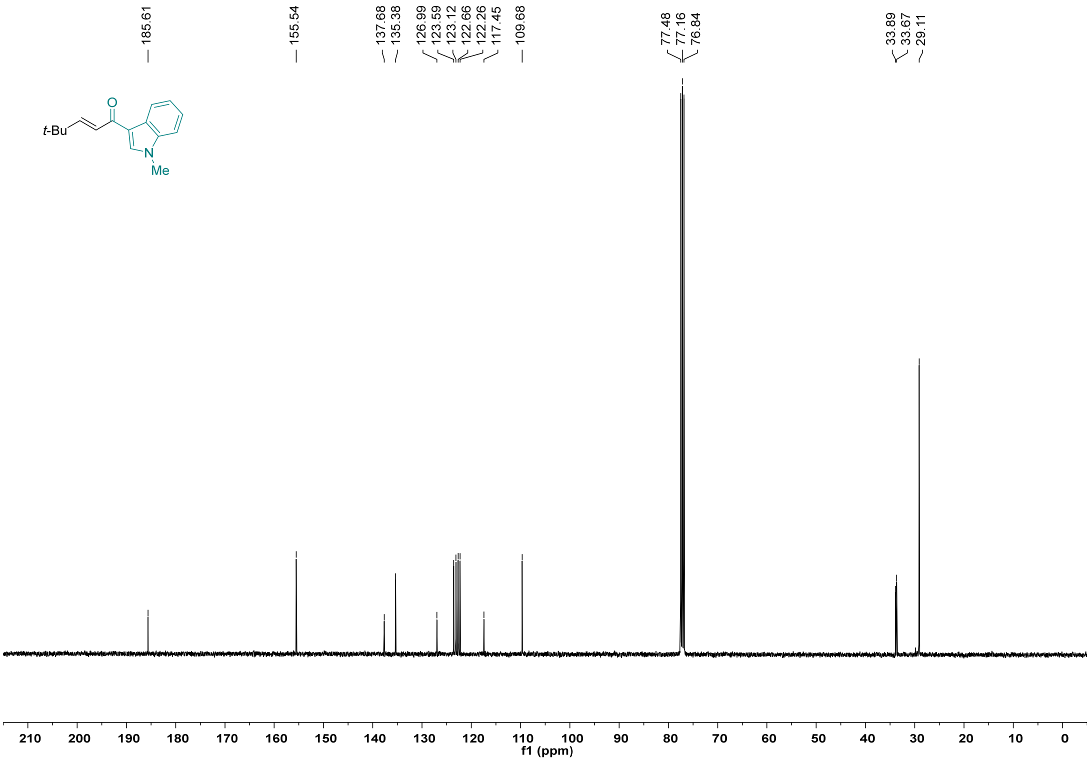

^{13}C NMR (100 MHz, $CDCl_3$) of compound **27**

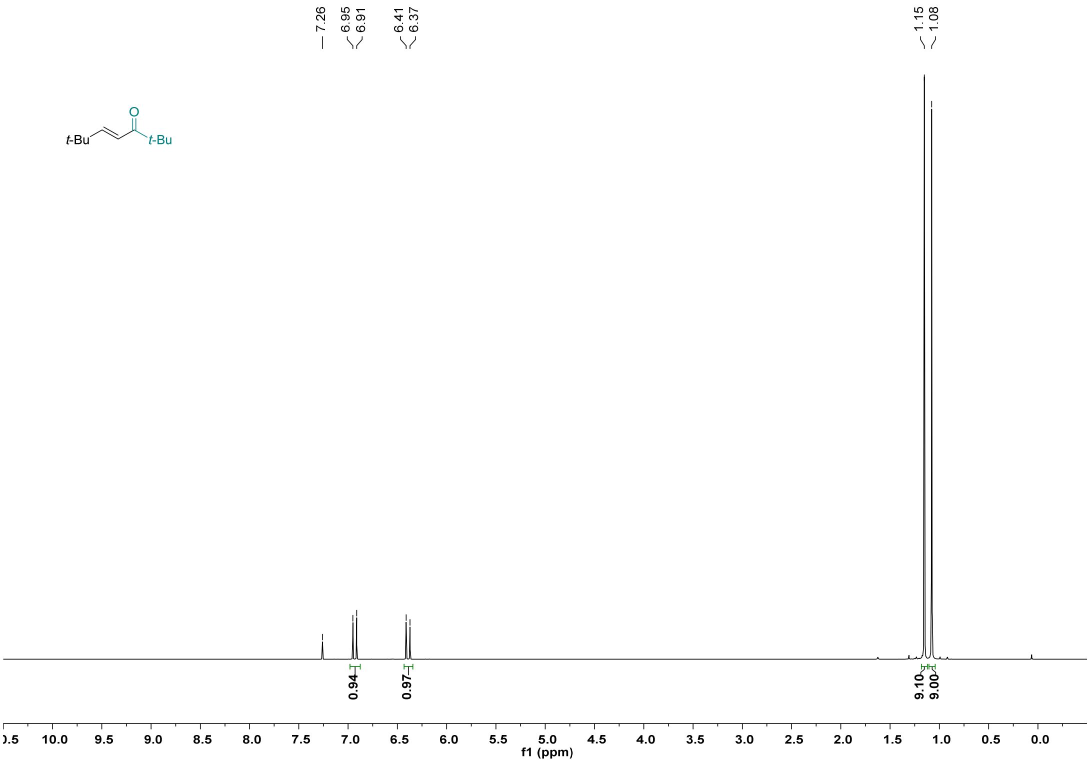

^{1}H NMR (400 MHz, $CDCl_3$) of compound **28**

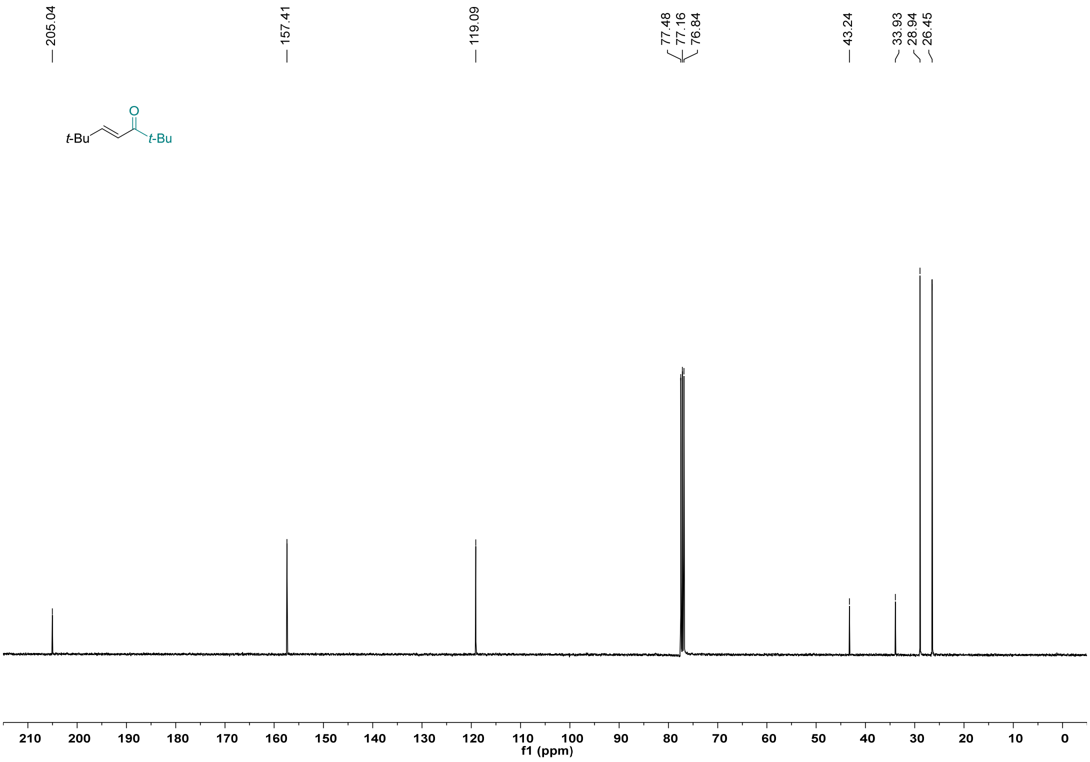

^{13}C NMR (100 MHz, $CDCl_3$) of compound **28**

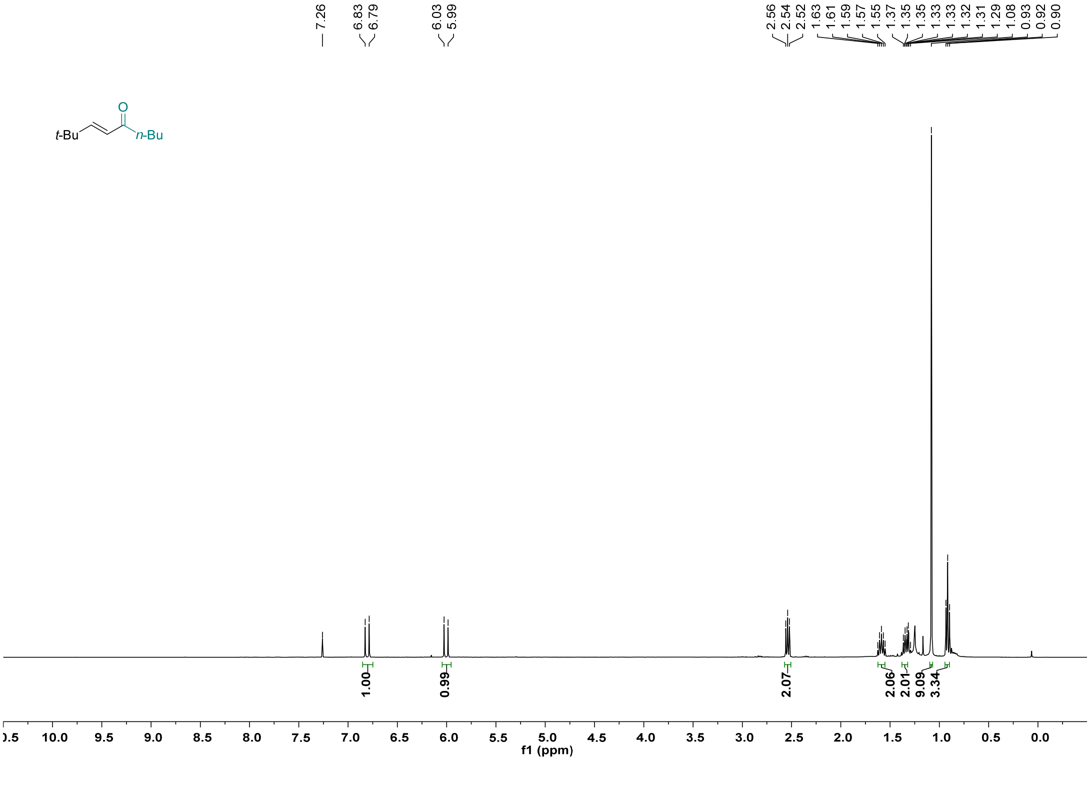

^{1}H NMR (400 MHz, $CDCl_3$) of compound **29**

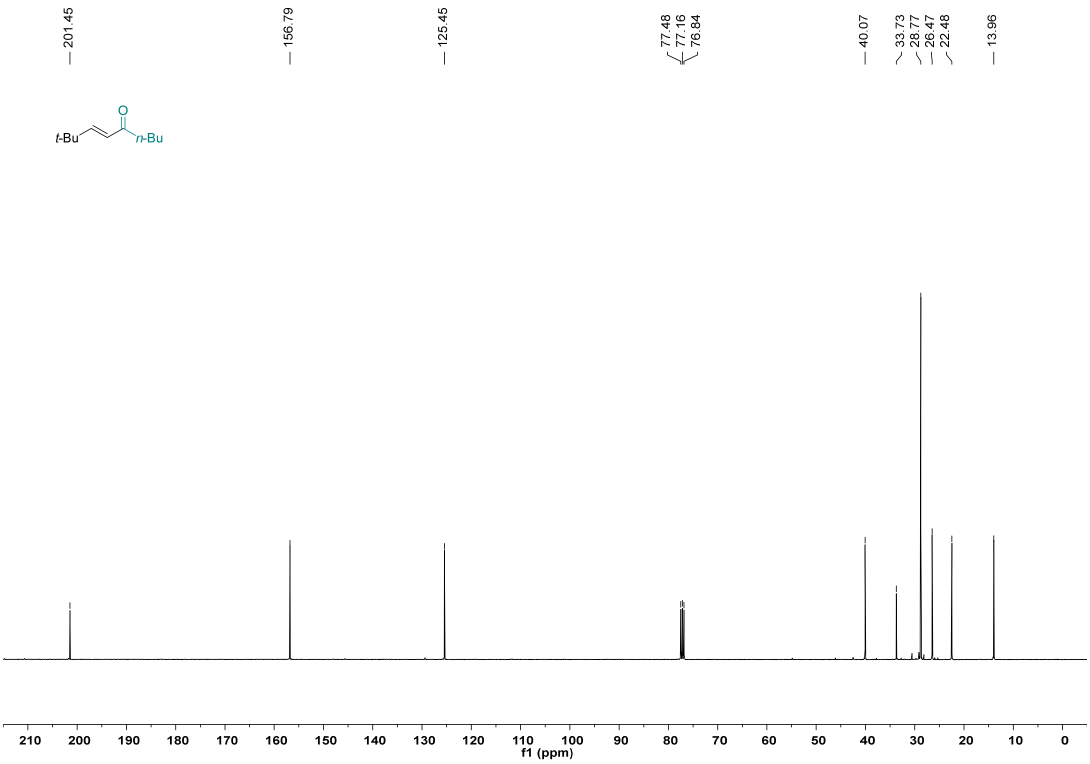

^{13}C NMR (100 MHz, $CDCl_3$) of compound **29**

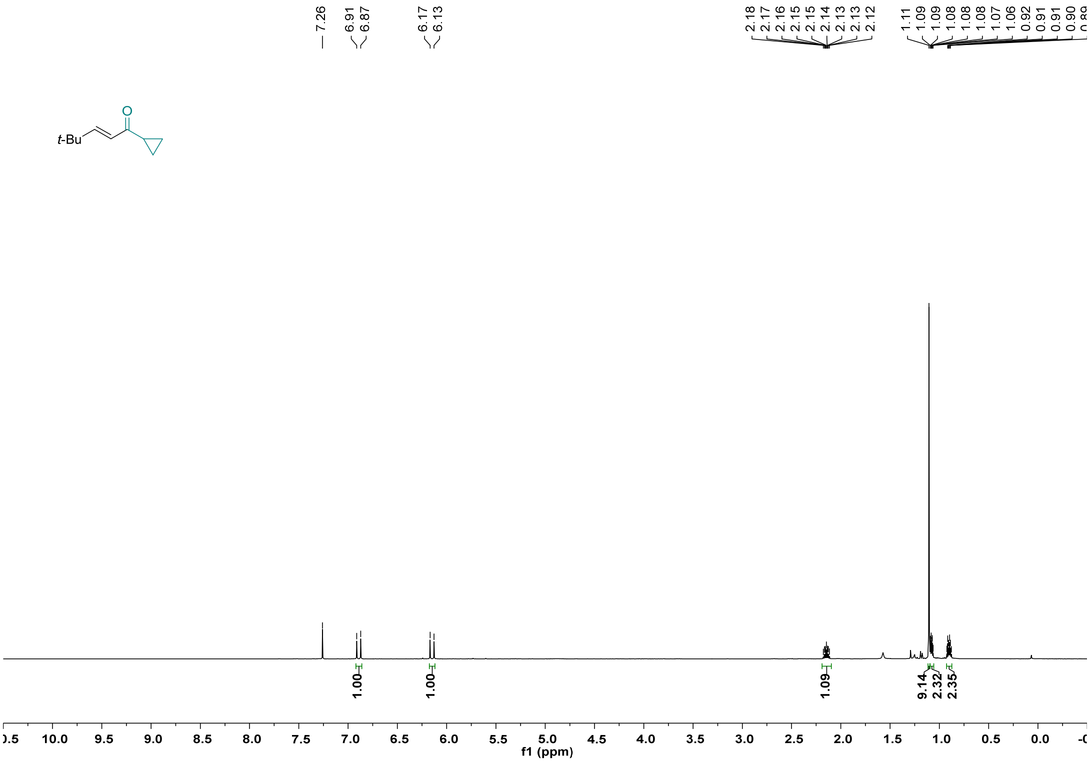

^{1}H NMR (400 MHz, $CDCl_3$) of compound **30**

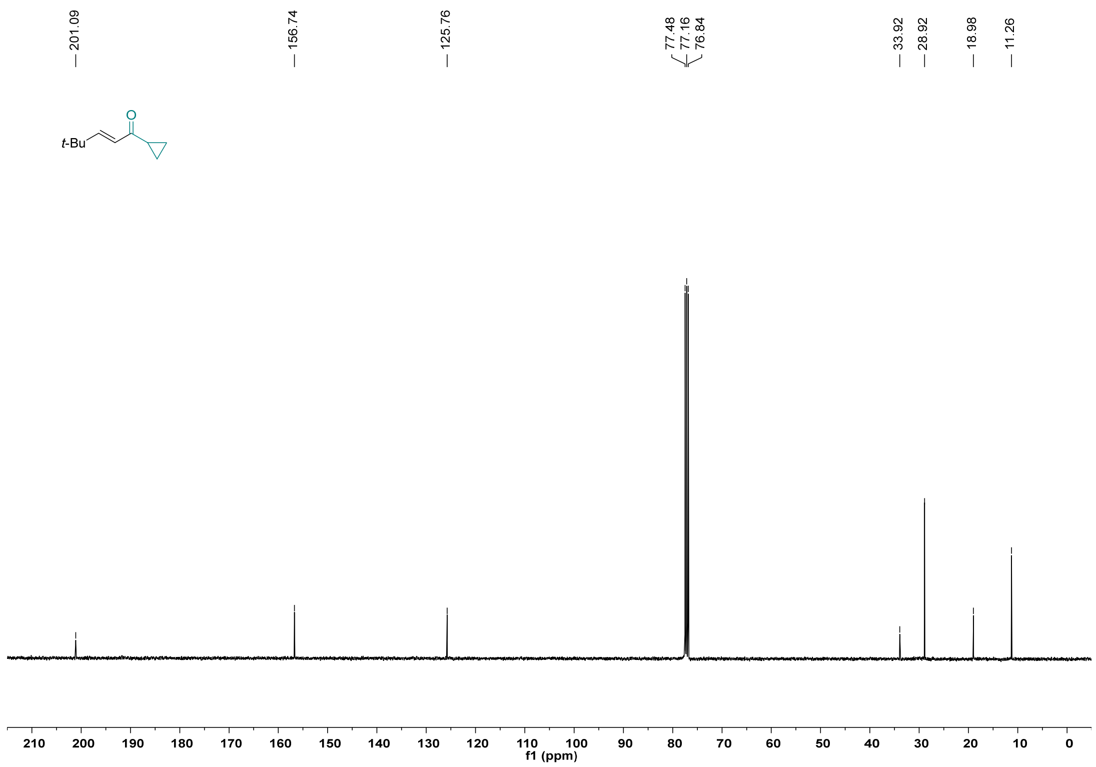

^{13}C NMR (100 MHz, $CDCl_3$) of compound **30**

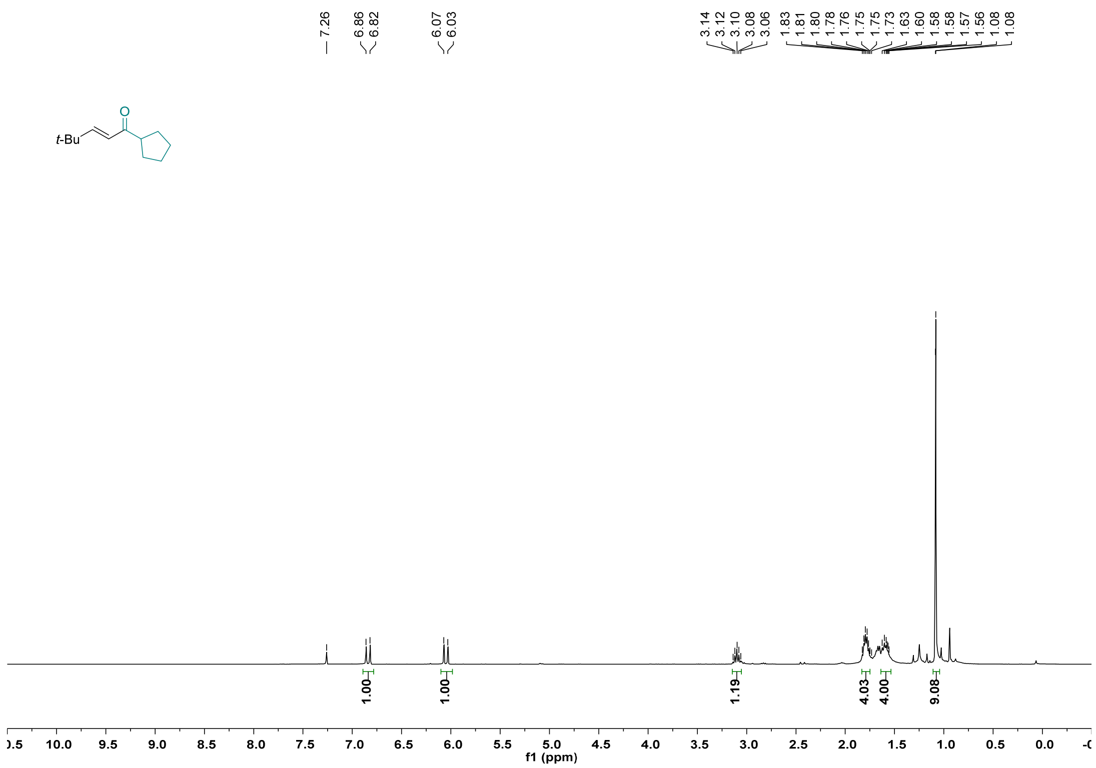

^{1}H NMR (400 MHz, $CDCl_3$) of compound **31**

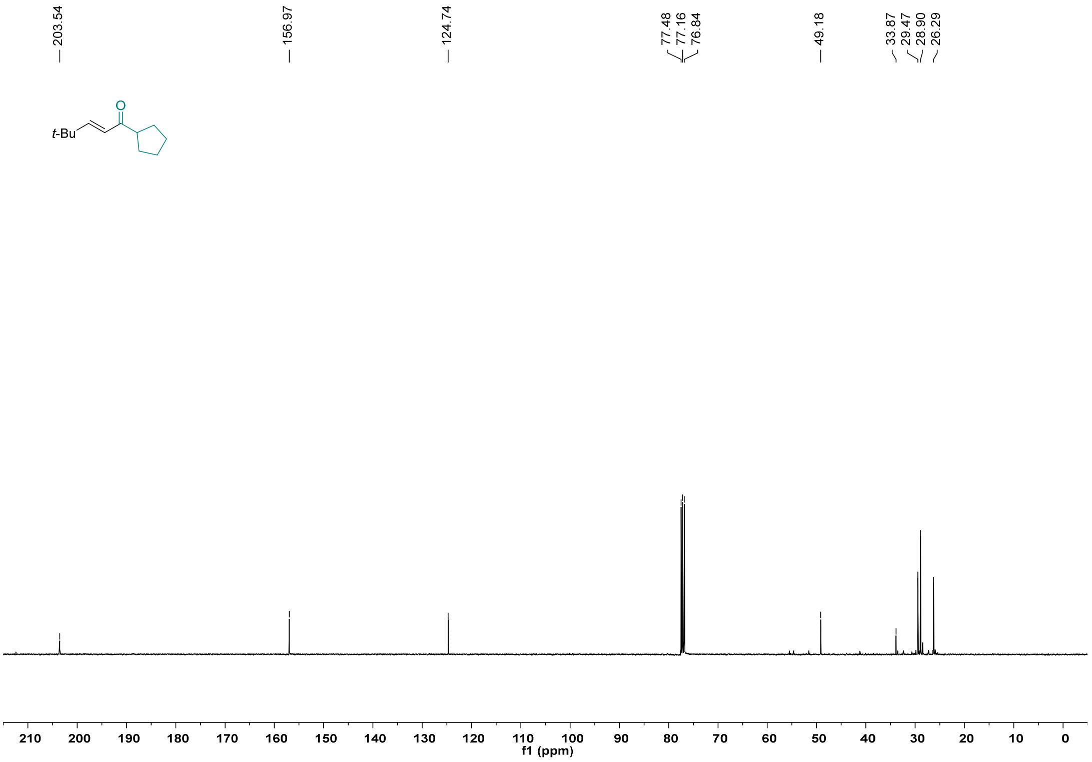

^{13}C NMR (100 MHz, CDCl$_3$) of compound **31**

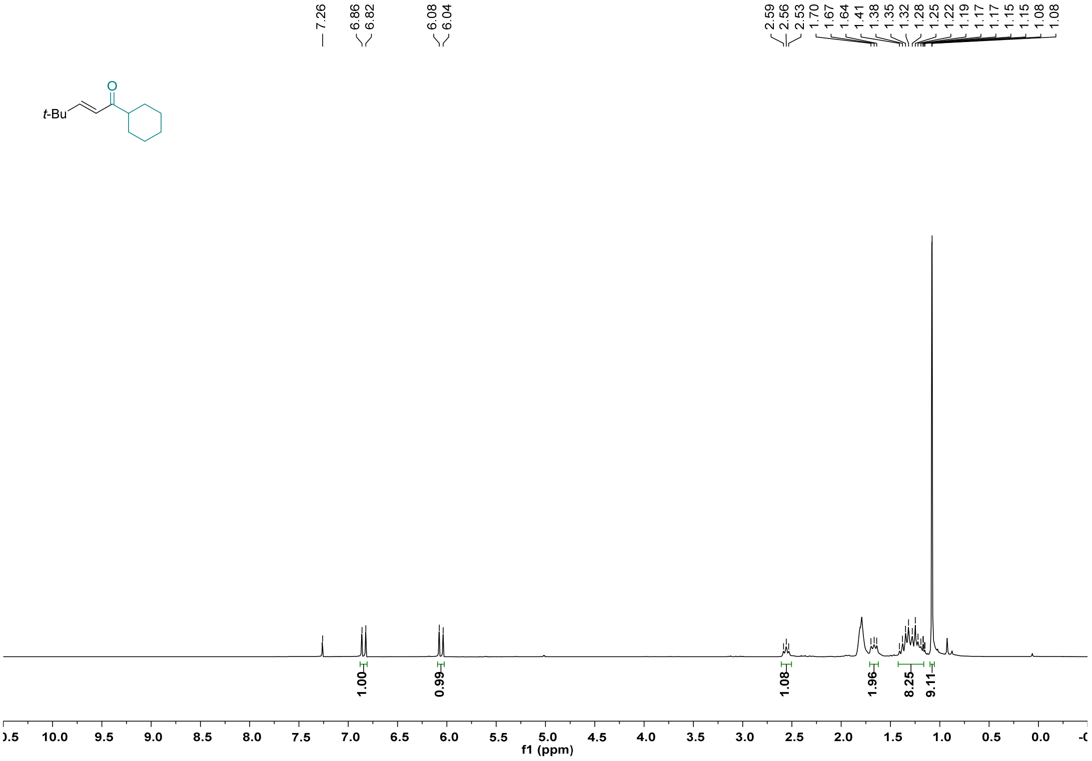

^{1}H NMR (400 MHz, CDCl$_3$) of compound **32**

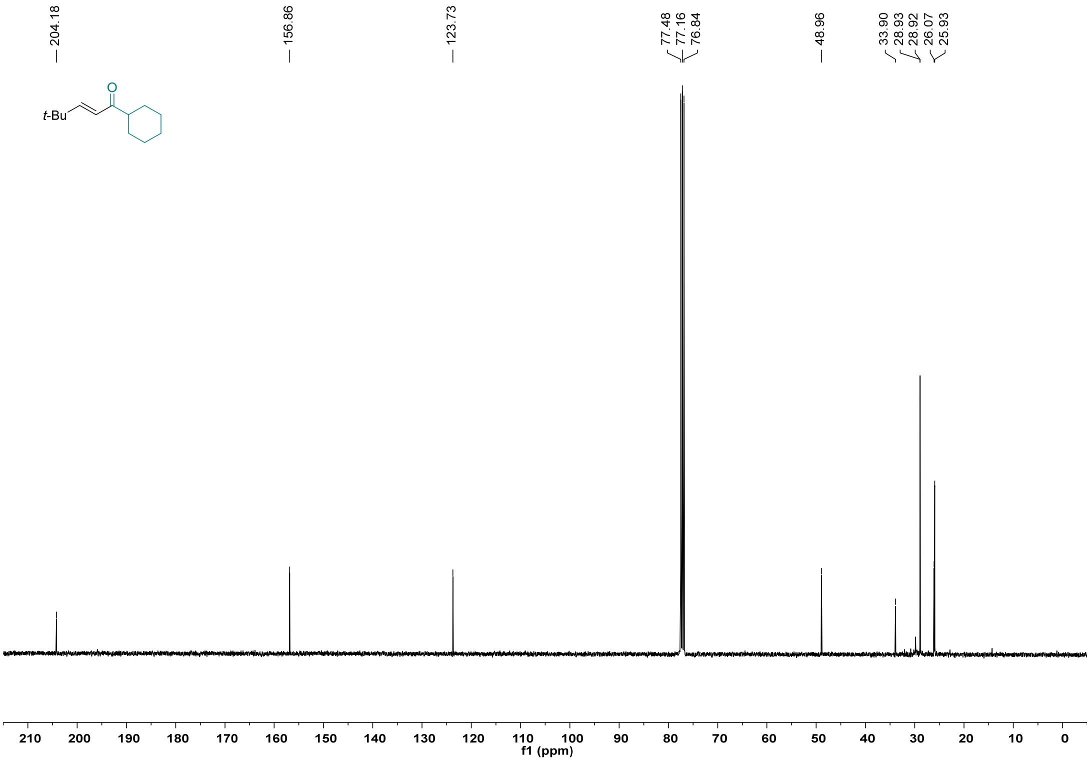

^{13}C NMR (100 MHz, $CDCl_3$) of compound **32**

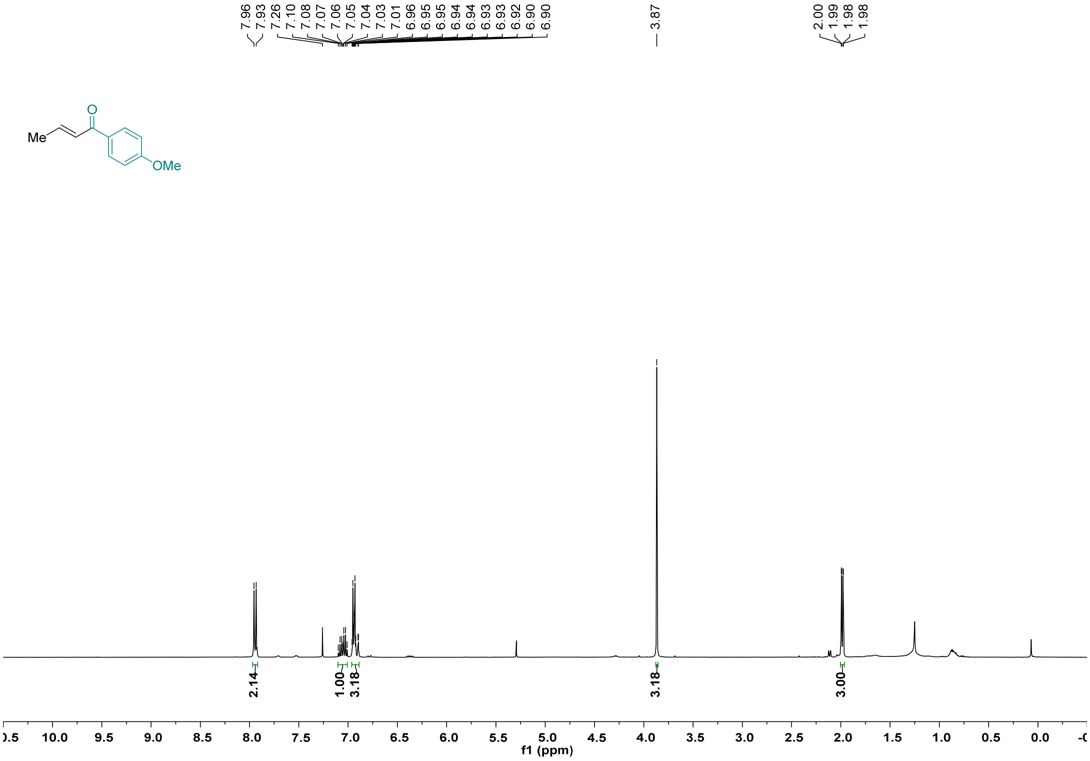

^{1}H NMR (400 MHz, $CDCl_3$) of compound **33**

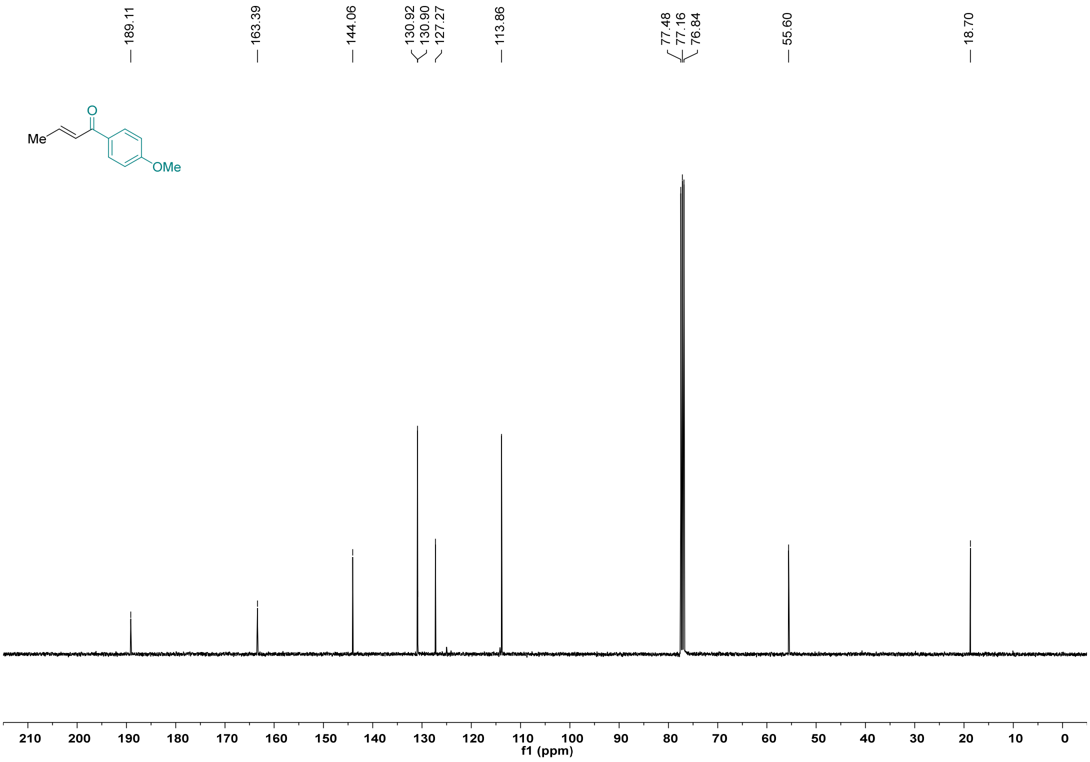

^{13}C NMR (100 MHz, $CDCl_3$) of compound **33**

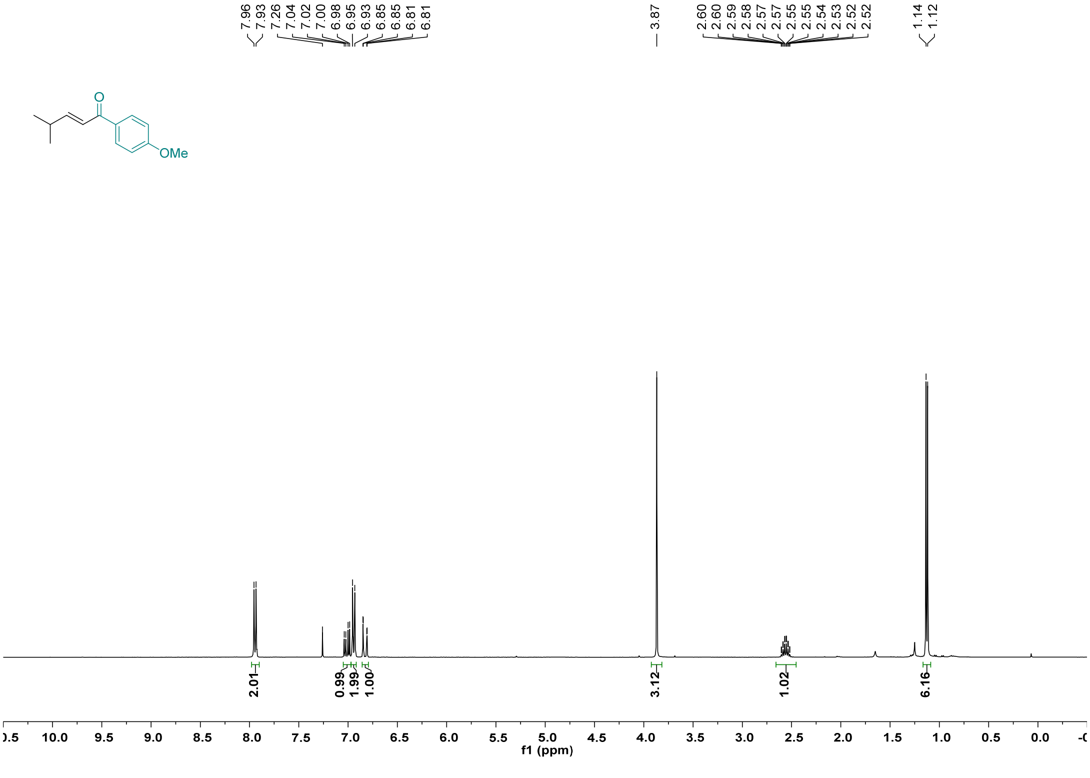

^{1}H NMR (400 MHz, $CDCl_3$) of compound **34**

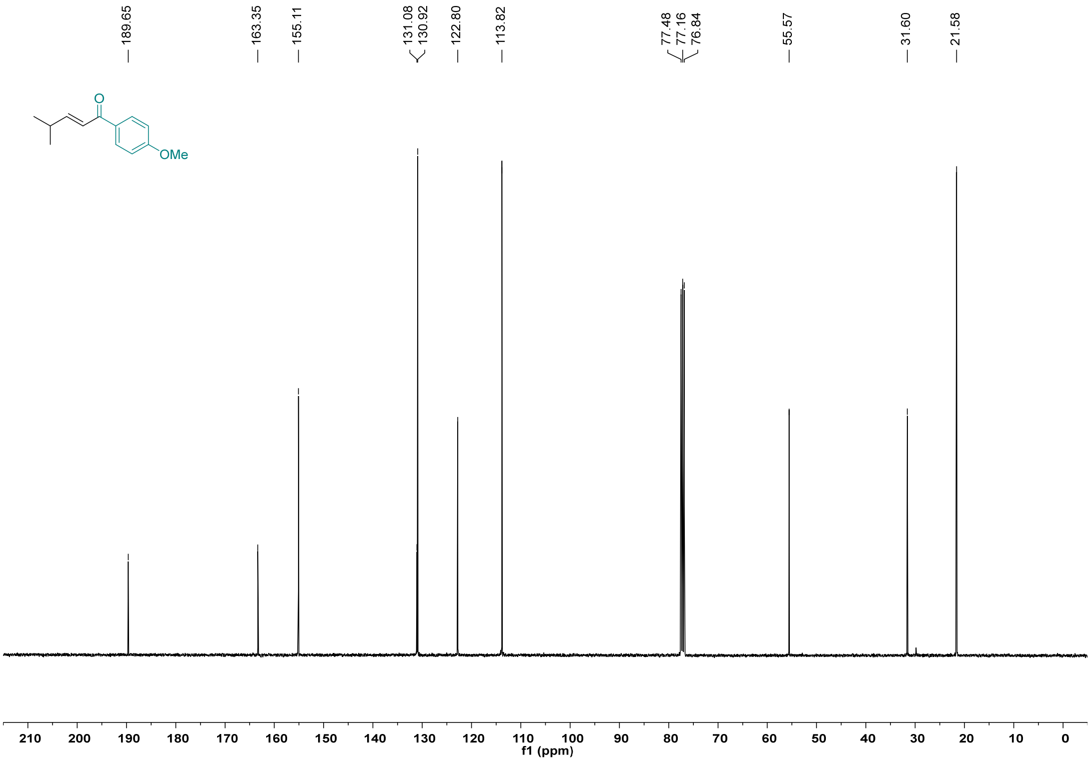

^{13}C NMR (100 MHz, $CDCl_3$) of compound **34**

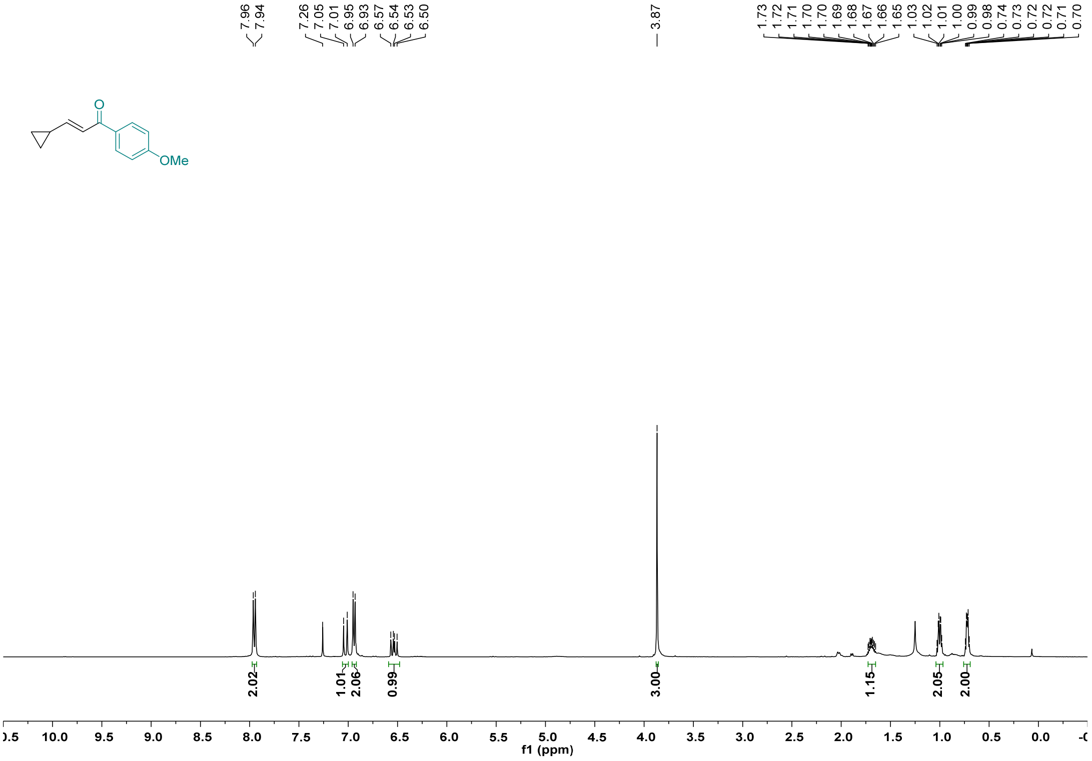

^{1}H NMR (400 MHz, $CDCl_3$) of compound **35**

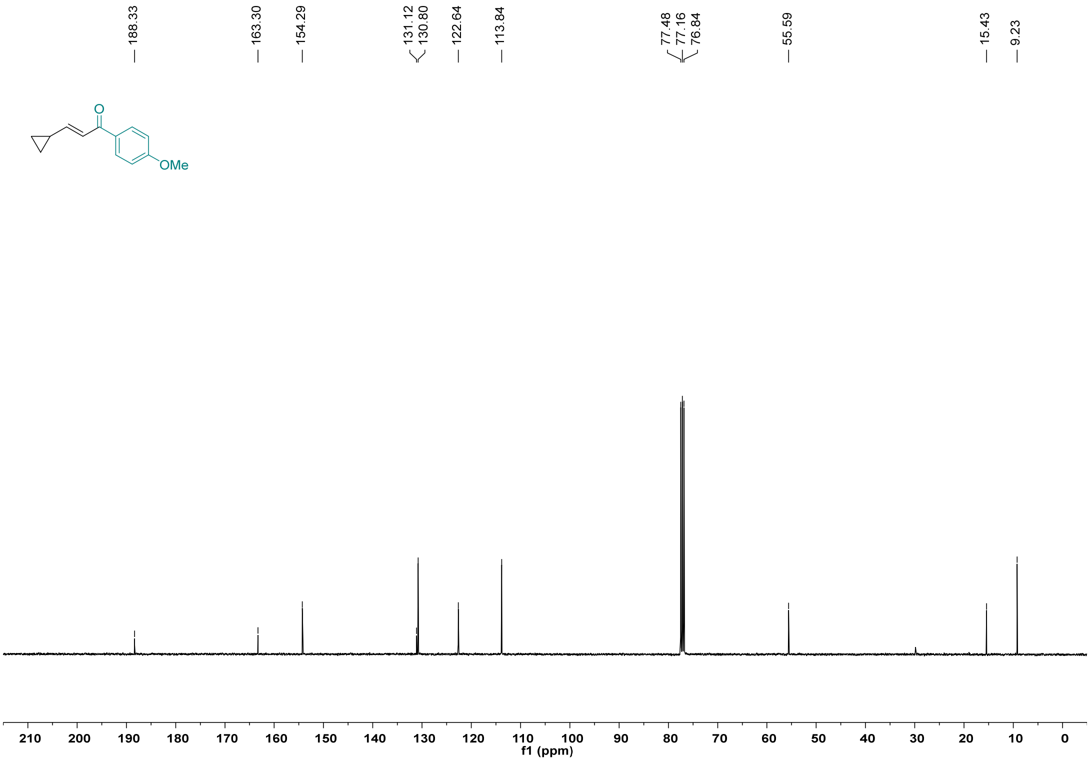

^{13}C NMR (100 MHz, $CDCl_3$) of compound **35**

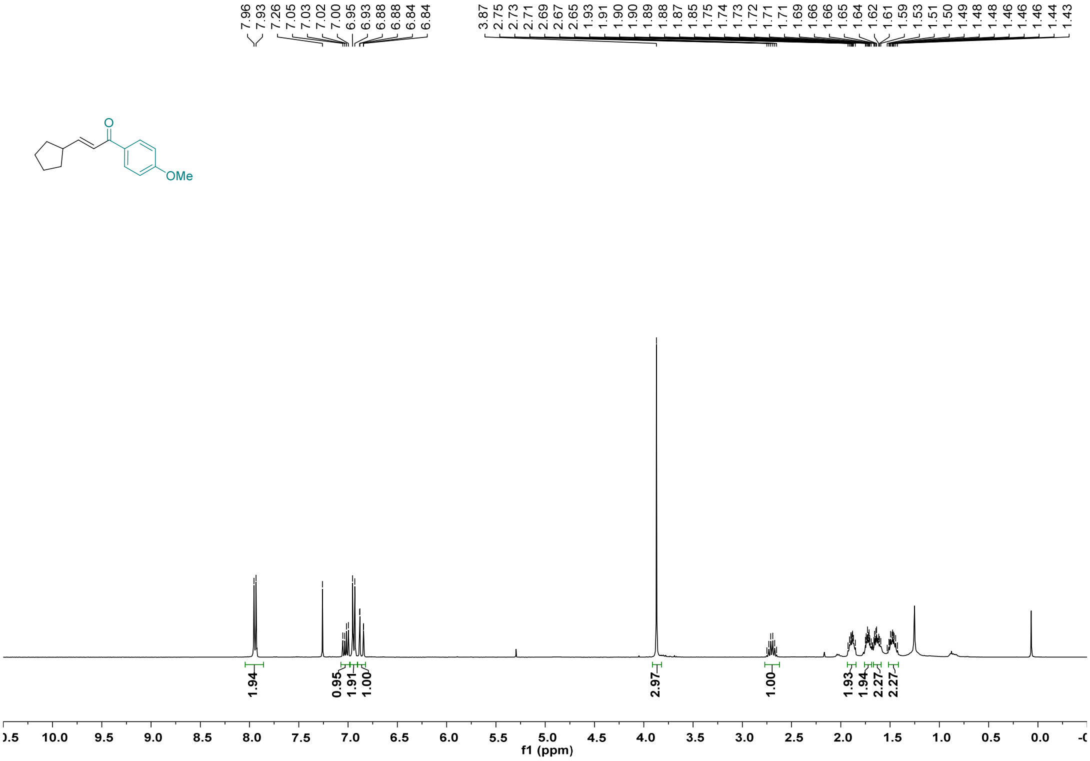

^{1}H NMR (400 MHz, $CDCl_3$) of compound **36**

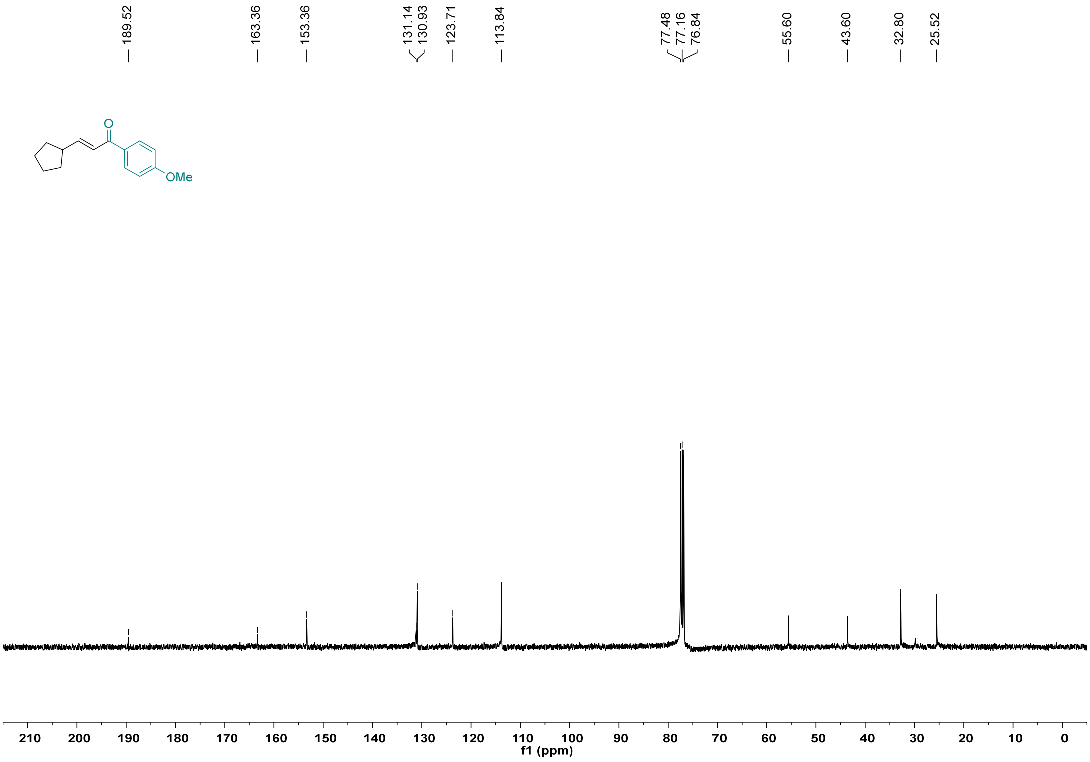

^{13}C NMR (100 MHz, $CDCl_3$) of compound **36**

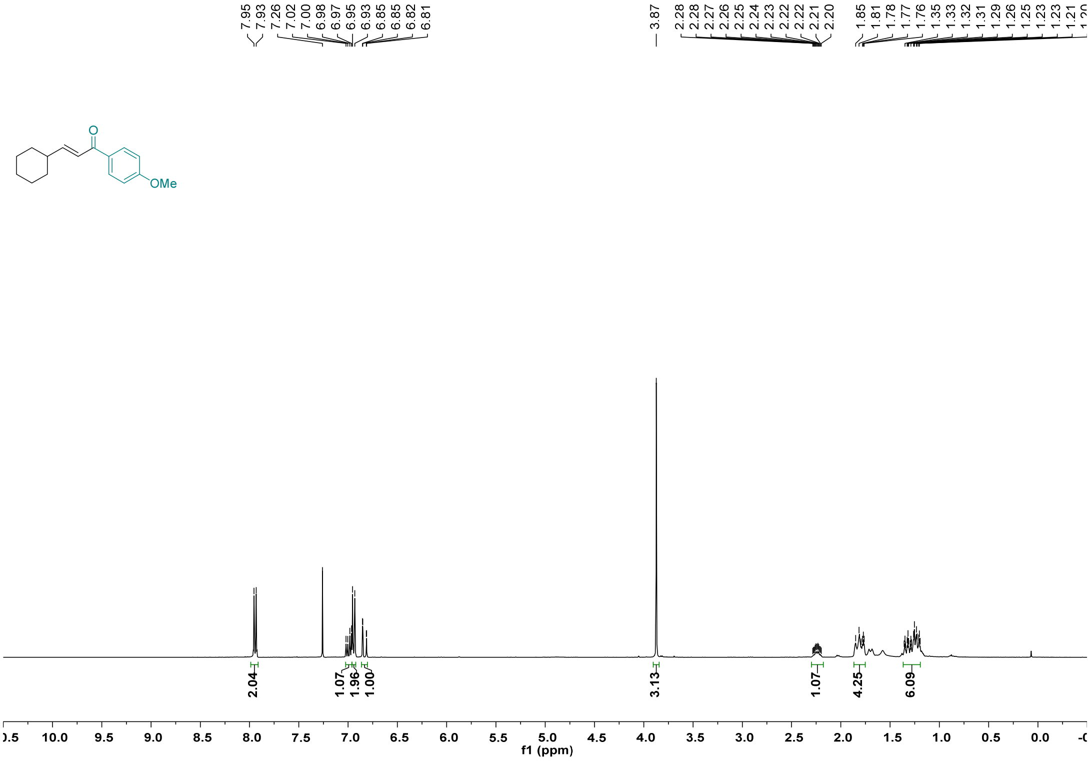

^{1}H NMR (400 MHz, $CDCl_3$) of compound **37**

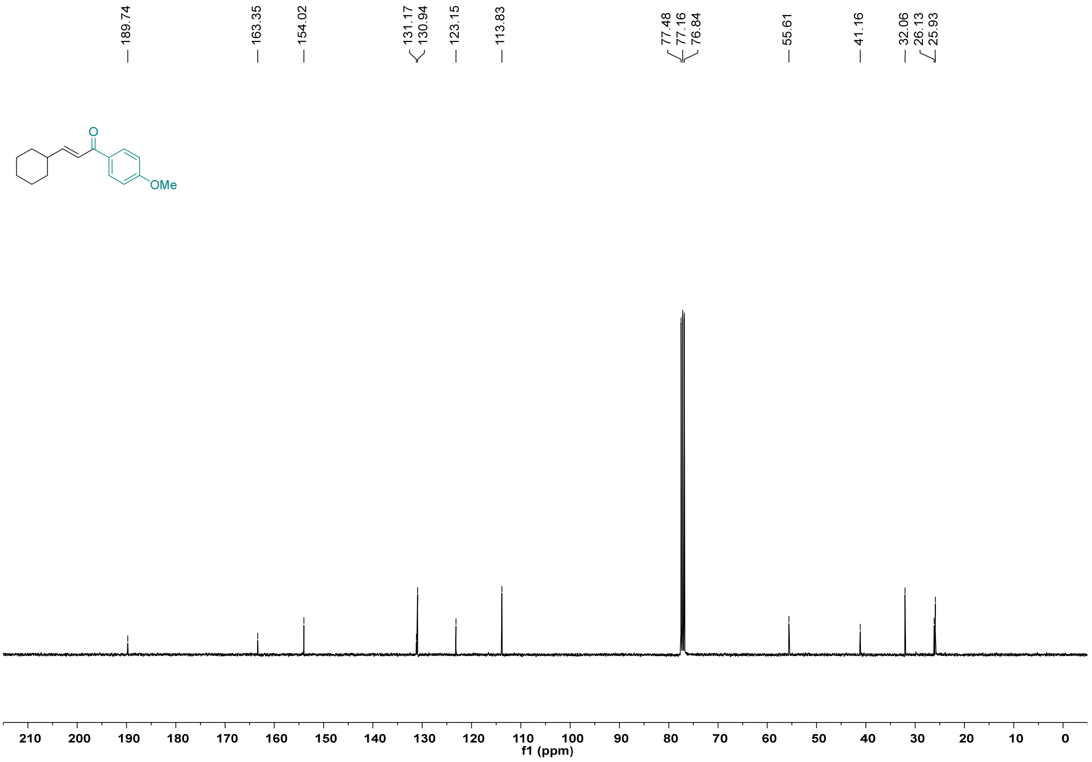

^{13}C NMR (100 MHz, $CDCl_3$) of compound **37**

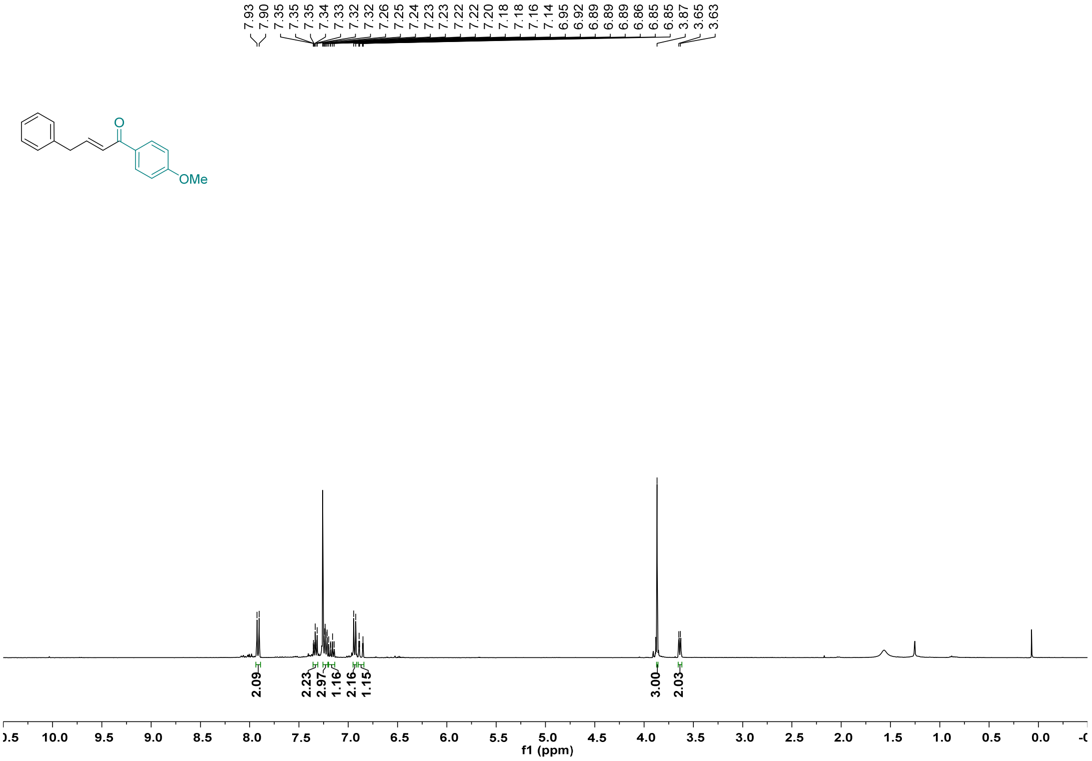

^{1}H NMR (400 MHz, $CDCl_3$) of compound **38**

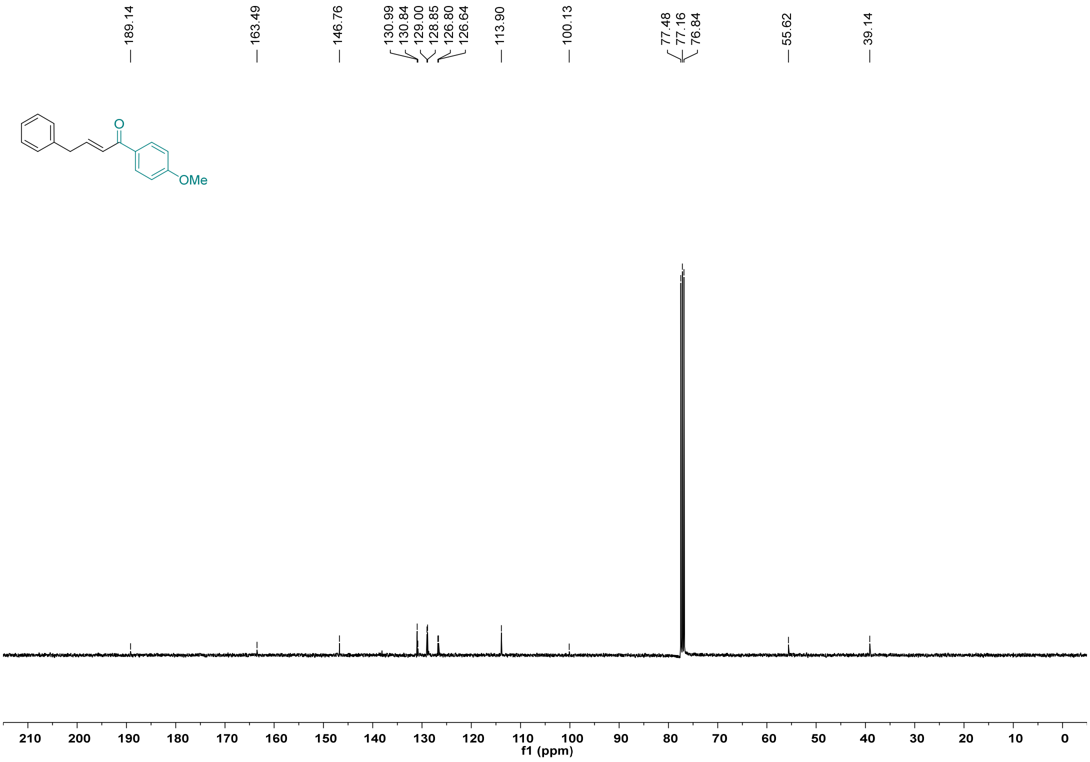

^{13}C NMR (100 MHz, $CDCl_3$) of compound **38**

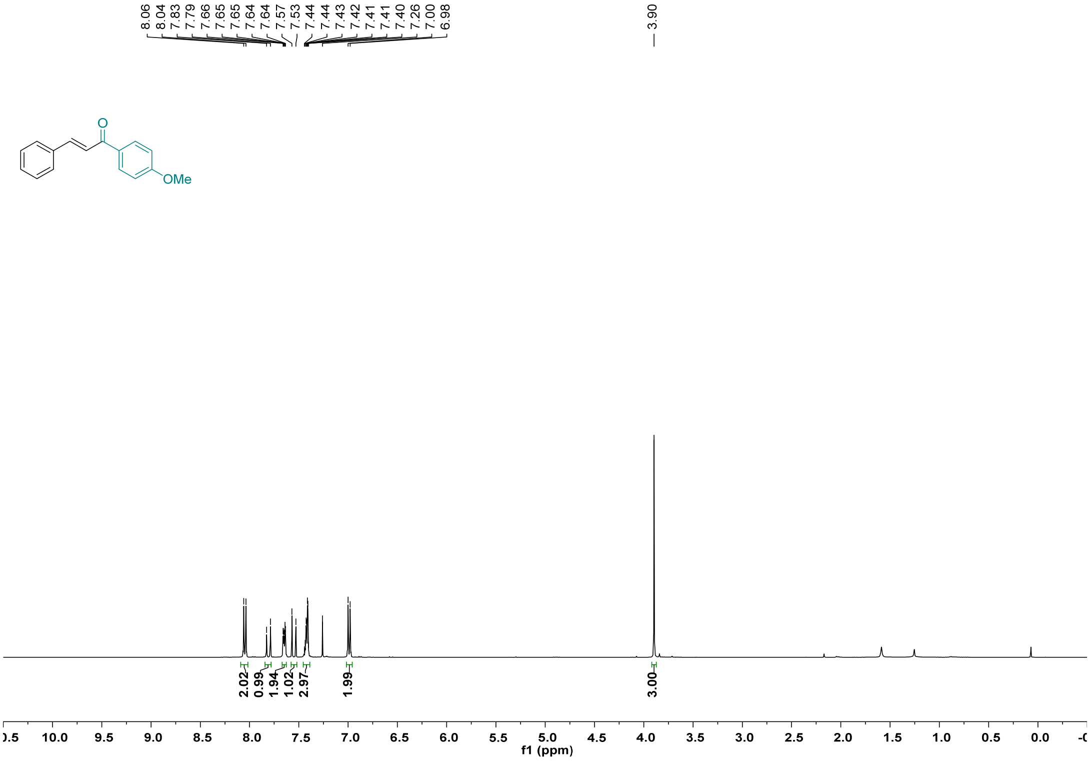

^{1}H NMR (400 MHz, $CDCl_3$) of compound **39**

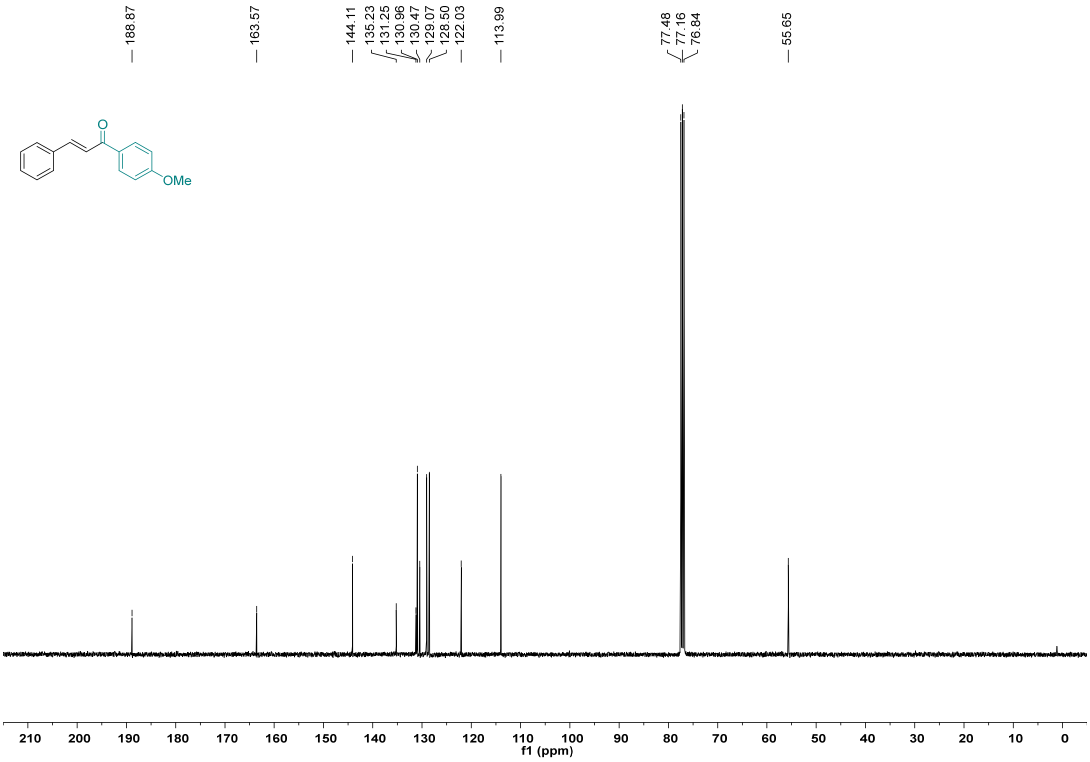

^{13}C NMR (100 MHz, CDCl$_3$) of compound **39**

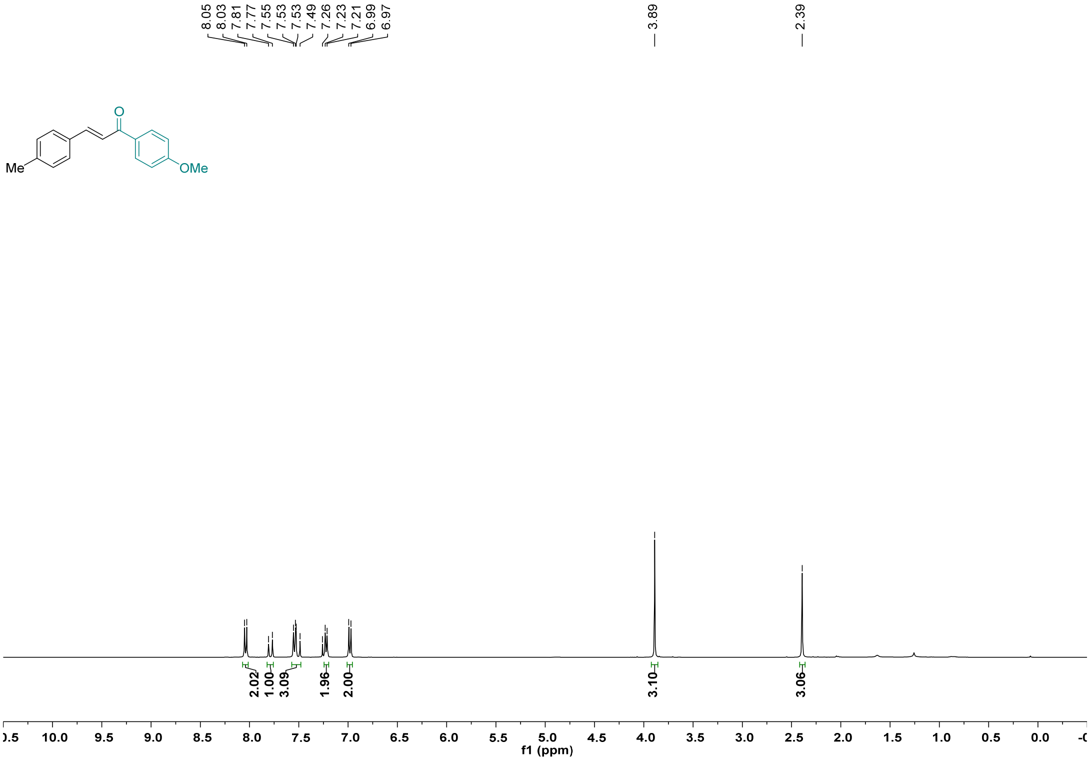

^{1}H NMR (400 MHz, CDCl$_3$) of compound **40**

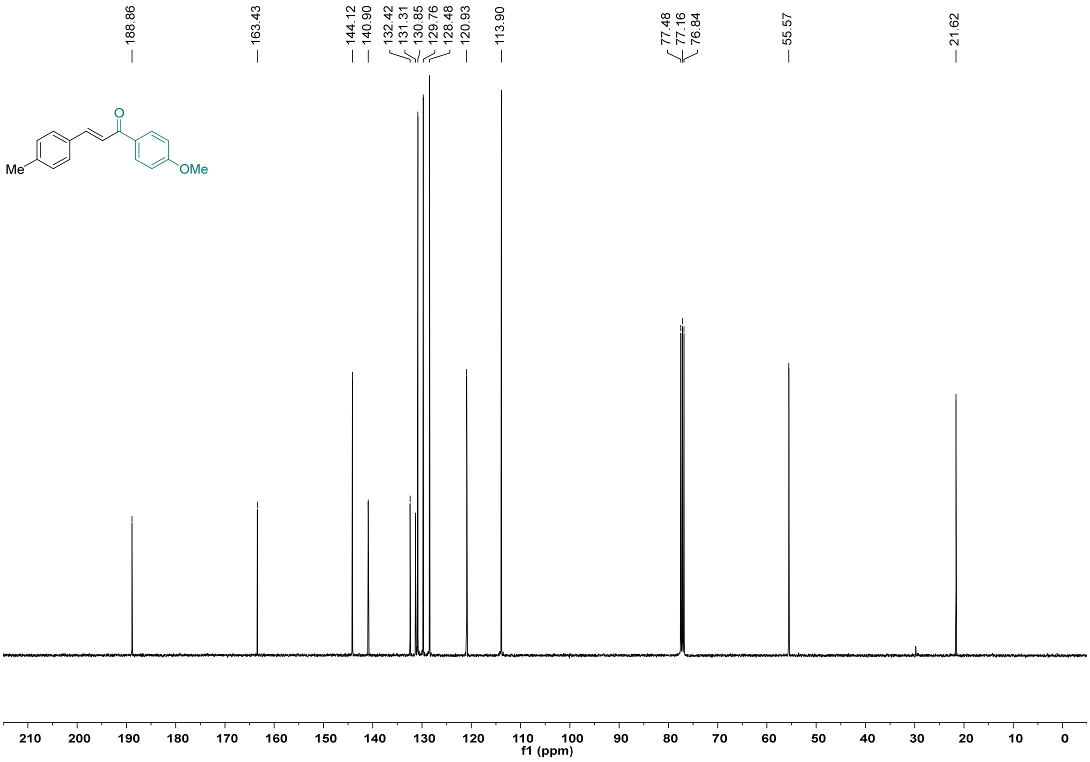

^{13}C NMR (100 MHz, $CDCl_3$) of compound **40**

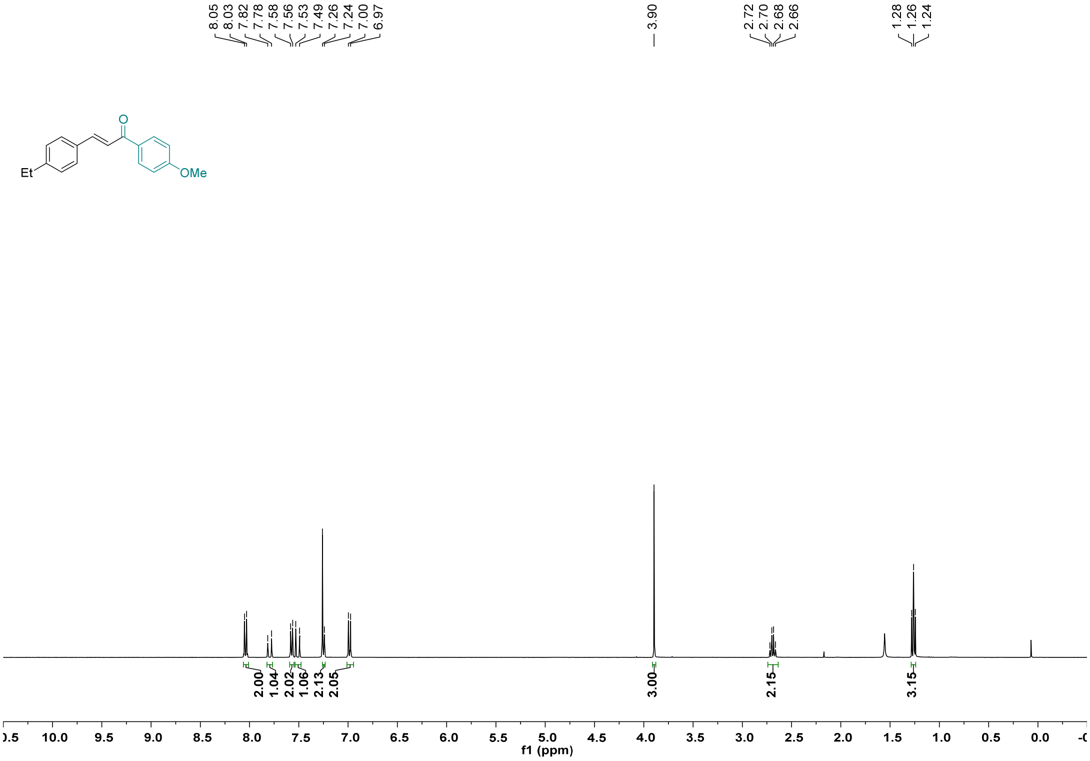

^{1}H NMR (400 MHz, $CDCl_3$) of compound **41**

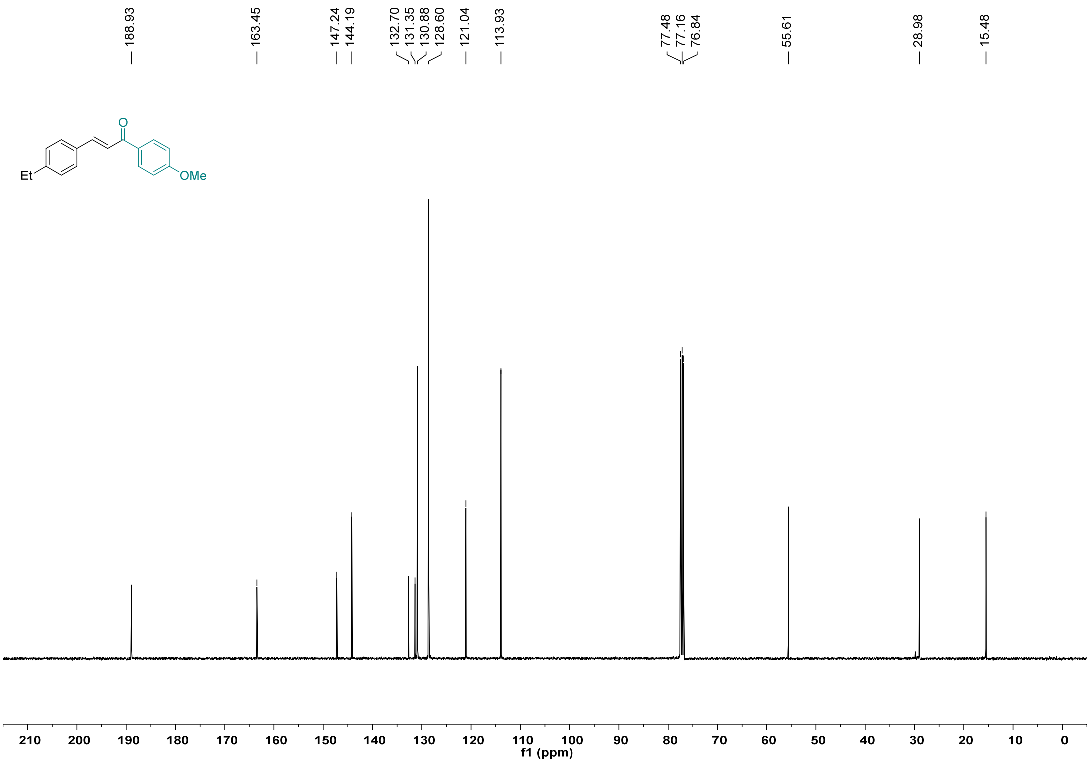

^{13}C NMR (100 MHz, $CDCl_3$) of compound **41**

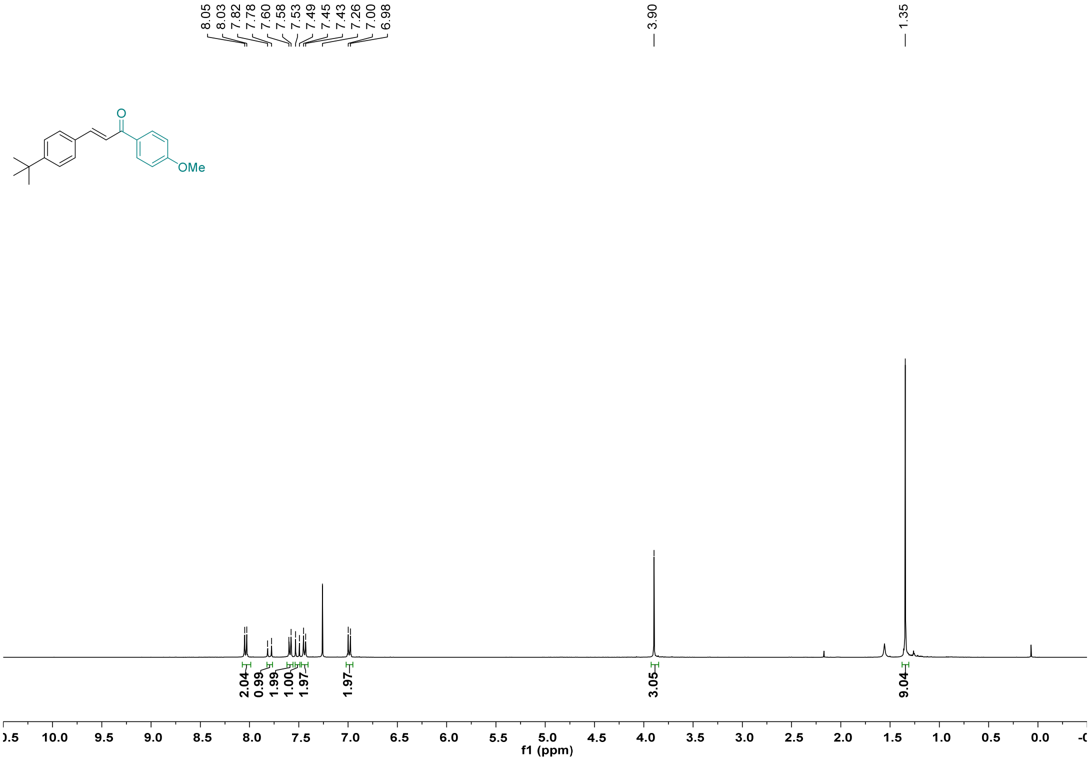

^{1}H NMR (400 MHz, $CDCl_3$) of compound **42**

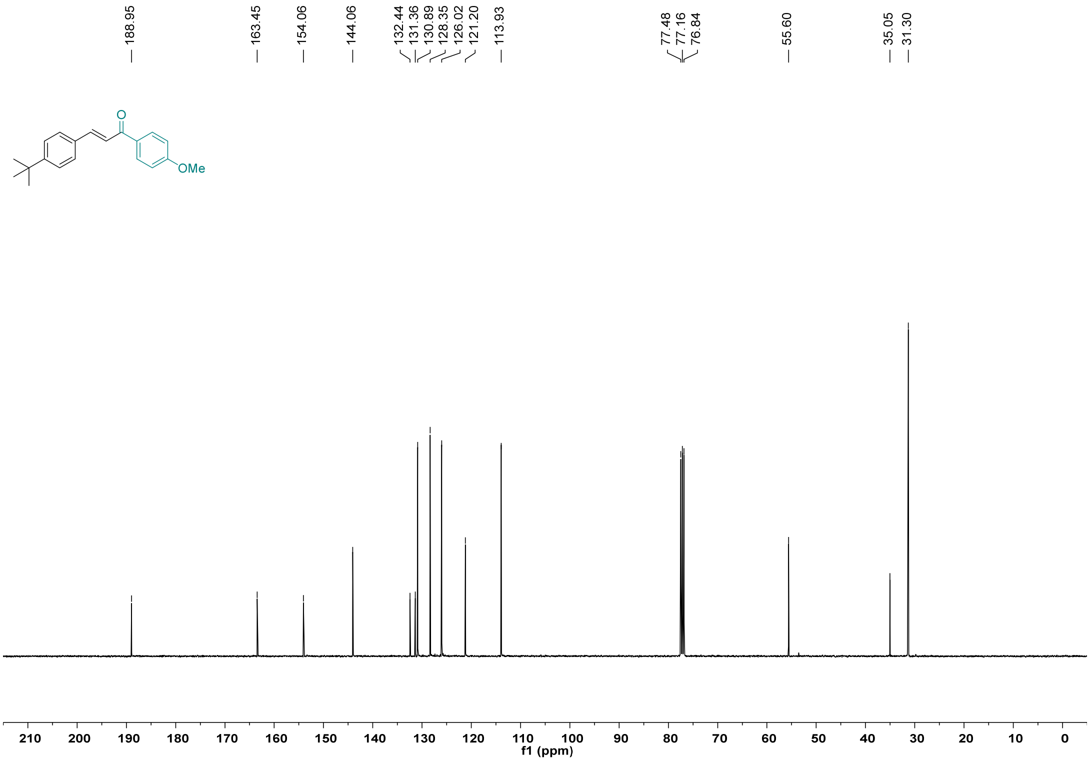

^{13}C NMR (100 MHz, CDCl$_3$) of compound **42**

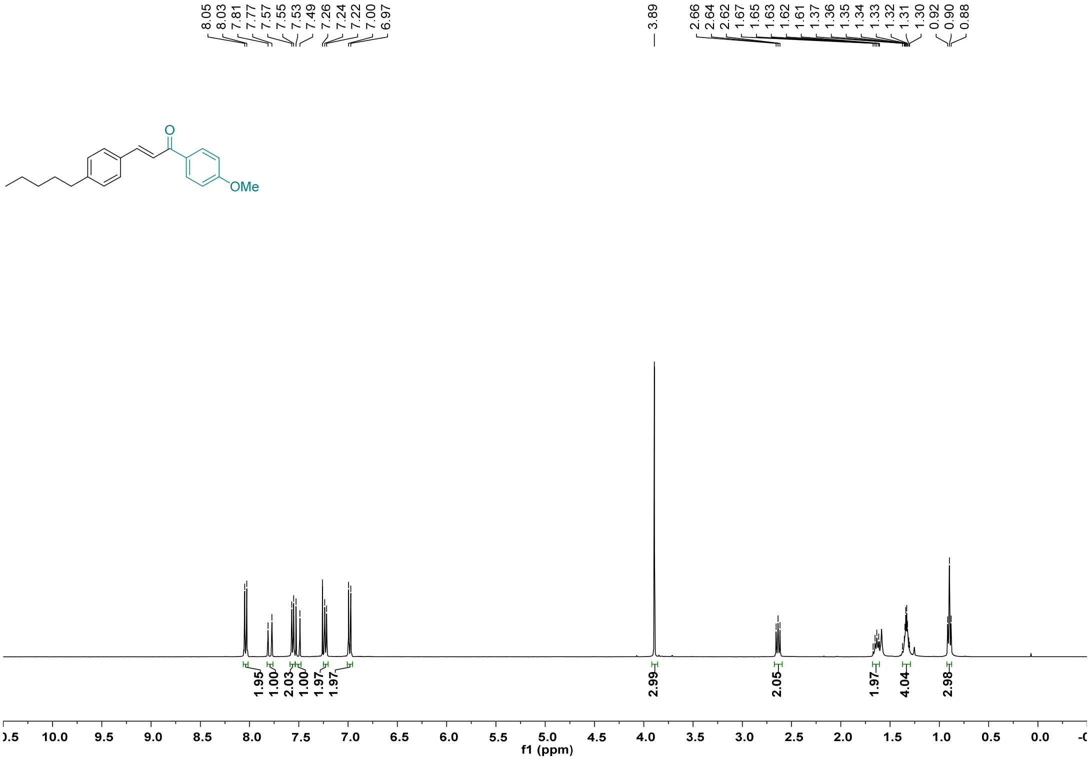

^{1}H NMR (400 MHz, CDCl$_3$) of compound **43**

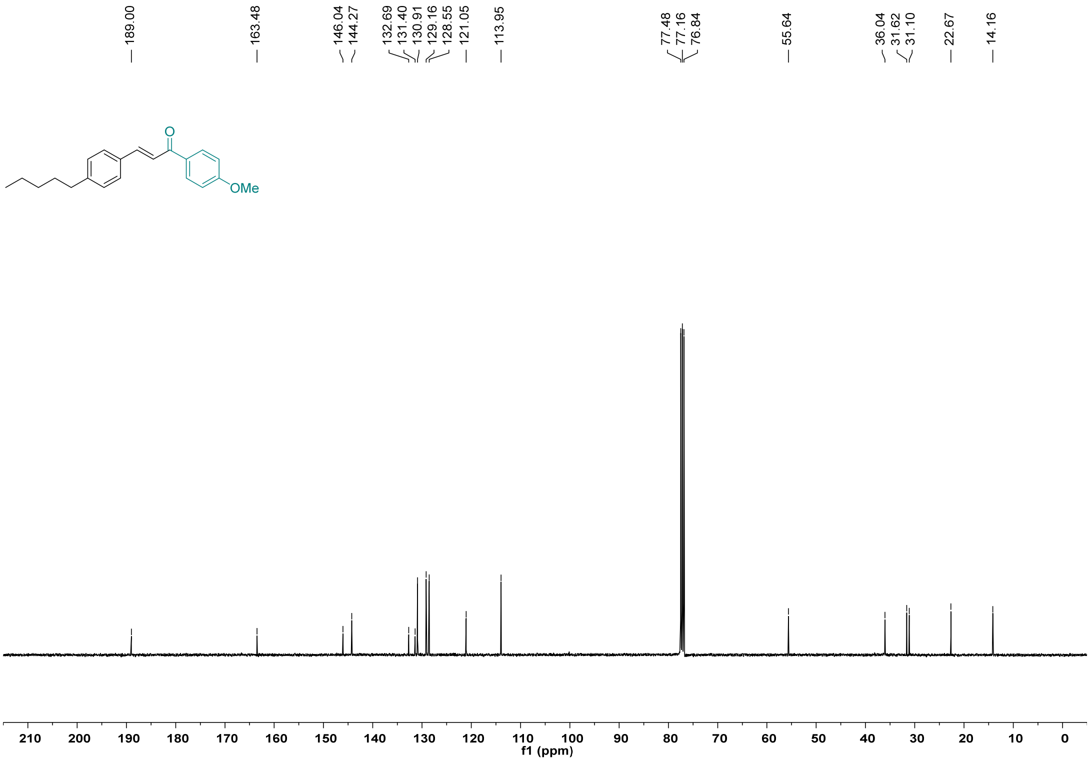

^{13}C NMR (100 MHz, $CDCl_3$) of compound **43**

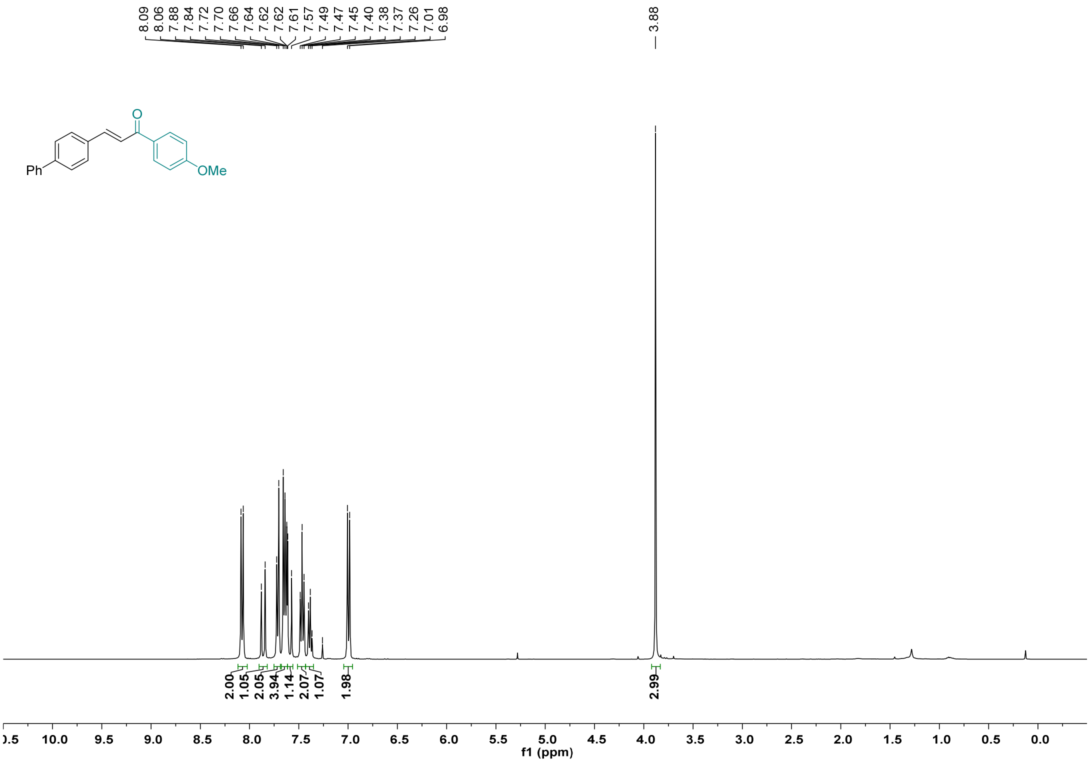

^{1}H NMR (400 MHz, $CDCl_3$) of compound **44**

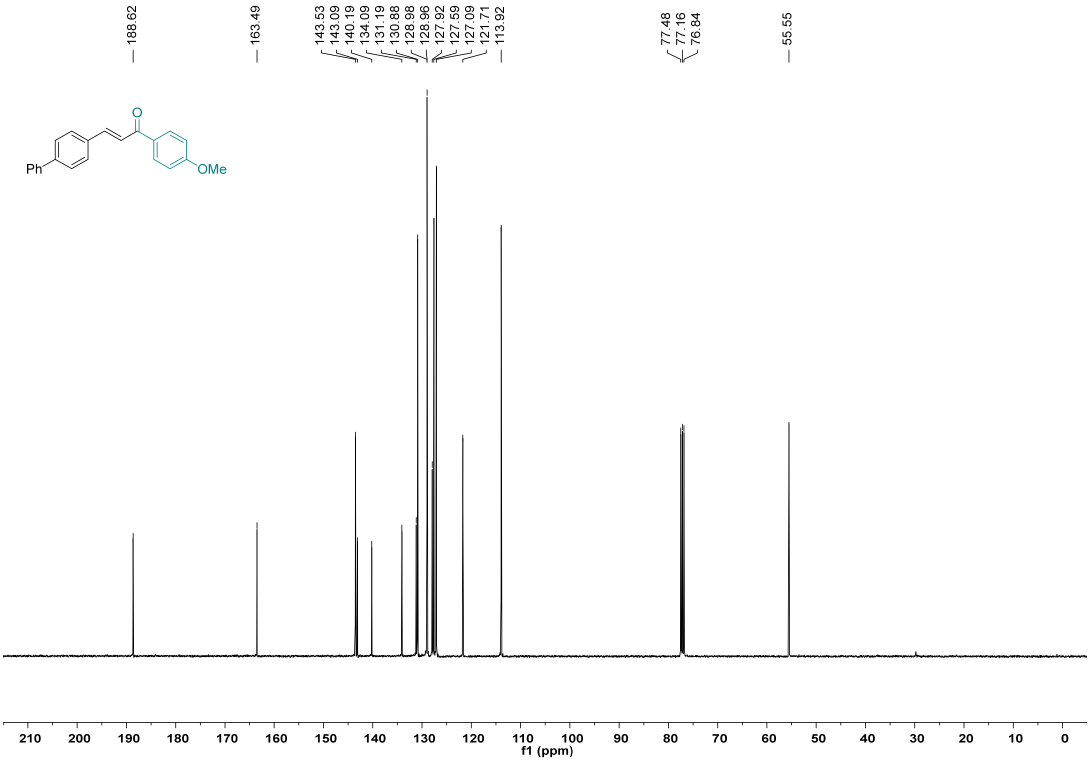

^{13}C NMR (100 MHz, CDCl$_3$) of compound **44**

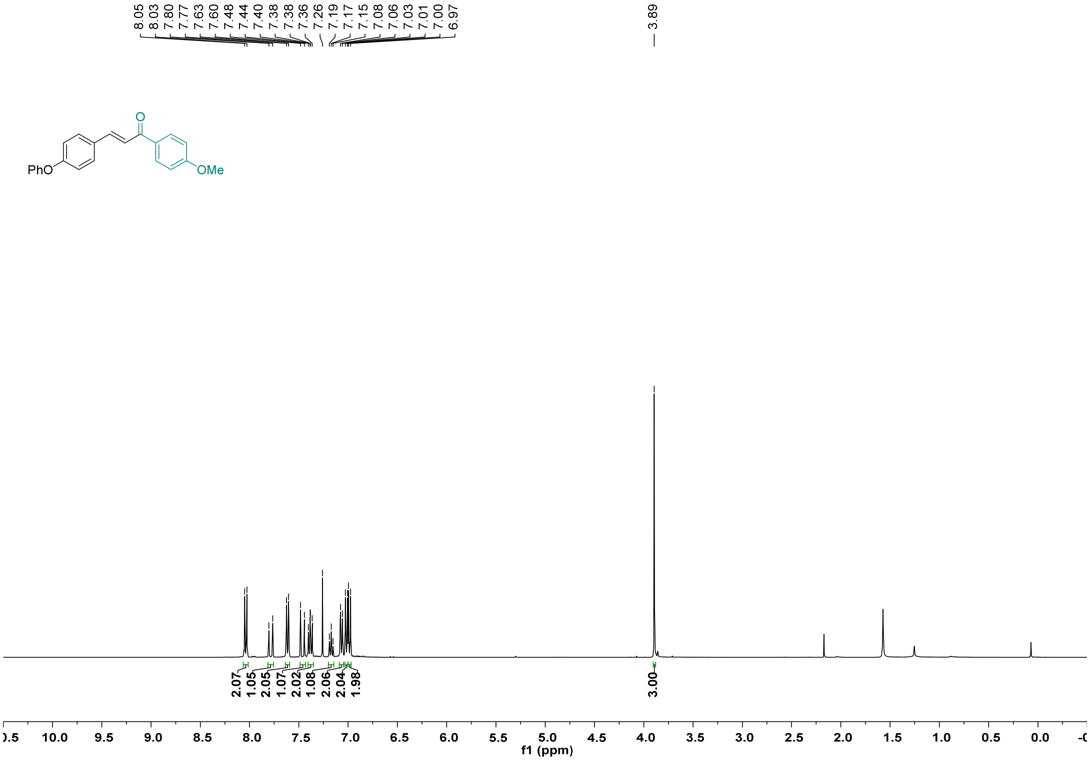

^{1}H NMR (400 MHz, CDCl$_3$) of compound **45**

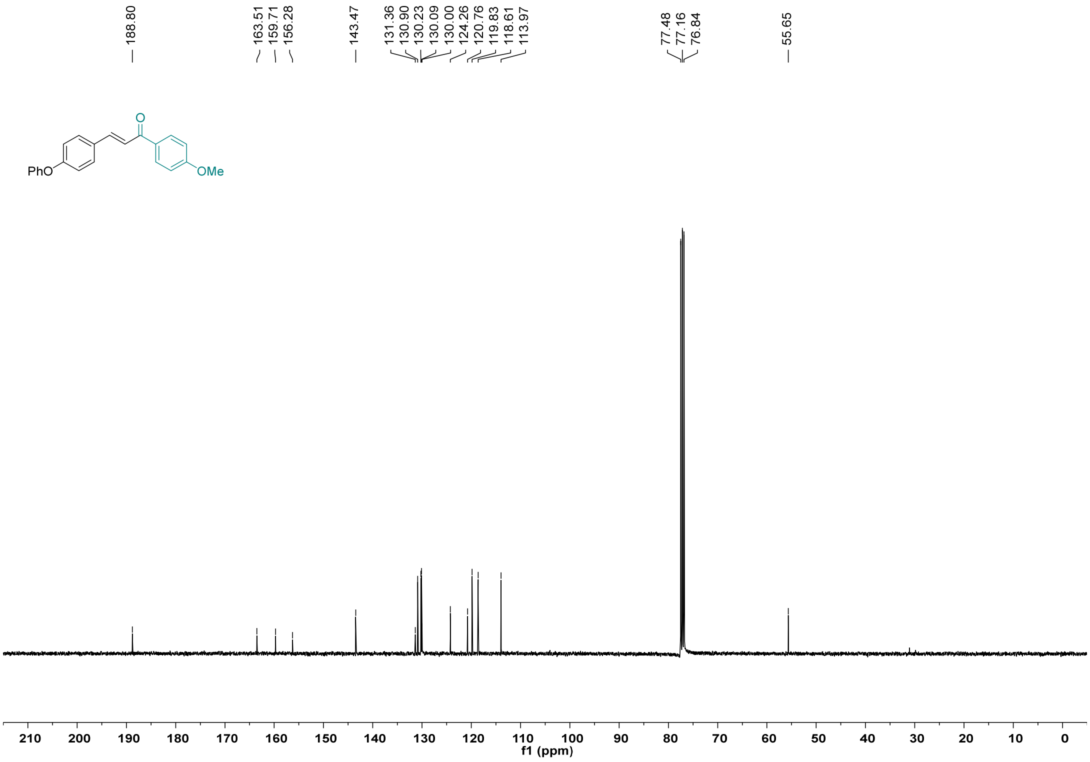

^{13}C NMR (100 MHz, $CDCl_3$) of compound **45**

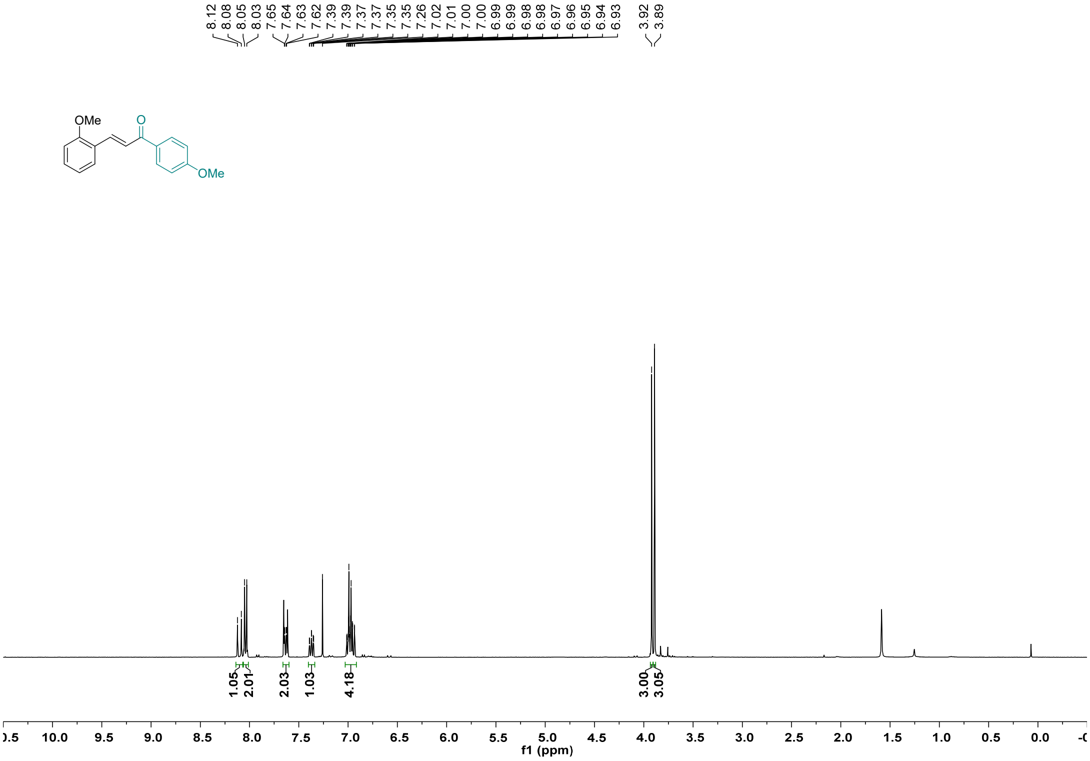

^{1}H NMR (400 MHz, $CDCl_3$) of compound **46**

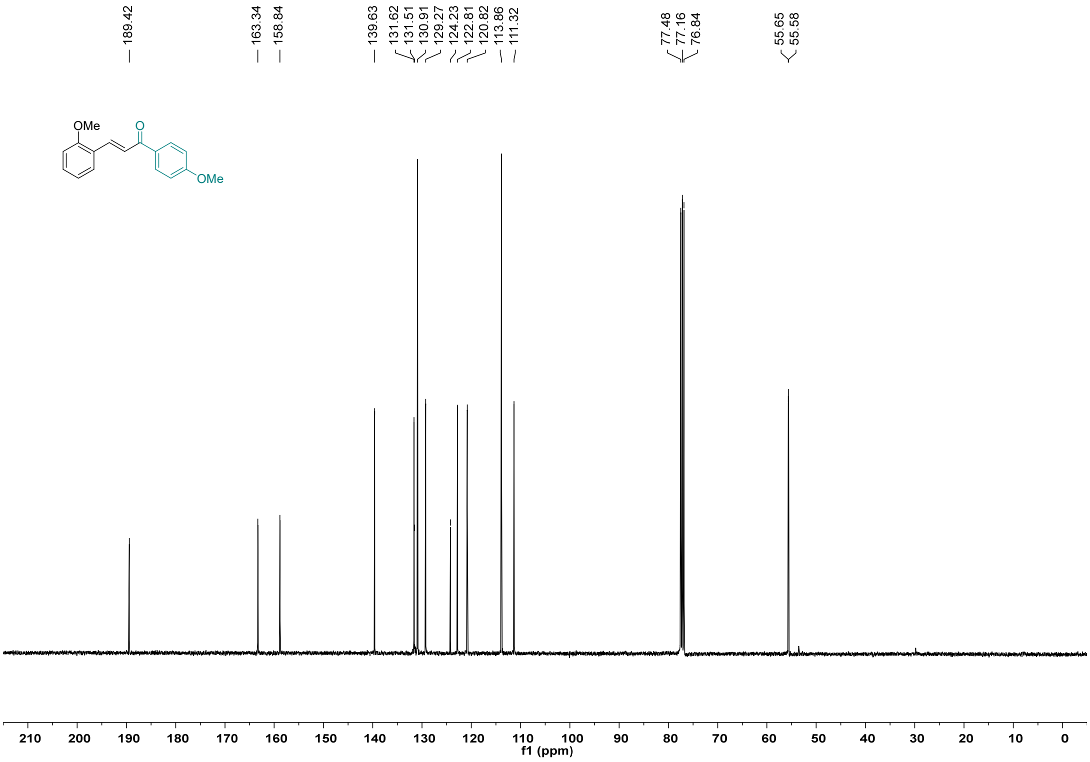

^{13}C NMR (100 MHz, $CDCl_3$) of compound **46**

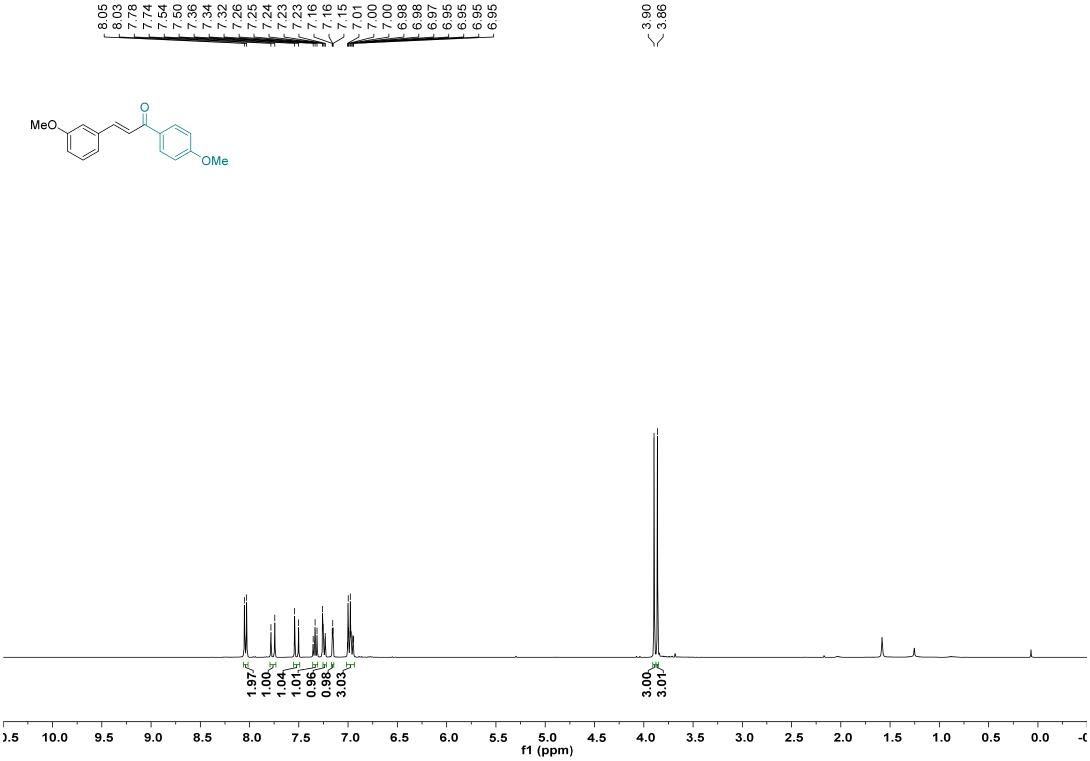

^{1}H NMR (400 MHz, $CDCl_3$) of compound **47**

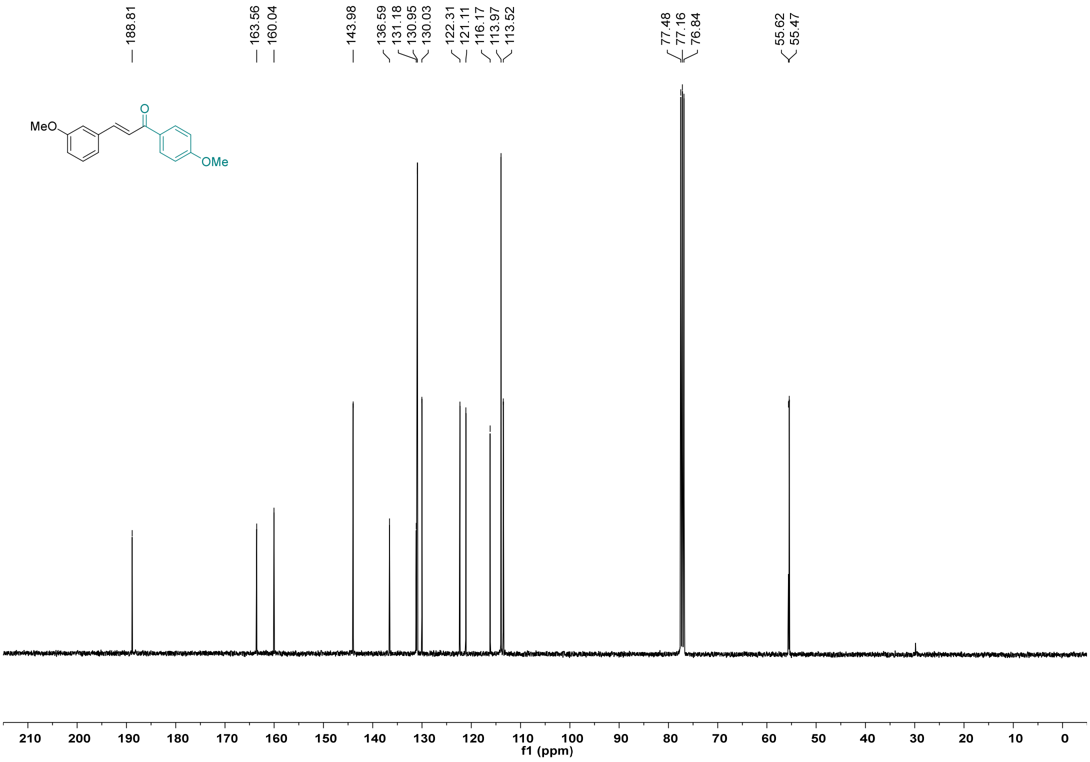

^{13}C NMR (100 MHz, $CDCl_3$) of compound **47**

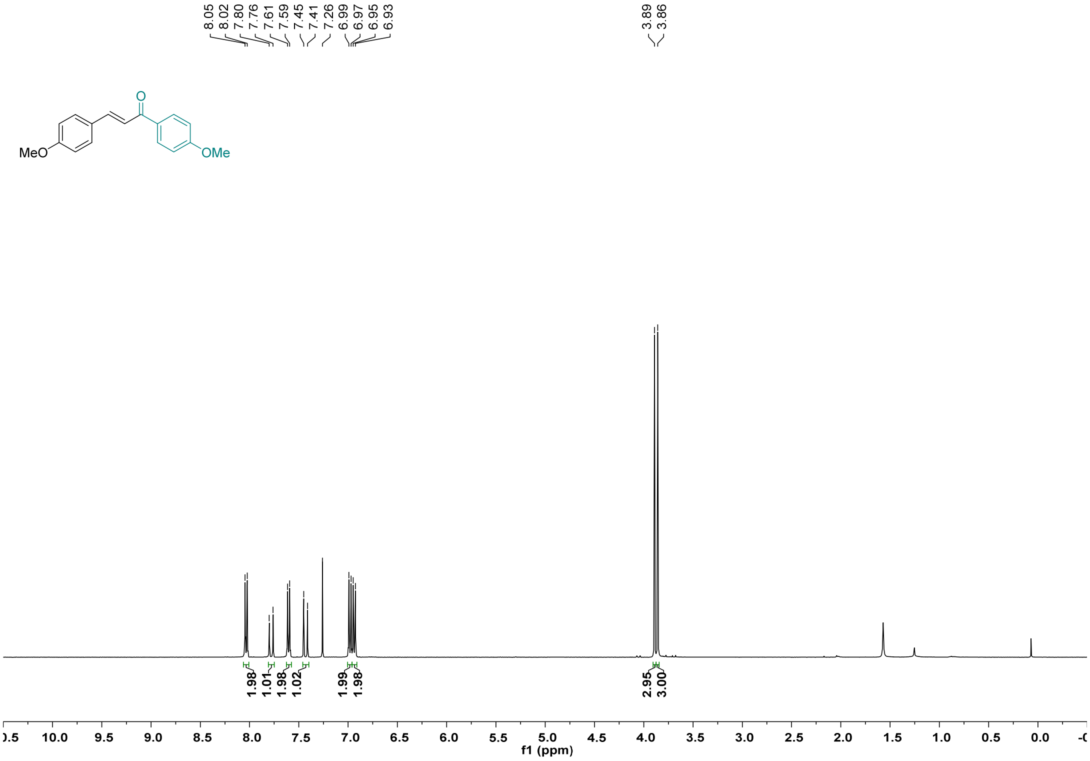

^{1}H NMR (400 MHz, $CDCl_3$) of compound **48**

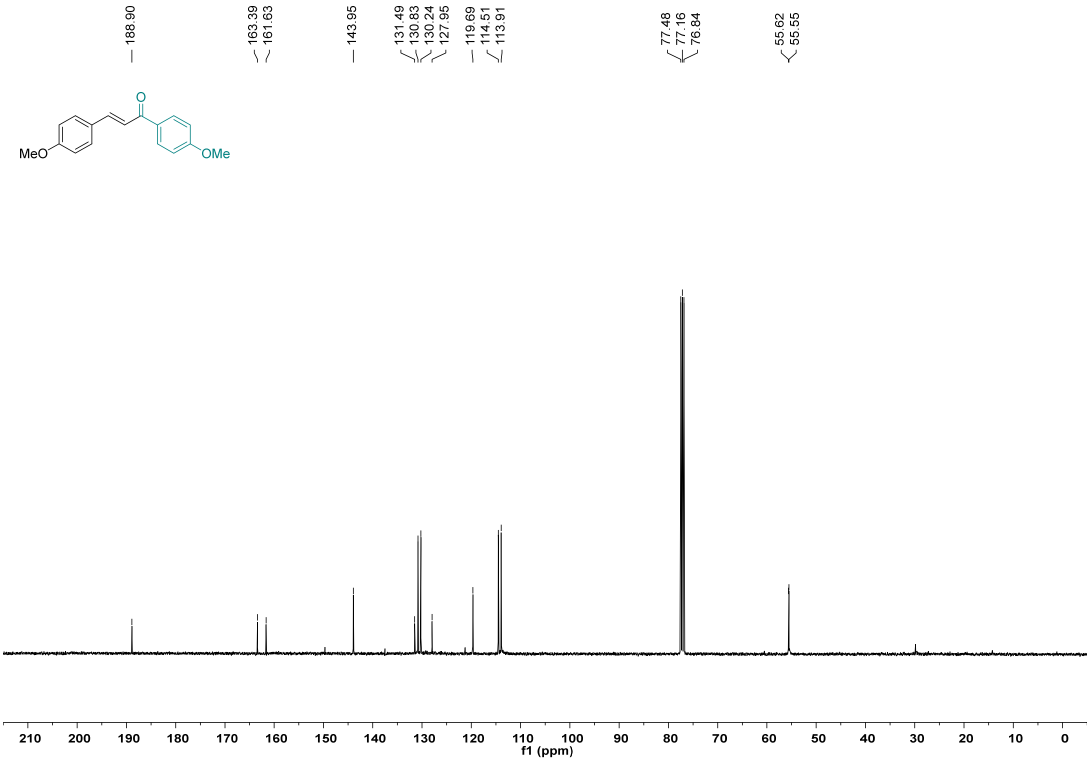

^{13}C NMR (100 MHz, $CDCl_3$) of compound **48**

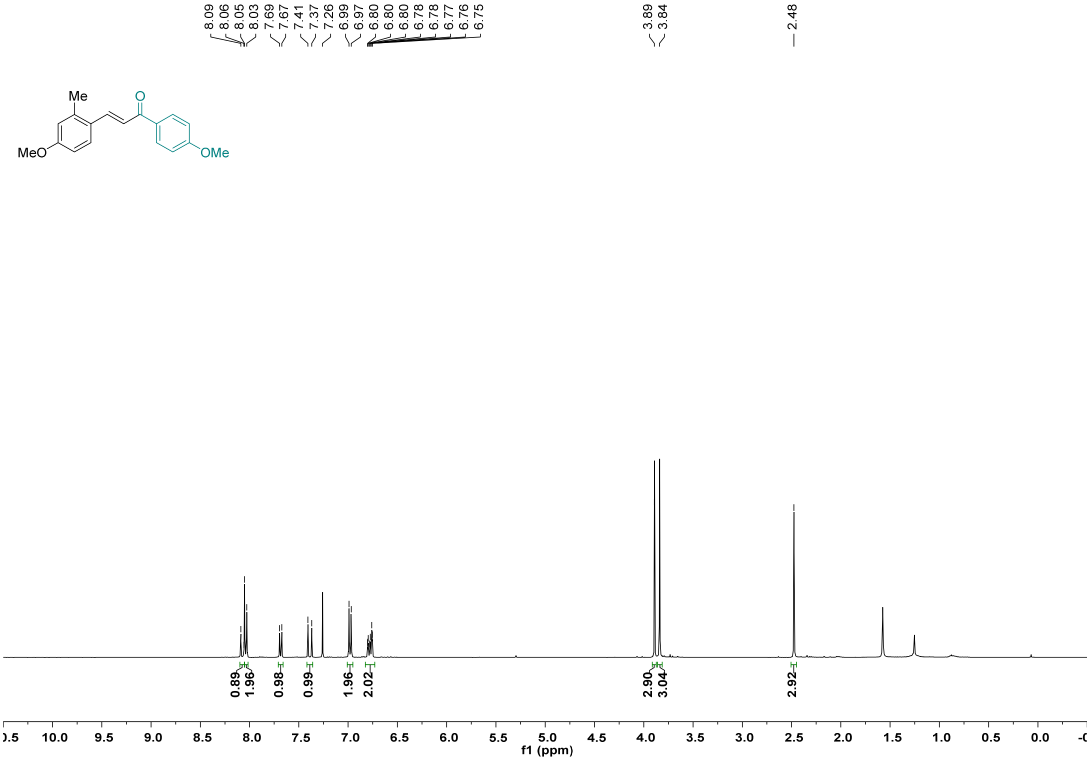

^{1}H NMR (400 MHz, $CDCl_3$) of compound **49**

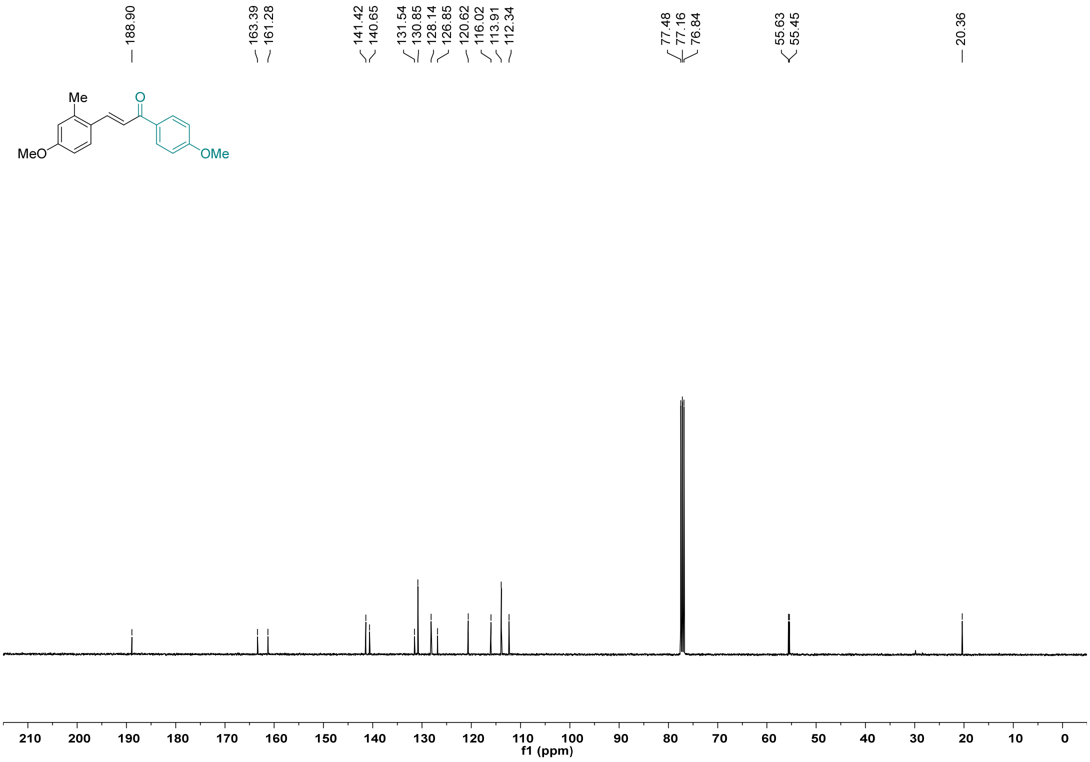

^{13}C NMR (100 MHz, CDCl$_3$) of compound **49**

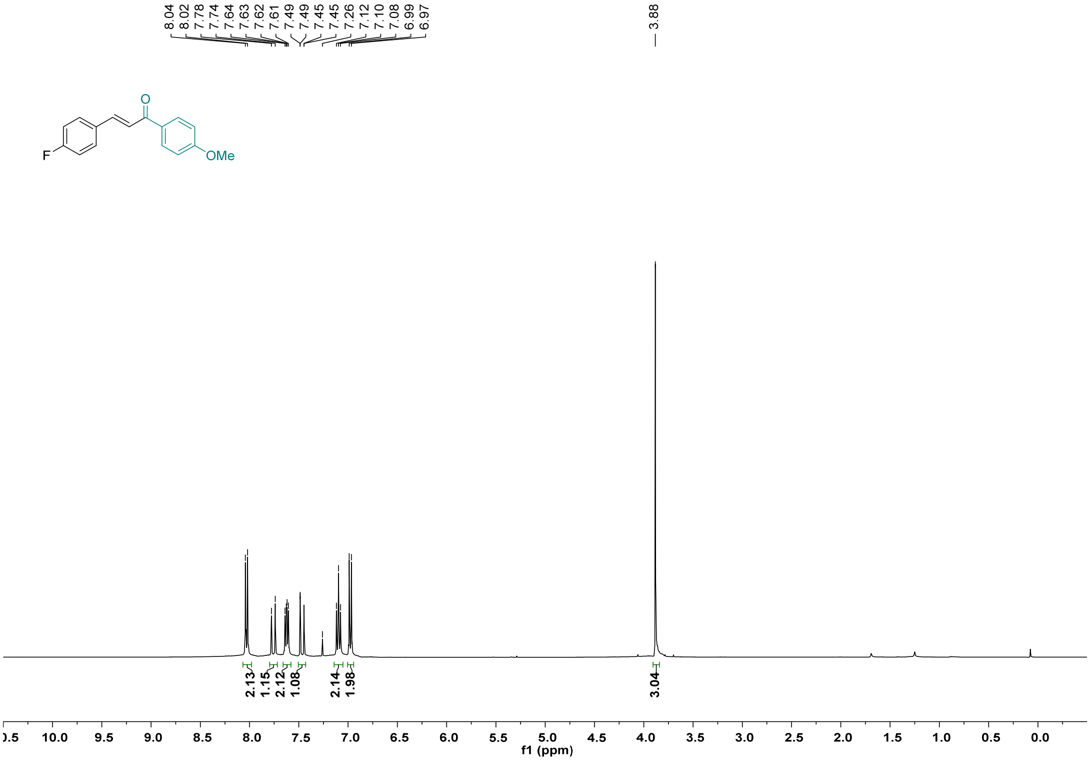

^{1}H NMR (400 MHz, CDCl$_3$) of compound **50**

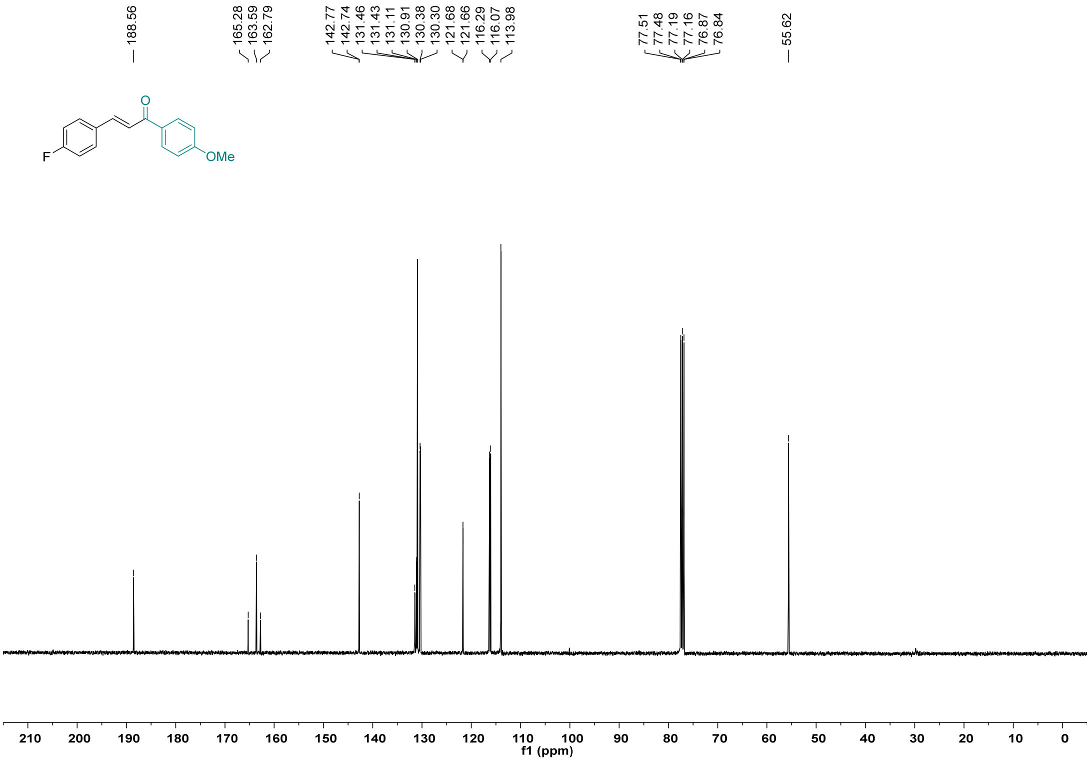

^{13}C NMR (100 MHz, $CDCl_3$) of compound **50**

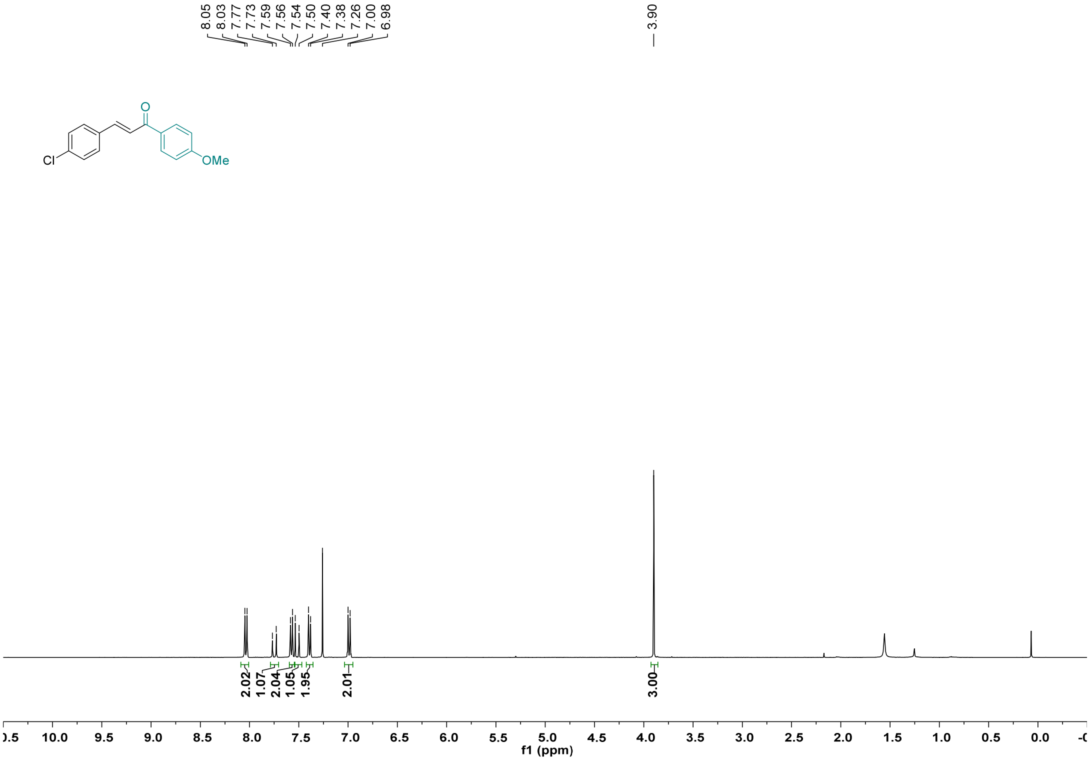

^{1}H NMR (400 MHz, $CDCl_3$) of compound **51**

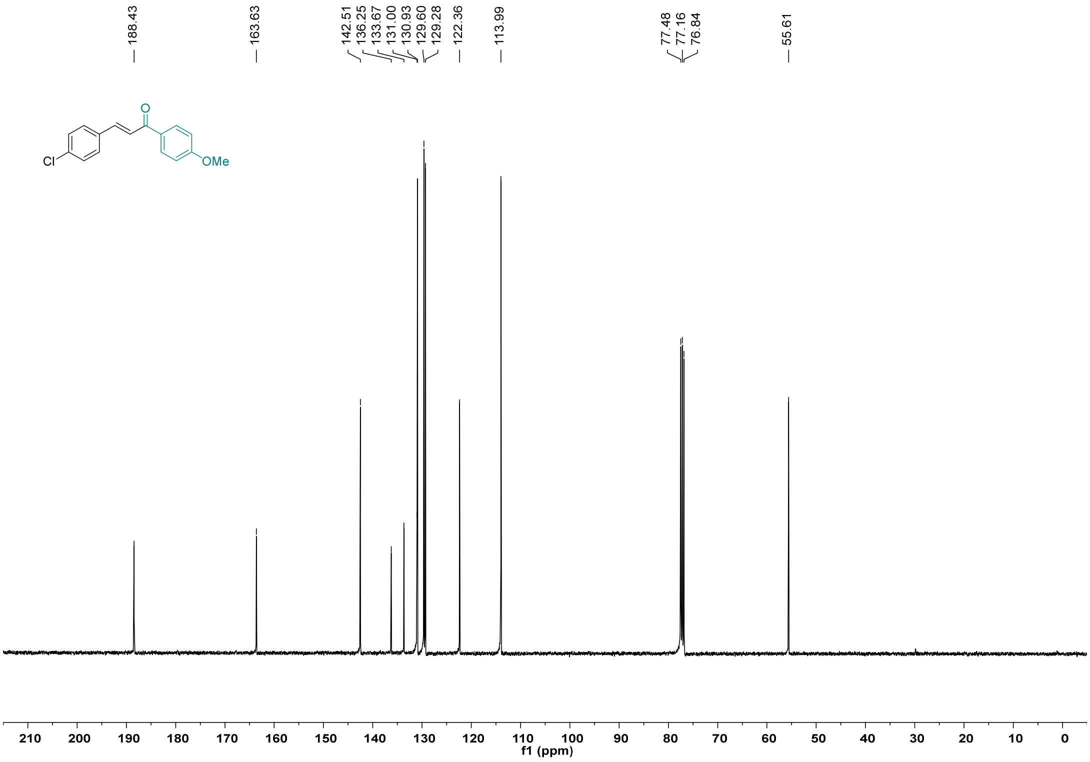

^{13}C NMR (100 MHz, $CDCl_3$) of compound **51**

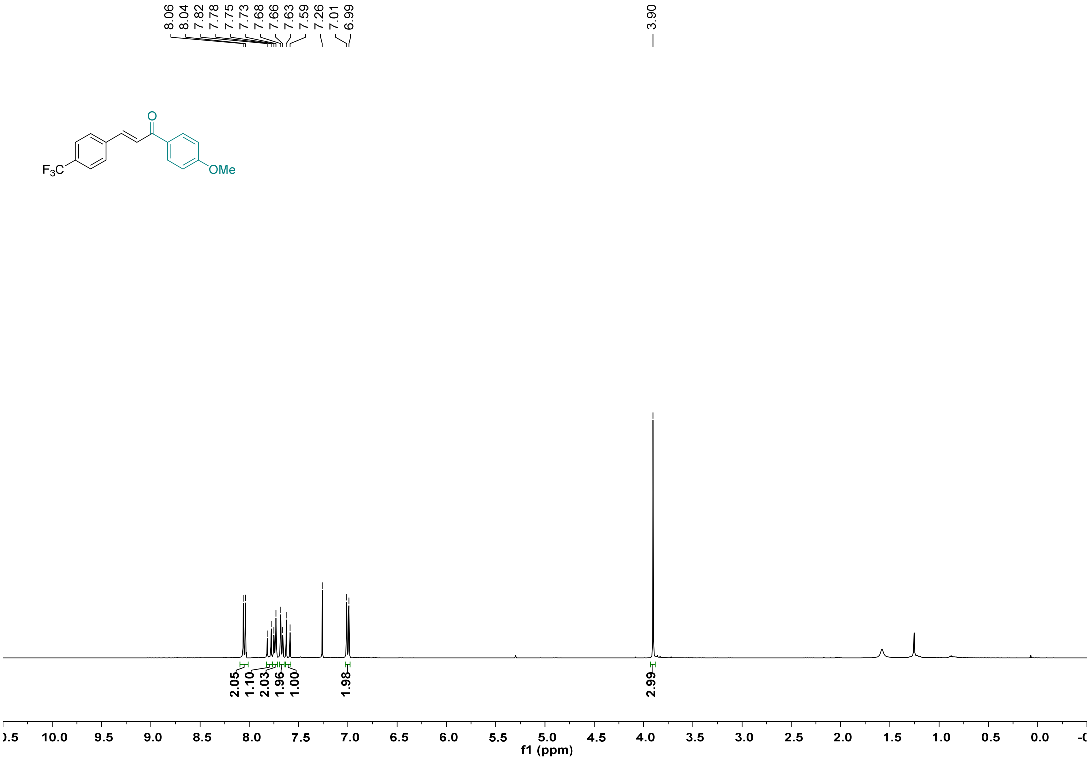

^{1}H NMR (400 MHz, $CDCl_3$) of compound **52**

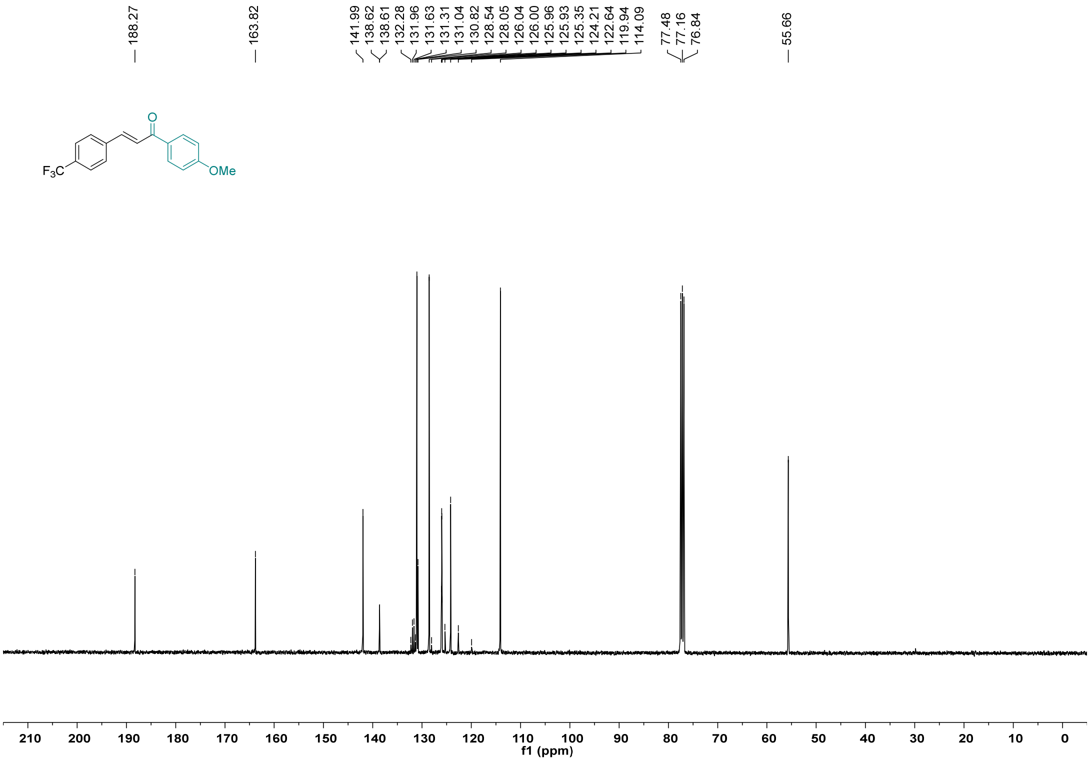

^{13}C NMR (100 MHz, $CDCl_3$) of compound **52**

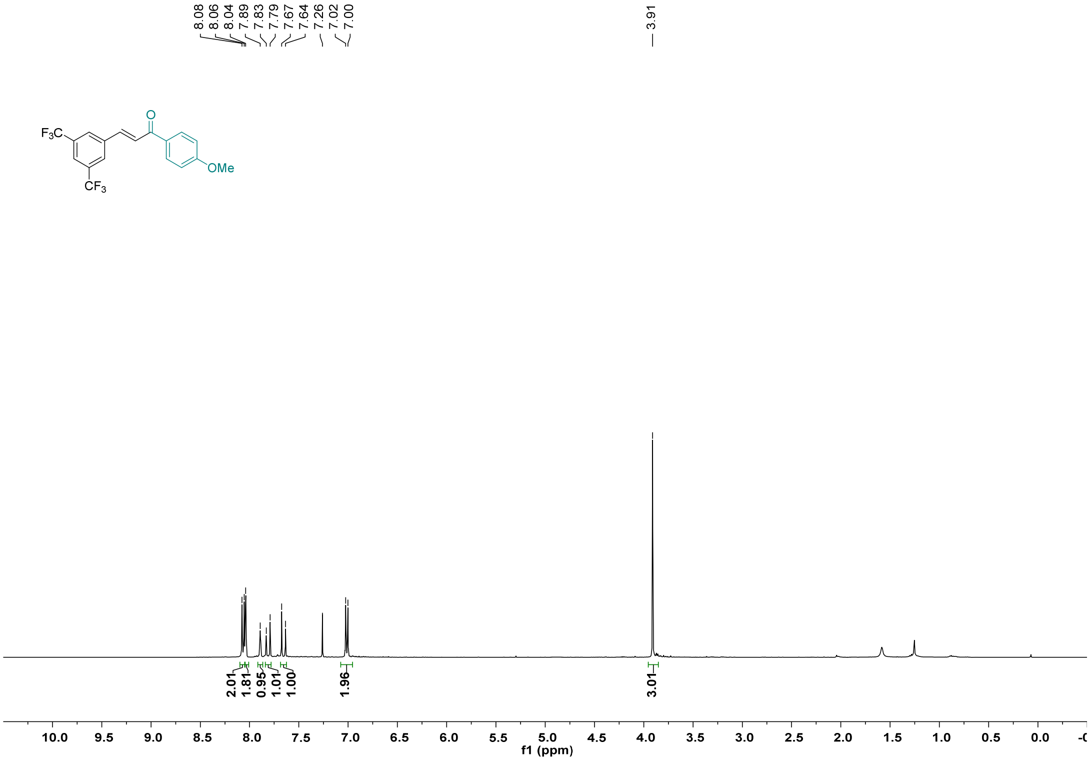

^{1}H NMR (400 MHz, $CDCl_3$) of compound **53**

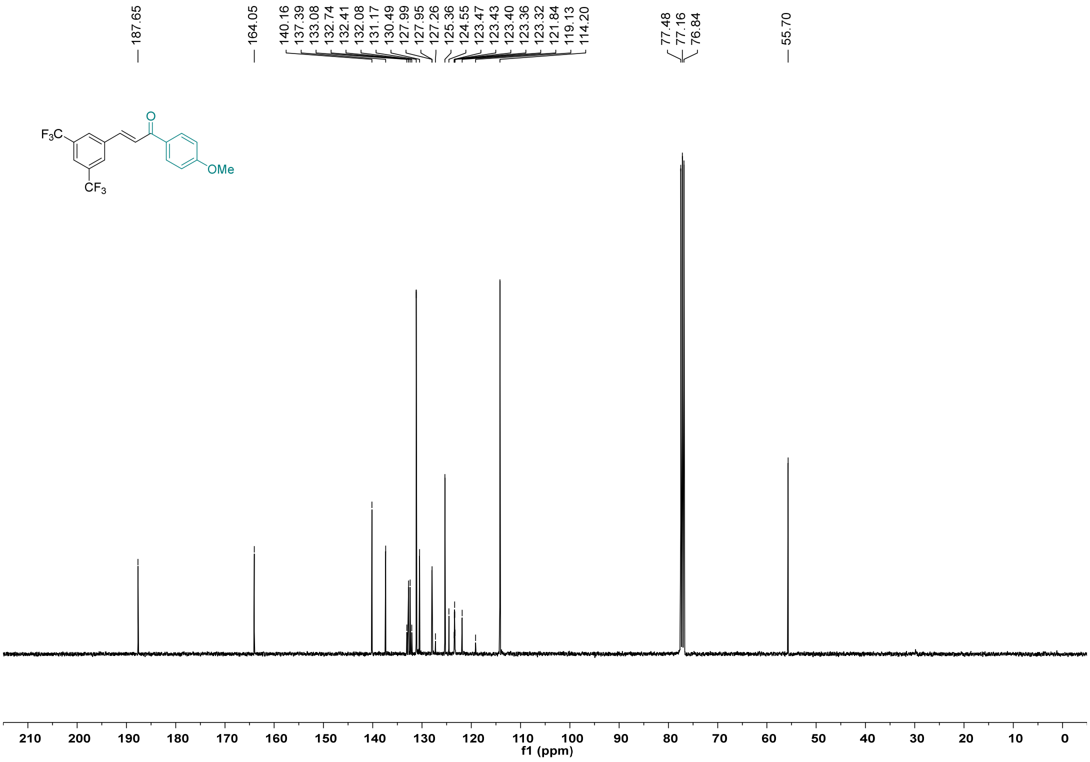

^{13}C NMR (100 MHz, $CDCl_3$) of compound **53**

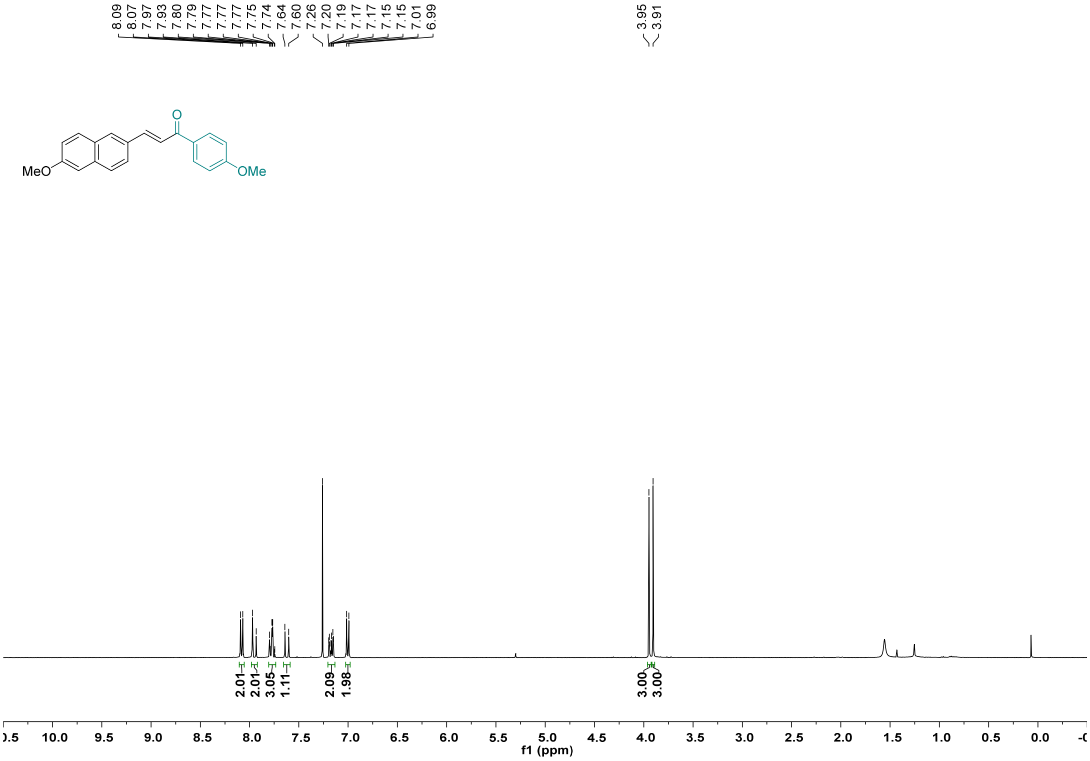

^{1}H NMR (400 MHz, $CDCl_3$) of compound **54**

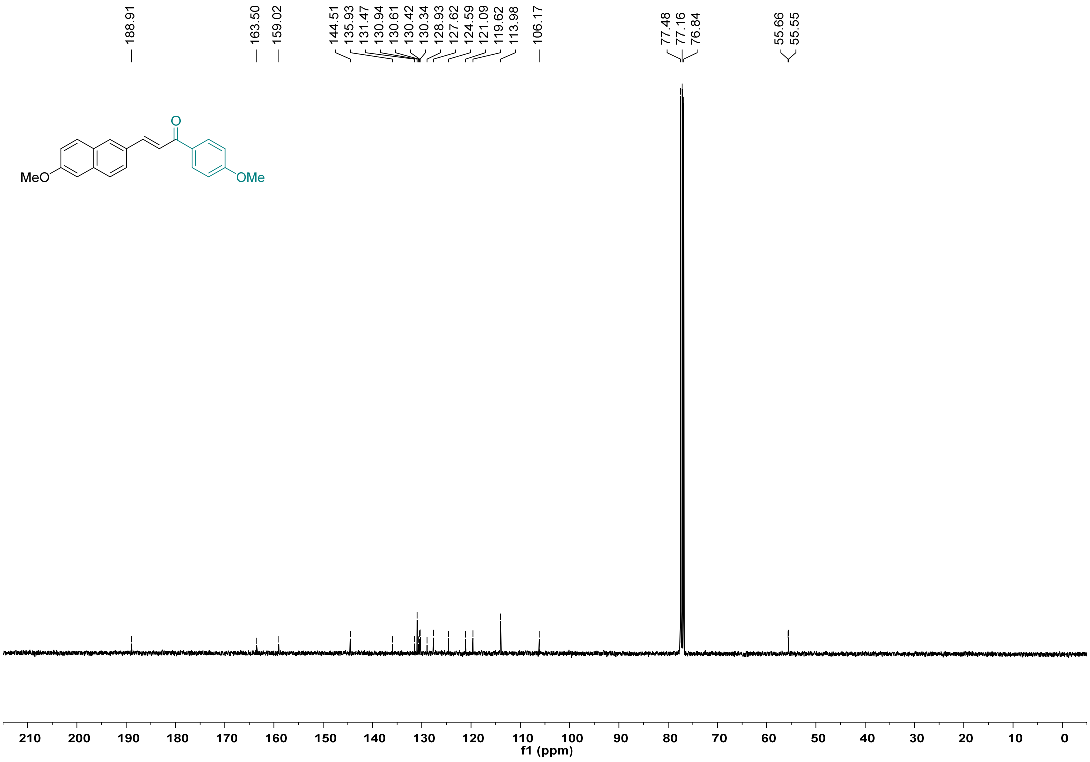

^{13}C NMR (100 MHz, $CDCl_3$) of compound **54**

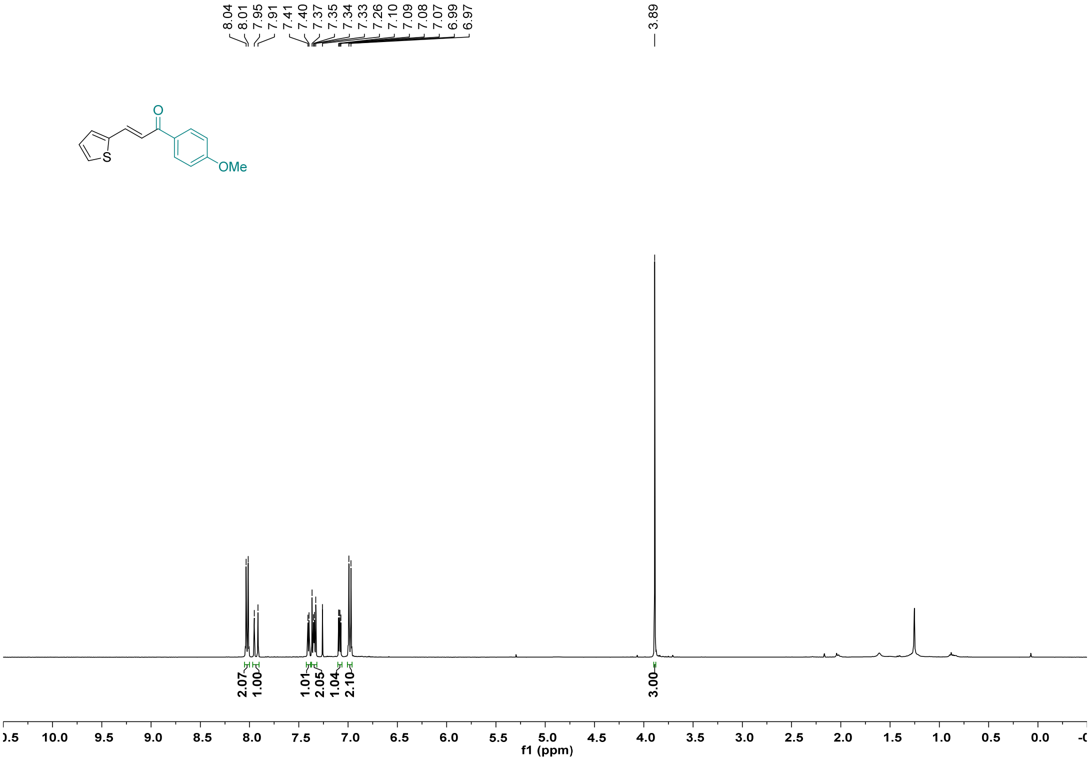

^{1}H NMR (400 MHz, $CDCl_3$) of compound **55**

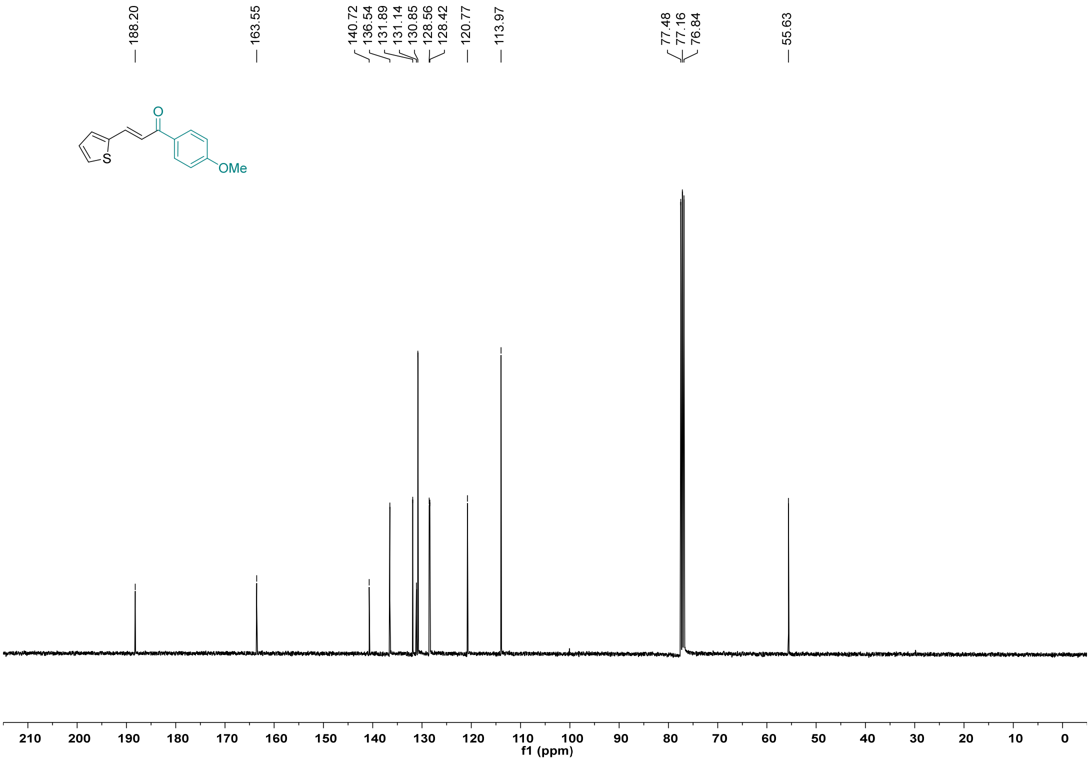

^{13}C NMR (100 MHz, $CDCl_3$) of compound **55**

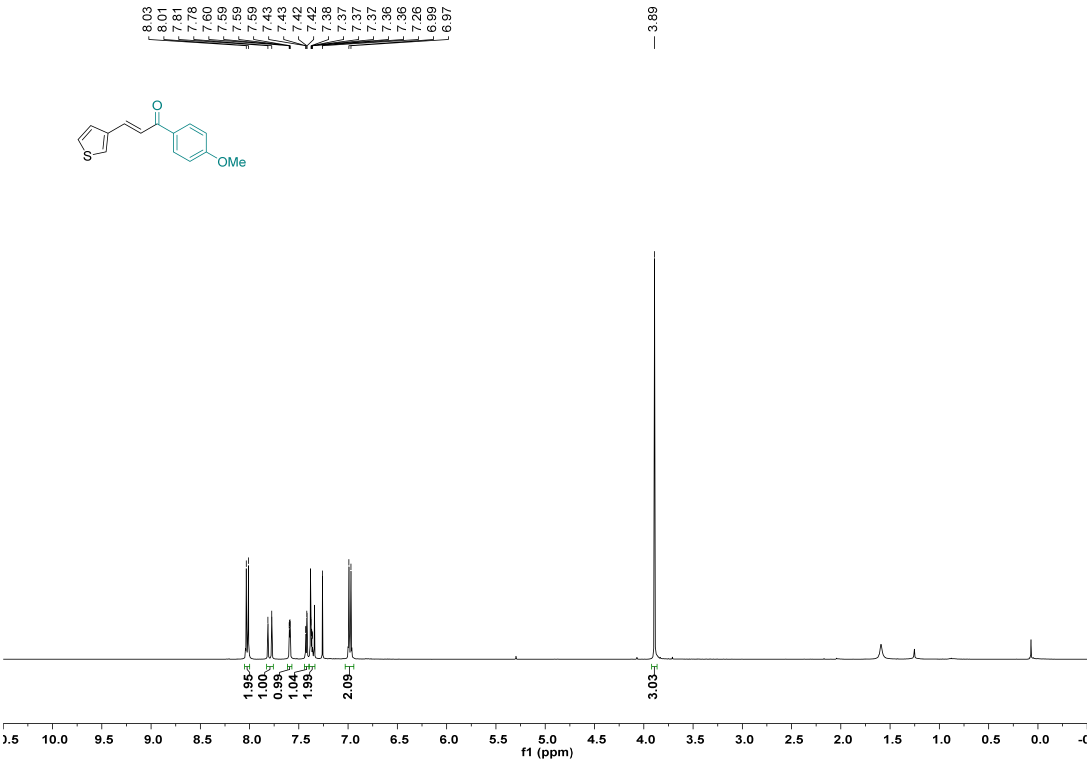

^{1}H NMR (400 MHz, $CDCl_3$) of compound **56**

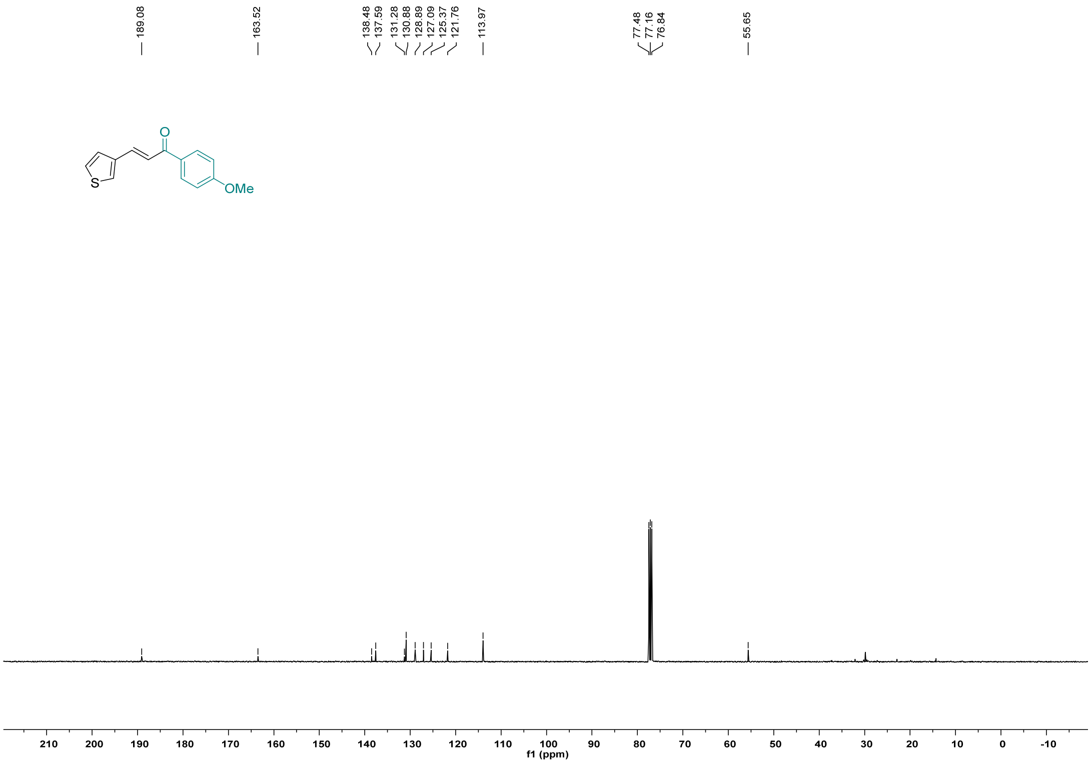

^{13}C NMR (100 MHz, $CDCl_3$) of compound **56**

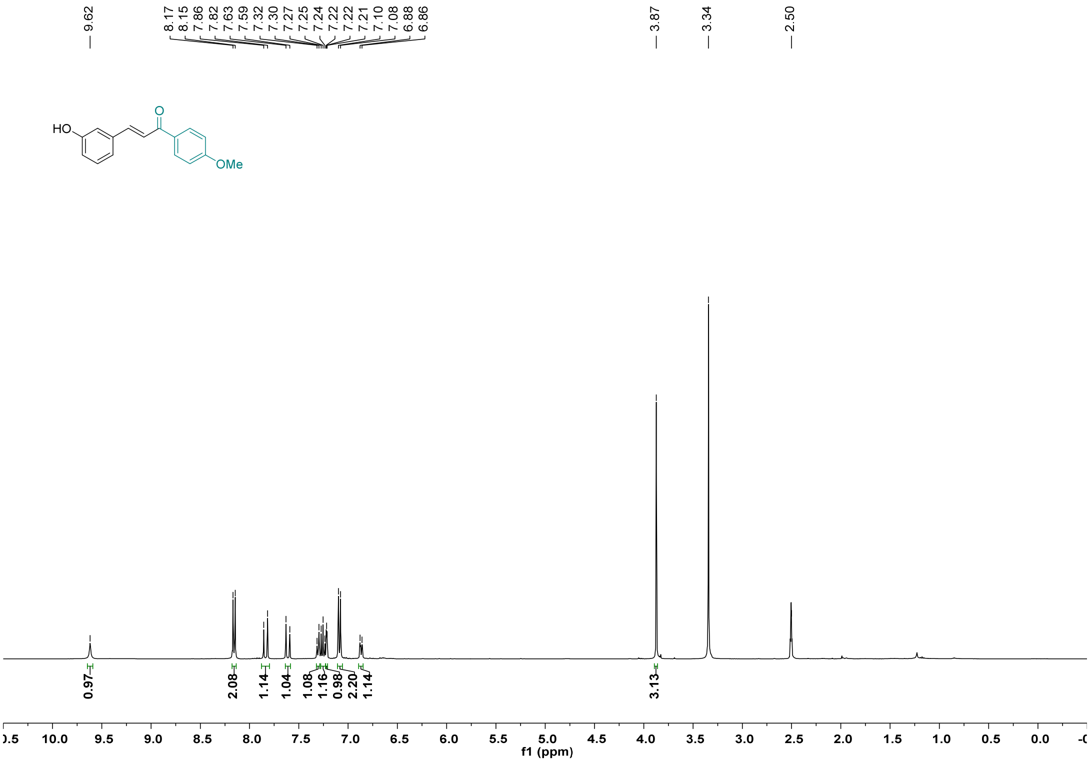

^{1}H NMR (400 MHz, DMSO-d_6) of compound **57**

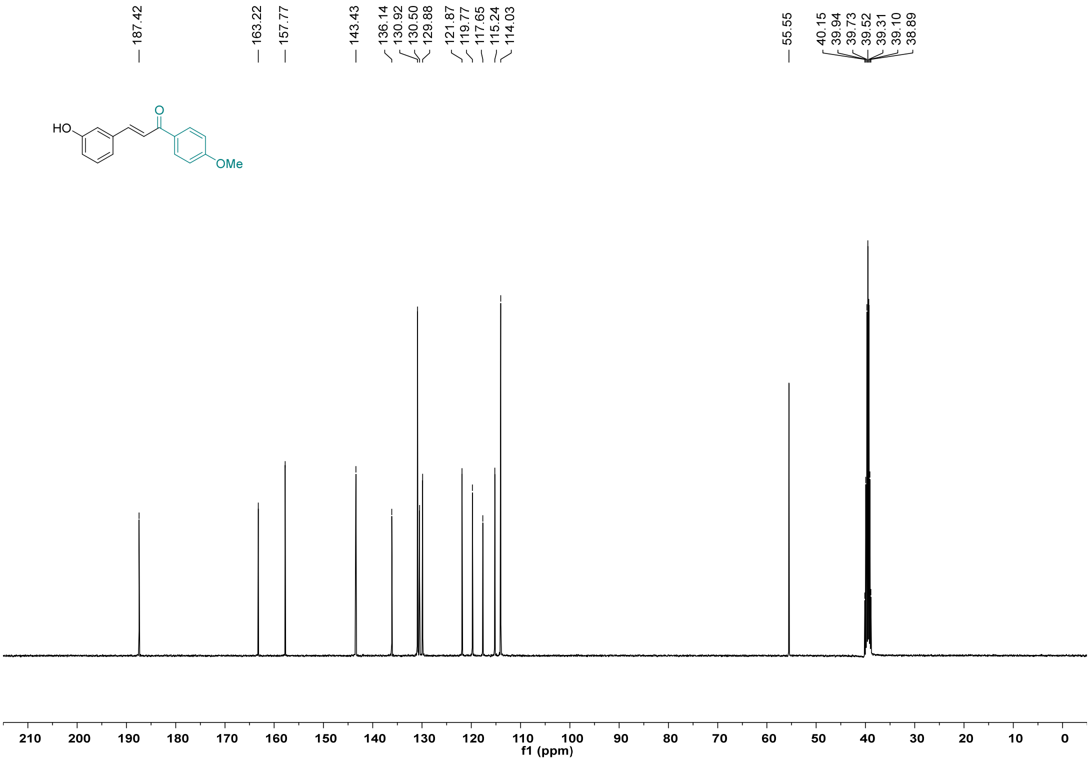

^{13}C NMR (100 MHz, DMSO-d_6) of compound **57**

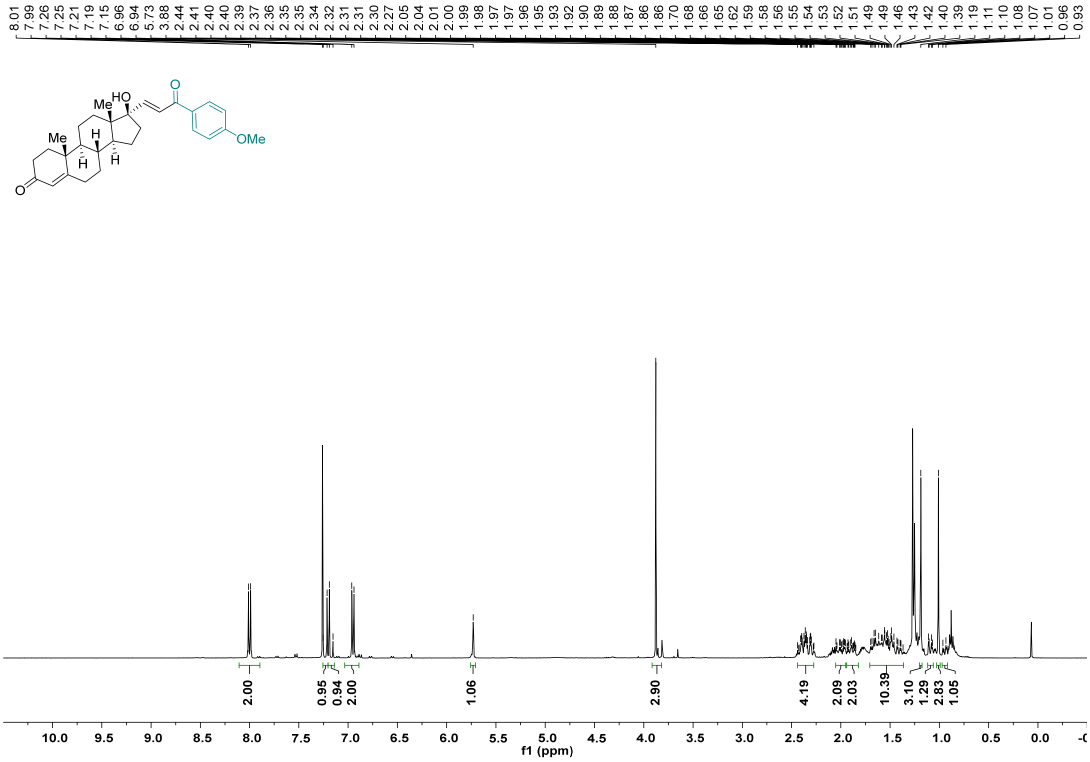

^{1}H NMR (400 MHz, DMSO-d_6) of compound **58**

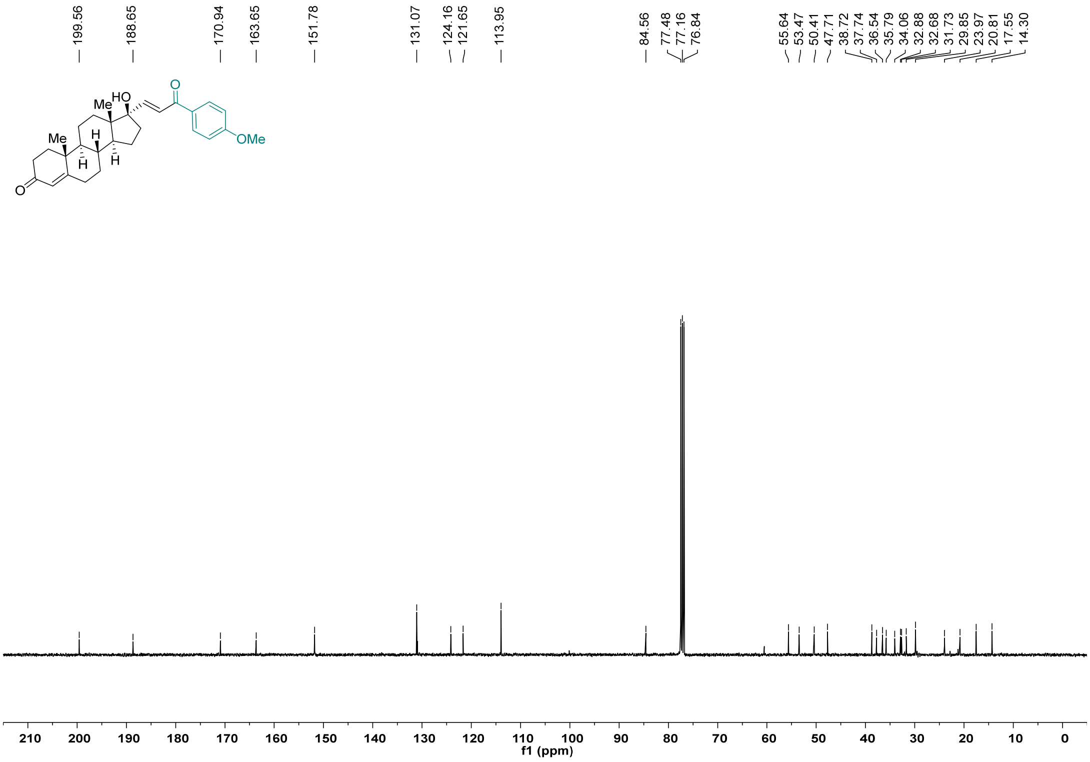

^{13}C NMR (100 MHz, DMSO-d_6) of compound **58**

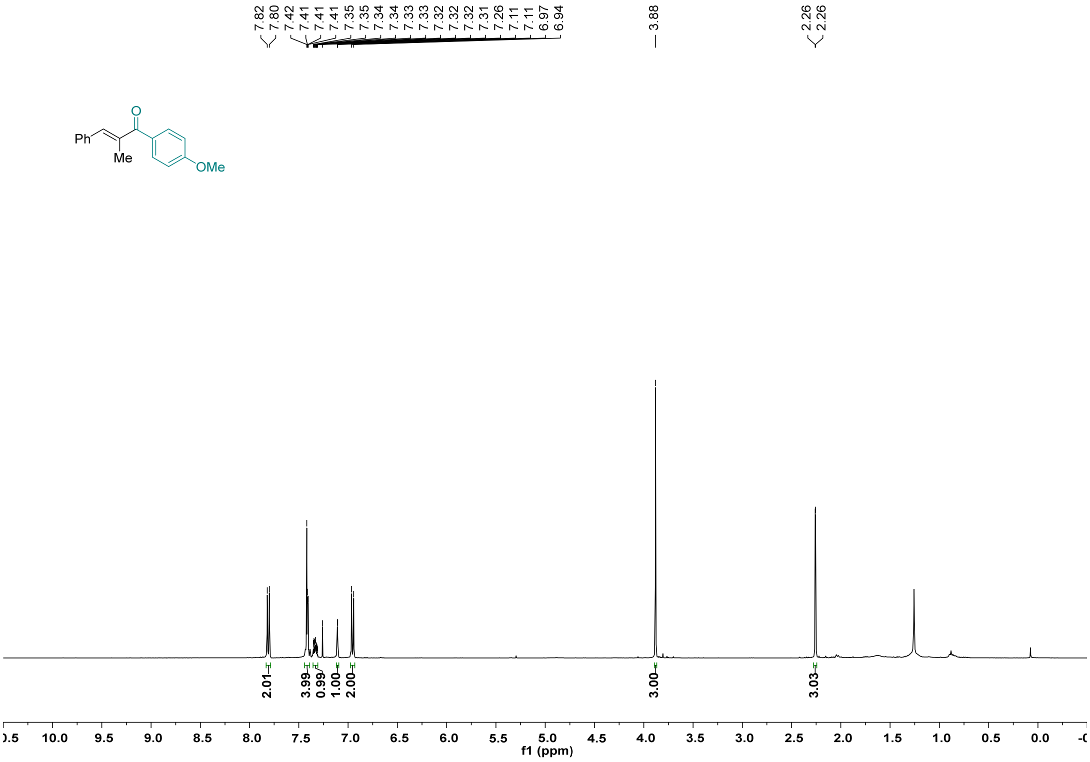

^{1}H NMR (400 MHz, $CDCl_3$) of compound **59**

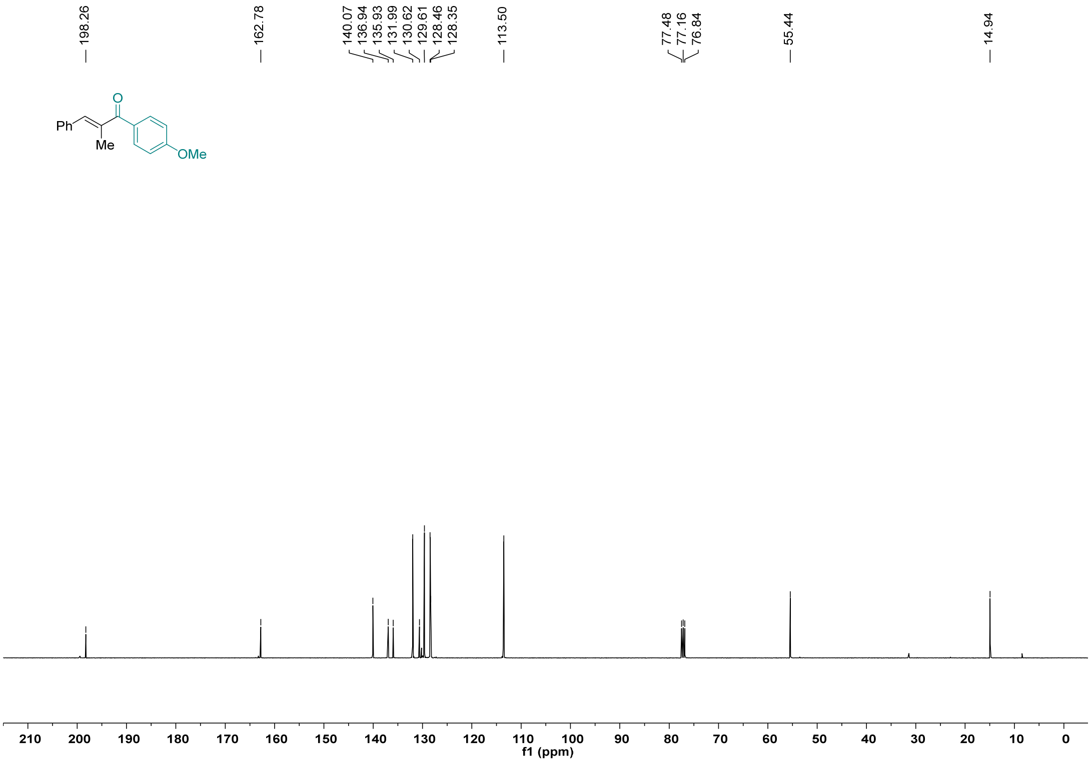

^{13}C NMR (100 MHz, $CDCl_3$) of compound **59**

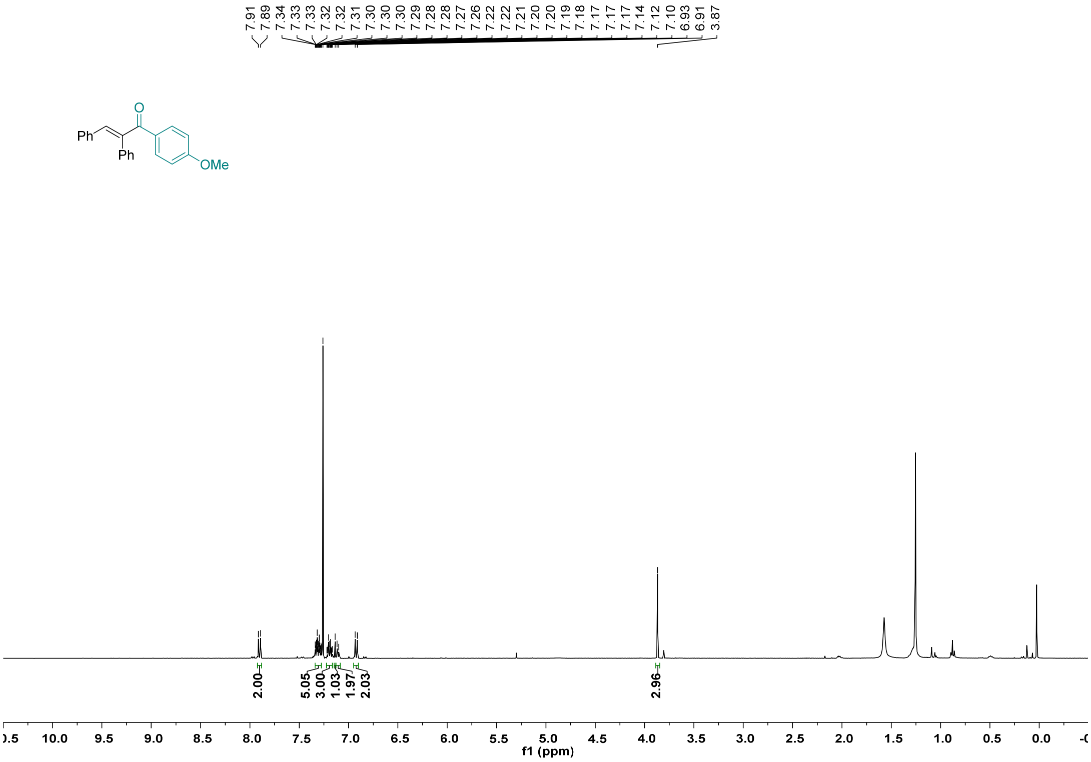

^{1}H NMR (400 MHz, $CDCl_3$) of compound **60**

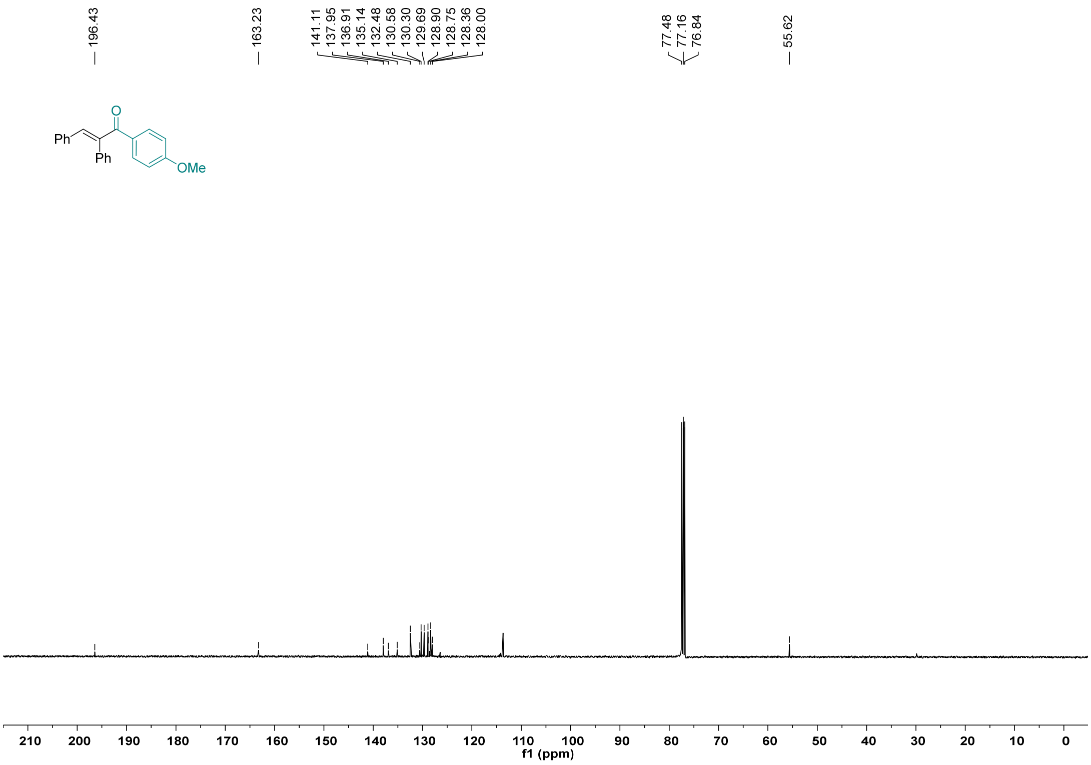

^{13}C NMR (100 MHz, CDCl$_3$) of compound **60**

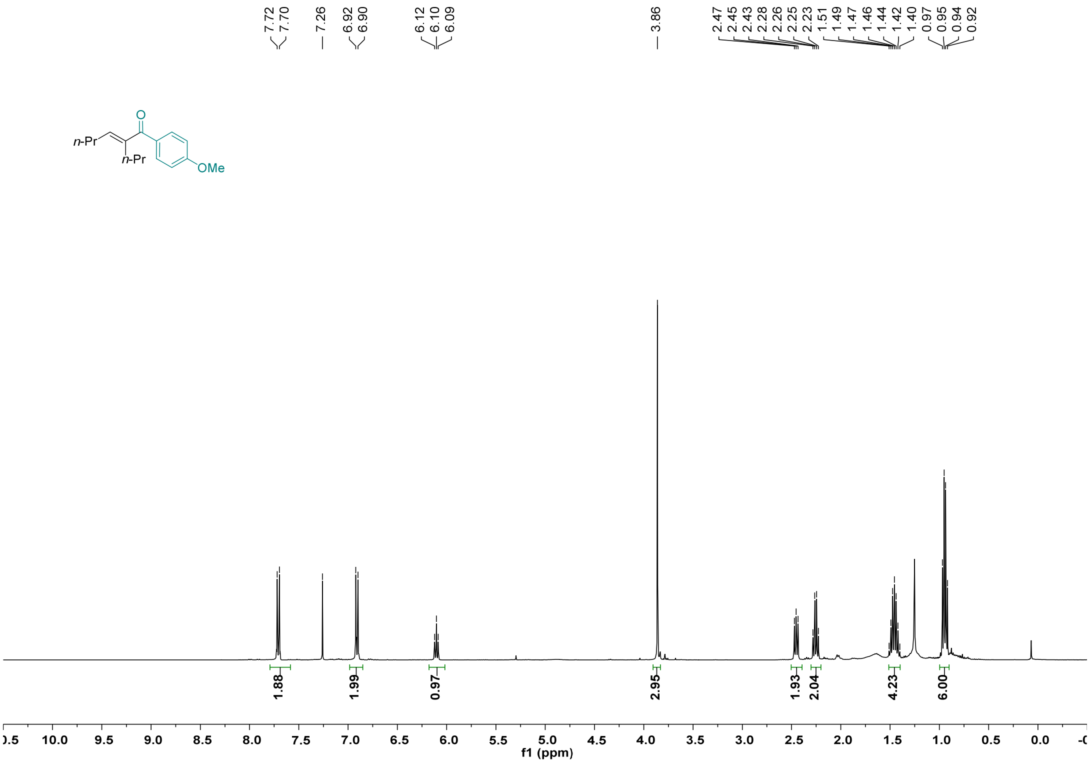

^{1}H NMR (400 MHz, CDCl$_3$) of compound **61**

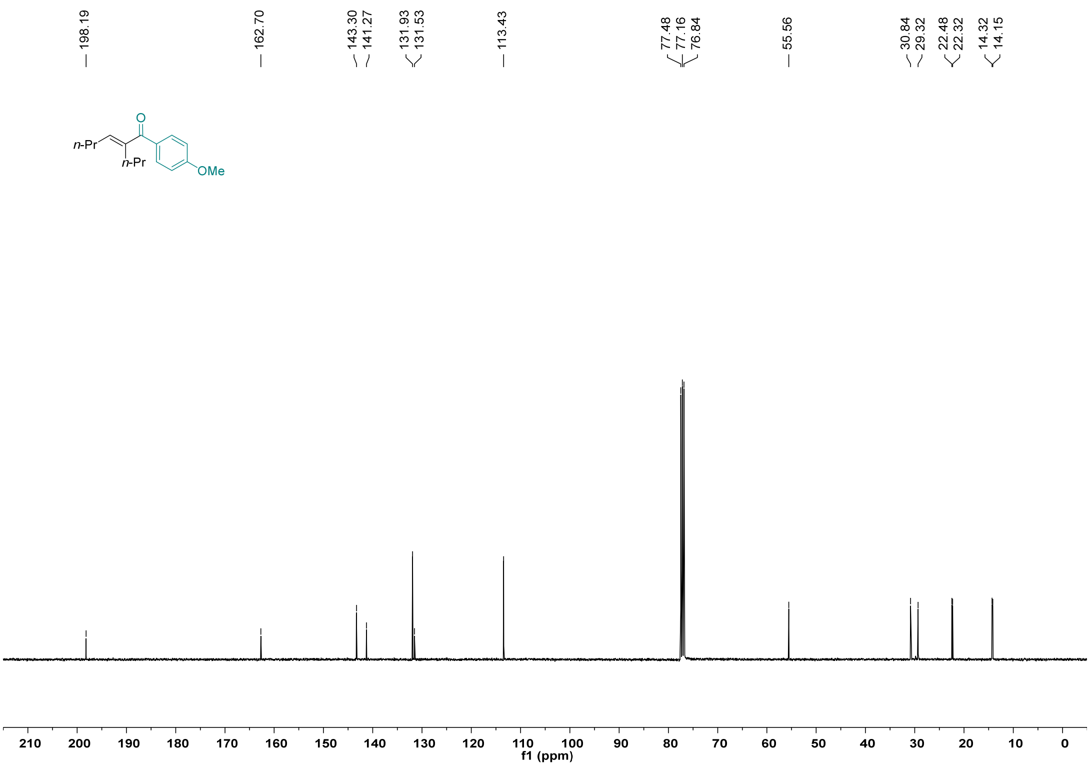

^{13}C NMR (100 MHz, $CDCl_3$) of compound **61**

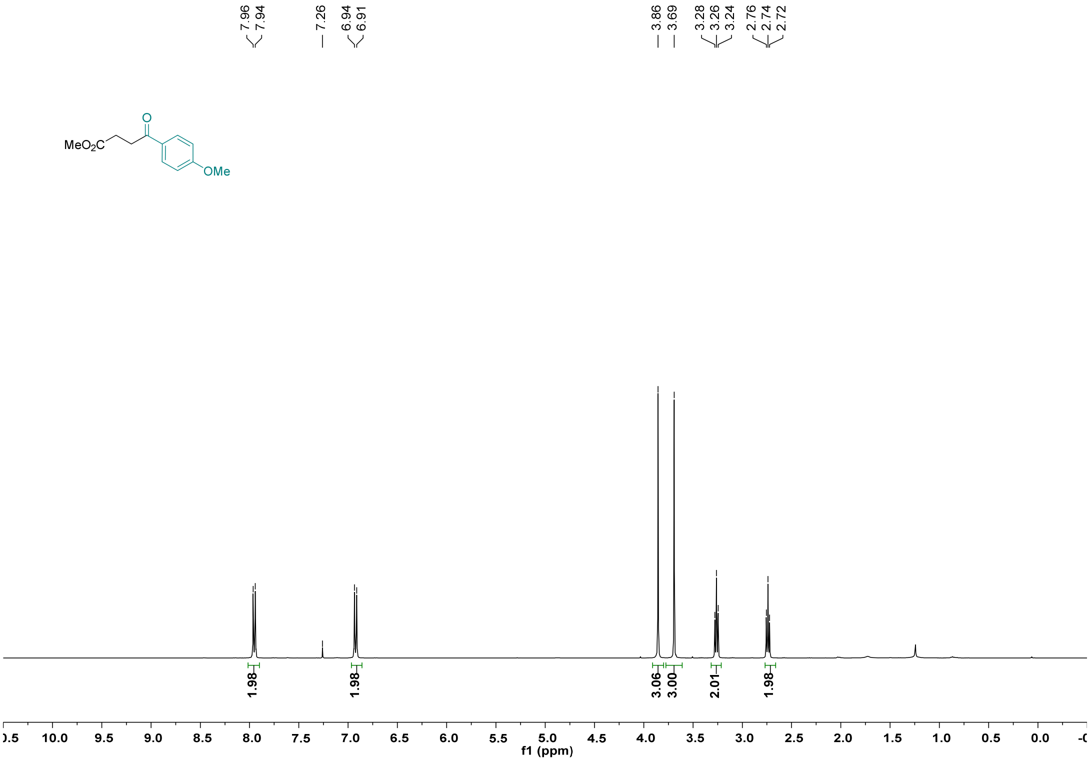

^{1}H NMR (400 MHz, $CDCl_3$) of compound **62**

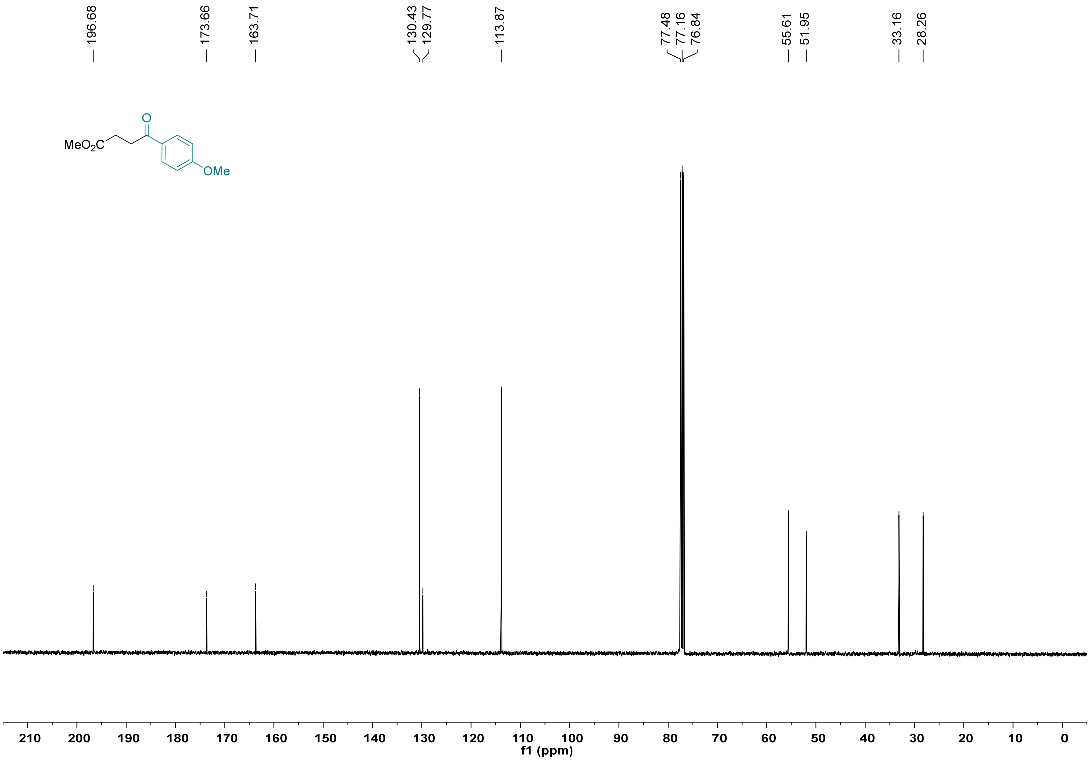

^{13}C NMR (100 MHz, $CDCl_3$) of compound **62**

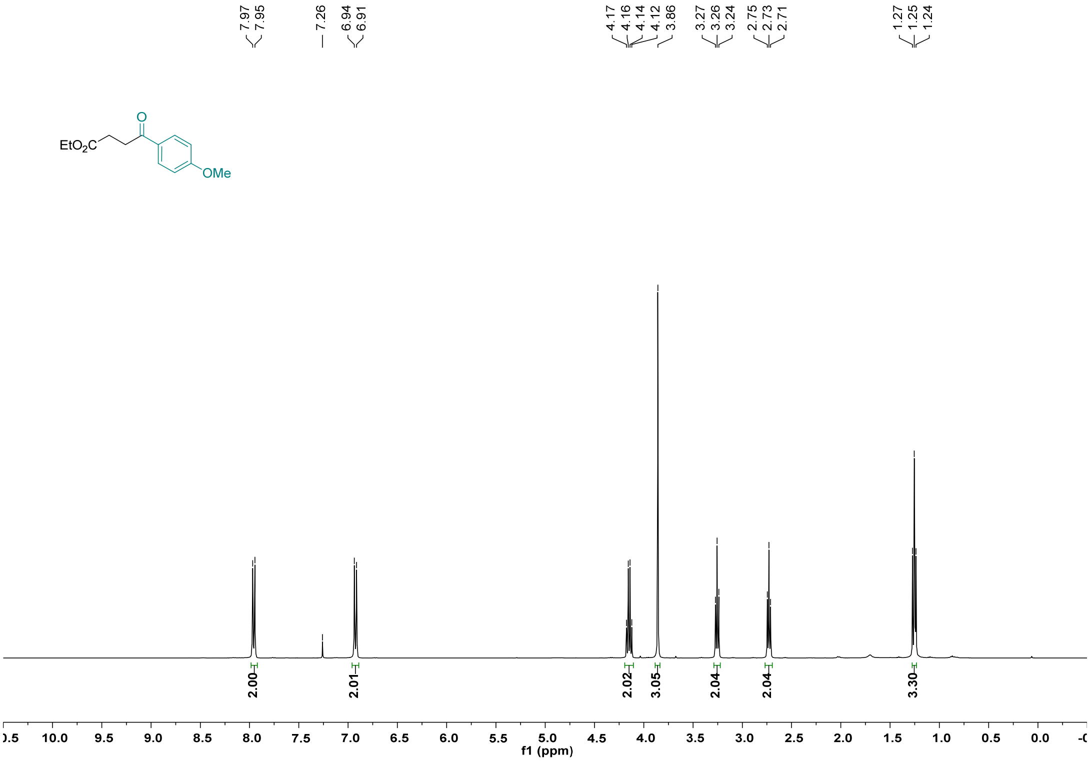

^{1}H NMR (400 MHz, $CDCl_3$) of compound **63**

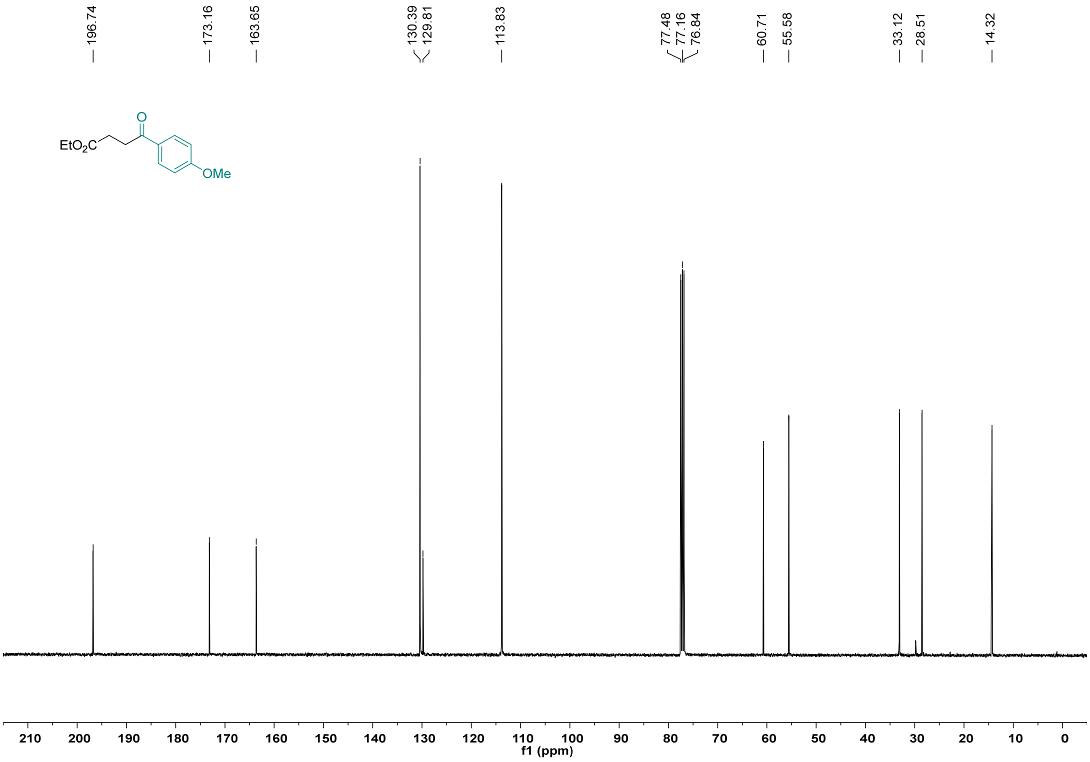

^{13}C NMR (100 MHz, $CDCl_3$) of compound **63**

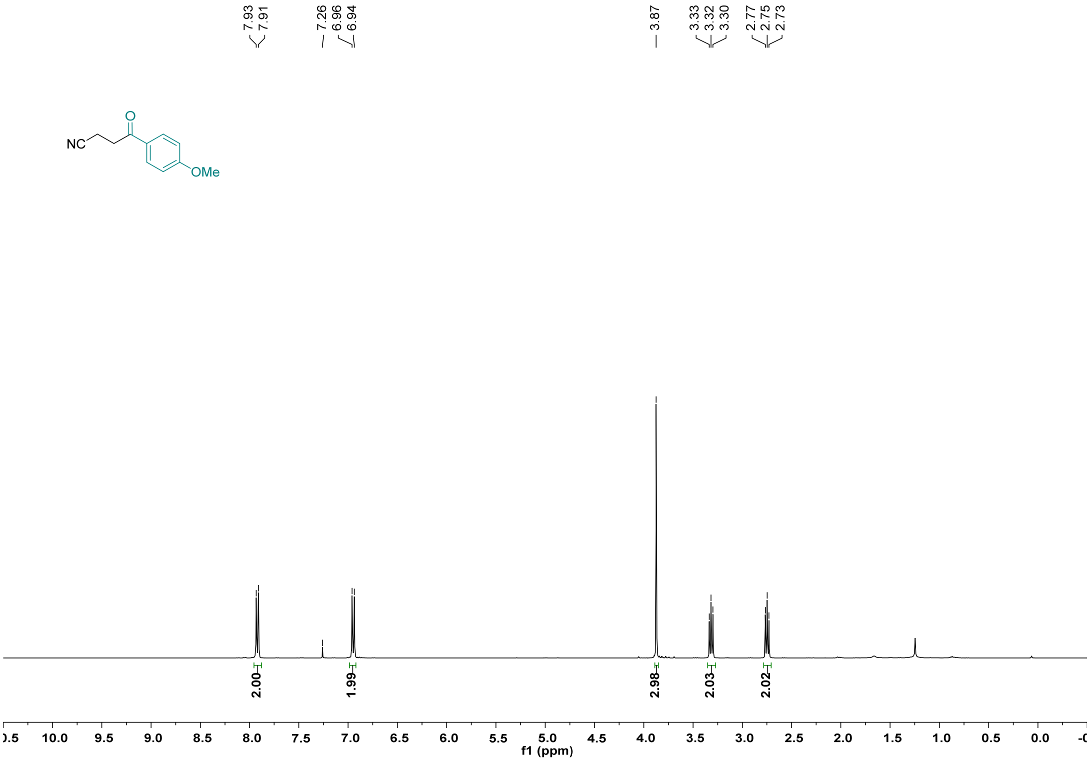

^{1}H NMR (400 MHz, $CDCl_3$) of compound **64**

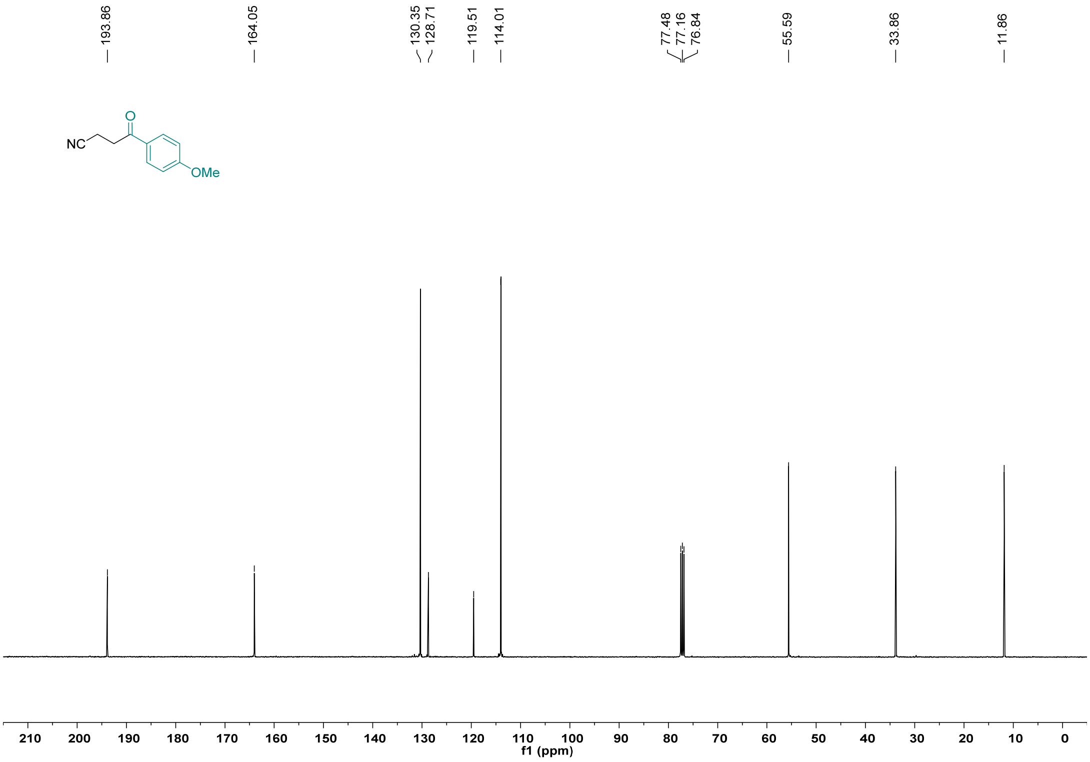

^{13}C NMR (100 MHz, $CDCl_3$) of compound **64**

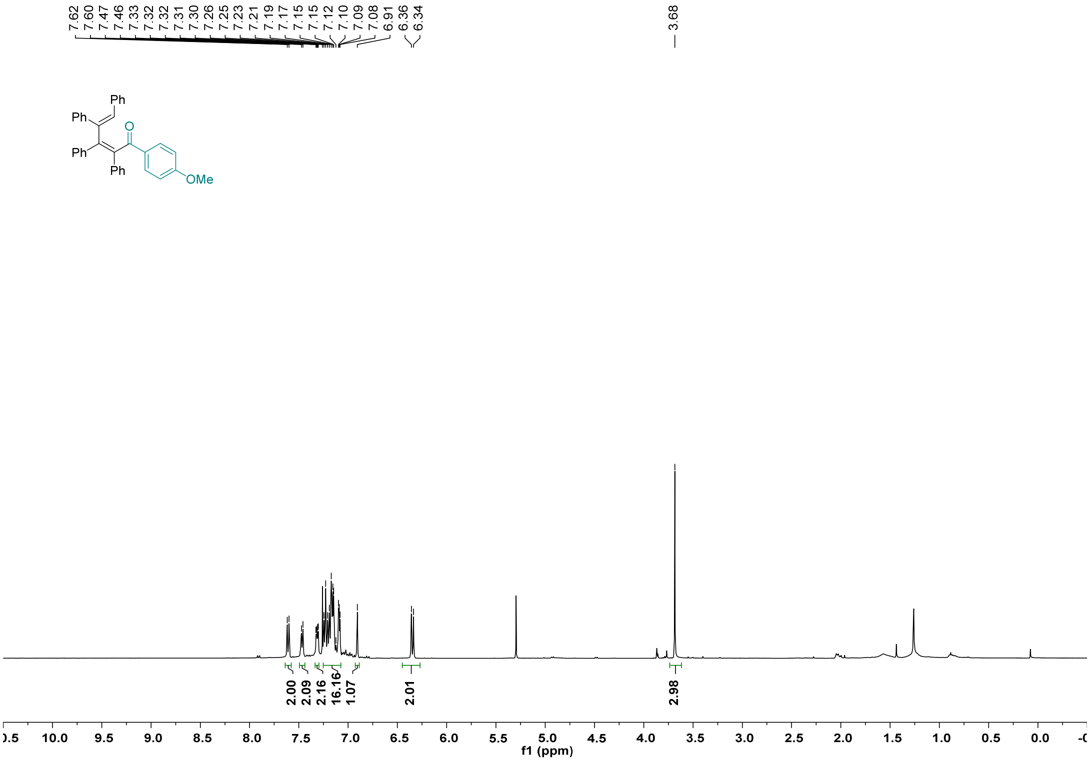

^{1}H NMR (400 MHz, $CDCl_3$) of compound **65**

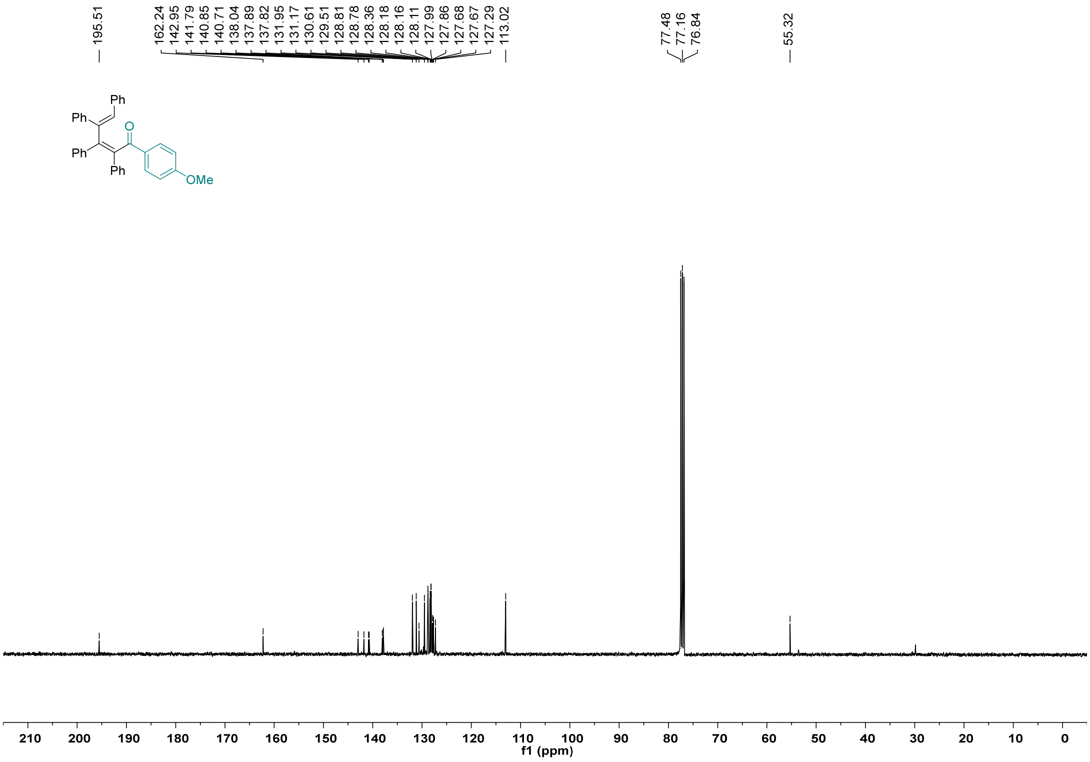

^{13}C NMR (100 MHz, $CDCl_3$) of compound **65**

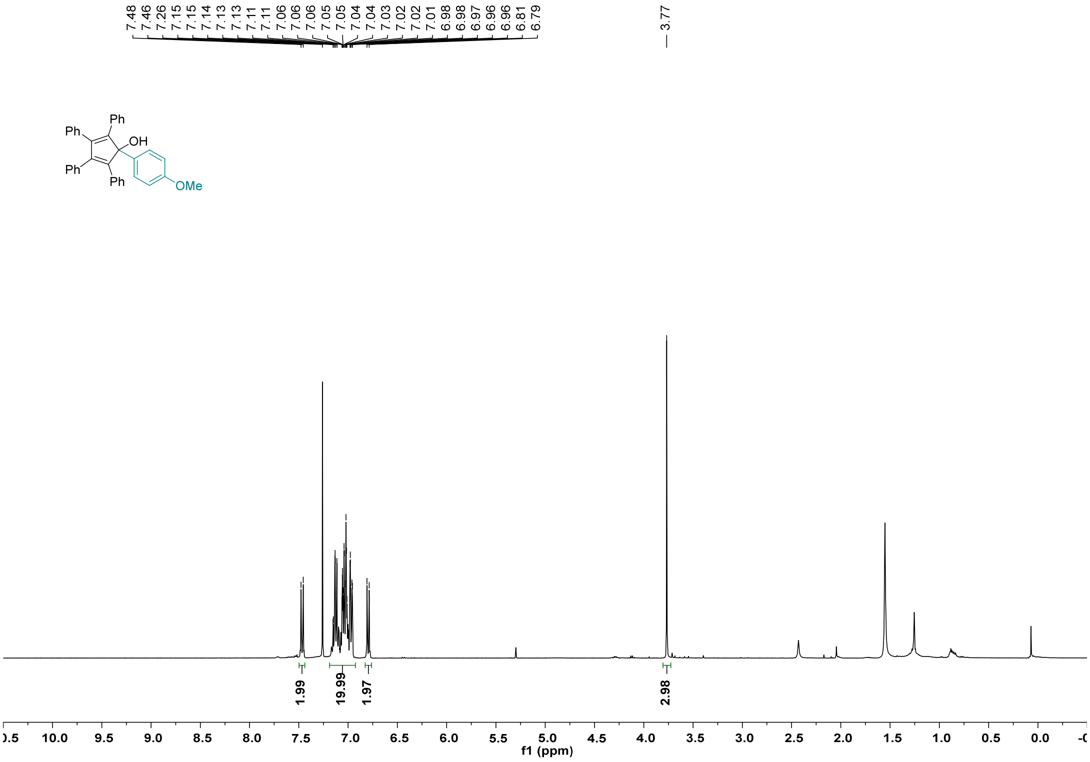

^{1}H NMR (400 MHz, $CDCl_3$) of compound **66**

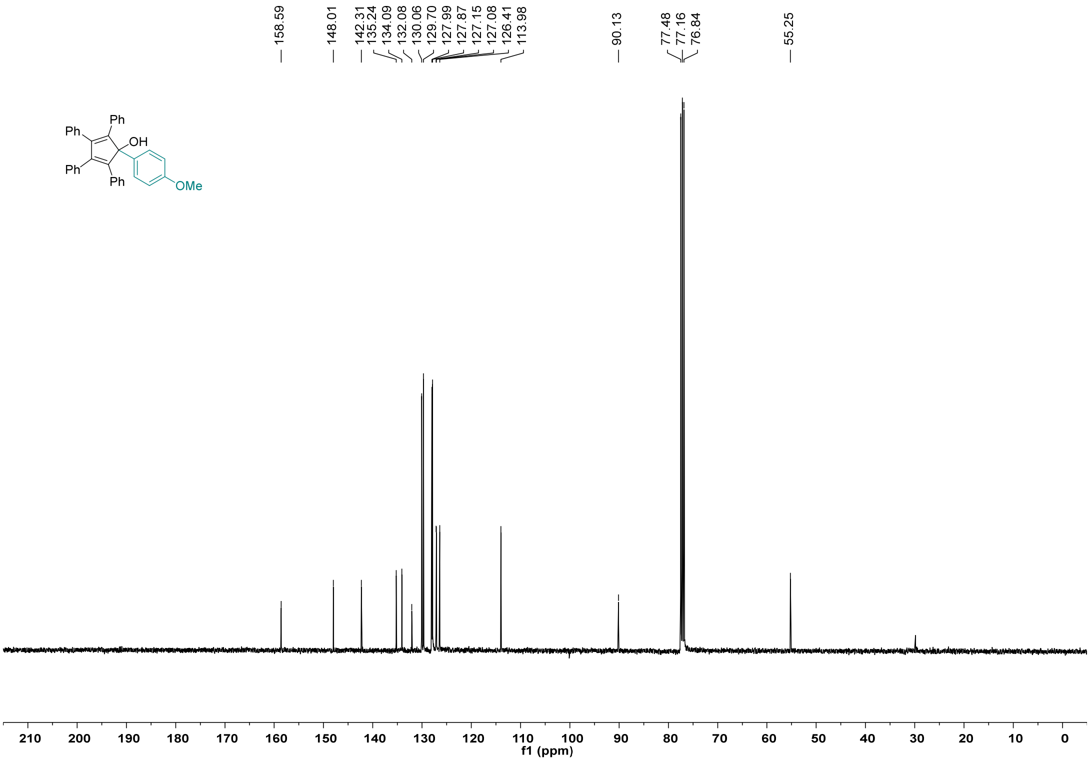

^{13}C NMR (100 MHz, $CDCl_3$) of compound **66**

X. Supplementary References

1. Zhu, F., Rodriguez, J., O'Neill, S. & Walczak, M. A. *ACS Cent. Sci.* **4**, 1652–1662 (2018).
2. Wang, J., Cary, B. P., Beyer, P. D., Gellman, S. H. & Weix, D. J. *Angew. Chem. Int. Ed.* **58**, 12081–12085 (2019).
3. Zaidi, S. H. H., Muthukumaran, K., Tamaru, S. & Lindsey, J. S. *J. Org. Chem.* **69**, 8356–8365 (2004).
4. Ociepa, M., Baka, O., Narodowiec, J. & Gryko, D. *Adv. Synth. Catal.* **359**, 3560–3565 (2017).
5. Dogutan, D. K., Zaidi, S. H. H., Thamyongkit, P. & Lindsey, J. S. *J. Org. Chem.* **72**, 7701–7714 (2007).
6. Rao, P. D., Dhanalekshmi, S., Littler, B. J. & Lindsey, J. S. *J. Org. Chem.* **65**, 7323–7344 (2000).
7. Dogutan, D. K., Ptaszek, M. & Lindsey, J. S. *J. Org. Chem.* **73**, 6187–6201 (2008).
8. Amari, T., Funahashi, S. & Tanaka, M. *Inorg. Chem.* **27**, 3368–3372 (1988).
9. Venyaminov, S. Y. *Anal. Biochem.* **248**, 234–245 (1997).
10. Bew, S. P., Hiatt-Gipson, G. D., Lovell, J. A. & Poullain, C. *Org. Lett.* **14**, 456–459 (2012).
11. Sheldrick, G. M. *Acta Cryst.* **A64**, 112–122 (2008).
12. Rana, N. K., Unhale, R. & Singh, V. K. *Tetrahedron Lett.* **53**, 2121–2124 (2012).
13. Lee, K., Gallagher, W. P., Toskey, E. A., Chong, W. & Maleczka, R. E. *J. Organomet. Chem.* **691**, 1462–1465 (2006).
14. Yu, M., Li, G., Wang, S. & Zhang, L. *Adv. Synth. Catal.* **349**, 871–875 (2007).
15. Jun, C.-H., Lee, H., Hong, J.-B. & Kwon, B.-I. *Angew. Chem. Int. Ed.* **41**, 2146–2147 (2002).
16. Onishi, Y., Yoneda, Y., Nishimoto, Y., Yasuda, M. & Baba, A. *Org. Lett.* **14**, 5788–5791 (2012).
17. Kan, J., Zhang, M., Zhang, X., Lou, X., Shang, Y., Xu, B., Yang, F. & Su, W. *Chem. Eur. J.* **25**, 15233–15238 (2019).
18. Li, J., Zhang, J., Li, M., Zhang, C., Yuan, Y. & Liu, R. *Chem. Commun.* **55**, 2348–2351 (2019).
19. Mahler, C., Müller, U., Müller, W. M., Enkelmann, V., Moon C., Brunklaus, G., Zimmermann, H. & Höger, S. *Chem. Commun.* **39**, 4816–4818 (2008).
20. Vellakkaran, M., Andappan, M. M. S. & Nagaiah, K. *RSC Adv.* **4**, 45490–45494 (2014).
21. Choi, J. W., Jang, B. K., Cho, N.-C., Park, J.-H., Yeon, S. K., Ju, E. J., Lee, Y. S., Han, G., Pae, A. N., Kim, D. J. & Park, K. D. *Bioorg. Med. Chem.* **23**, 6486–6496 (2015).
22. Lator, A., Gaillard, S., Poater, A. & Renaud, J.-L. *Chem. Eur. J.* **24**, 5770–5774 (2018).
23. HanLee, I.-S., Jeon, H. J. & Lee, C.-K. *Bull. Korean Chem. Soc.* **32**, 687–692 (2011).
24. Songthammawat, P., Wangngae, S., Matsumoto, K., Duangkamol, C., Ruchirawat, S. & Ploypradith, P. *J. Org. Chem.* **83**, 5225–5241 (2018).
25. Wang, C., Morimoto, T., Kanashiro, H., Tanimoto, H., Nishiyama, Y., Kakiuchi, K. & Artok, L. *Synlett* **25**, 1155–1159 (2014).
26. Kuş, M., Artok, Ö. A., Ziyanak, F. & Artok, L. *Synlett* **17**, 2587–2592 (2008).

27. Sutar, R. L., Sen, S., Eivgi, O., Segalovich, G., Schapiro, I., Reany, O. & Lemcoff, N. G. *Chem. Sci.* **9**, 1368–1374 (2018).
28. Jiang, J., Liu, J., Yang, L., Shao, Y., Cheng, J., Bao, X. & Wan, X. *Chem. Commun.* **51**, 14728–14731 (2015).
29. Ishikawa, T., Mizuta, T., Hagiwara, K., Aikawa, T., Kudo, T. & Saito, S. *J. Org. Chem.* **68**, 3702–3705 (2003).
