# Supplementary material for: Synthesis of α,β-unsaturated ketones through nickel-catalysed aldehyde-free hydroacylation of alkynes
Source: Commun Chem. 2022 Feb 3;5:13. doi: 10.1038/s42004-022-00633-3 (PMC9814684; doi:10.1038/s42004-022-00633-3)
Supplement: Supplementary file 4 — Description of Additional Supplementary Files [file 42004_2022_633_MOESM4_ESM.pdf]

Description of Additional Supplementary Files

File Name: Supplementary Data 1

Description: The CIF data for compound 27

File Name: Supplementary Data 2

Description: The CIF data for compound 66
